# Re-annotation of presumed noncoding disease/trait-associated genetic variants by integrative analyses

**DOI:** 10.1038/srep09453

**Published:** 2015-03-30

**Authors:** Geng Chen, Dianke Yu, Jiwei Chen, Ruifang Cao, Juan Yang, Huan Wang, Xiangjun Ji, Baitang Ning, Tieliu Shi

**Affiliations:** 1 The Center for Bioinformatics and Computational Biology, Shanghai Key Laboratory of Regulatory Biology, the Institute of Biomedical Sciences and School of Life Sciences, East China Normal University, Shanghai 200241, China; 2 National Center for Toxicological Research, US Food and Drug Administration, Jefferson, AR 72079, USA; 3 Center for Pharmacogenomics, School of Pharmacy, Fudan University, Shanghai 201203, China

## Abstract

Using RefSeq annotations, most disease/trait-associated genetic variants identified by genome-wide association studies (GWAS) appear to be located within intronic or intergenic regions, which makes it difficult to interpret their functions. We reassessed GWAS-Associated single-nucleotide polymorphisms (herein termed as GASs) for their potential functionalities using integrative approaches. 8834 of 9184 RefSeq “noncoding” GASs were reassessed to have potential regulatory functionalities. As examples, 3 variants (rs3130320, rs3806932 and rs6890853) were shown to have regulatory properties in HepG2, A549 and 293T cells. Except rs3130320 as a known expression quantitative trait loci (eQTL), rs3806932 and rs6890853 were not reported as eQTLs in previous reports. 1999 of 9184 “noncoding” GASs were re-annotated to the promoters or intragenic regions using Ensembl, UCSC and AceView gene annotations but they were not annotated into corresponding regions in RefSeq database. Moreover, these GAS-harboring genes were broadly expressed across different tissues and a portion of them was expressed in a tissue-specific manner, suggesting that they could be functional. Collectively, our study demonstrates the benefits of using integrative analyses to interpret genetic variants and may help to predict or explain disease susceptibility more accurately and comprehensively.

To date, thousands of human disease/trait-associated single-nucleotide polymorphisms (SNPs) have been reported by GWAS^1^; however, they were mainly annotated with RefSeq genes^2^,^3^,^4^,^5^,^6^,^7^,^8^. With the development of sequencing technologies and bioinformatics algorithms, several different databases (e.g. Ensembl^9^, GENCODE^10^ [equivalent to Ensembl], UCSC^11^ and AceView^12^) also provided their own gene annotations in addition to RefSeq^2^. Although recent genome/exome sequencing studies including TCGA (The Cancer Genome Atlas)^13^ have used RefSeq/Ensembl gene annotations, almost no study that integrated different gene databases to comprehensively annotate identified genetic variants was available from the literature. Accurate functional annotation of variants to the proper regions of the genome is crucial for understanding the biological significances of GASs in human disorders and in unraveling the underlying mechanisms of associated diseases/traits^14^,^15^. Variants in the 5′ untranslated regions (UTRs) may influence the promoter activity of a gene^16^, whereas the variants in the 3′ UTRs may change the mRNA degradation rate mediated by microRNAs and RNA-binding proteins (RBPs)^17^. Variants located at splice junctions may alter the splicing patterns of genes^18^, On the other hand, coding variants could contribute to altered gene functions in different ways depending on the characteristics of variants: non-synonymous variants can induce protein function changes and synonymous variants may alter the translation efficiency^19^,^20^.

In terms of assessing variant functions, many factors may influence the interpretation, understanding and utilization of GWAS data: (i) previously reported GASs were mainly mapped to the intronic or intergenic regions of the RefSeq database that contains a limited number of genes and transcripts^1^; (ii) many human genes still remain un-annotated due to the limitation of sequencing technologies and inconsistencies in the consensus human genome^21^; (iii) mammalian genes often encode multiple distinct isoforms through alternative splicing/transcription^22^,^23^; (iv) the variabilities among different genome databases of RefSeq^2^, Ensembl^9^, UCSC^11^ and AceView^12^ introduce disparities in gene annotations^24^; and finally, (v) many noncoding genetic variants may have regulatory roles^25^. Therefore, accurate functional assessment of variants is crucial for understanding the biological significances of GASs in associated diseases/traits^14^,^15^.

Remarkably, multiple human gene annotations from distinct databases provide an opportunity to obtain a more complete human gene set and enable us to perform a more comprehensive evaluation of functional genetic variations^24^. On the other hand, the developments of in silico tools for better annotation of regulatory elements allow us to further interrogate the regulatory potential of GASs^26^,^27^,^28^,^29^. In addition, RNA-Seq technologies provide unprecedented opportunities to identify and characterize the expressed genes/transcripts. Some prior studies have sought to characterize the function of noncoding GASs^25^,^30^,^31^,^32^. However, these studies did not combine different gene/regulatory annotation databases, high-throughput sequencing data and experimental assay to annotate noncoding genetic variants integratively. Here, we reevaluated the GASs that were previously annotated at RefSeq intronic and intergenic regions using integrative approaches to explore their various biological features, including regulatory possibility, coding capability and alternative splicing potential. Our integrative strategy re-annotated a large portion of RefSeq noncoding GASs to the promoter or intragenic regions of Ensembl, UCSC and AceView human genes, or to diverse known or predicted regulatory regions.

## Results

### Annotating GASs with regulatory features

**Figure 1:**
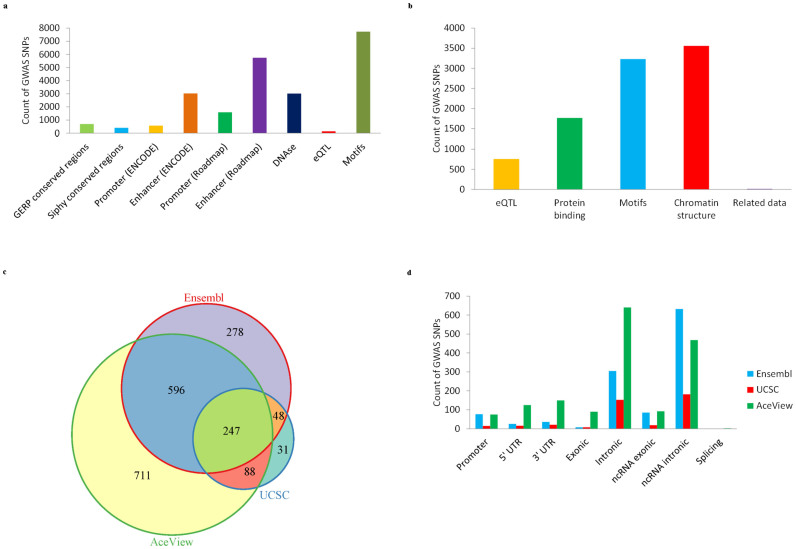
Lots of non-RefSeq-exonic GASs were annotated with diverse regulatory features by different gene annotations. (a) Count distribution of non-RefSeq-exonic GASs that were re-defined with different regulatory features using the HaploReg database. The enhancers catalogued in HaploReg included the experimentally validated and predicted ones. (b) Count distribution of non-RefSeq-exonic GASs that were re-defined with regulatory features using the RegulomeDB database. “Related Data” denotes the GASs that were supported by the data types of manually curated regions or validated functions in RegulomeDB. "Protein binding" represents the GAS category supported by ChIP-seq data, which usually refers to TF binding. (c) Intersection of the reassessed non-RefSeq-exonic GASs among Ensembl, UCSC and AceView annotations. (d) Count distribution of non-RefSeq-exonic GASs in different functional regions of Ensembl, UCSC and AceView genes.

For a whole set of 9828 GASs acquired from the National Human Genome Research Institute GWAS Catalog^1^, we filtered out the GASs that were located within exonic regions and splicing boundary areas (2 bp away from an exon/intron boundary) of RefSeq protein-coding genes, to obtain 9184 RefSeq noncoding GASs. Of the 9184 GASs, 8733 (95.09%) and 5853 (63.73%) were mapped to at least one regulatory features, including promoter or enhancer, regulatory motifs, DNase footprinting sites, expression quantitative trait loci (eQTL) and conserved sequences, using the HaploReg^26^ and RegulomeDB^27^ databases (Supplementary Table S1), respectively. Moreover, many of these GASs were predicted to be associated with multiple regulatory features (Fig. 1a and b), suggesting their dynamic regulatory functions. We also found that 700, 405 and 343 GASs (800 in total) are located in evolutionarily conserved regions, using GERP^33^ and SiPhy^34^,^35^ predictions from the HaploReg database, and using phastCons^36^ predictions of mammals from the UCSC database, respectively. In addition, 185 GASs fall into the regions of transcription factor binding sites (TFBS) in the UCSC database by conservation alignments among human, mouse and rat DNA sequences. These results indicate that the great majority of the GASs in RefSeq noncoding regions may have regulatory potential.

### Remapping noncoding GASs to Ensembl, UCSC and AceView gene annotations

Since the RefSeq human gene annotation is considered as incomplete, we used ANNOVAR^37^ to further characterize these 9184 RefSeq noncoding GASs with the Ensembl^9^, UCSC^11^ and AceView^12^ human gene annotations. Overall, 1169, 414 and 1642 GASs (1999 GASs in total, since some GASs could be re-annotated by two or three databases) were re-mapped to promoters (1 kb upstream of transcription start site) or intragenic regions of 959 Ensembl (307 protein-coding genes that are defined as genes annotated with at least one protein isoform), 336 UCSC (160 protein-coding) and 1364 AceView (887 protein-coding) genes, respectively (Fig. 1c, Supplementary Table S2, S3 and S4). The intragenic regions include 5′ UTRs, 3′ UTRs, CDSs (coding sequences), introns, splicing boundary areas, ncRNAs (non-coding RNAs) (Fig. 1d). None of these GASs was found to fall into splicing boundary areas of Ensembl and UCSC genes; however, 2 GASs were observed in alternative splicing boundaries of 2 AceView genes. Interestingly, 49 GASs that were associated with prostate, breast, colon cancer and other diseases/traits were remapped to the 8q24 region by Ensembl, UCSC or AceView annotations, while the 8q24 region is usually considered as a “gene desert” but a hotspot for multiple epithelial cancers^38^,^39^.

**Figure 2:**
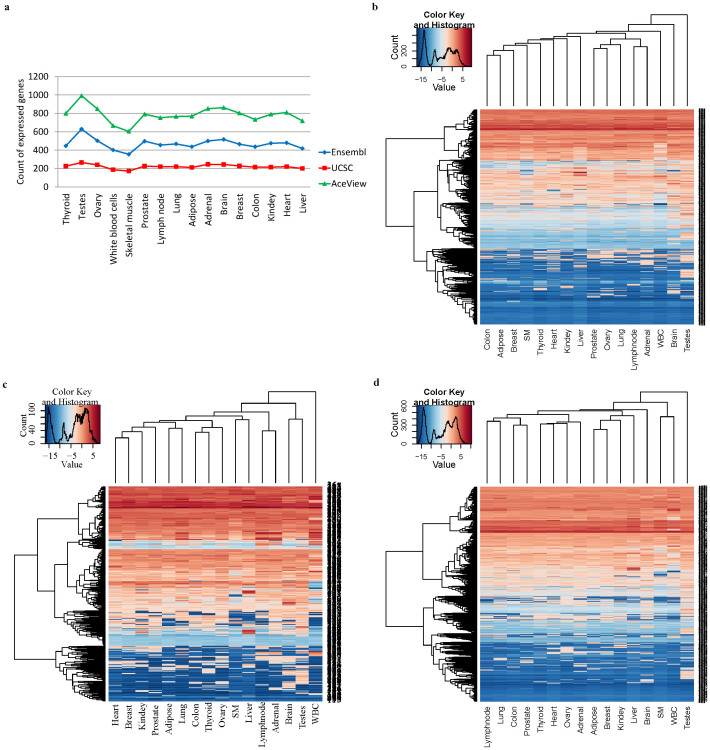
Expression profile of Ensembl, UCSC and AceView GAS-harboring genes. (a) Count distribution of GAS-harboring genes identified in the Ensembl, UCSC or AceView database. It illustrates the number of expressed GAS-harboring genes from the Ensembl, UCSC or AceView databases detected in 16 different human tissues. (b) Expression clustering for the 959 GAS-harboring genes by the Ensembl annotation in 16 different tissues. The unit for expression level is FPKM (fragments per kilobase of transcript per million mapped reads or read pairs) and is shown in ln scale. “SM” represents skeletal muscle and “WBC” denotes white blood cells. (c) Expression clustering for the 336 GAS harboring genes by the UCSC annotation. (d) Expression clustering for the 1364 GAS harboring genes by the AceView annotation.

To investigate whether these Ensembl, UCSC and AceView genes that harbor GASs were expressed, we further analyzed the RNA-Seq data of 16 distinct human tissues from the Illumina Human Body Map (HBM) 2.0 project. The RNA-Seq data of each tissue were first mapped to the human transcriptomes from Ensembl, UCSC and AceView databases using Bowtie^40^. Next, we quantified gene expression by employing MMSEQ pipeline (see Methods)^41^. We found that 797 Ensembl genes (harboring 991 GASs), 311 UCSC genes (harboring 398 GASs) and 1212 AceView genes (harboring 1476 GASs) were expressed in at least one of the 16 human tissues (Fig. 2a). Furthermore, expression clustering analyses showed that many of these GAS-harboring genes were expressed across different tissues and a portion of them was expressed in a tissue-specific manner (Fig. 2b, c and d). The expression of these GAS-harboring genes suggested their potential important biological functions in human cells. Apparently, using the RefSeq annotation solely to perform functional annotation for variants may mis-classify many GASs. Our reclassification of the 1999 GASs into functional regions should allow better interpretation of GASs with their associated phenotypes.

### Reannotating RefSeq noncoding GASs as coding variants

Although the majority (95.25%) of the 1999 re-defined GASs are located in the noncoding regions by Ensembl, UCSC and AceView annotations, 95 (4.75%) are redefined here as coding SNPs (associated with 79 diseases/traits). Of these, 31 have unknown coding information (detailed allele information was unavailable from original reports), 27 are synonymous, 35 are non-synonymous, 1 is stop-gain (gain of premature stop codon) and 1 is stop-loss (loss of stop codon). Among these 95 redefined coding SNPs, 8 are re-annotated into the CDS of 8 Ensembl genes (Supplementary Table S5). However, these SNPs were previously defined as intronic variants by RefSeq annotation, since the RefSeq database did not include some exons of these genes. Similarly, 8 GASs (5 intronic SNPs and 3 intergenic SNPs by the RefSeq annotation) are re-annotated into the coding regions by the UCSC annotation (Supplementary Table S6). Additionally, 90 GASs (72 intronic SNPs and 18 intergenic SNPs by the RefSeq annotation) are re-annotated as coding SNPs by the AceView annotation (Supplementary Table S7). Among the 90 coding SNPs identified by the AceView annotation, 5 and 6 are also identified by the Ensembl (2 synonymous and 3 non-synonymous) and UCSC (1 unknown, 1 synonymous, 1 stop-gain and 3 non-synonymous) annotations, respectively, further supporting the possibility of protein-coding.

**Figure 3:**
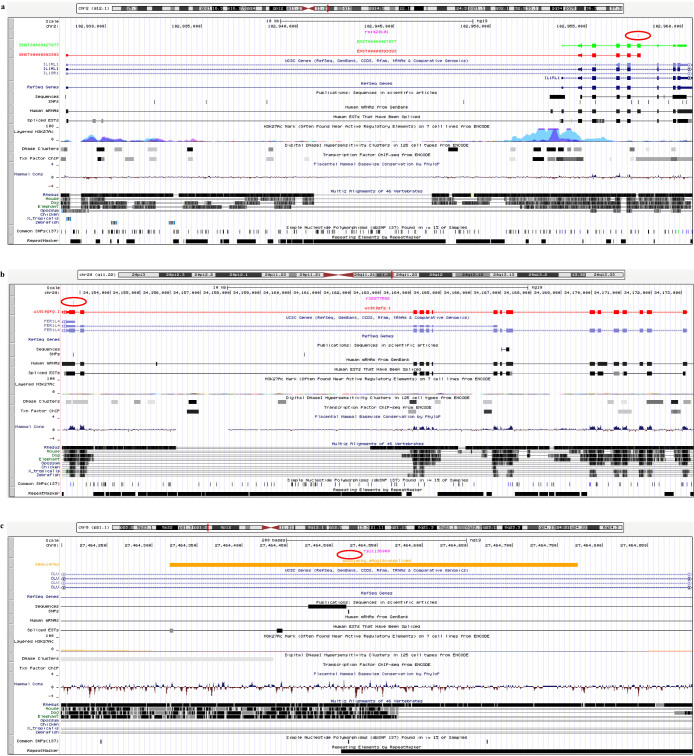
Examples of non-RefSeq-exonic GASs that were remapped to the coding regions by Ensembl, UCSC and AceView annotations. (a) An example of a non-RefSeq-exonic GAS is remapped to the Ensembl coding area. The GAS rs1420101 (in red ellipse) was in the intronic region of the *IL1RL1* gene by the RefSeq annotation; however, it can be remapped to the coding region of *ENSG00000115602* gene by the Ensembl annotation, since an exon (chr2: 102957648–102957817) of *IL1RL1* was absent from the RefSeq annotation. The variant rs1420101 introduces a non-synonymous mutation for two Ensembl transcripts, *ENST00000427077* (green) and *ENST00000393393* (red), and these two transcripts encode an identical protein. (b) An example of a non-RefSeq-exonic GAS is remapped to a coding region by the UCSC annotation. GAS rs2277862 (in red ellipse) was located in the RefSeq intergenic region. It can be remapped to the coding region of *FER1L4* gene (unavailable in the RefSeq annotation) by the UCSC annotation. The variant rs2277862 introduces a non-synonymous mutation to the transcript *uc010gfg.1* (red) of *FER1L4* gene. (c) An example of a non-RefSeq-exonic GAS is remapped to a coding region by the AceView Annotation. The RefSeq intronic GAS rs11136000 (in red ellipse) is remapped the coding region the gene *smawjarby* by the AceView annotation. The AceView gene *smawjarby* was missing by the RefSeq annotation. The Variant rs11136000 introduces a non-synonymous mutation to the transcript *smawjarby*.

Functional evaluation of proteins that harbor newly defined coding GASs using the Blast2GO software suite^42^ indicated that these proteins may contribute to the physiology/pathology of related diseases/traits (Supplementary Table S5, S6 and S7). For example, the *IL1RL1* SNP rs1420101 that was associated with eosinophil counts (*P* = 5 × 10^−14^)^43^ was considered as “intronic” by the RefSeq annotation; however, it was re-annotated as a non-synonymous coding variant for two distinct isoforms (the two transcripts encode an identical protein owing to their same CDSs but distinct UTRs) of the Ensembl gene *ENSG00000115602* (Fig. 3a). This Ensembl protein is predicted to be involved in the negative regulation of interferon-gamma production, I-kappaB kinase/NF-kappaB cascade and T-helper 1 type immunity. The SNP rs4246905 (intronic SNP by the RefSeq annotation) was associated with ulcerative colitis (*P* = 6 × 10^−12^)^44^ and inflammatory bowel disease (*P* = 3 × 10^−32^)^45^. However, our re-assessment using the Ensembl annotation indicated that this SNP would introduce a non-synonymous coding variant in the transcript *ENST00000374044*, which has functional roles in the activation of NF-kappaB-inducing kinase activity and the positive regulation of cytokine secretion. Associated with heart rate (*P* = 1 × 10^−6^)^46^, the SNP rs3117035 was an intronic variant by the RefSeq annotation, while using the UCSC annotation, this SNP is re-annotated as a premature stop codon resulting in a truncated protein derived from the *HLA-DPB2* gene. The RefSeq “intergenic” SNP rs2277862 was associated with the trait of cholesterol metabolism (*P* = 4 × 10^−10^)^47^. Re-assessment with the UCSC annotation suggested that this SNP would introduce a non-synonymous coding variant in the gene *FER1L4* whose protein product may be an essential component of the cell membrane and may play a role in protein binding (Fig. 3b). Alzheimer's disease associated SNP rs11136000 (*P* = 6–9 × 10^−10^)^48^ was identified as an intronic variant by the RefSeq annotation; however, it is re-mapped to be a non-synonymous coding variant in the gene *smawjarby* of the AceView database (Fig. 3c). Functional assessment of the protein suggested that it could be involved in the regulation of dendritic spine morphogenesis and synapse assembly.

### Validating the regulatory properties of GASs by *in vivo/vitro* experiments

**Figure 4:**
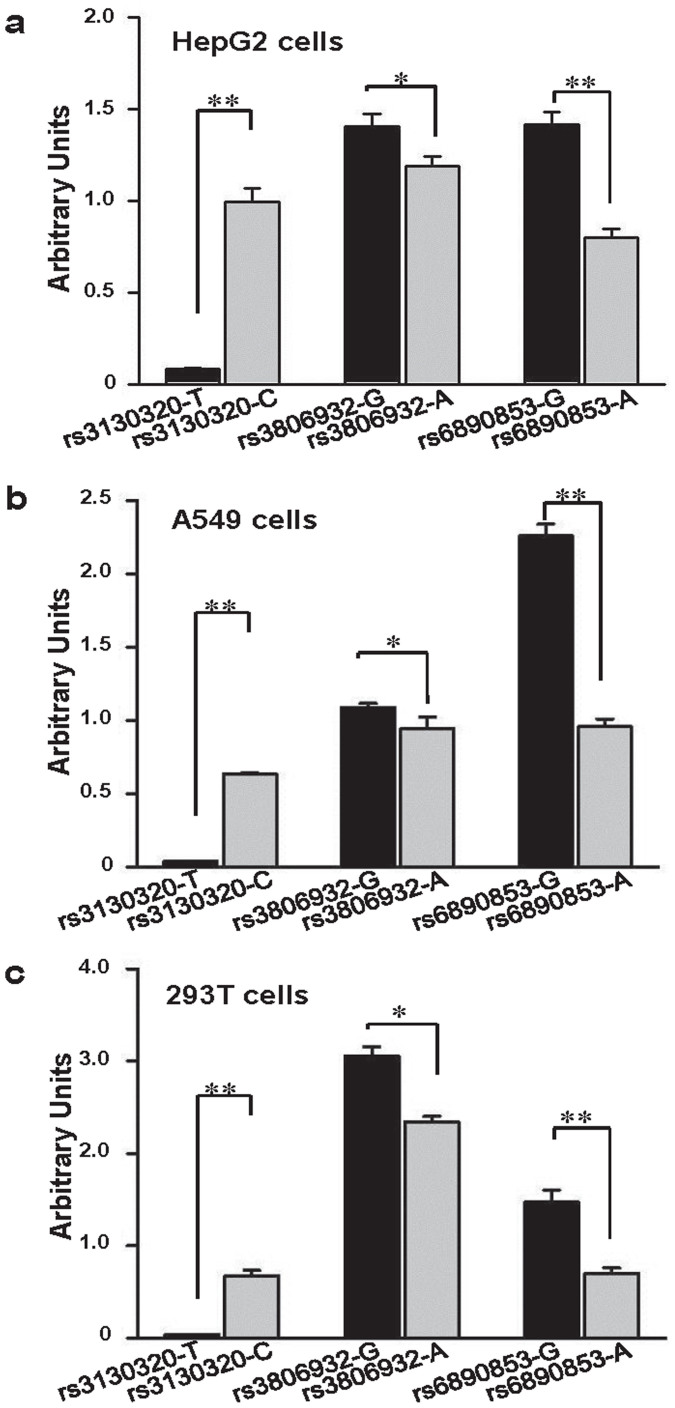
Reporter gene assays with constructs containing the potential regulator regions with different alleles. (a), (b), or (c), represents luciferase activity of different constructs with mutations at the polymorphic sites expressed in HepG2, A549 or 293T cells, respectively. All constructs were co-transfected with pRL-SV40 to standardize transfection efficiency. Luciferase levels of pGL3-PU and pRL-SV40 were determined in a triplicate manner. Fold increase was measured by defining the activity of the empty pGL3-PU vector as 1. Data shown are the means ± SD from 3 independent transfection experiments, in which each condition was performed in triplicate. * *P* < 0.05, and ** *P* < 0.001 compared with each of the construct.

To investigate the potential functions of some non-coding, non-transcribed GASs, we conducted *in vivo/vitro* experiments to explore their regulatory properties, using variants rs3130320, rs3806932, and rs6890853 as examples (see Methods). We constructed a series of pGL3-Basic reporter plasmids (plasmids with minimum promoter activity), and pGL3-PU reporter plasmids (modified plasmid with moderate promoter activity), both encompassing the core potential regulator regions that harbor the rs3130320, rs3806932 and rs6890853 polymorphic sites, respectively, and then transiently transfected these plasmids into HepG2, A549 and 293T human cell lines. We found the regulator regions failed to exhibit any promoter activity in pGL3-Basic reporter plasmids (data not shown), but indeed exhibited enhancer or silencer activity in pGL3-PU reporter plasmids. As shown in Fig. 4, the reporter gene expression driven by rs3806932-G was significantly higher than that driven by rs3806932-A in HepG2, A549 or 293T cells. Also, the rs3806932-G construct displayed a higher activity compared with pGL3-PU (the promoter activity of the pGL3-PU was defined as arbitrary unit 1) in HepG2 or 293T cells, implying a potential enhancer role of the rs3806932-G allele harboring sequence. Similar results were observed in the reporter assays for rs6890853 G allele harboring sequence, in comparison to rs6890853-A allele. As to rs3130320, the rs3130320-T construct yielded decreased activity compared to the pGL3-PU or rs3130320-C plasmid in HepG2, A549 or 293T cells, respectively, suggesting a possible negative regulation role of the rs3130320-T allele harboring sequence.

**Figure 5:**
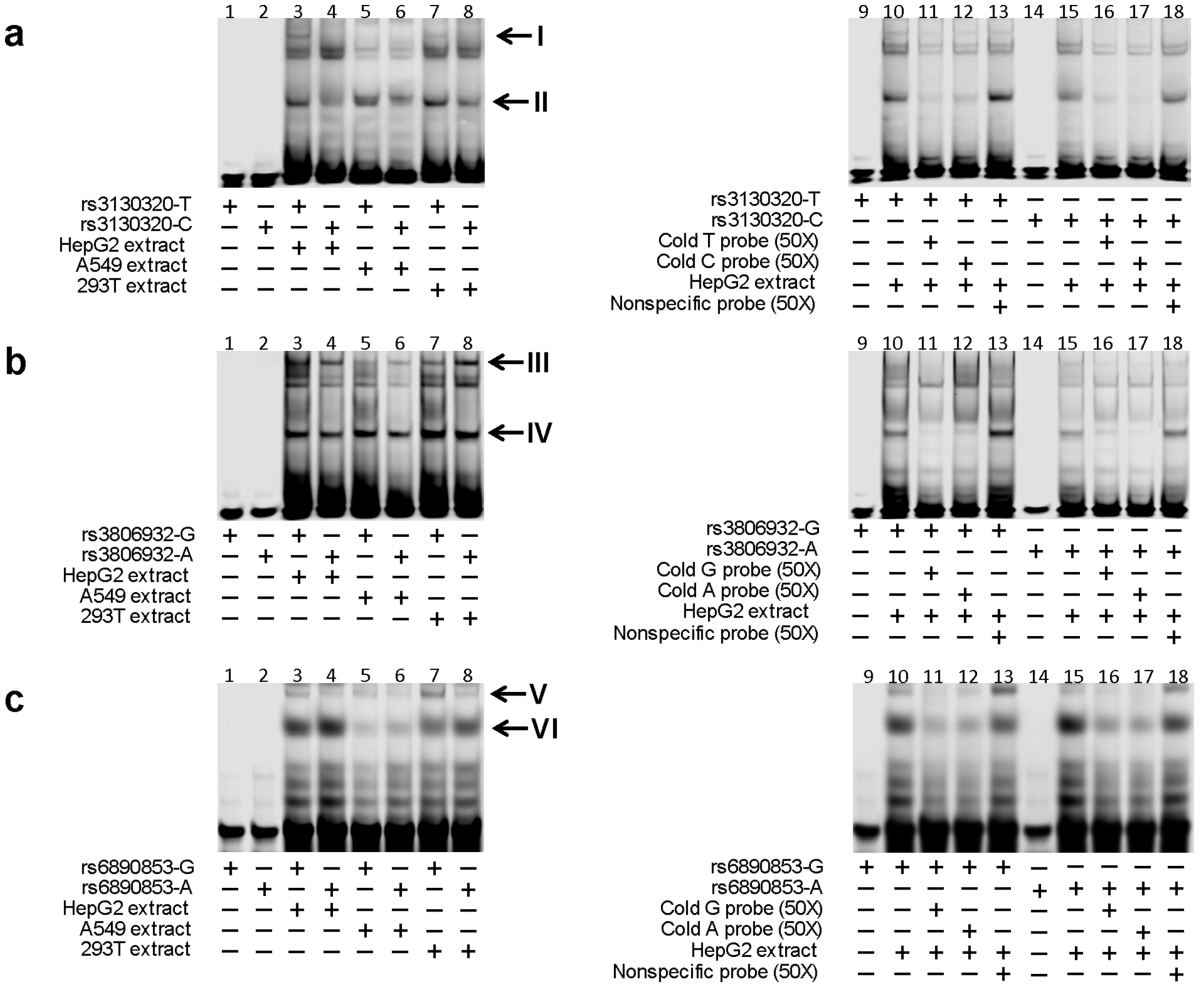
Electrophoretic mobility-shift assays (EMSA). (a) EMSA with IRD800-labeled oligonucleotides containing the rs3130320T or rs3130320C allele and nuclear extracts from HepG2, A549 or 293T cells, respectively. (b) EMSA with IRD800-labeled oligonucleotides containing the rs3806932G or rs3806932A allele and nuclear extracts from HepG2, A549 or 293T cells, respectively. Each *arrow* indicates a major oligonucleotide/nuclear protein complex. (c) EMSA with IRD800-labeled oligonucleotides containing the rs6890853G or rs6890853A allele and nuclear extracts from HepG2, A549 or 293T cells, respectively. Each *arrow* indicates a major oligonucleotide/nuclear protein complex. *Lanes 1*, *2*, *9*, and *14* showed mobility of the labeled oligonucleotides without nuclear extracts; *Lanes 3*, *4*, *10*, and *15* showed mobility of the labeled oligonucleotides with HepG2 nuclear extracts in the absence of competitors; *Lanes 5*, and *6* showed mobility of the labeled oligonucleotides with A549 nuclear extracts in the absence of competitors; *Lanes 7*, and *8* showed mobility of the labeled oligonucleotides with 293T nuclear extracts in the absence of competitors; *Lanes 11* and *16, 12* and *17*, and *13* and *18* showed mobility of the labeled oligonucleotides with nuclear extracts in the presence of unlabeled wild allele, mutant allele, and non-specific competitor, respectively. Each *arrow* indicates an oligonucleotide/nuclear protein complex.

Electrophoresis mobility shift assays (EMSAs) were then performed to examine whether the rs3130320, rs3806932, and rs6890853 polymorphic sequences exhibited the ability to bind nuclear proteins. Under our experimental conditions, the nuclear extracts from HepG2, A549 or 293T cells were able to specifically bind both rs3130320-T and rs3130320-C probes (Fig. 5a, *Band* *I* and *Band* *II*; Fig. 5a, *Lanes* *11–13* and *Lanes* *16–18*), but with significant differences in binding affinity binding efficacy (Fig. 5a; *Lane* *10* versus *Lane* *15*). Two sequence-specific DNA-protein binding bands were also observed in rs3806932 EMSAs (Fig. 5b, *Band III* and *Band* *IV*), in which rs3806932-G probe showed a higher protein-binding affinity in *Band IV* but a decreased protein-binding affinity in *Band III*. Similar results were observed in rs6890853 EMSAs (Fig. 5c, *Band V* and *Band VI*). These observations suggest the potential regulatory roles of these SNPs in altering transcriptional activity.

In the original GWAS studies, rs3130320, rs3806932 and rs6890853 were found to be associated with systemic lupus erythematosus^49^, eosinophilic esophagitis^50^ and primary biliary cirrhosis^51^, respectively. Our experimental validation showed that rs6890853 has a regulatory role in HepG2 cells, suggesting the relevance of its expression in liver, with a liver disease-primary biliary cirrhosis. Furthermore, except rs3130320 was annotated as known eQTL in RegulomeDB^27^, rs3806932 and rs6890853 were not characterized as eQTLs previously.

## Discussion

In the current study, we used diverse resources coupled with integrative approaches to reassess the GASs annotated previously as noncoding by RefSeq in a systematic way. Approximately 96% of these RefSeq noncoding GASs were mapped to genomic regions with various functional features, suggesting that these GASs may have potential biological significances. Diverse regulatory elements are interspersed within the human genome to provide specific binding/interaction sites for corresponding proteins. DNA-protein interactions are crucial for regulating chromatin structures and gene expression. Moreover, GASs in regulatory elements may affect DNA-protein interactions by disturbing transcription factor binding sites or by changing the alleic chromatin states^25^ in ways that impact relevant phenotypic traits.

Previously, GASs found in most GWASs were annotated or functionally evaluated mainly by using the RefSeq gene annotation database^1^; however, the incomplete RefSeq annotation database mapped many GASs into intergenic regions that actually could be located in genetic regions when annotated using other databases. By reannotating the RefSeq noncoding GASs to functional gene regions using different annotation databases, such as Ensembl, UCSC and AceView, 1999 GASs were reassigned to have potential biological functions (promoters, intragenic regions or coding sequences). The fact that gene annotations among different databases are distinct, but complementary, provides the basis for such a large number of RefSeq noncoding GASs to be re-associated with human genes. Each database, having its specific gene annotation pipeline with unique properties, differs from other databases in human gene annotations, which could lead to certain disparities in gene annotation qualities and quantities^24^. Therefore, the integrative application of the gene annotation using diverse databases could obtain a more comprehensive gene set, and thus avoid the mis-annotation of a portion of GASs that results from using RefSeq gene annotation alone.

Expression profile analysis using RNA-Seq data shows that the majority of the genes harboring relocated GASs are widely expressed in different human tissues, demonstrating that these GAS-harboring genes are transcribed and are likely to possess functional roles. The reassessment of GASs into promoters, UTRs or CDSs could lead us to reconsider their potential functions that affect gene expression or protein structure/function. The ENCODE project indicated that ~75% of the human genome could be transcribed, but it was not estimated how many genes (including both protein-coding and noncoding) are expected to be undiscovered^52^. It is easy to estimate the percentage of transcribed regions accounting for the human genome; however, it is still difficult to determine how many genes are undiscovered/unannotated due to the limitations of sequencing technologies and bioinformatics algorithms as well as the incompleteness of human genome^22^,^53^. Many novel genes/transcripts reconstructed by Cufflinks^54^ or other transcriptome reconstruction tools based on RNA-Seq data were gene fragments other than full-length genes with complete structure^53^,^55^. With the fast evolution of both sequencing technology and bioinformatics methodology, the human gene annotation will be more comprehensive and sophisticated. It is worth noting that those unannotated genes could have important functions, and cannot be simply treated as transcription noise. For example, we have learnt a lesson that the important transcript biotype of long noncoding RNAs were considered as non-functional previously^56^,^57^. The biological functions of those unannotated genes/transcripts could be characterized in the future with the advancement of technology and evolution of our knowledge towards biology.

In addition, many GASs were located in non-transcribed regions, and whether these variants act as functional polymorphisms or if they only serve as genetic markers is not clear yet. In this study, we selected 3 GASs in regulatory or intergenic regions to validate their functional significance using a series of biochemical assays. Interestingly, we observed the core regions harboring the polymorphic sites failed to exhibit any promoter activity, but indeed exhibited enhancer or silencer activity. Furthermore, significant differences in enhancer or silencer activity were observed between the different allelic sequences, most likely due to different binding activities toward unknown transcriptional factors. All these results support the notion that the GASs in non-transcribed regions might affect gene transcription by long-range regulation.

In summary, our integrative analytical strategy using diverse databases, annotation tools and experimental approaches have dramatically impacted SNP functional assessment, by which the majority of noncoding GASs in the RefSeq database were re-annotated into diverse functional categories, including intragenic and promoter areas, and other possible regulatory regions. The re-classification of these GASs provided an important example for further unraveling the relationships between many noncoding GASs and phenotypes, which may help to explain and predict the susceptibility of diverse diseases. Moreover, our integrative approaches could be applied to characterize the genetic variants identified by genome/exome sequencing data as well.

## Methods

### Original GASs and annotation resources used in this study

We archived all published GASs from the National Human Genome Research Institute GWAS Catalog^1^ and removed those SNPs that do not have clear rs numbers, therefore, 9828 GASs were obtained. We downloaded the Ensembl human gene annotation file (GTF format)^9^ (version 71, corresponding to GENCODE 16^10^) from the Ensembl database, and the UCSC^11^ and AceView^12^ gene annotations from the UCSC Table Browser and AceView database, respectively. To annotate the GASs with these three databases, we also downloaded the related human protein sequences and mRNA sequences from the Ensembl, UCSC and AceView databases. To investigate the conservation of the GASs, we obtained the human conserved DNA elements across mammals predicted by phastCons^36^ from the UCSC database as well. The UCSC tfbsConsSites track was also downloaded for predicting the transcription factor binding sites of GASs.

### Mapping the GASs to regulatory features

To reassess noncoding GASs, we eliminated the SNPs that were mapped to the exonic and splicing regions of the RefSeq database from the whole set of 9828 GASs. The sequences of risk alleles for many GASs are the same as the reference sequences of the human genome, which can be explained by several reasons: (i) the opposite alleles of GASs were reported as risk in the database owing to their relative risk (RR) or odds ratio (OR) < 1^1^; (ii) some reference alleles may become detrimental in evolution due to environmental pressures; and (iii) a fraction of reference alleles could be different among distinct populations because of polymorphisms. We then explored non-RefSeq-exonic GASs with the annotation of regulatory features catalogued in the HaploReg^26^ and RegulomeDB^27^ databases. Annotated regulatory features included promoter, enhancer, regulatory motifs, DNAse footprinting sites, expression quantitative trait loci (eQTL), and conserved elements (predicted by GERP^33^ and SiPhy^34^,^35^). We separately conducted the mapping analyses for GASs using the web servers of HaploReg and RegulomeDB.

### Annotating the GASs for gene features using different databases

We then further annotated those noncoding GASs with the human gene annotation databases Ensembl, UCSC and AceView, using ANNOVAR^37^. The noncoding GASs were mapped to various regions of genes in these three databases. Specifically, remapped GASs fell into one of the following categories: (i) remapped to the CDSs (coding sequences) and splicing regions (2 bp away from an exon/intron boundary) of the Ensembl, UCSC or AceView databases; (ii) remapped to the areas of 5′ UTRs (untranslated regions) and 3′ UTRs of genes in the Ensembl, UCSC or AceView databses; (iii) remapped to the intronic regions of the genes in the Ensembl, UCSC or AceView databases, but they fell in the RefSeq intergenic areas by the RefSeq annotaion; (iv) remapped to the ncRNA (noncoding RNA) exonic regions of genes in the Ensembl, UCSC or AceView databases; and (v) relocated to the promoter regions (1 kb upstream of transcription start site) of genes in the Ensembl, UCSC or AceView databases.

### Expression quantification, protein identification and functional annotation of GAS related genes

We used the RNA-Seq data of 16 distinct tissues from the Illumina Human Body Map (HBM) 2.0 project (ArrayExpress ID: E-MTAB-513) to investigate the expression profile of GASs related genes that were re-annotated by three databases. Quantification of the expression of genes and their isoform was conducted with the MMSEQ pipeline. We first separately aligned each RNA-Seq dataset to the Ensembl, UCSC and AceView transcriptome using Bowtie^40^ (version 1.0.0) with the parameters of “-a --best --strata -S -m 100 -X 500 --chunkmbs 256”. Then, we utilized MMSEQ^41^ (version 1.0.2) to estimate the expression of genes and isoforms in each tissue. To distinguish the genes with low expression from noises, we considered that genes with a posterior standard deviation of expression lower than 1.5 were expressed.

In order to interpret the functional consequences of re-annotated noncoding GASs by the Ensembl, UCSC and AceView annotations, we carried out the functional assessment for GAS related proteins using Blast2GO^42^ suite with default parameters.

### Luciferase reporter gene assays

To experimentally validate the potential regulatory roles of those GASs, we used the following criteria to selected GASs for *in vivo/vitro* experiment: i) the SNPs are within the intergenic regions of RefSeq genes; ii) the SNPs are located in the promoter region (1 kb upstream of transcription start site) of the genes, according to at least two annotations of Ensembl, UCSC and AceView databases; iii) the genes with SNPs of interest in the promoter regions are expressed in the 16 different human tissues of Illumina Human Body Map (HBM) 2.0 project; iv) and then we randomly selected rs3130320, rs3806932 and rs6890853 as the target polymorphisms to experimentally validate their regulatory roles.

To facilitate function experiments, the pGL3-promoter (with moderate promoter activity) vector (Promega, Madison, WI) was modified according to the Universal USER Cassette (New England Biolabs, Beverly, MA) protocol with some modifications. Briefly, we removed the Xba I site by site-specific mutation and inserted double strand oligonucleotides (sense 5′-GCT GAG GGA AAG TCT AGA GGA TCC TCT AGA TGT CTC CTC AGC-3′, antisense 5′-GCT GAG GAG ACA TCT AGA GGA TCC TCT AGA CTT TCC CTC AGC-3′) in the Sma I cloning site of the pGL3-promoter vector, generating pGL3-PU vector. The pGL3-PU vector was digested with Xba I and Nt.BbvC I (New England Biolabs) to generate linearized nicked vector for subsequent plasmid construction. Cloning primers were designed to amplify target sequences with extension oligonucleotides 5′-GGA GAC AU-3′ or 5′-GGG AAA GU-3′ in their 5′ end. PCR products were digested with USER enzyme (New England Biolabs) and cloned into the linearized nicked pGL3-PU vector prepared as described above.

Three DNA fragments corresponding to the regulator regions harboring the rs3130320, rs3806932 and rs6890853 polymorphic sites were generated by PCR (primers are available upon request) and subcloned into pGL3-PU, respectively. The resultant plasmids, designated rs3130320-T, rs3806932-G or rs6890853-G, respectively, were sequenced to confirm that they contained exclusively rs3130320 T, rs3806932 G or rs6890853 G allele. The rs3130320-C, or rs3806932-A or rs6890853-A plasmid construct, that contains rs3130320 C, rs3806932 A or rs6890853 A allele, respectively, was then created by site-specifical mutations based on the rs3130320-T, rs3806932-G or rs6890853-G plasmid, respectively. All constructs used in this study were restriction mapped and sequenced to confirm their authenticity.

Cell lines HepG2, A549 and 293T were used for luciferase assays. The constructed reporter plasmid or the blank pGL3-promoter plasmid was cotransfected with pRL-SV40 (Promega) to the cells, respectively, using Lipofectamine reagent (Life Technologies, Carlsbad, CA). The pRL-SV40, containing Renillareniformis luciferase, was used to standardize transfection efficiency. For each plasmid, at least three independent transfection experiments were carried out, and each was performed in a triplicate manner. A *t*-test was used to examine the differences in luciferase reporter gene expression with different genotypes. *P* < 0.05 was used as the criterion of statistical significance.

### Electrophoretic Mobility Shift Assays

Nuclear extracts were prepared from HepG2, A549, or 293T cells, using NE-PER extraction reagents (Pierce, Rockford, IL). For electrophoretic mobility shift assays (EMSA), synthetic double-stranded and 5′IRD800-labeled oligonucleotides (22–24 bases) corresponding to the rs3130320 T, rs3130320 C, rs3806932 G, rs3806932 A, rs6890853 G or rs6890853 A allele sequences and HepG2, A549, or 293T cell nuclear extract were incubated at 25^o^C for 20 min using the Light Shift Chemiluminescent EMSA Kit (Pierce, Rockford, IL), respectively. The reaction mixture was separated on 6% PAGE by electrophoresis, and the resultant mobility shifts were detected by Odyssey CLx Infrared Imaging System (LI-COR Biosciences, Lincoln, NE). Unlabeled oligonucleotides at 50-fold molar excess were added to the reaction for competition assays.

## Author Contributions

G.C., B.N. and T.S. conceived and designed the study, G.C., D.Y., J.C., R.C., J.Y., H.W. and X.J. performed analyses. D.Y. carried out the experiments. G.C., D.Y., B.N. and T.S. wrote the manuscript. All the authors read and approved the final manuscript.

## Supplementary Material

Supplementary Information Supplementary information
