# Supplementary material for: Re-annotation of presumed noncoding disease/trait-associated genetic variants by integrative analyses
Source: Sci Rep. 2015 Mar 30;5:9453. doi: 10.1038/srep09453 (PMC4377585; doi:10.1038/srep09453)
Supplement: Supplementary Information [file srep09453-s1.pdf]

Supplementary information

Re-annotation of presumed noncoding disease/trait-associated genetic variants by integrative analyses

Geng Chen[1,3#], Dianke Yu[2#], Jiwei Chen[1], Ruifang Cao[1], Juan Yang[1], Huan Wang[1], Xiangjun Ji[1], Baitang Ning[2*], Tieliu Shi[1*]

[1]The Center for Bioinformatics and Computational Biology, Shanghai Key Laboratory of Regulatory Biology, the Institute of Biomedical Sciences and School of Life Sciences, East China Normal University, Shanghai 200241, China, [2]National Center for Toxicological Research, US Food and Drug Administration, Jefferson, AR 72079, USA, [3]Center for Pharmacogenomics, School of Pharmacy, Fudan University, Shanghai 201203, China.

# These authors contributed equally to this work.

*** Corresponding Authors:**

Tieliu Shi

Center for Bioinformatics and Computational Biology

East China Normal University

Shanghai 200241, China

Tel: 86-21-54345020

Fax: 86-21-54344922

E-mail: tieliushi01@gmail.com

Baitang Ning

National Center for Toxicological Research

US Food and Drug Administration

Jefferson, AR 72079, USA

Email: Baitang.Ning@fda.hhs.gov

Table S1. Regulome DB score for re-annotated GASs

Chromosome	Coordinate	dbSNP ID	Regulome DB score
chr9	36997416	rs3758171	2b
chr9	37002114	rs7020413	5
chr9	137269455	rs34312136	5
chr9	137280938	rs35079168	5
chr9	137284922	rs4501664	2b
chr9	137285502	rs11102986	5
chr9	27543280	rs3849942	6
chr8	120353266	rs2469997	6
chr8	135566566	rs7827545	5
chr8	135567045	rs1372662	4
chr8	27456252	rs2279590	5
chr8	27464518	rs11136000	6
chr8	27468861	rs9331888	2b
chr6	32433166	rs2395185	6
chr6	32560858	rs28366298	6
chr6	32109978	rs204999	6
chr6	32383107	rs9268528	1f
chr6	32384720	rs9268542	6
chr6	32428284	rs6903608	6
chr6	32572250	rs2858870	1f
chr6	32321553	rs2395148	6
chr6	32389647	rs3135363	6
chr6	32664959	rs9357152	1f
chr6	33624732	rs999943	5
chr6	160907133	rs3127599	6
chr6	160969737	rs10755578	5
chr5	36423930	rs7735940	2b
chr4	131131415	rs11099040	6
chr4	5367984	rs7697839	6
chr4	5368223	rs7673097	2b
chr3	2886526	rs17584516	5
chr3	2893273	rs4629318	5
chr3	2895683	rs11713158	5
chr22	32024979	rs11703808	5
chr22	32025912	rs761746	5
chr22	32027448	rs12627933	1f
chr22	32049916	rs9621305	5
chr20	16455773	rs932541	5
chr20	16459308	rs6044001	6
chr20	16460714	rs6044003	5
chr20	33545615	rs17310467	2b
chr20	33753261	rs6060278	5
chr20	15599530	rs200752	6
chr20	15606420	rs200759	6

chr20	48554976	rs2235617	4
chr20	32738611	rs1015362	4
chr2	192010487	rs7574070	6
chr2	192015071	rs7572482	1b
chr2	192017770	rs897200	1d
chr2	201571142	rs2540051	4
chr2	83293379	rs10496289	6
chr2	60713234	rs10189857	5
chr2	60718042	rs1427407	5
chr2	60718346	rs7599488	5
chr2	60719969	rs766432	5
chr2	60720950	rs4671393	5
chr2	60721310	rs7584113	5
chr2	60721346	rs7557939	5
chr2	60722039	rs6706648	5
chr2	60722240	rs6738440	5
chr2	60723265	rs7565301	6
chr2	60723671	rs6729815	5
chr2	60724085	rs1896295	5
chr2	60724086	rs1896296	5
chr2	60725450	rs7606173	4
chr2	34284851	rs9308945	6
chr2	34305256	rs10495809	6
chr19	48522868	rs3815908	6
chr19	48531215	rs2560966	6
chr12	45925754	rs7960483	6
chr12	45930972	rs10785581	5
chr12	104317995	rs1165668	3a
chr12	104318171	rs1165669	3a
chr12	69727189	rs317689	5
chr12	69824023	rs7297610	6
chr11	24740433	rs2716458	6
chr11	24741658	rs12798374	6
chr11	14915309	rs2060793	5
chr11	71234106	rs11234027	6
chr10	13991864	rs7081208	4
chr10	14008444	rs2446581	6
chr10	14016158	rs17314229	4
chr10	5353689	rs9423406	6
chr10	5354676	rs7896729	5
chr1	61820071	rs17121983	5
chr1	61821818	rs7556462	4
chr1	34180841	rs16835742	5
chr1	34186192	rs528059	6
chr1	34189428	rs544991	6
chr1	145395603	rs12091564	1f
chr1	145416055	rs10218795	6

chr1	207692048	rs6656401	5
chr1	207784967	rs3818361	6
chr1	67590460	rs11209002	6
chr1	67601131	rs11209003	4
chr1	67670915	rs2902440	6
chr1	67685597	rs11465802	5
chr1	67702525	rs11465804	6
chr1	67722566	rs10889676	6
chr1	67726103	rs9988642	5
chr1	67730054	rs6669582	5
chr1	67730627	rs10789230	5
chr14	69034681	rs999737	6
chr5	150473673	rs999556	4
chr4	89220943	rs9995093	5
chr4	77360430	rs9992101	5
chr3	46339811	rs9990343	4
chr3	11040438	rs9990174	4
chr16	56985138	rs9989419	1f
chr6	43757895	rs998584	4
chr21	37809376	rs9984974	3a
chr21	36416331	rs9983044	5
chr21	41415043	rs9981861	6
chr18	42725661	rs998124	6
chr21	44156768	rs9979235	6
chr21	35652238	rs9978142	5
chr21	43836389	rs9976767	5
chr21	27716653	rs9975851	5
chr2	53502774	rs996712	5
chr18	12606462	rs9959145	4
chr18	73536710	rs9956878	5
chr18	33097959	rs9953852	5
chr18	71878516	rs9951925	5
chr18	76657778	rs9951602	6
chr12	109840939	rs9943753	6
chr4	82165789	rs994014	6
chr16	53800753	rs9940128	4
chr1	218860067	rs993925	5
chr16	57002731	rs9939224	5
chr16	88331639	rs9938149	5
chr16	16730727	rs9937036	6
chr16	86403117	rs9936833	5
chr16	73439354	rs9934948	6
chr16	60314655	rs9932186	6
chr16	68820945	rs9929218	4
chr16	89818088	rs9926296	6
chr11	131336073	rs992564	5
chr16	7504853	rs9924951	5

chr16	11210414	rs9923856	5
chr16	31107688	rs9923231	5
chr16	18024849	rs9922516	5
chr16	54494423	rs9921518	5
chr16	375781	rs9921222	1f
chr6	162001435	rs992037	6
chr7	93691743	rs9918668	5
chr4	15545409	rs9918079	5
chr2	169143034	rs9917256	6
chr17	70098160	rs9913711	6
chr4	100322444	rs991316	6
chr17	75701696	rs9906155	5
chr17	12637370	rs9905820	6
chr13	33387245	rs990324	6
chr17	32464153	rs9901756	6
chr17	76868613	rs9900972	5
chr17	13062794	rs9900808	6
chr17	59456588	rs9895661	4
chr17	42064986	rs9895585	2b
chr10	127135837	rs989507	6
chr17	40507979	rs9891119	5
chr16	31313252	rs9888739	5
chr3	169080184	rs9883650	5
chr3	123096819	rs9883204	6
chr6	33042879	rs987870	1f
chr3	48487337	rs9876781	6
chr3	13961156	rs9875589	5
chr8	129946153	rs987525	4
chr4	138227608	rs987360	6
chr6	50803049	rs987237	6
chr3	126993098	rs9871760	5
chr3	7529554	rs9870680	5
chr16	86322458	rs987052	4
chr3	72437412	rs9863706	4
chr3	1345424	rs9861887	6
chr3	195800546	rs9859260	4
chr3	141078187	rs9857275	5
chr3	188087627	rs9851967	5
chr3	38719934	rs9851724	5
chr3	53062660	rs9847710	6
chr3	138025395	rs9846480	6
chr3	13743131	rs9846232	5
chr3	32842100	rs9845475	4
chr3	135974215	rs9844666	6
chr1	119503842	rs984222	4
chr3	114362763	rs9841504	6
chr3	108992	rs9841287	6

chr3	133589323	rs9835973	5
chr3	36856029	rs9834970	4
chr1	88132379	rs983332	6
chr3	142649109	rs9832727	5
chr3	78353590	rs9831754	6
chr3	49719728	rs9822268	5
chr5	45285717	rs981782	6
chr3	41912650	rs9815354	6
chr3	95688842	rs9814870	6
chr3	128652552	rs9810890	5
chr3	142982898	rs9810857	5
chr3	105010889	rs9810233	6
chr13	24658355	rs9805786	5
chr10	130248925	rs9804317	6
chr1	167156499	rs9803659	6
chr8	4282617	rs980238	6
chr5	150594801	rs979455	4
chr8	129264588	rs9792269	6
chr2	214838512	rs9789347	6
chr15	74548772	rs9783698	6
chr3	67373976	rs977102	6
chr3	191897217	rs975121	6
chr10	128777389	rs9733352	6
chr1	85290408	rs9728717	5
chr6	127391843	rs972275	5
chr13	88762345	rs969962	6
chr8	7189357	rs9692809	6
chr5	26389261	rs969088	4
chr5	55861785	rs9686661	4
chr22	39670850	rs968451	1f
chr17	47084710	rs9674544	4
chr20	6620892	rs967417	6
chr2	218310339	rs966423	6
chr10	89338632	rs9664222	3a
chr10	95895176	rs9663362	5
chr6	35363756	rs9658108	5
chr3	105586713	rs9657904	5
chr8	138905295	rs9657451	6
chr9	100556108	rs965513	6
chr20	3381548	rs965469	5
chr2	100825366	rs9653442	6
chr15	77963886	rs9652490	4
chr8	57155597	rs9650315	6
chr16	87461968	rs9646303	6
chr13	38065445	rs9646096	6
chr11	116648916	rs964184	1f
chr11	30749089	rs963837	6

chr2	88166460	rs9636470	6
chr18	21749614	rs9635963	5
chr17	49613784	rs9635759	6
chr11	9111557	rs963167	2b
chr11	13620171	rs9630182	6
chr2	6697087	rs962528	5
chr22	40452118	rs9623117	5
chr22	33084510	rs9621532	5
chr9	22362103	rs961831	5
chr20	6404280	rs961253	5
chr22	39955872	rs9611198	4
chr22	32867527	rs9609565	6
chr22	23644793	rs9608102	5
chr22	37919266	rs9607469	6
chr1	36571919	rs96067	5
chr13	114622596	rs9604529	5
chr2	223937558	rs960246	5
chr8	59708308	rs960089	5
chr13	73728138	rs9600079	6
chr4	2395296	rs959770	4
chr8	100835181	rs959695	6
chr13	50842439	rs9596270	5
chr5	126181861	rs959573	5
chr13	42952144	rs9594738	4
chr13	39370594	rs9594293	6
chr13	71883213	rs9592783	6
chr5	165063883	rs958994	5
chr1	110770222	rs958798	5
chr13	99385989	rs9582259	5
chr13	25082629	rs9581094	3a
chr13	108257219	rs957788	5
chr13	80668873	rs9574565	6
chr13	78808913	rs9574199	6
chr13	73908845	rs9573163	1f
chr13	44637404	rs9567349	6
chr8	122909686	rs956225	5
chr13	94341095	rs9561428	6
chr13	99956621	rs9557195	4
chr13	98016415	rs9556711	5
chr13	23864656	rs9552911	6
chr13	19311240	rs9552416	6
chr13	40505509	rs9548988	5
chr10	133957760	rs954820	5
chr13	38531580	rs9548119	6
chr13	38363449	rs9548050	5
chr13	85054265	rs9546711	6
chr13	73916627	rs9543325	6

chr13	29368705	rs954108	5
chr13	43770473	rs9533425	2b
chr13	43128576	rs9525638	5
chr13	93331885	rs9523762	6
chr13	99126302	rs9517320	2b
chr13	99096033	rs9517302	5
chr13	100196951	rs9513627	6
chr13	28429737	rs9512900	5
chr13	28077143	rs9512730	6
chr13	27920610	rs9512637	6
chr13	24205194	rs9510787	5
chr15	78926017	rs950776	1f
chr6	32961360	rs9500927	6
chr4	126431918	rs950063	5
chr15	45801034	rs950027	6
chr6	134576510	rs9493873	6
chr6	127456121	rs9491697	6
chr6	127452638	rs9491696	2b
chr6	98919263	rs9491640	6
chr6	124691236	rs9491140	6
chr6	116312892	rs9488822	6
chr6	114591985	rs9488363	6
chr6	114017132	rs9488238	6
chr6	109742014	rs9487094	5
chr6	135435500	rs9483788	2a
chr6	108916572	rs9480865	6
chr6	150358011	rs9479482	6
chr6	157441049	rs9478751	4
chr6	136133658	rs947583	4
chr10	6390449	rs947474	4
chr6	44946505	rs9472414	6
chr6	43811761	rs9472138	5
chr6	41303804	rs9471576	5
chr6	38106843	rs9470794	6
chr6	36623378	rs9470361	4
chr6	35341849	rs9470004	5
chr6	33706478	rs9469578	5
chr6	33489881	rs9469457	5
chr6	32658309	rs9469220	1f
chr6	31258836	rs9468925	6
chr6	24441745	rs9467160	4
chr6	21384612	rs9466056	2b
chr6	20717254	rs9465871	4
chr6	31271715	rs9461688	6
chr6	21455152	rs9460635	5
chr9	117049890	rs946053	3a
chr6	20567540	rs9460521	5

chr6	158389740	rs9458975	6
chr9	22115285	rs944797	5
chr20	60243410	rs944260	4
chr1	16393356	rs9442235	5
chr14	103572814	rs944002	5
chr1	95549535	rs9437689	5
chr6	43795967	rs943072	4
chr1	11046854	rs9430161	4
chr1	118855586	rs9428104	5
chr1	192995212	rs9427573	6
chr1	153769399	rs9426935	5
chr10	105675945	rs9419958	5
chr10	81139461	rs941873	4
chr13	41013976	rs941823	6
chr14	101306044	rs941576	5
chr12	57023283	rs941207	2b
chr12	54114157	rs941184	6
chr9	118202715	rs9409154	6
chr9	123951916	rs9408928	5
chr6	143994198	rs9403521	5
chr6	135427816	rs9402686	6
chr6	134720066	rs9402592	5
chr6	110007646	rs9400317	6
chr6	135419017	rs9399137	6
chr6	122146033	rs9398652	6
chr6	154986663	rs9397738	6
chr6	45095162	rs9395066	6
chr6	33618161	rs9394159	5
chr6	11042908	rs9393903	4
chr6	23077310	rs9393366	5
chr4	69167670	rs939207	5
chr6	148423370	rs9390537	5
chr6	143943313	rs9390123	2b
chr6	126698718	rs9388489	6
chr6	106094056	rs9386463	6
chr6	152295612	rs9383951	4
chr6	151987356	rs9383938	5
chr6	16343055	rs9383153	5
chr6	35502201	rs9380516	5
chr6	417726	rs9378805	5
chr6	3294823	rs9378357	6
chr6	31327700	rs9378249	5
chr6	135427143	rs9376092	6
chr6	133867801	rs9375969	6
chr6	109616419	rs9374080	1b
chr6	147701132	rs9373523	6
chr15	57910163	rs937254	6

chr6	150913960	rs9372078	4
chr6	152790572	rs9371601	6
chr6	31802540	rs9368699	4
chr6	30938882	rs9368649	5
chr6	55148102	rs9367630	5
chr6	158435571	rs9365723	5
chr6	166128637	rs9364813	5
chr6	160833663	rs9364554	4
chr6	76265641	rs9360921	5
chr6	20812587	rs9358372	5
chr6	32809847	rs9357155	6
chr6	167383074	rs9355610	5
chr6	69294484	rs9351730	6
chr6	47453377	rs9349407	4
chr6	12903956	rs9349379	6
chr6	41925158	rs9349205	4
chr6	41914377	rs9349204	6
chr6	31575275	rs9348876	5
chr5	90762747	rs933688	5
chr1	47404185	rs9332998	6
chr21	31100796	rs933117	5
chr10	95895939	rs932764	6
chr6	25641199	rs932316	6
chr6	138266684	rs9321637	6
chr6	135494874	rs9321490	3a
chr6	122114450	rs9320841	5
chr8	101919320	rs931812	4
chr13	24432466	rs9318086	5
chr13	63634349	rs9317284	5
chr13	51094113	rs9316500	4
chr19	22614121	rs931608	6
chr13	39516626	rs9315632	6
chr5	35803576	rs931555	5
chr13	36663301	rs9315385	6
chr13	33693836	rs9315204	5
chr13	30458736	rs9314986	5
chr4	55241580	rs9312648	6
chr4	168139379	rs9312517	4
chr2	73743981	rs9309473	6
chr15	101558561	rs930847	5
chr1	9432389	rs9308447	5
chr21	31386242	rs9305406	6
chr21	29475195	rs9305354	6
chr18	39937508	rs9304270	5
chr2	42981238	rs930421	5
chr17	44187256	rs9303525	1b
chr17	43805193	rs9303521	6

chr17	56847944	rs9303401	6
chr17	37976468	rs9303277	6
chr17	80408814	rs9303029	2a
chr16	50719102	rs9302752	2b
chr13	94952831	rs9301951	5
chr11	41915365	rs9300039	6
chr8	55437523	rs9298506	5
chr7	98759116	rs9297145	6
chr6	14597981	rs9296949	6
chr6	53924696	rs9296736	5
chr6	40440499	rs9296335	6
chr6	39361853	rs9296295	6
chr6	38365840	rs9296249	6
chr6	32218988	rs9296015	6
chr6	22131928	rs9295536	6
chr6	20652716	rs9295474	2c
chr5	40437947	rs9292777	4
chr3	190252634	rs9290936	5
chr3	188442479	rs9290877	6
chr3	178429938	rs9290663	6
chr2	217481270	rs9288520	4
chr2	176717740	rs9287989	6
chr3	87152168	rs9284813	6
chr6	32736694	rs9276606	6
chr6	32687972	rs9275698	6
chr6	32681630	rs9275596	6
chr6	32678998	rs9275572	6
chr6	32669155	rs9275390	6
chr6	32666294	rs9275319	6
chr6	32625868	rs9273349	1f
chr6	32606755	rs9272535	1f
chr6	32604371	rs9272346	6
chr6	32602268	rs9272219	6
chr6	32599998	rs9272105	6
chr6	32595222	rs9271858	4
chr6	32586853	rs9271366	6
chr6	32576477	rs9271100	1f
chr6	32573990	rs9270984	6
chr6	32432834	rs9268923	6
chr6	32431146	rs9268877	6
chr6	32429642	rs9268853	6
chr6	32408526	rs9268645	1f
chr6	32379488	rs9268516	6
chr6	162161618	rs926849	5
chr6	32341352	rs9268402	6
chr6	32319636	rs9268301	6
chr6	32205109	rs9267911	6

chr6	31883678	rs9267673	1f
chr6	31870855	rs9267665	5
chr6	31867252	rs9267663	2b
chr6	31336417	rs9266406	4
chr6	31274379	rs9264942	6
chr6	31111355	rs9263739	1f
chr6	31024807	rs9262632	6
chr11	27667201	rs925946	5
chr6	29723160	rs9258260	1f
chr6	29356330	rs9257809	1f
chr2	227179629	rs925735	6
chr4	187678865	rs925642	5
chr2	28614793	rs925255	4
chr4	17919810	rs925098	6
chr1	67760139	rs924080	5
chr6	170379024	rs924043	5
chr11	12072502	rs923175	5
chr8	126534670	rs921720	5
chr8	19651160	rs920590	5
chr7	121018578	rs917727	5
chr21	43275494	rs915832	5
chr9	82310897	rs914715	5
chr20	48955423	rs913678	4
chr13	99131293	rs912330	4
chr9	841151	rs912062	2b
chr14	68753592	rs911263	5
chr20	23612736	rs911119	4
chr20	33171771	rs910873	6
chr1	30567595	rs910696	5
chr14	75626041	rs910316	4
chr6	32315726	rs910049	6
chr1	22573941	rs909814	5
chr11	1941945	rs909116	5
chr1	152454590	rs908922	2b
chr3	140540717	rs908821	6
chr1	235092599	rs908327	5
chr11	1874071	rs907611	2a
chr4	26137018	rs907499	5
chr6	37451695	rs904251	5
chr12	53273903	rs902774	2a
chr10	45966421	rs901683	2b
chr5	178699310	rs901254	5
chr3	156798774	rs900400	6
chr10	44458713	rs898549	5
chr8	95960510	rs896854	5
chr2	45188352	rs895636	5
chr3	142894410	rs894177	5

chr4	89203669	rs893971	6
chr15	74229064	rs893817	4
chr17	75400099	rs892961	4
chr3	397552	rs892295	4
chr19	29912293	rs892073	5
chr5	147225513	rs891992	6
chr8	130491751	rs891835	5
chr6	118578042	rs89107	6
chr5	175956270	rs890835	5
chr16	79645988	rs889472	6
chr5	56031883	rs889312	3a
chr5	172984113	rs889014	5
chr2	59302876	rs887912	6
chr16	11158884	rs887864	5
chr16	3023604	rs886427	4
chr6	30782001	rs886424	1b
chr12	131621761	rs885389	4
chr9	93181530	rs883924	6
chr2	136976254	rs882300	6
chr6	43806608	rs881858	4
chr21	48019867	rs881827	5
chr9	123652897	rs881375	2b
chr1	10796865	rs880315	5
chr6	31139451	rs879882	5
chr3	41137671	rs87938	6
chr16	73068677	rs879324	5
chr14	57221138	rs878889	6
chr10	50042950	rs877819	6
chr1	159674932	rs876537	5
chr19	16467758	rs875622	5
chr4	26108196	rs874040	2b
chr1	40132794	rs873917	4
chr9	126154353	rs872863	5
chrX	37854726	rs872690	5
chr4	54799244	rs871606	4
chr12	42171981	rs871392	5
chr16	5585851	rs870288	6
chr10	112909104	rs869244	5
chr9	129465324	rs867559	5
chr1	167411383	rs864537	3a
chr3	186554291	rs864265	6
chr14	74990745	rs862034	5
chr1	209977110	rs861020	4
chr17	15826020	rs859267	5
chr1	158612547	rs857721	6
chr1	158575728	rs857684	6
chr7	149203661	rs855913	6

chr7	94954695	rs854572	5
chr7	94930390	rs854555	6
chr14	25271519	rs854384	6
chr8	133929916	rs853308	5
chr20	17122592	rs852069	6
chr7	28185090	rs849141	4
chr7	28196221	rs849134	6
chr7	108548659	rs848353	5
chr6	34923863	rs847845	6
chr2	61091949	rs842636	5
chr2	188196468	rs840616	6
chr14	75916463	rs84044	3a
chr1	167408669	rs840016	4
chr12	125261592	rs838880	5
chr2	234273241	rs838705	5
chr1	59762467	rs835367	2b
chr5	52020395	rs830884	5
chr9	33217265	rs830407	6
chr12	122395776	rs830124	6
chr2	222801698	rs824931	6
chr1	205764639	rs823156	5
chr1	205713377	rs823128	4
chr3	186480205	rs822354	6
chr19	33909709	rs8182584	5
chr12	109025426	rs8179116	5
chr9	110125810	rs817858	5
chr9	136135551	rs8176704	5
chr3	39553787	rs816488	5
chr9	84222617	rs815847	4
chr2	47399063	rs815815	4
chr22	26394723	rs8139900	6
chr3	99592595	rs813218	5
chr21	47165428	rs8127571	5
chr20	38644557	rs8120917	6
chr20	34865239	rs8115222	5
chr19	10520063	rs8112449	6
chr19	46034557	rs8111589	6
chr19	46307405	rs8111071	5
chr19	10213153	rs8109578	2b
chr19	16185558	rs8109288	5
chr19	58469015	rs8106858	6
chr19	40170052	rs8103033	4
chr19	38735612	rs8102476	6
chr19	30296852	rs8102137	5
chr19	33364627	rs8101881	5
chr19	42521107	rs8099939	5
chr19	39743164	rs8099917	4

chr18	46991159	rs8099594	3a
chr18	56109858	rs8099014	6
chr15	61329787	rs809736	5
chr18	48713039	rs8096445	5
chr18	57503377	rs8093763	4
chr18	68142413	rs8092443	6
chr18	7068461	rs8090011	6
chr18	10078070	rs8089099	5
chr18	40288292	rs8085804	6
chr18	74952757	rs8084125	5
chr18	3522005	rs8083633	5
chr18	22731359	rs8083432	6
chr17	68190825	rs8079702	6
chr17	38166878	rs8078723	1f
chr17	55823551	rs8077059	5
chr17	27714586	rs8076739	5
chr22	19154521	rs807669	4
chr17	8943928	rs8076457	5
chr2	15782470	rs807624	5
chr17	56010959	rs8074980	5
chr17	51237940	rs8073783	5
chr17	55119993	rs8073390	6
chr17	44081063	rs8070723	6
chr17	33843511	rs8070473	5
chr17	45768835	rs8070463	6
chr17	59483765	rs8068318	5
chr6	26200676	rs806794	4
chr17	38051347	rs8067378	1f
chr17	70696102	rs8066857	5
chr17	54203359	rs8065311	6
chr16	56725172	rs8064100	5
chr13	50841322	rs806321	3a
chr6	91207350	rs806276	4
chr16	83709665	rs8058532	6
chr16	27434926	rs8057551	6
chr16	85092747	rs8056742	6
chr6	31616365	rs805303	6
chr6	31622605	rs805297	1f
chr16	88777241	rs8052560	5
chr16	57563670	rs8052123	4
chr16	62100210	rs8050940	6
chr16	4646877	rs8050907	5
chr16	66158735	rs8050896	6
chr16	53816274	rs8050136	4
chr16	11691752	rs8049607	4
chr16	28837514	rs8049439	1b
chr16	69135048	rs8047014	5

chr16	53839134	rs8044769	4
chr8	11612697	rs804280	1f
chr15	91521336	rs8042680	6
chr15	49939845	rs8039808	6
chr15	74305965	rs8039584	4
chr15	73978336	rs8038465	5
chr15	41196383	rs8036080	2a
chr15	74716608	rs8036030	4
chr15	29006092	rs8033165	6
chr15	56194876	rs8032158	6
chr15	78816056	rs8031948	6
chr15	69059083	rs8030672	6
chr15	75718668	rs8028182	1d
chr15	89249138	rs8027587	5
chr15	25602949	rs8025093	6
chr15	96708290	rs8023580	6
chr15	49192790	rs8023445	1f
chr14	90298274	rs8021963	6
chr14	51170878	rs8020441	6
chr14	67453857	rs8020095	6
chr14	90679909	rs8017423	6
chr14	103563194	rs8017161	4
chr14	35832665	rs8016947	4
chr14	97393684	rs8016620	5
chr14	96122407	rs8015016	6
chr14	75322793	rs8014204	1f
chr7	66030611	rs801193	6
chr1	228997834	rs801114	5
chr1	228996621	rs801112	5
chr14	101690044	rs8008758	5
chr14	66262962	rs8007846	5
chr14	93501025	rs8006385	4
chr14	96027152	rs8005962	4
chr14	88472594	rs8005161	5
chr14	90034971	rs8004664	6
chr13	92015976	rs8002779	6
chr13	48387721	rs8001976	6
chr13	80692810	rs8001641	5
chr13	94233128	rs7998314	6
chr13	113331867	rs7998202	6
chr13	102000944	rs7996217	6
chr13	40350911	rs7993214	4
chr13	100555037	rs7992643	5
chr4	1734238	rs798766	6
chr7	2763101	rs798544	1f
chr7	2789879	rs798502	1b
chr7	2795956	rs798497	1f

chr7	2801802	rs798489	5
chr13	86690526	rs7984869	6
chr13	80623345	rs7981942	3a
chr12	123822710	rs7980687	5
chr12	121420259	rs7979473	5
chr12	52251271	rs7976059	6
chr12	54159276	rs7969151	1f
chr3	120017479	rs796825	6
chr12	131862902	rs7965445	5
chr12	74087807	rs7964120	6
chr12	73828955	rs7963521	6
chr12	122365582	rs7961894	4
chr12	71663101	rs7961581	5
chr12	122625991	rs7953704	5
chr12	28017158	rs7953528	6
chr12	121403723	rs7953249	1f
chr11	92651001	rs7950811	6
chr11	5225446	rs7950726	6
chr9	79610627	rs79460104	6
chr11	47336319	rs7944584	5
chr3	4437357	rs794185	4
chr11	122522374	rs7941030	6
chr11	10669227	rs7940646	3a
chr11	11504227	rs7940423	5
chr11	56242260	rs7939886	6
chr19	41302705	rs7937	5
chr11	46722220	rs7932354	4
chr11	68994496	rs7931342	6
chr10	31415105	rs793108	6
chr11	34805848	rs7929679	4
chr11	76301374	rs7927997	6
chr11	76301315	rs7927894	5
chr11	12698039	rs7926971	4
chr10	94481916	rs7923837	6
chr10	53493472	rs7922491	6
chr10	90818376	rs7920888	6
chr10	76808759	rs7919006	4
chr10	80925576	rs7916441	5
chr9	16000234	rs79156074	5
chr10	104775907	rs7914558	6
chr10	105714398	rs7913069	6
chr10	45319653	rs7911712	5
chr10	65138909	rs7910927	6
chr10	68374083	rs7909235	6
chr1	114607363	rs79079833	6
chr10	34231274	rs7905537	5
chr11	83620786	rs790356	4

chr10	114758348	rs7903146	5
chr10	114754087	rs7901695	6
chr3	122380922	rs790116	6
chr10	66448471	rs7900909	6
chr3	194327097	rs789852	3a
chr10	20792333	rs7897194	6
chr10	3155172	rs7896691	5
chr10	65104499	rs7896518	6
chr12	70331826	rs789560	6
chr10	23434743	rs7893600	5
chrX	29640817	rs7890572	6
chr4	82150005	rs788867	6
chr16	78335043	rs78867184	6
chrX	6220473	rs7885458	3a
chr9	137704781	rs7874142	5
chr9	119134795	rs7869550	5
chr9	116991070	rs7868992	5
chr10	26774693	rs786870	1f
chr9	132444098	rs7866070	5
chr9	130619636	rs7865146	5
chr9	16368731	rs7864648	4
chr9	71229178	rs7864204	5
chr9	108936673	rs7861820	5
chr6	150380883	rs78546022	5
chr9	126352217	rs7852296	4
chr9	133478826	rs7851693	6
chr9	100549012	rs7850258	5
chr9	139111869	rs7849585	5
chr8	122908502	rs7844723	6
chr5	77987523	rs784420	5
chr8	19845375	rs7841189	2c
chr16	11222237	rs78394940	5
chr8	128539359	rs7837688	6
chr8	142540924	rs7837045	2b
chr15	83254707	rs783540	6
chr8	63883592	rs7834588	3a
chr8	57100148	rs7833986	5
chr8	18707870	rs7833787	5
chr8	23818686	rs7833268	5
chr6	161137989	rs783147	6
chr8	142300314	rs7827290	2b
chr8	69563694	rs7825271	6
chr8	11104110	rs7824557	6
chr8	52216761	rs7821565	6
chr8	78093836	rs7821178	5
chr8	82928597	rs7820074	6
chr8	11045160	rs7819412	6

chr8	19786890	rs7816032	5
chr8	129427517	rs7815944	6
chr8	57179019	rs7815788	6
chr8	36459453	rs7814403	6
chr8	11340180	rs7812879	6
chr7	98760503	rs7809799	5
chr7	117067821	rs7808424	5
chr7	151407800	rs7805747	5
chr7	26891664	rs7804356	6
chr7	100458092	rs7801190	5
chr2	27741236	rs780094	3a
chr2	27742602	rs780093	5
chr2	27743153	rs780092	6
chr7	47779968	rs7800244	5
chr7	151546588	rs7795096	5
chr7	94009633	rs7792596	6
chr7	75951229	rs7789940	6
chr7	40965126	rs7789197	6
chr7	129023596	rs7787531	5
chr7	158223105	rs7786808	4
chr7	116577123	rs7782376	5
chr7	96133530	rs7781370	6
chr7	75975585	rs7779014	4
chr7	10149975	rs7778619	5
chr7	36546068	rs7777593	5
chr7	121033120	rs7776725	5
chr6	135418634	rs7775698	3a
chr6	32658078	rs7775228	1d
chr6	32657577	rs7774434	1f
chr6	149435110	rs7772697	5
chr6	30795170	rs7772131	5
chr6	14598819	rs7770731	2b
chr6	131256363	rs7769153	5
chr6	133146795	rs7769051	6
chr6	20686572	rs7766070	3a
chr6	51099255	rs7762246	5
chr6	29970588	rs7758512	6
chr6	32345282	rs7758128	5
chr6	20679708	rs7756992	5
chr6	46675024	rs7756935	6
chr6	30848252	rs7756521	5
chr3	112995073	rs775227	4
chr6	152791473	rs7747960	6
chr6	106435268	rs7746082	6
chr6	32859711	rs7744666	6
chr6	35322762	rs7744392	3a
chr7	97924553	rs77443149	4

chr6	31336099	rs7743761	6
chr6	34165720	rs7742369	4
chr6	77789807	rs7738636	6
chr5	135214676	rs7734448	5
chr5	147856332	rs7733088	6
chr5	7228046	rs7729273	6
chr5	4720584	rs7727102	6
chr5	618585	rs7726839	5
chr5	31927316	rs7719829	6
chr3	101748637	rs771767	5
chr5	66833256	rs7717572	5
chr5	44875004	rs7716600	6
chr5	150270419	rs7714584	6
chr1	34750935	rs771390	6
chr5	60731457	rs7709645	3a
chr5	122774784	rs7705033	4
chr5	74625486	rs7703051	6
chr5	88088438	rs770189	5
chr4	88755827	rs7698623	6
chr4	114000955	rs7694725	6
chr4	7423750	rs7694661	5
chr19	45410001	rs769449	4
chr4	88186508	rs7694379	6
chr4	86641148	rs7692808	6
chr4	145568351	rs7689420	4
chr3	111201784	rs76884941	5
chr4	176859025	rs7687921	6
chr4	189584352	rs7686384	5
chr4	123523874	rs7682241	4
chr18	50285397	rs768048	6
chr4	106061533	rs7679673	5
chr9	124098534	rs767770	5
chr4	116934078	rs7676999	6
chr4	182399694	rs7672826	5
chr13	111470037	rs767210	5
chr4	10056375	rs7671266	4
chr4	11674660	rs7671189	6
chr4	89883978	rs7671167	6
chr1	19718823	rs7667	5
chr4	190123317	rs7665939	5
chr4	99796783	rs7665590	6
chr4	103551602	rs7665090	6
chr4	86651463	rs7660702	6
chr4	122665513	rs7659604	5
chr4	189587078	rs7659062	5
chr4	1254534	rs7656416	5
chr3	15648003	rs7651039	4

chr3	43467894	rs7650267	5
chr3	185834289	rs7647305	6
chr3	158453278	rs7646881	3a
chr3	172476418	rs7646507	6
chr3	86916881	rs7642134	5
chr3	69173374	rs7638995	6
chr3	191667776	rs7635839	5
chr2	47022542	rs76351433	6
chr3	177766463	rs7634528	6
chr3	143056466	rs7632299	5
chr3	4703936	rs7632000	6
chr3	37234588	rs7631605	6
chr3	179661317	rs7630877	2b
chr16	675679	rs763014	5
chr3	87241496	rs7629490	6
chr3	70626359	rs7628219	6
chr3	167348837	rs7627289	5
chr3	156836905	rs7624327	6
chr3	177304297	rs7620503	6
chr3	52279593	rs7618915	1f
chr3	28705763	rs7617877	5
chr3	49210731	rs7617480	1b
chr3	132727902	rs7617456	6
chr3	71115750	rs7616330	4
chr3	46205685	rs7616215	5
chr3	33065338	rs7613051	6
chr3	103307641	rs7612581	6
chr3	177596988	rs7612209	4
chr3	189343423	rs7610017	5
chr2	61204855	rs7608910	6
chr2	23948952	rs7608623	6
chr2	219279096	rs7607369	6
chr2	237521841	rs7607316	5
chr2	228135179	rs7606754	6
chr2	200676925	rs7605378	6
chr2	206836611	rs7603514	6
chr2	14801500	rs7602441	4
chr2	35093292	rs7600259	6
chr2	198962242	rs7595412	6
chr2	161171453	rs7593730	6
chr2	68646782	rs7592330	5
chr2	228715374	rs7591163	6
chr2	216898657	rs7590720	5
chr2	43540124	rs7590268	3a
chr7	75953296	rs758944	6
chr2	134363031	rs7588567	5
chr2	213168767	rs7588550	6

chr2	8949069	rs7587928	6
chr2	39843196	rs7587205	5
chr17	3786906	rs758642	4
chr2	148478335	rs7584099	6
chr16	4257312	rs75825892	3a
chr2	15664755	rs7581919	5
chr2	121195180	rs7581710	5
chr2	30445025	rs7579944	4
chr2	46537603	rs7579899	5
chr2	227020652	rs7578326	4
chr2	99382891	rs7578035	2a
chr2	56008903	rs7577894	3a
chr2	85699749	rs7577642	5
chr5	137707314	rs757647	1f
chr17	59497276	rs757608	5
chr2	233077063	rs7571816	2b
chr2	135837905	rs7570971	6
chr2	79709353	rs7570469	6
chr2	21100777	rs7569328	5
chr2	178684719	rs7567851	5
chr2	127982644	rs7567389	5
chr2	134434823	rs7567288	6
chr5	133446574	rs756699	5
chr2	176654737	rs7566934	6
chr2	101424330	rs7565981	5
chr2	36673554	rs7562790	5
chr1	184002873	rs756199	5
chr2	127889636	rs7561528	6
chr2	644952	rs7561317	6
chr2	24247513	rs7561273	1f
chr2	171256596	rs7561268	5
chr2	151637935	rs7560163	5
chr2	21208210	rs7557067	4
chr1	165718978	rs7555523	6
chr1	237266602	rs7554607	5
chr1	200877561	rs7554511	4
chr1	159698548	rs7553007	5
chr1	84254550	rs7552393	5
chr1	25273199	rs7551188	5
chr9	100696202	rs755109	6
chr1	247675558	rs7550918	6
chr1	38602072	rs7547921	5
chr1	207938235	rs7545126	6
chr1	97164342	rs7544736	3a
chr10	79680433	rs754466	5
chr1	240987753	rs7544372	4
chr1	100049784	rs7543130	5

chr1	95070040	rs7542900	5
chr1	221111779	rs7542375	6
chr13	24293858	rs753955	6
chr1	17722362	rs7538876	5
chr1	26741543	rs7532866	6
chr1	157670815	rs7528684	4
chr1	210536024	rs7527939	2b
chr1	207872289	rs7527798	4
chr1	156341493	rs7525133	3a
chr1	104019630	rs7524694	6
chr1	22698446	rs7524102	5
chr1	200881594	rs7522462	5
chr1	22490723	rs7521902	5
chr1	236313126	rs7520258	5
chr1	195914272	rs7517337	6
chr1	93009437	rs7515577	5
chr1	200672905	rs7512898	1f
chr22	50676258	rs7511006	6
chr19	3428833	rs7507204	2b
chr18	11987271	rs7506045	6
chr18	50517775	rs7504990	6
chr15	74241505	rs750460	5
chr11	125172592	rs750338	5
chr17	36101155	rs7501939	5
chr16	57006589	rs7499892	4
chr15	92552028	rs7495052	6
chr14	29021927	rs7493138	5
chr6	6283665	rs749005	6
chr15	43559230	rs748404	1f
chr11	115398745	rs7483296	2b
chr10	37982096	rs7474896	6
chr9	133464083	rs7466269	2b
chr8	143691837	rs7465272	5
chr11	109995943	rs746463	6
chr8	118909259	rs7459527	5
chr7	26008051	rs745580	4
chr6	32730011	rs7453920	6
chr2	76759574	rs74529274	6
chr14	52989227	rs745080	1f
chr15	67446784	rs744910	3a
chr5	104867935	rs7447447	5
chr15	96372628	rs744738	5
chr4	29903051	rs7442317	6
chr17	40514200	rs744166	4
chr22	27042105	rs744016	5
chr22	37551606	rs743777	4
chr3	64727085	rs7433808	5

chr22	43518274	rs742134	1f
chr6	106582919	rs742108	5
chr1	18979873	rs742071	4
chr22	27435819	rs742004	5
chr19	13024249	rs741702	5
chr1	153848995	rs7414227	1f
chr1	150860470	rs7412746	1f
chr19	1124834	rs740495	5
chr16	23888839	rs7404928	4
chr16	23864589	rs7404095	1f
chr16	6156325	rs7403856	5
chr10	118575605	rs740363	1f
chr15	38822904	rs7403531	5
chr7	29921523	rs740145	2b
chr11	116684027	rs7396835	1f
chr11	48518892	rs7395662	6
chr11	3036323	rs739401	5
chr22	27051298	rs739310	5
chr22	29130011	rs738722	6
chr8	142359549	rs7386474	1f
chr22	38569005	rs738322	4
chr3	184945848	rs7374394	6
chr20	55990404	rs737092	2b
chr12	54417575	rs736825	3a
chr22	42218855	rs7364180	4
chr3	52835353	rs736408	1f
chr19	33757061	rs736289	5
chr20	2800800	rs7360412	3a
chr16	28885658	rs7359397	1f
chr15	67702906	rs7359257	5
chr6	53123117	rs735860	4
chr22	36679057	rs735854	5
chr6	22753046	rs7356884	6
chr11	123361396	rs735665	4
chr13	21280033	rs735539	5
chr12	121438843	rs735396	4
chr4	5758868	rs735172	5
chr1	2513215	rs734999	1f
chr2	219756382	rs7349332	5
chr4	9923003	rs734553	5
chr12	6291092	rs7342306	5
chr10	114479261	rs7342028	6
chr13	100041737	rs7335046	5
chr13	112221296	rs7333181	5
chr13	33147547	rs7332115	6
chr4	10050140	rs733175	6
chr13	53517883	rs732949	6

chr13	41558109	rs7329174	4
chr13	92501663	rs7328464	6
chr14	75365728	rs732765	4
chr13	36771016	rs7327064	6
chr13	21209511	rs7326068	6
chr18	48378233	rs732528	6
chr19	5582534	rs732505	5
chr13	24296861	rs7324557	4
chr13	88704705	rs7323893	5
chr13	45900266	rs7323755	2b
chr13	111030577	rs7319311	5
chr13	22709669	rs7318731	5
chr19	33899064	rs731839	5
chr13	114012897	rs7317038	6
chr12	132085195	rs7315621	5
chr12	115891402	rs7315438	5
chr12	2523354	rs7312105	6
chr1	38196840	rs731174	5
chr12	121424860	rs7310409	5
chr12	5981352	rs7307889	4
chr14	101142889	rs730570	4
chr3	48487047	rs730566	1f
chr12	121402931	rs7305618	5
chr14	52518810	rs730532	5
chr12	63004582	rs7302017	6
chr12	131390753	rs7299940	1e
chr11	121243715	rs72991	5
chr6	43804570	rs729761	2b
chr12	117327591	rs7294919	6
chr12	114637422	rs7294372	6
chr7	128568959	rs729302	5
chr21	32216252	rs7283316	6
chr17	7552122	rs72829446	5
chr17	46123003	rs72823592	4
chr7	120305148	rs728115	6
chr21	43227914	rs7279297	5
chr15	51534546	rs727479	6
chr17	7537791	rs727428	2b
chr20	3193892	rs7270101	6
chr20	25298086	rs7267979	6
chr8	71139329	rs72663955	5
chr19	45432556	rs7259004	6
chr19	8271720	rs7258249	5
chr19	8433195	rs7255436	4
chr19	45146102	rs7255066	5
chr19	1803581	rs7253430	5
chr4	128342119	rs724950	6

chr19	8671999	rs7249094	5
chr19	18467885	rs7248363	1f
chr14	76129590	rs724743	4
chr18	7755770	rs7243299	5
chr18	11282318	rs7243066	6
chr18	47159089	rs7240405	5
chr3	141105569	rs724016	4
chr18	46395021	rs7240004	4
chr18	56384191	rs7238078	6
chr18	4773109	rs7237848	6
chr18	77392378	rs7233060	5
chr18	23393103	rs7231041	5
chr18	47160813	rs7228085	6
chr8	516478	rs722782	6
chr18	43187129	rs7227483	5
chr18	37165304	rs7226835	6
chr17	53364787	rs7224610	4
chr17	58966537	rs7224438	4
chr17	38770285	rs7221109	6
chr17	79118572	rs7220048	5
chr17	72268207	rs7219585	5
chr17	38923	rs7217319	1f
chr17	38069948	rs7216389	6
chr17	65898808	rs7216064	2b
chr17	78737286	rs7215564	4
chr2	63131730	rs721048	5
chr17	64133725	rs7209395	6
chr17	37543448	rs7208487	5
chr17	45425114	rs7206971	6
chr16	661334	rs7204439	4
chr16	67693796	rs7204371	4
chr16	11641179	rs7203193	5
chr16	75247244	rs7202877	5
chr16	53821614	rs7202116	2b
chr16	11177800	rs7200786	5
chr16	73009023	rs7199343	6
chr16	68383046	rs7197653	5
chr16	30642866	rs7197475	6
chr2	42004996	rs719593	5
chr16	24578457	rs7195386	6
chr16	69563889	rs7193778	4
chr16	73029159	rs7193343	2b
chr16	76318541	rs7193230	6
chr16	79363813	rs7191820	5
chr16	11406802	rs7191700	4
chr16	58622177	rs7188697	5
chr16	72220372	rs7186908	6

chr16	30635658	rs7186852	1f
chr15	84573040	rs7183263	2b
chr15	96844726	rs7181753	5
chr15	40360740	rs7181230	4
chr15	90447945	rs7178909	1f
chr15	62380258	rs7178424	2b
chr15	70018989	rs7176508	6
chr4	10104669	rs717615	5
chr15	94036687	rs7175404	6
chr15	62396941	rs7173964	5
chr15	62396388	rs7172432	5
chr15	38907040	rs7171171	5
chr15	31463377	rs7169523	1f
chr15	56340895	rs7169431	5
chr15	93638400	rs7168353	6
chr10	112006485	rs716595	6
chr15	73652173	rs7164883	3a
chr15	68715031	rs7164335	5
chr15	92211773	rs7164176	3a
chr15	78698758	rs7163013	5
chr14	65758641	rs7159888	5
chr14	72883873	rs7159300	4
chr13	24795488	rs715921	4
chr14	96169130	rs7158782	6
chr14	64235555	rs7157785	5
chr14	76703350	rs7156960	5
chr14	65502238	rs7155454	5
chr14	92485880	rs7155279	6
chr14	92427221	rs7153027	6
chr14	84597384	rs7152554	6
chr14	101159415	rs7149242	1f
chr14	65865624	rs7147624	6
chr14	70644168	rs7146198	6
chr14	64275811	rs7144584	6
chr14	61734639	rs7141238	6
chr7	72864868	rs714052	6
chr12	50247467	rs7138803	6
chr22	30592486	rs713875	1f
chr12	119989645	rs7137869	6
chr12	90081187	rs7136259	6
chr2	25158007	rs713586	1f
chr12	68500074	rs7134599	5
chr12	110000192	rs7134594	5
chr12	20473757	rs7134375	5
chr22	19156116	rs712964	3a
chr11	10350537	rs7129220	4
chr11	2233573	rs7127900	5

chr11	64313290	rs7124676	4
chr11	66662730	rs7122539	5
chr11	121954656	rs7121446	5
chr17	35850552	rs712039	4
chr11	22843154	rs712022	6
chr11	116829652	rs7120173	6
chr11	47286289	rs7120118	1f
chr11	103525753	rs7118412	6
chr11	128436952	rs7117932	5
chr11	47127152	rs7117404	1f
chr11	66826159	rs7112925	5
chr11	117037360	rs7112513	5
chr11	22829756	rs7111546	4
chr11	2213165	rs7111341	5
chr11	15710083	rs7108738	4
chr4	82149830	rs710841	6
chr11	129473689	rs7107217	5
chr11	69239740	rs7105934	3a
chr3	189645932	rs710521	6
chr11	112064430	rs7105056	6
chr11	103526023	rs7103004	6
chr11	69143283	rs7102705	5
chr10	61449563	rs7094971	6
chr10	22847438	rs7094131	6
chr10	63830285	rs7090871	5
chr10	6110828	rs7090512	5
chr10	64187563	rs7089814	5
chr10	63752158	rs7089424	3a
chr10	114498475	rs7086803	4
chr4	57821308	rs708547	5
chr10	122939373	rs7085142	6
chr10	101813801	rs7084921	5
chr17	42442343	rs708382	6
chr10	31990622	rs7081678	5
chr6	14636962	rs707824	5
chr10	101274364	rs7078219	6
chr10	118827559	rs7078160	6
chr10	81705432	rs7078012	6
chr10	18759628	rs7076247	6
chr10	65050658	rs7075195	6
chr10	71099912	rs7072268	3a
chr10	100156852	rs7072216	6
chr10	79401315	rs7071206	4
chr10	12277991	rs7068966	1f
chr10	6098823	rs706779	5
chr10	6098948	rs706778	5
chrX	53974053	rs7065696	6

chr12	56390635	rs705702	1f
chr10	3667725	rs705471	5
chr10	3658918	rs705469	6
chr9	26935995	rs7045881	4
chr9	137568050	rs7044529	5
chr9	8196510	rs7044355	6
chr9	85135914	rs7043482	6
chr9	77149836	rs7042950	5
chr9	110872306	rs7042864	6
chr9	4287465	rs7041847	4
chr10	80841147	rs704010	2b
chr9	845515	rs7040024	6
chr10	80955066	rs703965	4
chr9	4289049	rs7034200	6
chr9	130110410	rs7029536	4
chr9	565916	rs7027930	6
chr9	124422402	rs7025486	5
chr5	58842773	rs702543	5
chr9	4291746	rs7020673	5
chr9	22137684	rs7018475	4
chr8	71591202	rs7017914	1f
chr8	19876745	rs7016880	6
chr8	90875917	rs7015630	6
chr22	20228541	rs701428	5
chr8	120647928	rs7007970	5
chr7	46753552	rs700752	4
chr7	46753490	rs700750	3a
chr8	72270081	rs7006821	6
chr8	41615137	rs7006290	4
chr8	89760310	rs7004633	6
chr8	108040351	rs7004587	6
chr8	96127029	rs7000734	5
chr8	145005560	rs6995402	5
chr8	106581527	rs6993770	2b
chr14	72692492	rs699363	6
chr8	141150168	rs6992848	5
chr8	68799376	rs6990917	5
chr8	126504725	rs6987702	6
chr8	34524002	rs6987004	5
chr8	9178267	rs6984305	4
chr8	131092412	rs6984045	2b
chr8	128106879	rs6983561	6
chr8	128413304	rs6983267	4
chr8	66463477	rs6981992	6
chr7	154072019	rs6977820	6
chr6	16742032	rs697739	5
chr7	100512118	rs6976053	1f

chr7	2582451	rs6972204	5
chr7	17287268	rs6968865	5
chr7	110047470	rs6968385	4
chr7	46242582	rs6964415	6
chr7	67426322	rs6961611	6
chr7	112094742	rs6956741	5
chr7	62577769	rs6956675	5
chr7	76639870	rs6955651	2b
chr7	2448492	rs6952809	3a
chr7	1886534	rs6952808	5
chr7	37757555	rs6951258	6
chr7	36610492	rs6948404	5
chr11	64097232	rs694739	1f
chr7	68611959	rs6945541	6
chr18	60126111	rs694419	5
chr7	69806022	rs6943555	6
chr7	72746647	rs6943090	6
chr7	155762156	rs6943029	5
chr7	81782513	rs6942458	5
chr6	4512978	rs6942328	5
chr6	15089150	rs6941421	2b
chr6	34683634	rs6938239	4
chr6	27248930	rs6932590	6
chr6	144079853	rs6931865	4
chr6	148788005	rs6930337	6
chr6	252144	rs6927090	2b
chr6	32612396	rs6927022	6
chr6	71660110	rs6922893	5
chr6	24107092	rs6922632	5
chr6	138006503	rs6920220	6
chr6	34238513	rs6918981	6
chr6	160402704	rs6917747	4
chr6	74871230	rs6912405	5
chr6	32282853	rs6910071	6
chr6	20728730	rs6908425	6
chr6	132228768	rs6907728	6
chr6	29943066	rs6904029	1f
chr6	31168028	rs6903896	5
chr6	28322295	rs6903823	2b
chr6	152157880	rs6902771	6
chr6	81481353	rs6902257	6
chr6	130358427	rs6899976	4
chr5	40424425	rs6896969	6
chr5	179032487	rs6894268	4
chr5	129792133	rs6894216	6
chr5	35852310	rs6890853	4
chr13	98966316	rs688872	5

chr5	31020520	rs6888304	6
chr5	11169944	rs6885224	6
chr5	11111770	rs6884431	6
chr5	156390296	rs6882076	6
chr5	77630853	rs6881634	5
chr22	26689634	rs688034	6
chr5	2109900	rs6879627	5
chr5	179731013	rs6879260	4
chr9	136137064	rs687621	5
chr5	3092191	rs6873793	6
chr9	136137105	rs687289	5
chr5	158826791	rs6871626	6
chr5	30899516	rs6871087	6
chr5	33636593	rs6868223	6
chr5	141445979	rs6867913	5
chr5	141513203	rs6863411	1f
chr5	173362457	rs6861681	6
chr5	55438579	rs6859219	2b
chr4	189120910	rs6857559	5
chr4	62437317	rs6856328	6
chr4	123478715	rs6852535	6
chr1	48025062	rs685001	5
chr4	110800096	rs6847149	5
chr4	102402214	rs6846071	6
chr4	148974601	rs6845865	4
chr4	26944313	rs6844153	6
chr4	148400818	rs6842241	5
chr4	113328077	rs6838440	6
chr4	10062325	rs6834555	6
chr4	35983110	rs6834483	6
chr3	182869817	rs683395	3a
chr4	40303632	rs6832151	4
chr4	18017729	rs6830062	5
chr5	17148910	rs682748	6
chr6	63160146	rs682238	6
chr4	27000994	rs6822297	5
chr4	30068212	rs6818288	6
chr4	111705767	rs6817105	6
chr4	73783403	rs6816344	6
chr4	1309900	rs6815464	4
chr4	137660382	rs6813479	5
chr4	77198985	rs6812193	5
chr3	186548564	rs6810075	5
chr3	10535770	rs6807064	3a
chr3	69252898	rs6806528	4
chr3	183843134	rs6806377	6
chr20	12969399	rs680379	6

chr3	11625413	rs6802119	5
chr3	38767314	rs6801957	4
chr3	176030095	rs6799767	5
chr3	35982129	rs6799705	6
chr3	96328826	rs6796026	5
chr3	64705364	rs6795735	6
chr3	171558343	rs6794092	6
chr3	67531387	rs6792584	5
chr3	60771107	rs6791644	5
chr3	14480777	rs6790433	5
chr1	172189888	rs678962	6
chr3	150467807	rs6788895	5
chr3	52506425	rs6784615	5
chr3	11675788	rs6782029	4
chr11	114431955	rs678170	5
chr3	32378270	rs6776297	6
chr3	141266492	rs6776003	6
chr3	59354157	rs6771019	5
chr3	53100213	rs6770152	6
chr3	185530289	rs6769511	4
chr3	57779835	rs6768930	3a
chr3	12551806	rs6766510	6
chr3	141102832	rs6763931	5
chr3	1512586	rs6763848	6
chr3	38681393	rs6763048	5
chr3	50093208	rs6762477	6
chr2	68650996	rs6759808	5
chr2	217208570	rs6756590	5
chr2	26526418	rs6753473	6
chr2	50760502	rs6750634	6
chr2	169041385	rs6749447	5
chr2	171461131	rs6749331	6
chr2	68070224	rs6747972	5
chr2	97410948	rs6746896	5
chr2	25659243	rs6746082	5
chr2	240418659	rs6743931	4
chr6	32578081	rs674313	4
chr2	234672638	rs6742078	5
chr2	162910222	rs6741949	5
chr2	7147972	rs6741819	2b
chr2	38277831	rs6741148	6
chr2	65667271	rs6740462	4
chr2	11220561	rs6739054	5
chr2	198896894	rs6738825	6
chr1	35687814	rs673604	6
chr2	103770237	rs6735786	5
chr2	21237543	rs673548	5

chr2	1777149	rs6735179	5
chr2	113841029	rs6734238	5
chr2	34480074	rs6733379	5
chr2	25276283	rs6733301	6
chr2	219084532	rs6733051	6
chr2	43587503	rs6732426	4
chr2	3748970	rs6730148	5
chr2	19999345	rs6728440	6
chr2	203745884	rs6725887	6
chr2	219943845	rs6724465	6
chr2	135479979	rs6723108	6
chr2	121301910	rs6721654	5
chr2	111989371	rs6720394	2b
chr2	11510947	rs6716724	4
chr2	151126402	rs6716455	5
chr2	33361424	rs6714546	5
chr2	101922169	rs6711606	5
chr2	135592380	rs6710823	6
chr2	70368922	rs6708331	4
chr2	30526779	rs6708166	6
chr2	74208361	rs6705628	4
chr1	169550962	rs6703865	6
chr1	243608966	rs6703335	6
chr1	207786288	rs6701713	4
chr1	175120078	rs6701037	1f
chr1	66089781	rs6700896	3a
chr1	89123442	rs6699417	5
chr3	28071443	rs669607	6
chr1	53629584	rs6695567	6
chr1	44129778	rs669446	5
chr1	232519149	rs669408	3a
chr1	220977332	rs6693017	4
chr1	61791862	rs6691768	5
chr1	100268990	rs6689305	6
chr9	85311146	rs668853	6
chr1	160077853	rs6688363	4
chr1	159342438	rs6687840	6
chr1	222164947	rs6687758	6
chr1	18244706	rs6686929	6
chr1	156356479	rs6686886	4
chr1	41530870	rs6686842	6
chr1	58367235	rs6679454	6
chr1	196686917	rs6677604	5
chr15	78863471	rs667282	5
chr1	164739170	rs6670655	4
chr1	24300848	rs6667686	5
chr1	15347639	rs6667220	2b

chr1	154814267	rs6666258	5
chr1	57269520	rs6662617	4
chr18	76415449	rs66591657	4
chr1	3280252	rs6658356	5
chr8	129567180	rs6651252	4
chrX	116222262	rs6646773	6
chrX	66510983	rs6625163	6
chrX	47174275	rs6611365	6
chrX	44400185	rs6610953	6
chr1	44117005	rs660899	5
chr6	32577379	rs660895	1f
chr1	93303602	rs6604026	5
chr19	7424527	rs6603109	6
chr10	17142525	rs6602175	5
chr10	3862541	rs6601764	5
chr8	11638243	rs6601606	1f
chr8	10671271	rs6601530	4
chr8	9184690	rs6601299	6
chr1	121200489	rs6600671	3a
chr4	939112	rs6599389	5
chr4	939086	rs6599388	5
chr3	40096617	rs6599077	4
chr15	99271134	rs6598541	4
chr5	131742227	rs6596075	1f
chr1	170633895	rs659580	5
chr11	87125437	rs6592362	6
chr11	128311058	rs6590330	6
chr11	128206409	rs6590322	4
chr11	122870682	rs6589964	6
chr11	116652422	rs6589566	6
chr1	53978118	rs6588480	2b
chr1	17216330	rs6586513	4
chr21	44478496	rs6586282	5
chr10	82377589	rs6586111	5
chr10	82254046	rs6586030	1f
chr10	124165614	rs6585827	5
chr10	119210374	rs6585436	5
chr10	81933747	rs6585424	5
chr10	101290300	rs6584283	5
chr10	100170382	rs6584202	5
chr10	94347829	rs6583826	6
chr12	38743507	rs6582630	4
chr8	135593724	rs6577655	4
chr15	25894657	rs6576443	6
chr14	101032216	rs6575793	5
chr14	94205344	rs6575353	6
chr14	78786158	rs6574433	5

chr9	136139264	rs657152	5
chr5	131430117	rs657075	5
chr6	130349118	rs6569648	6
chr6	106829536	rs6568433	4
chr6	106435024	rs6568421	5
chr17	78348493	rs6565681	3a
chr16	83639334	rs6563943	6
chr13	84130231	rs6563353	6
chr8	9814410	rs656319	6
chr13	54435236	rs6561750	6
chr10	1676530	rs6560749	5
chr9	79038169	rs6560517	6
chr8	6152918	rs6559140	6
chr8	145139521	rs6558295	1f
chr5	158787384	rs6556412	6
chr5	180226672	rs655601	6
chr5	13740975	rs6554809	6
chr1	187902980	rs655167	5
chr3	36864488	rs6550435	6
chr2	634904	rs6548238	4
chr2	28646800	rs6547853	5
chr11	128586154	rs654723	5
chr2	62713532	rs6545946	5
chr2	61772256	rs6545883	6
chr2	25131315	rs6545814	1f
chr2	60534198	rs6545803	5
chr2	25118884	rs6545800	6
chr2	47844504	rs6544997	5
chr2	21204024	rs6544366	6
chr2	113529182	rs6542095	6
chr4	123198434	rs6534347	6
chr4	90797300	rs6532197	5
chr4	88773848	rs6532023	6
chr3	61794053	rs652889	6
chr11	31905533	rs652722	6
chrX	26369898	rs6526555	6
chr6	93713804	rs652520	6
chr22	46421841	rs6519955	2b
chr21	34542786	rs6517147	5
chr19	11202305	rs6511720	4
chr6	160581373	rs651164	5
chr19	4830627	rs6510827	5
chr19	348742	rs6510725	4
chr19	35973288	rs6510489	6
chr9	136153874	rs651007	5
chr17	52548124	rs6504909	6
chr17	48591208	rs6504663	5

chr17	46617018	rs6504340	1f
chr17	38095173	rs6503525	6
chr17	10574158	rs6503319	6
chr17	68291132	rs6501384	6
chr16	6817374	rs6500818	5
chr16	69830327	rs6499255	4
chr16	68674787	rs6499188	5
chr16	68326199	rs6499165	1f
chr16	11249328	rs6498169	1b
chr16	11081248	rs6498142	6
chr16	10630776	rs6498068	5
chr15	85825566	rs6496932	5
chr15	90893667	rs6496667	5
chr15	95511836	rs6496074	6
chr15	75125644	rs6495122	1f
chr9	136154303	rs649129	2b
chr13	20736215	rs6490525	5
chr12	121363723	rs6489785	1f
chr12	124203831	rs6488898	4
chr12	13927895	rs6488619	6
chr12	9371331	rs6487679	6
chr11	13361523	rs6486122	4
chr11	10390580	rs6484218	6
chr10	129678673	rs6482992	5
chr10	54574995	rs6480975	6
chr10	65006455	rs6479891	1f
chr9	99097526	rs6479272	6
chr9	117568765	rs6478109	4
chr9	117545665	rs6478106	5
chr9	115997248	rs6477998	6
chr9	289060	rs6476030	6
chr8	41549193	rs6474359	5
chr2	31464828	rs647316	4
chr8	78178484	rs6473015	5
chr5	134499091	rs647161	6
chr8	130725664	rs6470764	5
chr8	120044828	rs6469804	6
chr8	98329764	rs6468544	5
chr8	37686748	rs6468442	2b
chr1	109818529	rs646776	1f
chr7	137203818	rs6467710	6
chr7	127164957	rs6467136	6
chr7	77416438	rs6465825	5
chr7	97816326	rs6465657	4
chr7	3915563	rs6462411	6
chr7	157510194	rs6459804	5
chr6	42731114	rs6458307	4

chr6	32855716	rs6457690	6
chr6	32663998	rs6457620	6
chr6	32663850	rs6457617	6
chr6	31074029	rs6457327	1f
chr6	62697745	rs6455128	6
chr5	40410934	rs6451493	3a
chr3	135926621	rs645040	5
chr4	31397617	rs6448771	6
chr3	58370176	rs6445975	5
chr18	355943	rs644435	5
chr3	5013875	rs6442925	6
chr3	15440555	rs6442522	2b
chr9	136142216	rs644234	6
chr3	46428189	rs6441975	6
chr3	46352383	rs6441961	1f
chr3	159728877	rs6441286	6
chr3	117574821	rs6438424	6
chr2	229969797	rs6436839	4
chr21	44768561	rs643608	5
chr2	217878208	rs6435957	6
chr2	215672545	rs6435862	4
chr9	15296033	rs643531	1f
chr9	136142354	rs643434	5
chr6	139839422	rs643381	2b
chr2	9721895	rs6432018	5
chr2	136506926	rs6430585	6
chr1	209989269	rs642961	4
chr1	235600128	rs6429082	6
chr6	140273646	rs642858	6
chr1	196844592	rs6428370	6
chr11	65560619	rs642803	4
chr1	158058108	rs6427419	5
chr1	157130565	rs6427356	6
chr1	20171859	rs6426833	4
chr1	22711472	rs6426749	6
chr11	118743771	rs6421571	5
chr5	176817635	rs6420094	1f
chr13	110790231	rs641862	4
chr8	2740501	rs641525	3b
chr11	118698536	rs638893	5
chr6	167177421	rs635808	5
chr15	35006072	rs634990	5
chr11	75282051	rs634552	5
chr1	177852579	rs633715	5
chr11	118754352	rs630923	1f
chr13	34906054	rs627871	6
chr2	31482299	rs625132	4

chr17	700019	rs623323	5
chr4	75502486	rs62314947	5
chr4	1039214	rs62296075	5
chr3	175103613	rs62287976	6
chr18	322521	rs621636	4
chr11	88913662	rs621313	6
chr20	33867696	rs619865	2b
chr11	20984204	rs61883261	5
chr10	6094696	rs61839660	3a
chr17	17068181	rs61744862	5
chr6	32574170	rs615672	6
chr11	69328763	rs614367	5
chr12	120974509	rs614226	2b
chr20	3187732	rs6139030	6
chr18	53210301	rs613872	6
chr14	94796183	rs61280460	6
chr20	55638749	rs6127921	6
chr20	46208604	rs6125048	5
chr20	45497722	rs6124878	5
chr20	49375241	rs6122972	5
chr9	136143441	rs612169	5
chr20	33730386	rs6120849	6
chr20	8114703	rs6118083	5
chr20	4698625	rs6116492	5
chr20	14388214	rs6110278	5
chr20	819661	rs6108038	5
chr20	7552503	rs6108011	5
chr6	138199416	rs610604	4
chr20	56501978	rs6099847	6
chr6	152080831	rs60945108	6
chr20	52349891	rs6091737	5
chr20	61669035	rs6089829	4
chr20	33975180	rs6088813	1f
chr20	33909783	rs6088792	1f
chr20	33799279	rs6088765	5
chr20	7180055	rs6085920	6
chr20	6980634	rs6085820	6
chr20	1995533	rs6081597	6
chr20	1759589	rs6080550	5
chr15	45934868	rs607541	5
chr20	44740195	rs6074022	1f
chr20	56663273	rs6070346	4
chr20	56044183	rs6070116	3a
chr5	134572228	rs606854	6
chr20	50559651	rs6068020	6
chr20	45460322	rs6066084	5
chr20	44554014	rs6065906	5

chr20	44534650	rs6065904	4
chr11	64546390	rs606458	1f
chr11	75276177	rs606452	5
chr20	48019817	rs6063399	6
chr20	47343058	rs6063312	6
chr20	62409712	rs6062314	5
chr1	193921547	rs606149	4
chr20	33914207	rs6060373	1f
chr20	33907160	rs6060369	1f
chr20	7294902	rs6054906	6
chr20	4611876	rs6052699	6
chr20	351943	rs6051520	4
chr6	139829665	rs605066	6
chr20	2518564	rs6049839	5
chr20	24058406	rs6049375	5
chr20	22559600	rs6048205	4
chr20	19802187	rs6046346	5
chr20	1941170	rs6045676	6
chr20	17436472	rs6044834	5
chr20	17368541	rs6044777	6
chr20	16536413	rs6044112	6
chr13	113772706	rs6041	5
chr20	11157010	rs6040399	6
chr11	116654434	rs603446	6
chr10	102075478	rs603424	5
chr20	35809782	rs6031882	3a
chr20	42573821	rs6031252	3a
chr20	39672617	rs6029526	6
chr20	38820804	rs6028945	5
chr20	37845476	rs6028335	6
chr20	59268709	rs6027755	5
chr20	58177614	rs6026990	5
chr20	57469072	rs6026584	2b
chr20	56070504	rs6025590	4
chr20	55109303	rs6024938	6
chr20	52447302	rs6022786	4
chr20	42946965	rs6017317	6
chr20	42854133	rs6017291	6
chr20	57751116	rs6015450	4
chr20	50702632	rs6013382	5
chr20	50637659	rs6013355	5
chr20	62297801	rs6011002	5
chr20	62309838	rs6010620	5
chr22	50086372	rs6009824	4
chr22	27852182	rs6005451	5
chr11	65260645	rs600231	2b
chr1	109822165	rs599839	6

chr11	68192345	rs599083	5
chrX	95079869	rs5990417	6
chrX	154014106	rs5987027	4
chrX	111646463	rs5982533	6
chr2	31474241	rs597800	5
chr18	6939946	rs597503	6
chrX	28233052	rs5971305	6
chrX	17698396	rs5955543	5
chrX	141360570	rs5954596	6
chrX	51229682	rs5945572	6
chrX	152899921	rs5945326	5
chr6	93847883	rs594442	6
chrX	89288226	rs5941436	6
chr11	65513106	rs593982	5
chrX	75347433	rs5937496	3a
chrX	68892915	rs5936487	6
chrX	9751473	rs5934683	5
chr12	115116351	rs59336	5
chrX	23383443	rs5925760	6
chrX	22892076	rs5925696	6
chr6	139840692	rs592423	5
chrX	118567701	rs5910578	6
chr22	26697194	rs591044	6
chr6	139844428	rs590856	6
chrX	139166682	rs5907577	6
chrX	146322622	rs5904726	6
chr11	64525215	rs589691	1f
chr2	139399353	rs58938945	5
chr1	201630954	rs586688	5
chr11	117851051	rs58603829	5
chr3	62733365	rs580384	6
chr9	136154167	rs579459	4
chr11	122030189	rs577948	5
chr22	51017352	rs5770917	1f
chr22	48929568	rs5768709	5
chr22	47532395	rs5766691	5
chr22	45150658	rs5765956	5
chr1	160746075	rs576523	5
chr22	28090206	rs5762311	6
chr22	43500211	rs5759167	2b
chr22	40820150	rs5757949	5
chr22	37467391	rs5756506	5
chr22	21939674	rs5754217	5
chr22	30581721	rs5753037	5
chr22	24992265	rs5751901	1f
chr22	23593050	rs5751614	2b
chr22	43635572	rs5751452	5

chr22	33059664	rs5749482	5
chr22	32880584	rs5749446	6
chr1	207032750	rs574773	4
chr16	50756773	rs5743289	6
chr2	190678544	rs5743030	6
chr12	102799597	rs5742692	6
chr13	34903091	rs571411	6
chr18	57839768	rs571312	6
chr9	139862632	rs57024841	5
chr2	169782879	rs569805	5
chr9	135344406	rs569434	6
chr9	22029546	rs564398	6
chr1	34173819	rs564148	6
chr11	126028716	rs563519	2b
chr10	34817987	rs563507	5
chr19	39849788	rs562638	5
chr11	114386829	rs561722	6
chr11	85800278	rs561655	5
chr13	113760033	rs561241	4
chr11	64150369	rs559928	1f
chr12	56070667	rs55874825	2c
chr19	7909882	rs558718	5
chr19	11188152	rs55791371	5
chr6	44594158	rs556621	6
chr4	122607177	rs55645543	5
chr13	113756539	rs555212	5
chr2	169791437	rs552976	5
chr18	55810732	rs549476	4
chr12	63180815	rs549433	5
chr1	5913620	rs548726	5
chr6	106568033	rs548234	5
chr11	125461708	rs548181	4
chr13	75776267	rs548097	5
chr4	74956371	rs546829	6
chr1	66762465	rs546784	6
chr11	30508107	rs545610	6
chr11	124537994	rs544368	6
chr15	35068308	rs543686	6
chr22	39687483	rs54211	5
chr6	31916950	rs541862	4
chr17	7184480	rs5415	1f
chr13	76326281	rs539514	6
chr11	64129721	rs538147	1d
chr11	85787823	rs536841	5
chr11	79065100	rs530965	2b
chr16	11373319	rs529866	4
chr2	45154907	rs528301	6

chr11	77154638	rs527589	6
chr1	47918820	rs527430	5
chr1	58757914	rs527409	5
chr11	59633492	rs526934	6
chr5	134356704	rs526896	5
chr10	13103284	rs525455	6
chr1	183176429	rs525410	1f
chr11	70506205	rs525304	5
chr9	22019128	rs523096	3a
chr12	28228566	rs522958	6
chr19	45376283	rs519113	1f
chr8	102944862	rs517811	6
chr1	177855516	rs516636	5
chr8	41519461	rs515071	5
chr9	136142202	rs514659	6
chr3	182870435	rs514636	5
chr1	234858596	rs514230	3a
chr6	33541718	rs513349	4
chr11	117075565	rs508487	5
chr9	136149398	rs507666	5
chr17	7118321	rs507506	4
chr9	136149228	rs505922	5
chr11	64357071	rs505802	5
chr11	243267	rs505404	6
chr11	64464084	rs504915	5
chrX	66942624	rs5031002	5
chr6	138195722	rs5029939	5
chr11	34605434	rs5028798	5
chr11	51414217	rs5017948	6
chr10	8093033	rs501764	2b
chr11	38278949	rs5004866	5
chr2	166704293	rs5001812	6
chr6	13332466	rs499818	1f
chr1	152493153	rs499697	5
chr16	15082864	rs4985167	5
chr16	15129458	rs4985155	4
chr11	69234493	rs4980785	5
chr9	117567012	rs4979462	2b
chr9	22098573	rs4977574	2c
chr5	1877279	rs4975709	5
chr5	1315659	rs4975616	5
chr2	176734597	rs4972489	6
chr2	20903014	rs4971516	6
chr1	150950061	rs4970988	1f
chr16	23765773	rs4968031	5
chr15	100759613	rs4965598	6
chr15	98518791	rs4965121	6

chr12	104192823	rs4964805	1f
chr12	106949986	rs4964469	6
chr11	61815802	rs4963452	2a
chr11	61494326	rs4963243	5
chr11	589563	rs4963128	5
chr21	44779679	rs496300	3a
chr10	126696871	rs4962416	5
chr9	136323753	rs4962153	1f
chr8	142104943	rs4961252	5
chr6	3359336	rs4959235	5
chr6	31099576	rs4959053	4
chr5	150450235	rs4958881	4
chr5	149623364	rs4958456	5
chr9	136154866	rs495828	5
chr5	583441	rs4957048	5
chr3	55035873	rs4955826	1f
chr3	170494408	rs4955755	5
chr2	42276920	rs4952590	5
chr1	201952573	rs4950806	4
chr1	30432218	rs4949526	5
chr1	31315592	rs4949316	4
chr10	63805616	rs4948496	4
chr10	62185493	rs4948418	5
chr6	31058177	rs4947296	6
chr6	110090048	rs4947019	4
chr6	86117128	rs494562	6
chr11	118574674	rs494459	6
chr1	112192121	rs494453	5
chr11	75909618	rs4944092	5
chr13	44217063	rs4942242	6
chr18	50192395	rs4940203	6
chr18	47167213	rs4939883	5
chr18	46453462	rs4939827	5
chr11	60793650	rs4939490	3a
chr11	119099905	rs4938642	5
chr11	111275132	rs4938534	5
chr11	110913239	rs4938174	6
chr11	128492738	rs4937362	5
chr11	128189100	rs4937314	2b
chr11	126281896	rs4937126	2b
chr11	126296588	rs4935969	5
chr15	89902031	rs4932217	4
chr15	89245238	rs4932194	5
chr6	87466491	rs493187	5
chr3	195751629	rs4927850	6
chr1	247214877	rs4925540	6
chr20	60921043	rs4925386	1f

chr20	60381798	rs4925295	5
chr20	59947043	rs4925189	5
chr15	40339493	rs4924410	4
chr15	36293604	rs4923705	5
chr8	18272437	rs4921914	6
chr8	18782046	rs4921617	5
chr7	50305862	rs4917014	5
chr15	53946592	rs491567	6
chr1	196886769	rs4915559	6
chr1	108366015	rs4915077	6
chr2	141362057	rs491391	5
chr11	5306508	rs4910742	5
chr8	139255917	rs4909764	3a
chr7	158136985	rs4909189	5
chr2	97228038	rs4907240	6
chr14	102454932	rs4906172	5
chr11	116298366	rs490592	4
chr14	97345805	rs4905558	6
chr14	92972161	rs4904947	4
chr14	69254190	rs4902647	4
chr14	69210198	rs4902642	5
chr14	75701220	rs4899554	4
chr14	69278203	rs4899260	1f
chr18	57882786	rs489693	5
chr6	135426572	rs4895441	1f
chr3	171995604	rs4894535	6
chr18	74101940	rs4891159	4
chr16	79431852	rs4888966	5
chr16	77783579	rs4888671	2b
chr15	75755466	rs4886707	5
chr3	118121334	rs488628	5
chr13	61113738	rs4886238	6
chr8	5461752	rs4875598	4
chr8	4284691	rs4875102	6
chr8	126537569	rs4871611	4
chr8	123706154	rs4871297	5
chr7	57699150	rs4870684	6
chr6	151901408	rs4870044	5
chr6	151907747	rs4869742	6
chr5	92567538	rs4869419	6
chr5	50928809	rs4865673	3a
chr4	41284710	rs4861096	5
chr3	159745862	rs485499	4
chr2	74202577	rs4852324	6
chr2	121245121	rs4849887	5
chr1	110366082	rs484959	4
chr2	111599705	rs4849121	4

chr1	230307181	rs4846922	2b
chr1	230295690	rs4846914	5
chr1	219750716	rs4846567	5
chr1	11788563	rs4846033	5
chr1	152492558	rs4845783	5
chr1	151801679	rs4845604	4
chr1	153479997	rs4845552	4
chrX	68805317	rs4844096	6
chr16	87991050	rs4843747	5
chr8	10462805	rs4841398	5
chr3	143902026	rs4839680	6
chr1	111033808	rs4839431	3a
chr10	49699956	rs4838605	5
chr10	50561506	rs4838508	6
chr9	128831096	rs4838320	5
chr9	123056636	rs4837752	5
chr5	130017286	rs4836519	6
chr5	124332102	rs4836133	4
chr12	99334528	rs483610	3a
chr4	146821409	rs4835265	5
chr8	15532584	rs4831760	6
chr22	45011760	rs4823246	5
chr22	29115065	rs4822983	6
chr22	27011419	rs4822752	4
chr22	23765264	rs4822410	5
chr22	37258502	rs4821544	1a
chr22	36616444	rs4821469	5
chr22	33056340	rs4821083	2b
chr22	29161006	rs4820792	6
chr22	24990212	rs4820599	5
chr22	41431341	rs4820425	6
chr22	37209295	rs4820255	5
chr21	37485061	rs4817775	4
chr20	5840538	rs4815868	4
chr20	3827308	rs4815617	1b
chr20	6699594	rs4813802	4
chr20	44763283	rs4813003	1f
chr20	42989266	rs4812829	6
chr20	57587770	rs4812048	1d
chr20	56796783	rs4811971	5
chr20	36469693	rs4811196	6
chr20	44747946	rs4810485	1f
chr20	44545047	rs4810479	6
chr20	62349585	rs4809330	6
chr20	62318219	rs4809324	4
chr19	2410483	rs4806846	6
chr19	35555189	rs4806073	4

chr19	34168272	rs4805924	4
chr19	33906122	rs4805885	5
chr19	42066278	rs4803480	1f
chr19	46849805	rs4802307	4
chr18	25773015	rs4800843	6
chr18	20727610	rs4800452	6
chr18	20724327	rs4800148	6
chr18	34928638	rs4799915	5
chr18	77573547	rs4799088	5
chr18	75912921	rs4798896	5
chr11	65551956	rs479844	5
chr11	64107476	rs479777	1f
chr18	13708573	rs4796995	6
chr17	34819190	rs4796217	5
chr17	25541277	rs4795519	5
chr17	38156711	rs4794822	1f
chr17	38089343	rs4794820	6
chr17	54850328	rs4794665	3a
chr1	172199572	rs479336	6
chr17	16845166	rs4792800	1f
chr17	13684916	rs4792394	6
chr17	11803460	rs4792192	4
chr17	2068931	rs4790881	6
chr17	2262702	rs4790333	4
chr17	76881702	rs4789939	5
chr17	74997896	rs4789400	5
chr16	71634810	rs4788815	3a
chr11	134139779	rs478881	5
chr16	28539847	rs4788084	1a
chr16	29885446	rs4787483	1f
chr16	6148029	rs4786816	5
chr11	64478062	rs478607	4
chr16	90066935	rs4785763	1f
chr16	50103733	rs4785204	6
chr16	52599187	rs4784227	3a
chr16	82662267	rs4783244	5
chr16	82594382	rs4783227	5
chr2	25301754	rs478222	2b
chr16	10975310	rs4781011	2b
chr16	11347857	rs4780355	1b
chr11	64048911	rs477895	5
chr15	93877424	rs4777845	3a
chr15	73082365	rs4777542	6
chr15	68080885	rs4776970	6
chr10	131426021	rs477692	1f
chr5	134467699	rs477687	5
chr15	70006872	rs4776472	3a

chr15	46639807	rs4775302	6
chr6	32569690	rs477515	6
chr15	58635582	rs4775031	5
chr13	111818831	rs4773330	5
chr13	110960711	rs4773144	2b
chr13	98053791	rs4771996	5
chr13	28020179	rs4771122	6
chr13	26037959	rs4770837	1f
chr12	114637847	rs4767234	6
chr12	111904370	rs4766578	6
chr12	2349583	rs4765905	6
chr12	125320849	rs4765623	5
chr12	124460166	rs4765127	5
chr12	14115481	rs4764043	6
chr12	14064460	rs4764039	5
chr12	9910163	rs4763879	1f
chr12	19866128	rs4762767	5
chr12	71634793	rs4760790	5
chr12	48173351	rs4760636	4
chr12	123796237	rs4759375	6
chr11	16403510	rs4756846	6
chr10	122720973	rs4752485	6
chr10	116139028	rs4751674	5
chr10	131992244	rs4751178	5
chr10	132547260	rs4750829	5
chr10	8633860	rs4749791	5
chr10	26162765	rs4749080	6
chr10	6606540	rs4748153	5
chr9	73784263	rs4745062	5
chr9	71434706	rs4744712	6
chr9	100740123	rs4743150	6
chr9	109632352	rs4743034	5
chr9	98256234	rs473902	5
chr8	41630404	rs4737009	4
chr7	134250321	rs4732038	5
chr7	123411222	rs4731120	5
chr7	107484436	rs4730276	6
chr7	107207694	rs4730250	6
chr7	96117917	rs4729260	6
chr11	125905257	rs472926	5
chr7	128573966	rs4728142	1f
chr7	150637862	rs4725982	5
chr7	27231761	rs4722672	4
chr7	1912221	rs4721135	5
chr6	45885060	rs4714888	6
chr6	35402784	rs4713858	6
chr6	33817928	rs4713693	6

chr9	15289577	rs471364	1f
chr6	29434413	rs4713226	5
chr6	10969140	rs4713103	5
chr6	25772046	rs4712972	6
chr6	20657864	rs4712524	5
chr6	20657563	rs4712523	4
chr6	33659045	rs4711336	1f
chr6	11074346	rs4711171	6
chr6	68181696	rs4710654	6
chr11	64584230	rs470763	6
chr5	76518441	rs4704397	6
chr5	80654965	rs4703516	5
chr5	99991647	rs4702982	6
chr5	10671039	rs4702718	1f
chr5	7649859	rs4702484	5
chr5	26139137	rs4701523	6
chr5	21779153	rs4701252	6
chr4	15737347	rs4698412	6
chr5	112242967	rs469727	5
chr5	178663407	rs469568	5
chr4	6270055	rs4689388	4
chr3	119227246	rs4688011	3a
chr3	53282302	rs4687718	5
chr3	184514612	rs4686760	4
chr3	883850	rs4684585	6
chr3	140247176	rs4683505	6
chr3	29275645	rs4680719	6
chr3	159698944	rs4680534	6
chr3	33020034	rs4678680	5
chr2	241563738	rs4676410	2b
chr2	241579107	rs4676406	5
chr2	109635256	rs4676049	3a
chr2	207840331	rs4675644	6
chr2	206086170	rs4675502	6
chr2	38044343	rs4670779	6
chr2	37940541	rs4670766	3a
chr2	172127919	rs4667682	6
chr2	27840639	rs4666002	6
chr2	23898316	rs4665630	6
chr2	160917496	rs4664308	5
chr2	235601063	rs4663476	4
chr2	129478841	rs4662834	5
chr1	41839821	rs4660531	5
chr1	41239510	rs4660456	4
chr1	40028179	rs4660293	4
chr1	165831648	rs4657482	4
chr1	162210609	rs4657178	3a

chr1	161272440	rs4657015	4
chr1	160856963	rs4656958	5
chr1	160830267	rs4656940	6
chr1	159326879	rs4656784	6
chr1	165687204	rs4656461	4
chr1	20227722	rs4654925	6
chr1	21786067	rs4654748	6
chr17	46988596	rs46522	1f
chr1	183837031	rs4651156	6
chr1	79238014	rs4650608	6
chr1	24519919	rs4649203	5
chr1	2709163	rs4648356	4
chr6	34845448	rs4646949	6
chr2	315214	rs4643574	5
chr17	21284222	rs4640244	5
chr11	118573518	rs4639966	5
chr11	18285773	rs4638289	4
chr9	21747802	rs4636294	6
chr5	1308551	rs4635969	6
chr2	40662545	rs4629203	4
chr3	32347823	rs4627791	5
chr5	141681787	rs4624820	6
chr3	36862979	rs4624519	6
chr12	102321934	rs4622329	4
chr5	113030163	rs4621553	6
chr4	38465470	rs4615179	5
chr7	120903814	rs4609139	6
chr7	44235667	rs4607517	5
chr3	64711903	rs4607103	6
chr17	49244746	rs4605213	2b
chr1	25044110	rs4601530	6
chr4	61966272	rs4599440	6
chr7	107503440	rs4598195	6
chr3	7982826	rs4591494	6
chr10	63467552	rs4590817	6
chr8	96106036	rs4590408	5
chr21	31177510	rs458685	5
chr10	92632898	rs4586057	5
chr16	80497600	rs4581712	5
chr5	75920971	rs457717	6
chr9	4834393	rs457287	4
chr11	21058730	rs4569005	6
chr5	55811091	rs456867	5
chr17	16848749	rs4561508	1f
chr11	24678818	rs4561213	6
chrX	121967358	rs4559365	5
chr21	31146168	rs455804	6

chr3	73540617	rs4557101	5
chr2	54718180	rs4557020	5
chr11	103530365	rs4554859	4
chr5	83173592	rs4552569	6
chr4	146794620	rs4547811	6
chr2	70736218	rs454305	1f
chr21	42746080	rs45430	5
chr1	154418878	rs4537545	4
chr8	108789815	rs4534106	5
chr15	100786270	rs4533267	5
chr6	32581888	rs4530903	6
chr11	127081590	rs4529888	6
chr8	134196848	rs4527850	5
chr16	19960319	rs4527026	4
chr14	23865884	rs452036	5
chr6	104432164	rs4520040	5
chr6	160137686	rs4516970	4
chr6	43925525	rs4513773	6
chr2	115049817	rs4513299	6
chr10	102501570	rs4509693	5
chr4	155481288	rs4508864	1f
chr4	123132491	rs4505848	6
chr18	56084053	rs4503880	5
chr3	59921019	rs4502542	4
chr16	15140210	rs4500751	1f
chr3	103438873	rs4493441	6
chr2	217894755	rs4491709	6
chr11	90956213	rs4491175	5
chr3	189356260	rs4488809	5
chr7	21938239	rs4487645	2b
chr15	68198910	rs448720	4
chr16	76566156	rs4485401	1f
chr5	26723782	rs4479806	6
chr4	9946655	rs4475146	5
chr12	88953958	rs4474514	6
chr4	162250931	rs4470583	6
chr20	59928236	rs4468878	5
chr11	132010116	rs4468361	4
chr14	77684669	rs4467006	5
chr14	65475539	rs4466998	6
chr5	82985738	rs4466137	6
chr4	40973526	rs4466078	5
chr10	59189177	rs4462262	6
chr5	109496959	rs4460176	5
chr5	76424948	rs4457053	6
chr8	128323180	rs445114	5
chr3	13736999	rs4450798	5

chr3	111423128	rs4450776	6
chr7	92408369	rs445	4
chr1	159357683	rs4446959	6
chr14	54410918	rs4444235	5
chr2	16715407	rs4441471	6
chr2	167177358	rs4438497	6
chr6	11310418	rs4437462	3a
chr1	153774275	rs4434872	5
chr17	36098039	rs4430796	4
chr1	66161460	rs4420065	6
chr6	31391400	rs4418214	5
chr21	42698906	rs441810	3a
chr5	44662514	rs4415084	6
chr8	128815028	rs4410871	2b
chr7	17284576	rs4410790	6
chr7	94053999	rs441051	5
chr11	95311421	rs4409785	4
chr10	101284236	rs4409764	3a
chr4	66470812	rs4404602	6
chr18	6941107	rs4398173	3a
chr11	133566984	rs4397868	6
chr8	90342185	rs4397449	5
chr19	45414450	rs439401	1b
chr4	80946474	rs4389526	6
chr1	66279124	rs4384209	6
chr1	209334216	rs4382726	6
chr6	9030335	rs438259	6
chr3	32329496	rs4380451	5
chr15	79111092	rs4380028	6
chr10	64963448	rs4379723	6
chr3	42303073	rs4377469	5
chr3	2654690	rs4370013	6
chr18	20735407	rs4369779	5
chr12	21368721	rs4363657	6
chr10	129274502	rs4363506	6
chr2	76240360	rs436000	6
chr11	17160147	rs4356203	6
chr11	18323516	rs4353250	5
chr2	37750979	rs4352210	5
chr17	61569731	rs4351	4
chr6	31365786	rs4349859	4
chr16	3124919	rs4349147	4
chr3	167837747	rs4345115	5
chr15	49735296	rs4338740	6
chr5	9474945	rs433755	6
chr16	20680205	rs433598	6
chr4	80949828	rs4333130	6

chr1	115875644	rs4332358	5
chr7	1950808	rs4332037	5
chr17	61563457	rs4329	5
chr3	128316434	rs4328821	5
chr1	159462380	rs4325129	5
chr6	28776116	rs4324798	5
chr14	88295599	rs4322600	6
chr13	98960761	rs4318070	5
chr6	29973924	rs4313034	1f
chr5	53300661	rs4311394	5
chr3	121793186	rs4308217	6
chr5	25967702	rs4307059	5
chr2	241495012	rs4305276	5
chr7	36191698	rs4302748	5
chr3	150042617	rs4301033	4
chr7	19106538	rs430	5
chr12	122276254	rs4298948	4
chr7	103625876	rs4298437	5
chr6	31930461	rs429608	5
chr7	135293127	rs4294134	6
chr21	47445790	rs4293630	5
chr16	20364587	rs4293393	6
chr14	54857285	rs4293296	5
chr11	82821381	rs4293143	6
chr5	168256239	rs4282339	4
chr6	126964509	rs4273712	5
chr8	107909008	rs4269515	6
chr6	156197501	rs4269383	5
chr1	196760416	rs426736	6
chr16	19028548	rs4265793	6
chr9	117566439	rs4263839	2b
chr5	152288452	rs4262150	6
chr6	24863074	rs4256430	6
chr3	18767403	rs4256159	4
chr2	69198387	rs4254535	6
chr4	187157457	rs4253252	6
chr1	2069171	rs425277	4
chr21	43707100	rs425215	5
chr14	54876183	rs4251631	6
chr12	44153825	rs4251424	5
chr19	47208480	rs425105	5
chr16	85946449	rs424971	5
chr1	163956502	rs424950	6
chr9	24092071	rs4246856	6
chr1	39380384	rs4246511	6
chr2	44074430	rs4245791	6
chr4	187152326	rs4241816	6

chr11	98466267	rs4237591	6
chr7	116162728	rs4236601	5
chr4	156998540	rs4234898	6
chr4	735149	rs4234853	5
chr4	7219932	rs4234798	4
chr1	159753182	rs4233356	6
chr5	176517325	rs422421	2b
chr16	23634025	rs420259	6
chr7	117991894	rs41997	6
chr3	169100885	rs419076	6
chr6	167406632	rs415890	1f
chr17	44859143	rs415430	6
chr9	107588776	rs4149311	2b
chr9	107589133	rs4149310	5
chr9	107647219	rs4149268	6
chr6	43272187	rs4149178	5
chr13	95898206	rs4148441	5
chr2	234673308	rs4148325	5
chr17	66875293	rs4148008	6
chr2	23238942	rs41458646	6
chr1	167718498	rs4145462	5
chr18	26676671	rs4145170	5
chr15	62312839	rs4143844	5
chr5	131862976	rs4143832	4
chr10	68640950	rs4142041	5
chr20	14747470	rs4141463	6
chr1	61815798	rs41350144	5
chr9	92036426	rs4132699	5
chr9	130107963	rs4130590	1f
chr18	40678234	rs4130047	6
chr17	76403983	rs4129767	5
chr1	154426263	rs4129267	2b
chrX	990179	rs4129148	6
chr1	159405958	rs4128725	6
chr22	22307518	rs412050	4
chr1	107577831	rs4118325	6
chr5	156608999	rs411174	6
chr19	41358623	rs4105144	3a
chr9	4744742	rs409801	6
chr16	82377780	rs4087296	3a
chr1	152550017	rs4085613	5
chr11	69596522	rs4084127	6
chr13	72768708	rs4083578	4
chr16	86368525	rs4083242	5
chr2	68359896	rs4078978	5
chr5	176784511	rs4075958	1d
chr2	18243533	rs4075511	6

chr5	52193124	rs4074793	1f
chr19	8644030	rs4072910	5
chr8	142736530	rs4072286	1f
chr14	59366314	rs405460	4
chr6	32184344	rs404860	5
chr8	90823686	rs40457	5
chr2	40396077	rs404005	5
chr2	17775031	rs4038131	5
chr2	12037491	rs4027132	6
chr5	1320721	rs402710	5
chr3	1623392	rs402675	6
chr6	160789295	rs402219	4
chr5	1322086	rs401681	5
chr11	65506821	rs4014195	6
chr17	39250638	rs4006360	6
chr6	32975013	rs399604	6
chr5	147845814	rs3995090	5
chr5	66152257	rs39861	5
chr17	19804246	rs397969	1f
chr20	1205885	rs397020	5
chr12	51727595	rs3951439	5
chr7	25133848	rs39453	4
chr11	48115088	rs3942852	1f
chr1	24431955	rs3934861	4
chr1	1005805	rs3934834	5
chr1	104598940	rs3934285	6
chr17	43719142	rs393152	1f
chr4	149079496	rs3931397	6
chr1	75235316	rs3931020	6
chr11	30760334	rs3925584	6
chr16	31347747	rs3925075	6
chr6	38440969	rs3923809	6
chr3	38624252	rs3922844	5
chr3	42909159	rs3919627	5
chr6	32685549	rs3916765	6
chr16	85944438	rs391525	4
chr7	103527368	rs3914132	6
chr3	171653902	rs3913363	4
chr17	2216257	rs391300	5
chr1	170569316	rs3903239	6
chr6	31940896	rs389884	4
chr1	4668669	rs3896439	5
chr6	29935249	rs3893464	4
chr3	194758009	rs3892715	5
chr19	33181483	rs3892630	6
chr2	66756975	rs3891585	5
chr12	106950694	rs3891355	6

chr1	2553623	rs3890745	6
chr9	107647654	rs3890182	5
chr17	64779429	rs3889237	5
chr16	79652540	rs386965	5
chr6	31184195	rs3869109	1b
chr16	82650716	rs3865188	6
chr19	54792760	rs386000	5
chr17	38128647	rs3859192	3a
chr9	4763175	rs385893	4
chr10	70011837	rs3858145	2b
chr6	31499602	rs3853601	5
chr5	166318189	rs3853240	6
chr6	111848190	rs3851228	6
chr10	81882369	rs3851050	5
chr14	78534905	rs3850370	5
chr9	138028676	rs3849221	5
chr17	14294020	rs3848445	6
chr12	131525052	rs3847687	5
chr11	2953722	rs3847646	5
chr10	21567564	rs3847375	6
chr5	74655725	rs3846663	6
chr10	119336745	rs384627	5
chr2	65758524	rs3845817	5
chr1	159218265	rs3845624	5
chr2	234180409	rs3828309	4
chr1	50937847	rs3827730	4
chr19	51726612	rs3826656	6
chr15	79235445	rs3825932	1f
chr15	58746829	rs3825776	2b
chr12	114795442	rs3825214	5
chr11	74345549	rs3824999	2b
chr4	6697765	rs3822262	1f
chr2	227773465	rs3820928	1f
chr1	53581669	rs3820201	4
chr6	31322366	rs3819299	4
chr11	16902267	rs381815	6
chr6	32368086	rs3817963	6
chr6	142750515	rs3817928	3a
chr11	47650992	rs3817334	6
chr11	1909005	rs3817198	5
chr17	71335259	rs3816995	5
chr4	25417243	rs3816587	6
chr8	142161063	rs3816063	6
chr7	20047574	rs38152	5
chr7	106938419	rs3815148	6
chr10	115481017	rs3814231	6
chr10	105647094	rs3814219	6

chrX	145748907	rs381365	6
chr16	79749352	rs3813582	5
chr16	79749275	rs3813579	5
chr6	7725759	rs3812163	5
chr3	133484028	rs3811647	5
chr19	50223265	rs3810265	5
chr18	6941661	rs3810046	5
chr15	63333723	rs3809566	5
chr13	110960942	rs3809346	4
chr7	116186240	rs3807989	3a
chr7	150667209	rs3807375	5
chr5	110405674	rs3806932	5
chr1	20142865	rs3806308	2b
chr6	32373697	rs3806156	1f
chr4	100056997	rs3805322	5
chr3	3130644	rs3804795	5
chr21	41993706	rs3804024	6
chr19	2160528	rs3803915	5
chr1	196701050	rs380390	6
chr12	113173493	rs3803064	6
chr11	111171708	rs3802842	1f
chr7	120974764	rs3801387	4
chr7	138411424	rs3800569	5
chr6	1918261	rs3800131	2b
chr6	44837355	rs3799977	1b
chr6	11040422	rs3798722	2b
chr6	11008621	rs3798713	5
chr4	82009706	rs3796804	6
chr4	1095280	rs3796619	5
chr3	52913278	rs3796352	2b
chr1	204112983	rs3795578	5
chr12	20860092	rs3794271	6
chr11	35329614	rs3794087	6
chr10	124219274	rs3793917	5
chr5	32768633	rs3792752	4
chr3	122839875	rs3792366	5
chr2	218729864	rs3791950	2b
chr2	56096891	rs3791679	2b
chr2	56111308	rs3791675	6
chr1	200007431	rs3790844	5
chr1	156456300	rs3790455	5
chr20	10639987	rs3790160	5
chr22	19889824	rs3788317	1f
chr21	43841327	rs3788013	3a
chr19	33893007	rs3786897	5
chr19	30990699	rs3786800	6
chr16	83757327	rs3784962	6

chr14	68749926	rs3784099	3a
chr14	81594379	rs3783938	4
chr14	55348117	rs3783637	2b
chr12	88953560	rs3782181	5
chr11	65336818	rs3782089	5
chr11	72373495	rs3781913	1f
chr11	76823483	rs3781684	5
chr10	96070374	rs3781264	4
chr21	40469519	rs378108	5
chr9	136835342	rs3780792	5
chr7	150669975	rs3778872	5
chr5	95234790	rs3777200	5
chr4	9995181	rs3775948	6
chr4	103511113	rs3774959	1b
chr3	30710242	rs3773643	6
chr3	121344139	rs3772130	6
chr2	70697666	rs3771514	5
chr2	102953616	rs3771180	2b
chr2	102986221	rs3771166	6
chr2	33471191	rs3769528	6
chr1	175296447	rs3766680	6
chr19	1046519	rs3764650	2a
chr19	1207237	rs3764640	1f
chr19	18747604	rs3764628	2a
chr19	15724202	rs3764563	6
chr17	46123931	rs3764400	6
chr6	32376787	rs3763317	6
chr6	32376470	rs3763313	6
chr1	67597118	rs3762318	6
chr1	157669277	rs3761959	6
chr1	230388988	rs3761944	5
chr9	123690238	rs3761847	4
chr20	3776174	rs3761218	1f
chr19	5839745	rs3760776	2b
chr19	5841355	rs3760775	5
chr17	29247714	rs3760318	4
chr11	94132138	rs3758785	5
chr9	75764564	rs3758354	1b
chr6	16127406	rs3757354	5
chr6	151914112	rs3757318	4
chr6	90957462	rs3757247	6
chr6	70074231	rs3757057	6
chr4	187185384	rs3756008	6
chr2	15729819	rs3755132	6
chr1	185014001	rs3753573	4
chr1	196620916	rs3753394	2b
chr1	2069680	rs3753242	4

chr10	123332576	rs3750817	4
chr9	71943001	rs3750552	4
chr6	32862606	rs3749982	2a
chr6	142767632	rs3748069	6
chr6	80257280	rs3747767	6
chr19	6690770	rs3745567	5
chr19	50926741	rs3745516	4
chr19	7734510	rs3745367	5
chr17	73888671	rs3744028	5
chr5	34951149	rs37439	6
chr15	79274045	rs3743200	5
chr15	85430968	rs3743162	5
chr12	6938871	rs3741920	4
chr11	116657560	rs3741298	5
chr11	2169773	rs3741208	5
chr10	126294166	rs3740540	5
chr1	17331675	rs3738814	2b
chr1	247348188	rs3738443	6
chr13	50306220	rs3736830	2b
chr7	94047265	rs3736638	5
chr2	27995780	rs3736594	1f
chr19	41310570	rs3733829	1f
chr9	21986846	rs3731211	4
chr3	12626515	rs3729931	1f
chr16	67225500	rs3729639	1f
chr11	5518155	rs372091	5
chr16	84578240	rs371915	5
chr16	58567237	rs37062	6
chr16	58566303	rs37060	6
chr7	21742973	rs368331	4
chr5	108948936	rs367615	5
chr22	30337585	rs36600	1f
chr7	101809850	rs365836	5
chr14	71352647	rs36563	5
chr21	31050816	rs363512	6
chr7	142104570	rs361433	6
chr5	40237373	rs36019094	6
chr2	219829205	rs359980	4
chr3	898660	rs35964523	6
chr5	173279841	rs359457	6
chr10	17388142	rs359312	5
chr3	55313399	rs358806	6
chr4	21476989	rs358592	6
chr15	58680642	rs35853021	5
chr8	94916102	rs35820208	6
chr18	46579969	rs357894	5
chr12	102875568	rs35767	3a

chr12	102912557	rs35747	6
chr1	8021972	rs35675666	4
chr4	90641339	rs356220	6
chr15	24741290	rs35600665	6
chr3	62112140	rs35593266	5
chr5	33955325	rs35390	5
chr2	228092034	rs35212277	6
chr11	70845203	rs35198051	5
chr5	19440167	rs349475	6
chr16	5003073	rs34924084	6
chr4	53412128	rs346923	4
chr6	80564835	rs346291	6
chr3	145173787	rs345013	6
chr2	179104297	rs34479159	6
chr3	156555983	rs344081	4
chr7	106372902	rs342296	5
chr7	106372218	rs342293	5
chr7	106359215	rs342275	4
chr1	214159255	rs340874	5
chr1	214118089	rs340849	5
chr13	36531547	rs34065801	5
chr4	87958394	rs340630	6
chr15	60878029	rs340005	4
chr15	60883280	rs339969	4
chr6	117210051	rs339331	2b
chr1	220914919	rs337161	5
chr19	30943676	rs33436	5
chr1	110439479	rs333960	5
chr17	4393497	rs333119	5
chr20	56029603	rs328506	5
chr5	7816644	rs326155	2b
chr8	19819438	rs326	1f
chr5	149213455	rs32576	5
chr8	19819327	rs325	5
chr6	41905274	rs3218097	5
chr12	4405388	rs3217901	4
chr12	4388270	rs3217810	5
chr13	113793848	rs3211770	2b
chr3	47927483	rs319690	6
chr5	140372221	rs31872	5
chr1	178142207	rs316274	2b
chr1	84792972	rs315553	6
chr7	4474131	rs314590	6
chr7	100453207	rs314370	5
chr6	105400836	rs314280	4
chr6	105407661	rs314277	6
chr6	105407998	rs314276	6

chr6	105417977	rs314268	5
chr17	7091649	rs314253	5
chr11	46743246	rs3136441	5
chr6	32413050	rs3135388	1f
chr6	32401216	rs3135338	1f
chr6	31312325	rs3134792	6
chr6	30537605	rs3132613	6
chr6	31475485	rs3132468	1f
chr9	137440211	rs3132306	5
chr6	31721032	rs3131379	1f
chr6	32172992	rs3131296	1f
chr6	31143581	rs3131018	1f
chr6	31106267	rs3130573	1f
chr6	31097300	rs3130559	5
chr6	31136452	rs3130501	1f
chr6	32223257	rs3130320	1b
chr6	32338694	rs3129943	5
chr6	32336186	rs3129934	6
chr6	32305978	rs3129900	6
chr6	32413544	rs3129889	1f
chr6	32409529	rs3129882	1f
chr6	32401078	rs3129860	6
chr6	32590924	rs3129763	1f
chr6	32663630	rs3129720	6
chr6	29670260	rs3129055	1f
chr1	152037514	rs3124314	6
chr10	51524970	rs3123078	4
chr13	63638328	rs3119939	5
chr5	134372684	rs31198	5
chr6	31620519	rs3117582	2b
chr6	33069892	rs3117242	6
chr6	32071016	rs3117181	6
chr6	32358269	rs3117099	5
chr6	32358512	rs3117098	6
chr6	33086248	rs3117035	6
chr13	51111354	rs3116602	5
chr6	32218842	rs3115573	6
chr4	89064580	rs3114018	5
chr4	87832600	rs3113494	4
chr16	7836296	rs3112740	6
chr17	27917770	rs3110496	1f
chr8	60130295	rs3110127	6
chr4	95153348	rs3106136	6
chr6	32681675	rs3104402	6
chr1	244533213	rs3101457	2b
chr6	31448975	rs3099844	6
chr16	83764203	rs3096277	5

chr9	90209136	rs3095748	6
chr6	31221667	rs3095254	6
chr6	31142244	rs3094188	6
chr19	10397402	rs3093030	4
chr6	167532792	rs3093024	6
chr6	167534289	rs3093023	1f
chr5	131402737	rs3091338	6
chr17	32593664	rs3091315	5
chr1	25674784	rs3091242	6
chr3	12116619	rs308971	6
chr2	204738918	rs3087243	3a
chr19	47661492	rs307896	3a
chr16	85975658	rs305061	5
chrX	43607032	rs3027409	5
chr17	8136091	rs3027234	6
chr1	159147451	rs3026968	4
chr9	136478354	rs3025343	5
chr1	206939903	rs3024505	2b
chr1	206943967	rs3024493	2b
chr18	60082092	rs3018362	4
chr1	44319372	rs3011225	2b
chr2	112495	rs300774	6
chr1	18795254	rs3007729	4
chr10	30177887	rs3006564	6
chr1	17591678	rs3003429	4
chr1	219727778	rs3001032	6
chr14	105729791	rs3000073	5
chr10	1630820	rs2999399	5
chr10	30519831	rs2995271	6
chr19	34309531	rs29941	5
chr10	26734586	rs2992257	4
chr1	197020657	rs2990510	6
chr7	40954480	rs29880	6
chr10	30096627	rs2986971	4
chr6	152285686	rs2982694	5
chr10	123352316	rs2981582	5
chr10	123337334	rs2981579	5
chr10	123346115	rs2981575	6
chr8	126481474	rs2980879	5
chr8	30262785	rs2979481	4
chr12	50319085	rs297941	6
chr8	30498858	rs2978263	6
chr12	4606167	rs2970818	6
chr2	47885475	rs2969775	5
chr7	150622161	rs2968864	6
chr19	8469737	rs2967605	1f
chr1	200892136	rs296547	5

chr5	59096922	rs2963826	5
chr5	17215443	rs2962370	5
chr12	57065712	rs2958154	6
chr18	60060734	rs2957128	5
chr8	126507388	rs2954038	5
chr8	126493745	rs2954033	5
chr8	126484525	rs2954026	5
chr8	126482076	rs2954021	6
chr2	208494233	rs2952768	6
chr12	57055290	rs2950390	1f
chr9	10139579	rs294845	6
chr11	55862090	rs2945816	6
chr2	227068079	rs2943634	6
chr17	43714849	rs2942168	6
chr6	152009637	rs2941740	6
chr8	109629902	rs2935776	6
chr1	113216542	rs2932538	2b
chr5	10695525	rs2930047	1f
chr16	81534789	rs2925979	2b
chr11	68556781	rs2924679	5
chr11	10388781	rs2923084	4
chr8	76573710	rs2922763	6
chr10	122591372	rs2919009	6
chr8	6323277	rs2916733	2a
chr20	31950844	rs291671	5
chr1	235720897	rs291353	3a
chr8	69992379	rs2912522	4
chr9	93563535	rs290986	5
chr12	11648417	rs2908835	6
chr12	70690084	rs2904524	5
chr16	11238782	rs2903692	6
chr20	39091486	rs2902940	5
chr9	93555738	rs290258	6
chr1	15792425	rs2901964	4
chr10	122900622	rs2901286	5
chr15	51516054	rs2899472	2b
chr11	18419805	rs2896526	6
chr5	13764418	rs2896103	6
chr8	13260535	rs289585	5
chr14	100133941	rs2895811	5
chr6	31263750	rs2894207	6
chr10	65261183	rs2893923	6
chr7	90915353	rs2888830	5
chr12	1638170	rs2887571	2b
chr1	111417914	rs2885805	4
chr13	90622454	rs2882281	5
chr3	88524174	rs2880961	6

chr1	162014631	rs2880058	5
chr14	27800176	rs2877832	6
chr3	123094450	rs2877716	4
chr13	67696144	rs2875517	6
chr17	38040762	rs2872507	6
chr15	99194895	rs2871865	4
chr4	89869331	rs2869967	5
chr11	34655362	rs286913	4
chr11	34662960	rs286905	3a
chr15	78827630	rs28675338	6
chr4	79513214	rs2867461	4
chr2	622826	rs2867125	5
chr18	9288455	rs2864527	2b
chr1	144992180	rs2863344	5
chr10	96766933	rs2860975	6
chr6	29906690	rs2860580	1f
chr6	32681276	rs2858331	6
chr15	93179174	rs285757	4
chr6	32763513	rs2857151	5
chr6	32670254	rs2856718	6
chr12	11855772	rs2856321	5
chr6	31472719	rs2855812	5
chr1	165403902	rs285480	5
chr17	61977247	rs2854160	6
chr5	1287193	rs2853677	5
chr5	1288546	rs2853676	5
chr7	18980266	rs2853552	5
chr21	44487403	rs2851391	1f
chr19	41224203	rs28493229	2c
chr18	12821592	rs2847281	5
chr10	43755753	rs28461806	5
chr6	31006854	rs2844665	6
chr6	31572955	rs2844479	6
chr6	168388927	rs2843012	5
chr6	160071158	rs2842992	5
chr6	7106315	rs2842895	4
chr6	41650735	rs2842643	5
chr6	32592736	rs28421666	5
chr9	140088629	rs28417902	5
chr9	87930044	rs2841498	5
chr14	105391004	rs2841277	5
chr21	44448717	rs2839627	5
chr21	44436176	rs2839619	5
chr21	46846943	rs2838923	5
chr21	45615022	rs2838519	4
chr22	50350970	rs28372448	5
chr21	40465533	rs2836878	5

chr21	39721763	rs2836326	6
chr6	51058720	rs283566	6
chr21	36238506	rs2834655	4
chr21	35690785	rs2834442	5
chr21	34796885	rs2834215	1f
chr21	33552511	rs2833693	1f
chr21	33381039	rs2833607	6
chr21	33285299	rs2833556	5
chr21	30489299	rs2832191	1f
chr21	28665346	rs2830840	4
chr21	28146188	rs2830487	6
chr21	25080395	rs2829459	6
chr21	23631675	rs2827312	6
chr5	50026464	rs282544	6
chr21	17828290	rs2823819	5
chr21	17483132	rs2823615	5
chr21	16817937	rs2823286	2b
chr1	237911003	rs2819770	6
chr1	237990121	rs2819742	6
chr1	201884951	rs2819348	5
chr1	44005279	rs2819332	5
chr6	118574060	rs281868	6
chr6	121098456	rs2817937	6
chr1	72812439	rs2815752	5
chr6	34618892	rs2814993	5
chr6	34546559	rs2814982	5
chr6	34552796	rs2814944	6
chr9	27536396	rs2814707	5
chr19	49214469	rs281380	5
chr9	34710259	rs2812378	5
chr14	71395603	rs2810114	6
chr1	159692572	rs2808634	6
chr1	117469780	rs2806864	5
chr19	10472932	rs280519	4
chr6	109268049	rs2798641	5
chr1	112437343	rs2798334	5
chr1	7879062	rs2797685	6
chr8	122706290	rs279612	6
chr1	219676041	rs2791553	6
chr10	59985670	rs2790232	5
chr10	59997925	rs2790216	5
chr1	53777630	rs2788032	5
chr1	197325907	rs2786098	5
chr5	127640860	rs27855	5
chr11	35093069	rs2785197	6
chr11	35155731	rs2785173	5
chr10	115781526	rs2782980	5

chr9	114850189	rs2782931	6
chr6	34199091	rs2780226	3a
chr1	158585414	rs2779116	6
chr9	90835725	rs2778031	6
chr9	84213159	rs2777777	4
chr9	81889357	rs2769967	6
chr14	100684191	rs2766692	6
chr13	24504954	rs2765086	6
chr10	3284006	rs2764980	5
chr13	50835714	rs2762051	5
chr5	6879977	rs275437	5
chr12	43929995	rs275380	6
chr1	64127467	rs2749097	1f
chr1	57126918	rs2746347	6
chr1	208128721	rs2745967	5
chr16	1073551	rs2744148	4
chr3	45731450	rs2742417	5
chr10	43612608	rs2742234	1f
chr8	134071832	rs2741200	5
chr22	24295285	rs2739330	6
chr19	11227325	rs2738446	5
chr8	6829084	rs2738113	6
chr8	6822784	rs2738048	6
chr4	90678540	rs2736990	3a
chr6	31843923	rs2736428	5
chr8	11343972	rs2736340	6
chr5	1286515	rs2736100	5
chr19	51364622	rs2735839	5
chr6	31505479	rs2734583	5
chr5	176842473	rs2731672	6
chr12	43160824	rs2731006	5
chr7	158724788	rs2730245	4
chr18	28141687	rs2729409	6
chr5	131665377	rs272889	6
chr4	183137397	rs2726807	5
chr8	81470119	rs272594	6
chr4	88919105	rs2725236	6
chr11	11228373	rs2722769	5
chr13	41110883	rs2721051	4
chr2	116655794	rs272000	6
chr1	234662889	rs271738	5
chr18	74987631	rs2717128	6
chr4	180383311	rs2716816	6
chr6	7240576	rs2714337	6
chr3	128338599	rs2712381	2b
chr8	138918944	rs2705293	6
chr16	86365570	rs2696835	4

chr14	99607479	rs2693676	6
chr7	77964787	rs2691543	6
chr1	238908352	rs2689154	5
chr1	237355984	rs268786	5
chr3	127895225	rs2687729	5
chr16	2249375	rs26868	5
chr12	90013088	rs2681492	4
chr3	121769521	rs2681424	6
chr5	10752314	rs267939	5
chr15	91450440	rs2677744	1f
chr1	150951476	rs267734	1f
chr3	99167106	rs2670321	5
chr12	77009059	rs2669010	6
chr17	61966464	rs2665838	4
chr3	156397748	rs2665390	5
chr12	96479266	rs2660869	6
chr4	102037718	rs2659546	6
chr12	56938382	rs2657888	5
chr8	92559914	rs2657195	6
chr19	17902333	rs265548	2a
chr15	63396866	rs2652834	5
chr15	63422771	rs2652822	6
chr16	28517708	rs26528	1f
chr1	3083711	rs2651899	5
chr3	122368346	rs2650951	6
chr12	121388961	rs2650000	1f
chr11	9109286	rs2647528	5
chr4	106268593	rs2647264	6
chr6	32668335	rs2647046	1b
chr6	32664457	rs2647012	6
chr8	11684462	rs2645424	5
chr1	220973562	rs2642442	5
chr8	97369633	rs2640806	6
chr16	61123146	rs2639889	6
chr19	48401892	rs2637125	5
chr4	106297512	rs2636697	6
chr15	29424754	rs2636061	2b
chr12	104421807	rs2629751	5
chr2	225047743	rs2629046	6
chr15	39148648	rs2624265	6
chr8	3594905	rs2623702	6
chr5	102596719	rs26232	2b
chr6	32741867	rs2621416	5
chr12	32476726	rs261902	6
chr8	11352540	rs2618476	1d
chr5	138949361	rs261532	5
chr20	5037773	rs261360	1b

chr15	58731152	rs261342	4
chr15	58726743	rs261334	5
chr8	34236991	rs2609653	6
chr5	10372617	rs2607292	6
chr1	219644223	rs2605100	5
chr3	108243550	rs2603127	5
chr16	4103870	rs2601828	4
chr6	30025502	rs259919	6
chr2	2590380	rs2598592	3a
chr3	13555835	rs2597513	4
chr6	31366594	rs2596542	6
chr3	22122395	rs2593321	6
chr2	176991778	rs2592394	4
chr3	52622085	rs2590838	6
chr2	120321816	rs2587695	6
chr17	48289069	rs2586502	6
chr2	232797965	rs2580816	4
chr2	23548613	rs2577720	6
chr10	52119296	rs2574975	5
chrX	91402219	rs2573905	5
chr15	66070692	rs2572207	4
chr6	1524569	rs2569872	4
chr19	10798903	rs2569507	4
chr1	68635389	rs2566755	6
chr19	21666209	rs2562456	5
chr2	60762501	rs2556378	1f
chr15	84315883	rs2554380	5
chr3	139606793	rs2554152	4
chr8	30936355	rs2553268	5
chr15	54868667	rs2553218	6
chr8	3189895	rs2551043	5
chr16	68024994	rs255052	1f
chr16	68013470	rs255049	5
chr5	96244548	rs2549794	1f
chr5	171076226	rs254893	5
chr5	53902338	rs2548621	6
chr5	28747322	rs2548003	6
chr5	158759899	rs2546890	4
chr5	176841338	rs2545801	1f
chr5	134443605	rs254560	5
chr2	15926167	rs2544527	5
chr19	54612627	rs254262	1f
chr6	118807846	rs25422	5
chr18	12779946	rs2542151	5
chr5	115568756	rs253959	6
chr4	16018323	rs2531154	5
chr5	11298222	rs2530215	6

chr5	106724587	rs252817	4
chr7	157090295	rs2527866	6
chr14	73081067	rs2526932	5
chr6	31408264	rs2524276	6
chr6	31252395	rs2524054	6
chr6	29899676	rs2524005	6
chr6	29941942	rs2523946	6
chr6	29828659	rs2523822	1f
chr6	29849618	rs2523809	6
chr6	30165272	rs2523722	1f
chr6	31322558	rs2523608	1f
chr6	31327063	rs2523590	6
chr6	29702509	rs2523395	1f
chr6	29705658	rs2523393	5
chr5	131801725	rs2522056	1f
chr11	61711474	rs2521572	5
chr11	2308761	rs2521263	5
chr10	5138035	rs2518049	2b
chr6	29918098	rs2517713	4
chr6	31018406	rs2517532	6
chr8	37977731	rs2517388	1f
chr16	2097157	rs2516739	1f
chr6	31481298	rs2516399	1f
chr9	107594363	rs2515629	5
chr5	172480335	rs251253	5
chr11	98125403	rs2509843	6
chr6	93615449	rs2506933	6
chr10	30335121	rs2505083	5
chr10	43814048	rs2503875	5
chr9	130976556	rs2502731	6
chr10	6946751	rs2501677	6
chr1	22373623	rs2501276	5
chr6	149329266	rs2500535	5
chr1	55512994	rs2495478	5
chr6	40536127	rs2494938	4
chr1	12741239	rs2489260	6
chr1	197631140	rs2488389	6
chr10	31515131	rs2484990	6
chr1	206626827	rs2483058	5
chr1	55504649	rs2479409	5
chr9	126525211	rs2479106	6
chr1	2392647	rs2477686	5
chr16	56990715	rs247617	5
chr15	75044237	rs2472304	5
chr15	75033399	rs2472299	6
chr15	75027879	rs2472297	4
chr15	75019448	rs2470893	4

chr8	140519516	rs2468677	6
chr15	45698792	rs2467853	6
chr10	126144178	rs2459210	6
chr7	29218158	rs245914	4
chr8	105359431	rs2458413	5
chr6	20109591	rs2457335	5
chr8	128192980	rs2456449	5
chr5	127169211	rs245201	6
chr11	120040441	rs2444240	5
chr16	4038386	rs2444217	5
chr10	68165266	rs2441755	6
chr8	32412358	rs2439312	1f
chr8	32432368	rs2439302	1f
chr10	17891821	rs2437258	6
chr5	159879977	rs2431697	1d
chrX	117088055	rs2430212	6
chr7	122202592	rs2429582	6
chr20	44702119	rs2425752	1d
chr17	44019711	rs242557	5
chr20	24717084	rs2424635	5
chr20	19363978	rs2424234	6
chr1	169491554	rs2420371	6
chr6	32804069	rs241428	5
chr15	50873343	rs2414059	6
chr22	39659772	rs2413583	6
chr22	37470223	rs2413450	5
chr22	30592068	rs2412980	1f
chr22	30529630	rs2412973	6
chr22	30494370	rs2412971	6
chr22	30486825	rs2412970	6
chr17	47445750	rs2411984	6
chr6	111673713	rs240993	6
chr11	97682563	rs2405657	6
chr4	154995462	rs2404916	3a
chr21	11002010	rs240444	6
chr11	18325145	rs2403254	6
chr7	2298226	rs2398668	1f
chr21	28696475	rs239713	6
chr6	32387808	rs2395163	6
chr6	31431779	rs2395029	5
chr10	65133155	rs2393967	5
chr12	121423955	rs2393791	6
chr7	35401446	rs2392362	6
chr1	90943906	rs2390582	6
chr10	8954223	rs2388896	4
chr13	29961331	rs2388082	6
chr10	129933455	rs2387326	6

chr12	115352730	rs2384550	6
chr12	113607501	rs2384207	5
chr8	74066178	rs2383876	6
chr4	180664652	rs2383393	6
chr14	33282469	rs2383378	6
chr20	5588448	rs238295	4
chr2	219151217	rs2382817	1f
chr9	6193454	rs2381416	6
chr10	5886733	rs2380205	3a
chr3	8808514	rs237899	5
chr9	82039361	rs2378383	6
chr20	47903018	rs237743	3a
chr7	82337858	rs2371208	6
chr10	32634971	rs2370759	1f
chr7	81786219	rs2367911	6
chr1	44215827	rs2367725	5
chr7	75059330	rs236670	6
chr2	234698789	rs2361502	6
chr14	76812490	rs2360997	6
chr17	831666	rs2360111	4
chr10	20899607	rs2359536	6
chr12	66117557	rs2358944	6
chr2	9924386	rs2357266	6
chr4	145522757	rs2353398	6
chr14	49372800	rs2352904	6
chr7	8718079	rs2349775	5
chr21	44445291	rs234720	1f
chr2	46642248	rs2346177	6
chr1	161946726	rs2340727	5
chr12	109895167	rs2338104	6
chr6	1573612	rs2338	4
chr3	53118738	rs2336725	1b
chr1	12046062	rs2336384	1b
chr1	11736599	rs2336030	4
chr16	71653636	rs2334880	6
chr14	73767213	rs2333194	5
chr1	85772008	rs233100	6
chr6	137973067	rs2327832	6
chr6	6612466	rs2326810	5
chr20	6035227	rs2326679	4
chr16	84987678	rs2326458	6
chr17	46720564	rs2326017	5
chr13	61965302	rs2323266	6
chr17	64098544	rs2319125	6
chr11	2752608	rs231906	5
chr2	204734486	rs231779	5
chr20	62418336	rs2315656	5

chr17	39046880	rs2315504	6
chr2	97413487	rs2314398	5
chr2	58222927	rs2312147	6
chr2	102663627	rs2310173	6
chr15	89863927	rs2307449	6
chr3	138119951	rs2306374	4
chr15	51569409	rs2305707	4
chr4	103457417	rs230529	6
chr19	19789527	rs2304130	6
chr5	149406732	rs2304069	2b
chr19	17420288	rs2303745	4
chr2	27715415	rs2303369	6
chr6	32725192	rs2301271	6
chr14	33207521	rs2300835	5
chr1	117104214	rs2300747	6
chr14	76005556	rs2300603	4
chr2	66781452	rs2300478	5
chr8	17800768	rs2299587	6
chr11	59837191	rs2298585	5
chr1	200960306	rs2297909	5
chr10	99359738	rs2297644	6
chr20	33722862	rs2295888	5
chr22	37591317	rs229541	6
chr13	28491197	rs2293941	5
chr8	116599198	rs2293889	2b
chr11	47440757	rs2293579	1f
chr3	119219933	rs2293370	3a
chr17	40481528	rs2293152	4
chr12	102513530	rs2292303	4
chr12	56482179	rs2292239	1f
chr1	200826768	rs2292096	6
chr17	38066239	rs2290400	6
chr15	79224682	rs2289700	5
chr19	49228271	rs2287921	4
chr17	42193184	rs228769	4
chr8	47947700	rs2287654	6
chr14	77684841	rs2287375	5
chr19	46202171	rs2287019	6
chr7	72987353	rs2286276	2b
chr4	103578636	rs228614	6
chr7	21584087	rs2285947	5
chr1	17306674	rs2284746	4
chr21	34776694	rs2284553	4
chr22	38544297	rs2284063	4
chr22	37534033	rs2284033	5
chr22	31013295	rs2283873	3a
chr7	92264409	rs2282978	2b

chr14	90758890	rs2282032	6
chr1	201081942	rs2281845	5
chr14	24033069	rs2281680	5
chr6	33060117	rs2281388	4
chr8	22404839	rs2280890	6
chr15	89395625	rs2280470	5
chr19	50000008	rs2280401	4
chr16	86018632	rs2280381	4
chr6	160668388	rs2279463	6
chr19	17283302	rs2279008	5
chr2	101668856	rs2278729	1f
chr15	80694921	rs2278702	5
chr20	34152781	rs2277862	1f
chr17	54773237	rs227731	6
chr5	156932375	rs2277027	6
chr20	52610495	rs2276498	6
chr1	160852045	rs2274910	5
chr9	4985878	rs2274471	4
chr14	55614635	rs2274273	6
chr6	25488582	rs2274089	4
chr9	114348616	rs2273788	5
chr20	10639542	rs2273061	2b
chr6	32337629	rs2273017	4
chr4	76889387	rs2273	1f
chr12	53554282	rs2272306	5
chr2	227915923	rs2272205	6
chr12	66224460	rs2272046	5
chr2	97405439	rs2271893	2b
chr16	67902069	rs2271293	6
chr6	32076498	rs2269426	1f
chr1	64108770	rs2269241	5
chr14	69408696	rs2268983	4
chr21	34781049	rs2268241	5
chr22	29793640	rs2267138	6
chr22	21922903	rs2266959	4
chr16	580123	rs2266928	4
chr10	94168796	rs2263638	5
chr12	121435586	rs2259816	1f
chr14	30594656	rs225848	5
chr6	142527141	rs225694	6
chr6	31380528	rs2256183	6
chr6	31431690	rs2255221	4
chr8	11343679	rs2254546	6
chr6	33143947	rs2254287	5
chr1	17834741	rs2254135	2b
chr1	8422675	rs2252865	5
chr7	54978923	rs2252586	5

chr7	29041189	rs2252521	6
chr1	153913769	rs2252508	4
chr17	30896454	rs225212	1f
chr15	43836477	rs2251844	6
chr1	159272059	rs2251746	5
chr8	11119036	rs2251301	5
chr11	112085315	rs2250417	1f
chr15	40322551	rs2250402	4
chr10	91962669	rs2250245	6
chr10	135352152	rs2249694	4
chr20	52791517	rs2248359	4
chr6	31265489	rs2247056	6
chr12	121416987	rs2244608	5
chr1	187412041	rs2244067	5
chr18	42956671	rs2243803	6
chr3	159709650	rs2243123	3a
chr21	40465177	rs2242944	5
chr10	64580574	rs224278	2b
chr11	66335307	rs2242663	1f
chr5	1280027	rs2242652	5
chr4	68447248	rs2242330	4
chr15	68086837	rs2241423	5
chr2	217554212	rs2241193	5
chr7	72856268	rs2240466	5
chr22	29192669	rs2239815	2b
chr14	23589056	rs2239633	4
chr14	74551969	rs2239557	3a
chr3	52855228	rs2239547	1f
chr15	92685777	rs2238355	6
chr12	112211832	rs2238151	2a
chr11	2858545	rs2237897	4
chr11	2857193	rs2237895	5
chr11	2839750	rs2237892	5
chr11	2810730	rs2237886	4
chr11	2766281	rs2237878	5
chr1	19032271	rs2236824	5
chr11	126283784	rs2236653	5
chr9	4844264	rs2236496	5
chr6	167360388	rs2236313	6
chr1	169580289	rs2235302	3a
chr8	2040103	rs2235121	6
chr5	150440096	rs2233287	5
chr19	41281015	rs2233152	5
chr11	65656563	rs2231884	1b
chr14	23977009	rs223116	5
chr7	100769537	rs2227631	1b
chr6	32413458	rs2227139	1f

chr9	120137757	rs2226006	5
chr10	85422853	rs2224865	5
chr3	159259290	rs2222328	5
chr8	28791158	rs2221894	6
chr8	127911595	rs2220321	5
chr8	72263955	rs2218488	5
chr2	211616893	rs2216405	6
chr12	22365834	rs2216228	5
chr7	147702691	rs2214681	6
chr6	98937145	rs2213553	6
chr11	5303062	rs2213169	6
chr20	16311309	rs2208059	6
chr20	11174902	rs2207418	6
chr6	50786007	rs2206271	5
chr6	35404353	rs2206030	5
chr1	173191474	rs2205960	4
chr17	3664974	rs220470	5
chr21	43502761	rs220299	4
chr4	111710168	rs2200733	6
chr1	160864220	rs2199936	6
chr12	62234832	rs2198776	6
chr8	19826372	rs2197089	6
chr11	119235403	rs2195525	4
chr2	8036259	rs2193071	3a
chr19	44511388	rs2191566	6
chr5	131770804	rs2188962	2a
chr6	32605883	rs2187668	1f
chr17	6607017	rs218676	6
chr10	96751269	rs2185570	4
chr10	119418103	rs2184898	5
chr8	116110724	rs218361	6
chr4	55395023	rs218238	6
chr20	21853099	rs2180439	6
chr1	30627711	rs2180233	6
chr1	88802011	rs2179965	6
chr6	149762536	rs2179367	5
chr2	194872182	rs2176528	6
chr3	122329159	rs2173763	4
chr5	2724361	rs2173226	5
chr15	93131631	rs2173063	5
chr12	103125689	rs2172873	5
chr4	75223710	rs2168889	6
chr18	58375933	rs2168303	6
chr2	24692808	rs2165738	1f
chr9	33799369	rs216345	6
chr6	158041282	rs2163287	5
chr17	2126503	rs216172	4

chr11	116647606	rs2160669	5
chr2	65220909	rs2160387	6
chr19	45695737	rs2159324	5
chr19	950379	rs2159128	5
chr7	107580838	rs2158836	5
chr9	22033365	rs2157719	5
chr7	32347334	rs215614	6
chr18	26606646	rs2155929	6
chr11	76299193	rs2155219	5
chr6	108988183	rs2153960	4
chr9	16864520	rs2153271	5
chr6	10897487	rs2153157	6
chr9	82345880	rs2151145	5
chr9	5893860	rs2150702	5
chr21	40569778	rs2150410	1f
chr10	81121695	rs2145998	5
chr20	6626217	rs2145272	5
chr1	230294915	rs2144300	4
chr20	13148151	rs2144134	5
chr6	41623032	rs2143678	1f
chr22	44391685	rs2143571	5
chr10	43341104	rs2142991	6
chr6	16197193	rs2142672	5
chr2	233513174	rs2140773	5
chr6	34975414	rs2140418	6
chr2	17988657	rs214034	6
chr17	27703348	rs2138852	5
chr2	63511434	rs2138798	6
chr1	90875887	rs2136093	6
chr3	72023699	rs2135319	5
chr6	99672013	rs2132683	6
chr3	194858373	rs2131877	5
chr4	185639168	rs2130392	5
chr1	21654734	rs213032	5
chr15	31829706	rs2125623	6
chr2	182328213	rs2124440	6
chr18	65017099	rs2124349	6
chr6	159490435	rs212388	4
chr2	19945576	rs2123536	5
chr5	44365544	rs2121875	1b
chr2	149557859	rs2121433	5
chr14	76650763	rs2121070	5
chr12	54270227	rs2120991	6
chr12	23639916	rs2120771	6
chr1	8447721	rs2120461	2c
chr3	59981524	rs212016	5
chr1	76106674	rs211718	6

chr11	83256867	rs2116483	5
chr8	73363988	rs2116078	5
chr11	112051168	rs2115763	6
chr19	7196564	rs2115386	5
chr5	122685127	rs2115172	6
chr2	170624220	rs2114646	5
chr4	55092625	rs2114039	5
chr5	75015241	rs2112347	6
chr19	32917454	rs2111504	1f
chr7	150517021	rs2110001	6
chr7	20791997	rs2108258	6
chr7	19049387	rs2107595	2b
chr7	118159548	rs2106595	6
chr22	31645758	rs2106294	1f
chr6	33825357	rs2104362	5
chr10	6099044	rs2104286	5
chr6	47230614	rs2103868	5
chr6	33546836	rs210142	2b
chr20	31277093	rs210135	5
chr6	33540208	rs210134	1f
chr12	117002657	rs2089222	5
chr3	170971290	rs2088885	5
chr4	111334729	rs2087160	6
chr3	196553866	rs2084385	6
chr8	19865174	rs2083637	5
chr5	158717788	rs2082412	5
chr17	59496648	rs2079795	1f
chr17	59465696	rs2079742	5
chr15	92657372	rs207954	1f
chr6	21996859	rs2078543	4
chr11	64334113	rs2078267	4
chr1	183358404	rs2078087	5
chr11	62197426	rs2077224	6
chr21	45709152	rs2075876	5
chr22	37310045	rs2075726	5
chr7	100240295	rs2075672	4
chr19	45395618	rs2075650	1f
chr17	48274290	rs2075555	4
chr11	116732511	rs2075292	4
chr17	33324381	rs2074518	1f
chr6	31240430	rs2074488	1f
chr17	35898341	rs2074409	6
chr17	44865438	rs2074404	1f
chr12	112645400	rs2074356	5
chr11	2484802	rs2074238	2a
chr7	18877873	rs2073963	6
chr5	131723287	rs2073643	4

chr22	24999103	rs2073398	2b
chr22	44394401	rs2073080	5
chr20	9365302	rs2072910	5
chr2	177042632	rs2072590	4
chr17	47390013	rs2072153	6
chr22	35777617	rs2071748	5
chr8	99129506	rs2071598	3a
chr6	32165443	rs2071278	1f
chr6	32171682	rs2071277	6
chr9	133748082	rs2070997	6
chr5	131819920	rs2070729	1f
chr12	49218170	rs2070615	2b
chr3	38442489	rs2070488	6
chr4	123373132	rs2069772	6
chr4	123377979	rs2069762	5
chr12	56364320	rs2069408	6
chr6	34302868	rs206936	5
chr1	234984987	rs2069084	3a
chr10	94839641	rs2068888	2b
chr13	69530663	rs2066219	5
chr10	112877800	rs2065779	5
chr3	102203044	rs2063640	5
chr3	56966245	rs2062583	5
chr8	120007419	rs2062377	5
chr8	119977791	rs2062375	6
chr13	43052879	rs2062305	4
chr19	44614207	rs2061333	6
chr19	7166108	rs2059807	5
chr1	217718131	rs2059397	5
chr16	78258809	rs2059238	6
chr2	207121781	rs2058710	5
chr2	103054448	rs2058660	6
chr12	3913426	rs2058350	4
chr7	72311816	rs2058059	4
chr7	94938256	rs2057681	2b
chr6	117819356	rs2057314	4
chr3	56530708	rs2054989	3a
chr3	178341581	rs2054399	5
chr16	28052962	rs205391	6
chr3	19375790	rs2053506	5
chr5	78272941	rs2052550	5
chr6	32730085	rs2051549	6
chr6	32339075	rs2050190	4
chr6	32155580	rs204993	1f
chr1	155194979	rs2049805	1f
chr7	42176281	rs2049622	6
chr18	42090666	rs2048485	5

chr4	146853164	rs2048161	6
chr5	25087724	rs2047267	6
chr6	151948365	rs2046210	6
chr15	71679958	rs2044029	6
chr15	58680953	rs2043085	6
chr16	74978617	rs2042415	6
chr20	52268994	rs2041278	3a
chr5	137419988	rs2040862	6
chr5	131973176	rs2040704	5
chr14	29249348	rs2038256	5
chr13	108484453	rs2036707	6
chr15	78851614	rs2036527	1f
chr16	8329326	rs2034588	6
chr5	153509595	rs2033195	5
chr21	35354522	rs2032314	6
chr11	73036480	rs2027760	4
chr9	112596910	rs2025875	5
chr20	60212493	rs2024714	5
chr7	43158476	rs2024125	6
chr19	1124030	rs2024092	5
chr22	18972449	rs2023634	5
chr6	30174130	rs2021722	4
chr13	50986117	rs201789	5
chr16	637211	rs2017567	4
chr10	70712061	rs2017305	6
chr12	29435479	rs2015599	4
chr8	133103289	rs2014357	6
chr12	121175523	rs2014355	5
chr21	36357860	rs2014300	5
chr15	87053845	rs2011905	5
chr12	219987	rs2011738	6
chr5	10159540	rs2009501	6
chr4	67300801	rs2009314	5
chr4	5221537	rs2008242	4
chr9	117592637	rs2006996	5
chr17	26601132	rs2006933	6
chr18	76409596	rs2002842	5
chr3	185629567	rs2002675	5
chr8	11276541	rs2002030	5
chr8	126477977	rs2001945	4
chr16	72108092	rs2000999	6
chr6	116387133	rs1999930	6
chr6	152068363	rs1999805	5
chr1	197727641	rs1998598	6
chr14	94957713	rs1998207	6
chr3	25538316	rs1997352	5
chr17	44856640	rs199515	1f

chr12	40428560	rs1994090	6
chr15	79080233	rs1994016	6
chr2	200290358	rs1992950	6
chr5	40415066	rs1992660	5
chr8	58840923	rs1992045	6
chr8	130624104	rs1991866	6
chr2	236128950	rs1991705	5
chr4	77420783	rs1986734	2b
chr4	125993501	rs1986655	5
chr22	45961903	rs1985671	5
chr11	61490485	rs198426	5
chr6	41536426	rs1983891	4
chr1	85311191	rs1983853	6
chr3	177793106	rs1982821	5
chr16	630664	rs1981483	5
chr4	145485737	rs1980057	5
chr5	15582004	rs1978633	5
chr3	37515826	rs197770	6
chr1	21766452	rs1976403	4
chr1	96943993	rs1973993	6
chrX	119867474	rs1972809	5
chr18	52863107	rs1970671	6
chr20	59853937	rs1970546	5
chr3	183876511	rs1969253	5
chr1	145723644	rs1967017	2a
chr4	89875908	rs1964516	6
chr8	73106915	rs1963982	6
chr4	169338519	rs1963569	5
chr14	41523461	rs1959947	5
chr9	123640499	rs1953126	2b
chr1	115734090	rs195204	6
chr14	27260042	rs1951082	6
chr4	8503358	rs1949733	6
chr14	69273904	rs194749	3a
chr11	56175670	rs1945213	6
chr11	134482465	rs1944866	4
chr18	45815416	rs1944582	6
chr18	71058621	rs1943816	6
chr7	139557946	rs194151	6
chr18	12550746	rs1940973	4
chr11	126809704	rs1939992	5
chr5	25692972	rs193741	6
chr1	190066385	rs1935881	6
chr10	96797469	rs1934953	6
chr10	96798547	rs1934951	5
chr1	109999837	rs1933182	6
chr6	45696787	rs1932040	4

chr22	25875264	rs1930961	1f
chr6	22017737	rs1928168	5
chr13	107780315	rs1927745	5
chr9	15986715	rs1927702	6
chr13	95874955	rs1926657	3a
chr4	2103095	rs1923775	5
chr3	169165887	rs1918974	6
chr10	57416960	rs1916521	6
chr3	57394765	rs1916284	6
chr1	239075713	rs1915279	5
chr15	76546932	rs1914816	5
chr3	145888456	rs1913185	6
chr4	187149539	rs1912826	5
chr3	146402170	rs1912785	6
chr8	96515887	rs1909881	4
chr6	50987767	rs190759	4
chr15	61164198	rs1902618	5
chr3	31795569	rs1902341	5
chr13	32125314	rs1902272	6
chr10	70000880	rs1900004	6
chr3	37518544	rs189897	6
chr5	110408001	rs1898671	5
chr15	36349845	rs1898036	6
chr8	8990576	rs189798	4
chr12	115346423	rs1896312	6
chr12	114802137	rs1895585	6
chr11	102070638	rs1894116	6
chr11	123956465	rs1893767	4
chr18	12809339	rs1893217	5
chr18	905124	rs1893154	5
chr9	26789939	rs1889899	5
chr9	113300834	rs1889321	3a
chr13	74520185	rs1886512	5
chr14	101251988	rs1884537	5
chr20	7106288	rs1884302	6
chr6	24491474	rs1883415	1f
chr6	33086447	rs1883414	3a
chr9	107664300	rs1883025	5
chr1	247689531	rs1881797	6
chr12	41721429	rs1880887	5
chr4	114379378	rs1880529	5
chr3	180551213	rs1879248	5
chr4	148393663	rs1878406	6
chr2	65608908	rs1876518	3a
chr2	19134747	rs1876040	5
chr7	138157389	rs1874326	4
chr15	63312631	rs1873147	3a

chr19	45326767	rs1871045	4
chr15	40595626	rs1869901	5
chr7	114357542	rs1869839	6
chr4	59305131	rs1869463	6
chr10	73527046	rs1867982	2b
chr16	86681030	rs1867485	5
chr1	30185661	rs1866967	5
chr18	73192445	rs1865721	6
chr19	22554853	rs1865075	6
chr5	146320822	rs1864982	6
chr17	43977826	rs1864325	6
chr16	56997232	rs1864163	5
chr19	10000321	rs1862471	5
chr4	15397905	rs1861046	4
chr17	69108752	rs1859962	5
chr4	95834033	rs1859156	6
chr1	75913825	rs1857353	5
chr13	30885222	rs185694	6
chr4	9790711	rs1850744	6
chr11	89005252	rs1847134	6
chr4	120962384	rs1845344	6
chr5	57320953	rs1844437	6
chr15	101251718	rs184234	5
chr5	110401871	rs1837253	5
chr12	93550300	rs1836127	5
chr8	98166912	rs1835740	6
chr15	48392164	rs1834640	6
chr11	112023826	rs1834481	5
chr14	77497974	rs183266	6
chr17	44788309	rs183211	6
chr3	133508463	rs1830084	6
chr2	161785168	rs1829975	6
chr5	98781102	rs1829883	6
chr4	145480779	rs1828591	6
chr2	192118046	rs1823913	1f
chr3	186560781	rs182052	5
chr10	59913150	rs1819658	6
chr6	167373546	rs1819333	1f
chr13	24980948	rs1816752	5
chr10	119379655	rs181654	6
chr10	72483009	rs1816002	5
chr10	119181870	rs181500	6
chr22	21932067	rs181362	5
chr22	21928640	rs181359	1f
chr4	121801789	rs180730	6
chr9	90031668	rs1806864	6
chr11	116599391	rs180358	5

chr7	93538294	rs180273	6
chr1	206946633	rs1800871	5
chr4	155483913	rs1800788	4
chr16	56995235	rs1800775	3a
chr12	6440008	rs1800693	5
chr15	58723674	rs1800588	4
chr6	31542307	rs1799964	4
chr7	44229067	rs1799884	5
chr3	55571759	rs1795648	5
chr1	171091874	rs1795240	5
chr6	32671247	rs1794275	5
chr12	123656724	rs1790100	5
chr4	100274285	rs1789924	4
chr3	3147980	rs17879755	5
chr18	46587653	rs1787200	6
chr7	126639603	rs17864092	6
chr11	125304008	rs1783925	5
chr7	139702592	rs17837497	5
chr14	56886685	rs17832777	5
chr17	29333515	rs17826255	1c
chr11	60760611	rs17824933	4
chr16	60733602	rs17822114	6
chr2	46826025	rs17818399	6
chr16	53813366	rs17817449	5
chr3	159665049	rs17810546	5
chr3	67416321	rs17806888	6
chr1	21821756	rs1780324	1f
chr9	15157976	rs1780159	5
chr18	47121315	rs17798991	5
chr16	79406917	rs17797882	4
chr14	91022184	rs17793829	5
chr8	23777005	rs17786744	6
chr17	30535855	rs17780304	5
chr13	36177818	rs1777672	6
chr9	25551356	rs17774966	6
chr18	57963116	rs17773430	5
chr6	34195010	rs1776897	4
chr16	79744547	rs17767419	6
chr19	3251218	rs17764205	2b
chr5	173359330	rs17763373	5
chr17	2171636	rs17761864	1f
chr17	54615616	rs17760296	6
chr18	54397621	rs17750015	6
chr21	30713597	rs17744121	5
chr15	53942927	rs17730436	6
chr5	150478317	rs17728338	5
chr18	3512215	rs17724172	4

chr1	159005648	rs1772408	6
chr17	68644188	rs17718586	6
chr2	163260690	rs17716942	5
chr1	82765051	rs1770678	6
chr1	213855587	rs17706439	6
chr12	112486817	rs17696736	5
chr5	173337852	rs17695092	6
chr19	33731550	rs17694108	5
chr6	27710164	rs17693963	6
chr7	126324590	rs17691394	5
chr4	55234824	rs17690232	6
chr3	39523002	rs1768208	6
chr15	29239816	rs17680945	5
chr16	76890125	rs17679567	5
chr18	43309910	rs17674580	5
chr1	240445595	rs17672135	5
chr8	118549375	rs17667932	6
chr5	72432035	rs17663555	5
chr2	193984620	rs17662626	6
chr10	18397246	rs17661538	6
chr11	270714	rs17655730	6
chr3	71020489	rs17651978	6
chr15	87436744	rs17647114	6
chr1	152062766	rs17646946	4
chr17	64917032	rs17645023	4
chr19	12225371	rs17638629	6
chr4	162602208	rs17638464	5
chr4	167343938	rs17635075	4
chr6	134544732	rs1763500	6
chr5	72431481	rs17632159	4
chr2	121089730	rs17625845	5
chr2	105158637	rs17624523	6
chr7	140592226	rs17623382	6
chr14	103822761	rs17616316	3a
chr5	16913854	rs17614462	5
chr15	93815128	rs17612678	4
chr6	35034799	rs17609940	5
chr6	32158318	rs176095	1f
chr17	38110688	rs17609240	5
chr10	72462993	rs17600642	5
chr4	176662086	rs17599018	6
chr7	96581810	rs17598306	6
chr18	53058237	rs17594526	5
chr5	2335580	rs17586674	5
chr10	35287649	rs17582416	5
chr9	81310679	rs1757948	4
chr5	141863603	rs17577085	6

chr6	37487043	rs1757171	6
chr5	76046938	rs17568628	6
chr15	27997246	rs17565841	6
chr2	19958495	rs17564315	6
chr9	17938350	rs1755289	4
chr9	17979577	rs1755271	6
chr2	47229838	rs17540621	5
chr15	51969667	rs17525472	6
chr1	65992624	rs1751492	5
chr7	46392566	rs17513961	6
chr5	110146445	rs17513503	6
chr17	59323042	rs17513268	5
chr2	37960612	rs17511102	5
chr3	189401775	rs17505102	6
chr1	85226298	rs1750491	6
chr1	107546374	rs17496332	6
chr2	2926012	rs17491951	5
chr15	78867481	rs17486278	2b
chr2	111797457	rs17483466	1f
chr8	19832645	rs17482753	6
chr1	63049592	rs1748195	6
chr4	16002287	rs17478107	6
chr7	106411857	rs17477177	6
chr8	27452846	rs17466684	4
chr1	222823528	rs17465637	6
chr10	44775823	rs1746048	5
chr11	61609749	rs174583	1f
chr2	216285374	rs17458018	4
chr11	61600341	rs174574	4
chr11	61597211	rs174570	2b
chr11	61571477	rs174550	5
chr11	61571381	rs174549	6
chr11	61571347	rs174548	1f
chr11	61570782	rs174547	6
chr11	61551926	rs174536	5
chr11	61678753	rs174479	5
chr11	61663690	rs174468	5
chr16	86017662	rs17445836	5
chr11	61639572	rs174448	5
chr5	58560716	rs17444059	5
chr12	120880433	rs17431357	6
chr2	185436261	rs17430279	6
chr12	117295332	rs17429217	2b
chr5	94154587	rs17418283	5
chr12	15930804	rs17415853	5
chr20	4101799	rs1741344	5
chr5	166389492	rs17404956	5

chr1	10385470	rs17401966	6
chr7	106409451	rs17398575	6
chr1	78623625	rs17391694	1f
chr4	34893854	rs17390445	6
chr5	111107114	rs173896	4
chr2	202807195	rs17385675	6
chr1	23536890	rs1738475	4
chr5	93557701	rs17376456	5
chr1	11852515	rs17375901	5
chr13	22487624	rs17369571	4
chr1	11862777	rs17367504	4
chr2	123641610	rs17367118	4
chr3	186570452	rs17366568	5
chr7	22514052	rs17364464	6
chr21	16812551	rs1736020	5
chr1	22499529	rs17360053	6
chr13	21915113	rs17356983	5
chr12	96027758	rs17356907	2b
chr19	45740770	rs17356664	1f
chr7	100315516	rs1734907	6
chr1	172053286	rs17346452	6
chr3	52844533	rs17331151	1b
chrX	132137155	rs17324272	6
chr8	126486408	rs17321515	5
chr4	77368846	rs17319721	5
chr19	41937094	rs17318596	1f
chr4	110825633	rs17316633	6
chr6	3433317	rs17309827	4
chr15	59824986	rs17302400	5
chr11	64331461	rs17300741	5
chr6	128021802	rs17299841	5
chr15	67442595	rs17293632	2a
chr16	57024316	rs17290922	4
chr9	32556379	rs17290760	1f
chr2	27635462	rs1728918	4
chr16	68591229	rs1728785	6
chr12	24770877	rs17287293	6
chrX	154233773	rs17281398	5
chr12	123575741	rs1727307	6
chr19	5834211	rs17271883	5
chr2	56592082	rs17268785	6
chr13	93323145	rs17267292	5
chr3	122048643	rs17265703	6
chr4	55407761	rs172629	5
chr1	208757585	rs17259784	5
chrX	149635294	rs17252936	5
chr3	121993246	rs17251221	6

chr12	90060585	rs17249754	6
chr5	40401508	rs17234657	5
chr7	134785740	rs17231212	5
chr15	67458638	rs17228212	5
chr1	150961868	rs1722784	6
chr2	161781772	rs1722636	5
chr16	50739581	rs17221417	4
chr10	618684	rs17221323	5
chr19	19662219	rs17216525	5
chr3	6481624	rs17216035	5
chr5	64433399	rs17206232	6
chr14	21725653	rs17197037	1f
chr15	65183800	rs1719271	4
chr20	13123732	rs17190927	6
chr2	119845316	rs17189298	6
chr18	67142856	rs17184557	6
chr12	65718298	rs17178006	5
chr5	10811473	rs17176973	5
chr2	112665200	rs17174870	6
chr7	33673264	rs17170316	4
chr5	132628883	rs17166496	4
chr12	124519845	rs1716403	4
chr7	103628035	rs17157903	4
chr5	102759657	rs17155315	3a
chr7	106495808	rs17153527	6
chr7	25608408	rs17151904	4
chr7	78043290	rs17150687	6
chr7	75978228	rs17149161	4
chr11	85017475	rs17148090	6
chr11	65249144	rs17146964	4
chr7	73026377	rs17145750	6
chr7	72982873	rs17145738	5
chr10	5918431	rs17145612	5
chr7	21504426	rs17144465	5
chr7	8177396	rs17143586	6
chr6	6341286	rs17142067	6
chr4	57786449	rs1713985	6
chr5	112996653	rs17135859	5
chr1	92211019	rs17131547	4
chr1	67790597	rs17129789	5
chr1	84232747	rs17129289	6
chr14	55122917	rs17127713	4
chr12	52263955	rs17126180	5
chr10	105033014	rs1712517	1f
chr11	118145685	rs17122021	1f
chr12	59440231	rs17121944	5
chr10	107516351	rs17119461	5

chr1	59799160	rs17119280	6
chr6	14719495	rs17119	5
chr11	113961761	rs17116334	6
chr10	104591392	rs17115100	5
chr1	56962820	rs17114036	4
chr10	102066184	rs17112901	6
chr14	27406891	rs17111920	6
chr14	81523127	rs17111394	6
chr14	71249801	rs17108533	6
chr10	123913329	rs17103138	6
chr14	65604727	rs17102423	4
chr10	111735749	rs17095355	5
chr14	59189360	rs17094983	5
chr3	28079084	rs170934	5
chr13	28929710	rs17086609	5
chr18	69439072	rs17085106	6
chr13	27531266	rs17085007	5
chr4	55087580	rs17084051	6
chr18	68842242	rs17083844	6
chr3	39847071	rs17079534	5
chr13	85665878	rs17079247	4
chr18	65285278	rs17077540	6
chr13	66356340	rs17077331	6
chr8	6351251	rs17077154	6
chr3	43230722	rs17075286	6
chr4	184278130	rs17074636	5
chr6	108323758	rs17069122	4
chr6	137452907	rs17066096	6
chr13	45055090	rs17065868	6
chr6	105406273	rs17065417	5
chr13	43570800	rs17064002	4
chr6	130031492	rs17057678	4
chr18	72787045	rs17056274	5
chr4	169530257	rs17054392	5
chr9	91260561	rs17054265	5
chr4	140423133	rs17050782	6
chr2	121306439	rs17050272	5
chr4	138582710	rs17049741	5
chr18	66314071	rs1704734	6
chr4	166255703	rs17046216	1f
chr3	66768363	rs17045031	5
chr4	111708286	rs17042171	5
chr12	108742819	rs17040430	5
chr1	118868404	rs17038182	5
chr3	12330410	rs17036170	4
chr3	12277844	rs17036101	6
chr2	68598954	rs17035378	2c

chr4	102560289	rs17031508	6
chr2	43687878	rs17030845	4
chr1	113190806	rs17030613	6
chr4	154703595	rs17030434	4
chr3	31445579	rs17027625	6
chr2	103091539	rs17027258	6
chr1	110645326	rs17025426	5
chr4	95614277	rs17022027	5
chr4	95562876	rs17021918	5
chr2	37248014	rs17020136	1b
chr19	51419545	rs1701930	6
chr12	56412486	rs1701704	6
chr3	71300566	rs17008402	4
chr4	142709722	rs17007695	5
chr4	142142728	rs17007017	4
chr2	122018762	rs17006292	4
chr4	77771344	rs17002253	2b
chr22	40996366	rs17002034	6
chr21	27002376	rs17001239	6
chr22	49812501	rs17000918	5
chr20	52807220	rs16999165	6
chr21	42471627	rs16998084	6
chr4	81184340	rs16998073	6
chr19	19658471	rs16996148	1f
chr21	36849418	rs16993221	4
chr22	45183013	rs16992796	6
chrX	32015375	rs16990008	6
chr20	4169078	rs16989303	6
chr19	56497959	rs16986856	5
chr19	53682041	rs16984547	5
chr20	57758719	rs16982520	5
chr15	86984239	rs16977195	6
chr16	27214983	rs16976587	4
chr15	55456509	rs16976033	5
chr19	38325535	rs16975963	6
chr1	21823291	rs1697421	6
chr16	71965195	rs16973500	3a
chr17	33944054	rs16971217	6
chr16	21113271	rs16970881	5
chr17	75944649	rs16970672	3a
chr16	89851032	rs16966142	5
chr16	57047298	rs16965039	3a
chr15	51530494	rs16964211	6
chr13	104186918	rs16962638	6
chr16	59940187	rs16961543	6
chr17	7633691	rs16956936	5
chr16	81489372	rs16955379	5

chr16	74881819	rs16948255	5
chr17	47566311	rs16948200	5
chr15	44219606	rs16948098	4
chr17	47440465	rs16948048	4
chr13	92203812	rs16946160	6
chr16	67928041	rs16942887	4
chr18	24156424	rs16942421	6
chr16	86014240	rs16940202	5
chr15	58471978	rs16939881	4
chr8	75373947	rs16938910	5
chr11	46052574	rs16938437	5
chr9	20098710	rs16937883	6
chr9	119839327	rs16934284	4
chr9	36969204	rs16933812	2b
chr11	16040174	rs16932455	6
chr11	37870980	rs16930685	6
chr11	2936951	rs16928809	1f
chr10	72982984	rs16928529	5
chr11	2486119	rs16928297	5
chr10	71093391	rs16926246	4
chr9	7043454	rs16925187	6
chr11	61417471	rs1692120	1f
chr11	24252527	rs16912285	6
chr9	125573029	rs16912238	5
chr11	5263852	rs16912210	6
chr10	60082667	rs16912145	5
chr11	12070664	rs16910421	4
chr9	97314740	rs16910061	5
chr9	98231007	rs16909898	6
chr9	122861296	rs16909449	6
chr5	169074055	rs169082	2b
chr10	56848984	rs16906916	3b
chr5	36309096	rs16902947	6
chr8	128320345	rs16902094	5
chr8	128124915	rs16901979	6
chr8	117630682	rs16892766	2b
chr6	161933934	rs16892673	6
chr8	38031344	rs16887244	1f
chr5	56023082	rs16886165	5
chr6	54265395	rs16885294	6
chr4	22021499	rs16872248	3a
chr8	105957539	rs16872085	6
chr5	73215236	rs16871023	5
chr6	33851172	rs16869652	6
chr2	181362378	rs16867321	6
chr2	180566677	rs16866933	5
chr3	189997395	rs16865258	5

chr2	224459682	rs16864968	5
chr2	224274621	rs16864755	6
chr2	14398051	rs16861531	5
chr1	18294486	rs16861326	6
chr2	11257504	rs16857178	5
chr1	162112909	rs16857031	5
chr1	205678125	rs16856186	5
chr3	169150631	rs16853722	5
chr3	168686675	rs16852912	4
chr1	177568798	rs16851585	5
chr3	140979410	rs16851254	4
chr4	75170139	rs16850885	3a
chr4	75160823	rs16850864	6
chr4	74872444	rs16850360	6
chr2	164906819	rs16849225	5
chr1	199356515	rs16844716	5
chr2	159670448	rs16843372	5
chr2	156925285	rs16839962	6
chr2	204089729	rs16839626	6
chr4	6058496	rs16838131	5
chr2	155002846	rs16836124	6
chr1	160421915	rs16832015	4
chr11	133800218	rs1682859	5
chr2	150509331	rs16827293	5
chr1	22485870	rs16826658	5
chr6	37174744	rs1680005	6
chr12	57968714	rs1678542	6
chr12	57503774	rs167769	5
chr8	13176406	rs1671400	5
chr4	1078186	rs1670533	6
chr19	34333913	rs166988	6
chr3	3199634	rs1669338	6
chr1	205235989	rs1668873	4
chr1	205237136	rs1668871	4
chr15	28530181	rs1667394	6
chr18	29187278	rs1667255	6
chr16	14388304	rs1659127	6
chr3	186466251	rs1656966	5
chr2	233426525	rs1656402	6
chr1	91569688	rs164898	4
chr16	12641114	rs1641895	2b
chr17	7545720	rs1641537	5
chr17	74283668	rs164009	5
chr7	28189410	rs1635852	2b
chr1	242040774	rs1635501	5
chr11	2844215	rs163182	5
chr5	76781470	rs163030	6

chr17	7578114	rs1625895	5
chr1	98502933	rs1625579	6
chr12	130497471	rs1624802	5
chr18	3328745	rs1623523	5
chr5	104069916	rs161645	4
chr1	7521584	rs1616122	5
chr10	29291897	rs1612122	6
chr8	90656987	rs160441	5
chr3	3658839	rs1601875	6
chr8	11359637	rs1600249	5
chr4	187195550	rs1593	5
chr6	150651899	rs1591830	6
chr5	59369793	rs1588265	6
chr4	112702634	rs1585471	6
chr13	66481814	rs1585440	4
chr6	86691939	rs1577917	6
chr19	45396218	rs157582	6
chr19	45395265	rs157580	1f
chr2	241308504	rs1574192	4
chrX	109820067	rs1573036	5
chr6	858969	rs1572438	5
chr9	121346416	rs1572299	6
chr1	99386292	rs1571500	2b
chr6	12048826	rs1570989	5
chr6	20382599	rs1570155	5
chr20	44742063	rs1569723	1f
chr1	169608916	rs1569476	5
chr2	201021953	rs1569175	6
chr12	131576190	rs1569019	4
chr15	83726178	rs1568657	1f
chr11	132829861	rs1567127	5
chr8	142556804	rs1566080	5
chr16	51021802	rs1566045	6
chr4	852312	rs1564282	5
chr11	60023086	rs1562990	6
chr8	128387851	rs1562430	5
chr10	13155725	rs1561570	1f
chr16	12708207	rs1560104	5
chr15	58080616	rs1559777	5
chr11	116607436	rs1558861	5
chr12	68504591	rs1558744	5
chr7	31155346	rs1558477	6
chr12	6289218	rs1558324	5
chr11	126619189	rs1557488	5
chr18	54752313	rs1557351	2b
chr20	33849178	rs1555322	6
chr8	126540050	rs1551398	4

chr11	131320069	rs1550976	5
chr15	58213413	rs1550576	4
chr2	127330953	rs1550404	6
chr17	53757786	rs1549519	6
chr15	71109146	rs1549318	6
chr21	43778894	rs1547374	5
chr22	29100710	rs1547014	6
chr16	89667336	rs154659	5
chr5	126032963	rs1546498	6
chr12	84564067	rs1545843	6
chr4	106048359	rs1541374	5
chr10	13755543	rs1541010	2b
chr6	466032	rs1540771	4
chr18	69248594	rs1539909	5
chr1	247600300	rs1539019	6
chr6	82794593	rs1538138	5
chr9	138529721	rs1537415	6
chr9	22169699	rs1537377	5
chr9	137440527	rs1536482	5
chr9	7925380	rs1535480	6
chr11	61597971	rs1535	1b
chr2	84350916	rs1534238	6
chr3	133537066	rs1534166	6
chr15	78530939	rs1533665	6
chr16	57005478	rs1532624	4
chr8	27466314	rs1532278	2b
chr14	49951349	rs1530947	6
chr10	63524590	rs1530440	5
chr3	25520581	rs1529672	4
chr13	103928006	rs1529276	6
chr2	64495626	rs1529102	1d
chr2	177909594	rs1529093	5
chr11	90884338	rs1528753	6
chr2	52713806	rs1526687	6
chr3	165280208	rs1523288	6
chr2	202949850	rs1521882	6
chr12	43253414	rs1520832	5
chr8	79401037	rs1520333	6
chr1	206944644	rs1518111	3a
chr8	69389216	rs1517114	6
chr2	227122215	rs1515110	6
chr20	57614001	rs151361	6
chr8	23526462	rs1512268	5
chr16	20674491	rs151222	6
chr16	28490516	rs151181	6
chr3	138713703	rs1511412	5
chr8	55310710	rs1504749	5

chr5	101859316	rs1502844	5
chr3	135235357	rs1502172	4
chr5	13779742	rs1502050	6
chr3	42093421	rs1498095	4
chr1	16505319	rs1497406	2b
chr7	78331464	rs1496766	5
chr1	67753507	rs1495965	6
chr8	18273299	rs1495743	6
chr8	18272880	rs1495741	6
chr12	71577100	rs1495377	4
chr1	220929355	rs1494373	6
chr4	145650020	rs1492820	6
chr12	40620807	rs1491942	1d
chr11	29195136	rs1491818	5
chr4	149321345	rs1490453	5
chr3	21719245	rs1490157	6
chr3	176422651	rs1490075	5
chr7	95631195	rs1488515	6
chr11	41852663	rs1484948	6
chr4	89039081	rs1481012	6
chr10	45161108	rs1480597	5
chr7	86696713	rs1476587	6
chr9	100316229	rs1475545	5
chr10	115235113	rs1472750	2b
chr1	145723738	rs1471633	2b
chr4	88775242	rs1471403	5
chr14	98303330	rs1471356	5
chr15	29888395	rs1471225	5
chr3	185529079	rs1470579	6
chr4	155347392	rs1466662	6
chr10	72688370	rs1466576	4
chr12	57534469	rs1466535	1f
chr14	69263598	rs1465788	4
chr2	43553948	rs1465618	6
chr3	188112553	rs1464510	4
chr12	24389659	rs1464500	5
chr12	131022009	rs1464108	6
chr16	10183086	rs1463132	6
chr8	80227447	rs1460163	6
chr14	98840442	rs1459148	5
chr11	80985547	rs1458095	6
chr4	81164722	rs1458038	6
chr2	65862377	rs1457451	6
chr7	50304460	rs1456896	5
chr7	50269671	rs1456893	4
chrX	32225254	rs1456737	6
chr8	128103936	rs1456315	5

chr15	93050965	rs1455782	5
chr18	11494198	rs1455244	4
chr2	207877464	rs1453160	5
chr21	28744355	rs1452093	3a
chr7	50622711	rs1451375	5
chr8	42546710	rs1451240	6
chr2	23414650	rs1449984	5
chr2	112375962	rs1448190	4
chr2	84246585	rs1447537	6
chr8	128485037	rs1447295	5
chr5	15364483	rs1447276	5
chr8	27076597	rs1446682	6
chr15	68735585	rs1445021	6
chr10	64560469	rs1444418	5
chr12	54342683	rs1443512	4
chr9	100550027	rs1443438	5
chr4	128320907	rs1443170	6
chr8	19868385	rs1441756	6
chr2	223936737	rs1440072	5
chr5	152504928	rs1438949	6
chr15	62404381	rs1436955	4
chr15	62414013	rs1436953	5
chr1	217058478	rs1436900	6
chr2	229510928	rs1435867	3a
chr5	155302581	rs1432723	6
chr2	61066665	rs1432295	4
chr17	54192522	rs1431318	6
chr1	68635074	rs1430742	6
chr4	148322386	rs1429107	6
chr2	137555223	rs1427593	6
chr11	125170638	rs1426153	2b
chr4	76030920	rs1426063	6
chr3	162681994	rs1425609	6
chr5	127151975	rs1421746	5
chr16	53800953	rs1421085	6
chr16	53757739	rs1421084	6
chr18	25167944	rs1420956	6
chr2	102957715	rs1420101	5
chr7	125391644	rs1419607	6
chr10	73135918	rs1417210	2a
chr13	47898878	rs1417205	6
chr1	162085308	rs1415259	6
chr1	95692309	rs1414896	6
chr1	65815658	rs1413885	6
chr13	93213475	rs1413191	6
chr9	22043925	rs1412829	5
chr10	91002926	rs1412444	6

chr9	1456796	rs1412259	6
chr10	34088052	rs1412115	5
chr9	80940575	rs1411916	3a
chrX	82535168	rs1410530	5
chr9	12672096	rs1408799	6
chr6	25842950	rs1408272	3a
chr20	61895919	rs1406961	5
chr7	88560877	rs1406503	5
chr2	51736993	rs1406428	5
chr22	50971265	rs140522	4
chr7	108544460	rs1404697	5
chr17	54839758	rs1401796	5
chr22	23922982	rs140174	2b
chr3	53680123	rs1401492	5
chr1	189861403	rs1400544	6
chr22	40697580	rs139909	2b
chr18	44752237	rs1398217	6
chr10	23665437	rs1398024	4
chr3	103245091	rs1397924	6
chr10	120966338	rs1397615	4
chr4	148047549	rs1395821	6
chr4	178318190	rs1395479	6
chr4	126359455	rs1395241	5
chr15	69398025	rs1392635	5
chr6	119866140	rs1392089	5
chr10	4719795	rs1391511	4
chr1	227797949	rs1390401	6
chr4	11503603	rs1390096	6
chr3	22292570	rs1388551	6
chr1	164689761	rs1387389	5
chr11	92673827	rs1387153	5
chr11	87819426	rs1386330	4
chr12	129300693	rs1385374	4
chr15	84522754	rs1383484	6
chr18	41480526	rs1380836	6
chr17	15193055	rs1380181	3a
chr14	54072857	rs1380131	6
chr15	75077366	rs1378942	1f
chr2	204272089	rs1376877	6
chr3	185531660	rs1374910	4
chr2	120145654	rs1374313	1b
chr12	70054909	rs1373453	6
chr13	106785264	rs1372791	5
chr3	16955258	rs1372072	5
chr3	143250069	rs1371924	5
chr8	101330208	rs1371867	5
chr4	74977836	rs1371799	4

chr7	9189540	rs1371737	4
chr12	76476955	rs1368578	5
chr2	56089539	rs1367226	4
chr8	120224805	rs1364705	6
chr16	69588571	rs1364063	1f
chr5	96252802	rs1363907	6
chr16	51458289	rs1362756	6
chr6	126767599	rs1361108	5
chr10	36885920	rs1360573	6
chr9	13007128	rs1360517	5
chr13	80717155	rs1359790	6
chr2	107678487	rs1357692	6
chr19	51393117	rs1354774	4
chr17	31737420	rs1354492	6
chr3	56849748	rs1354034	6
chr18	49404963	rs1351435	6
chr12	66351825	rs1351394	5
chr4	75224589	rs1350666	5
chr11	91952153	rs1350445	6
chr10	63348766	rs1350172	3a
chr12	20531755	rs1348582	5
chr2	216893636	rs1344694	6
chr3	169300218	rs1344555	6
chr7	105542579	rs13438712	6
chr7	97620690	rs13438327	1f
chr6	31354559	rs13437082	6
chr5	129000385	rs13436218	4
chr4	56467213	rs13434995	6
chr2	201648638	rs13430864	6
chr2	43638837	rs13429458	5
chr2	25492466	rs13428812	6
chr6	44739948	rs1342371	3a
chr9	6190075	rs1342326	6
chr1	173301515	rs1342038	2b
chr2	127662896	rs13418717	6
chr2	44662474	rs13414205	5
chr6	22304203	rs1341239	5
chr2	79539987	rs13409348	6
chr2	168565692	rs13408808	6
chr2	102955081	rs13408661	5
chr2	53782558	rs13407662	6
chr2	138142163	rs13405020	6
chr2	219081296	rs13403276	5
chr2	35662796	rs13402855	5
chr2	120513132	rs13401620	6
chr2	84245167	rs13398848	6
chr2	231091222	rs13397985	4

chr2	234502120	rs13394720	3a
chr2	73818935	rs13391552	6
chr2	26272601	rs13388915	5
chr2	33701889	rs13385731	4
chr2	20888264	rs13385191	6
chr2	209578599	rs13383928	6
chr1	154814352	rs13376333	1f
chr1	239644170	rs13373941	6
chr5	40791883	rs13361707	4
chr5	150223386	rs13361189	5
chr5	167500459	rs13358864	3a
chr5	78845710	rs13358260	6
chr1	111684275	rs1335645	4
chr1	117100956	rs1335532	3a
chr14	58385364	rs1335515	5
chr18	65400424	rs13353224	2b
chr16	1532462	rs13336428	4
chr16	10559262	rs13335336	6
chr16	20365653	rs13333226	6
chr9	22136488	rs1333051	5
chr16	76878861	rs13330107	5
chr3	62478193	rs13325751	6
chr3	85502844	rs13323436	6
chr3	58556840	rs13315591	5
chr3	33015468	rs13314993	5
chr1	203492719	rs13303128	6
chr9	4814947	rs13300663	5
chr6	142753337	rs1329705	6
chr10	93348119	rs1329650	4
chr9	37037975	rs1329568	4
chr1	196702809	rs1329428	5
chr9	29545874	rs13290799	6
chr9	12396730	rs13289810	6
chr8	66974251	rs13279522	4
chr8	99088580	rs13278732	5
chr8	23082970	rs13278062	1f
chr8	11349185	rs13277113	1d
chr9	113131162	rs1327533	6
chr8	70493973	rs13273088	5
chr8	23584225	rs13273073	4
chr8	79034458	rs13272568	6
chr8	17282410	rs13271465	6
chr10	19929512	rs1326986	5
chr8	142632314	rs13263959	5
chr8	124714721	rs13258681	5
chr1	176792248	rs1325598	6
chr8	128104342	rs13254738	2b

chr8	28995021	rs13251954	2b
chr9	12582564	rs1325154	6
chr7	73010441	rs13247874	5
chr9	13557490	rs1324183	5
chr13	43727848	rs1324015	4
chr7	72971230	rs13233571	4
chr1	192541020	rs1323292	4
chr7	151120947	rs13232179	6
chr7	36961319	rs13230047	6
chr6	17241153	rs1322846	5
chr7	73017004	rs13226650	4
chr7	106763217	rs13224682	5
chr6	36622899	rs1321311	1f
chr6	109598963	rs13210693	6
chr1	169073345	rs1320976	4
chr6	168941623	rs13208776	2b
chr6	93197299	rs13207034	6
chr6	127167071	rs13204965	6
chr6	128245764	rs13204742	5
chr10	105846073	rs1320448	5
chr2	679178	rs1320333	6
chr4	126933291	rs1320267	6
chr6	10163967	rs13195786	2b
chr6	26500562	rs13194984	4
chr6	27037079	rs13194491	6
chr6	27143882	rs13194053	6
chr6	31241108	rs13191343	4
chr5	105297528	rs13189969	3a
chr5	112723566	rs1318772	6
chr6	6331935	rs1318606	4
chr5	138850904	rs13181561	1f
chr22	50976753	rs131788	4
chr5	149825869	rs13177918	3a
chr5	108113343	rs13177718	6
chr5	92487542	rs13173682	6
chr1	20140035	rs1317209	5
chr3	169497584	rs1317082	6
chr5	158175668	rs13170526	5
chr12	124399549	rs1316952	1f
chr5	125338474	rs13169113	4
chr5	179471200	rs13161895	6
chr5	39058355	rs13160161	5
chr5	168899986	rs13156607	6
chr4	123115501	rs13151961	6
chr14	68699593	rs1314913	2a
chr4	27194070	rs13149020	6
chr4	187257575	rs13148903	6

chr4	77412139	rs13146355	5
chr4	109217928	rs13144621	6
chr4	116196305	rs13144478	5
chr4	145506455	rs13141641	5
chr4	156645512	rs13139571	6
chr4	76416386	rs13137105	6
chr4	100011170	rs13132688	5
chr4	94887030	rs13130787	6
chr4	9926966	rs13129697	5
chr4	123218312	rs13119723	6
chr4	1365126	rs13118159	5
chr4	183821661	rs13114435	6
chr4	177542972	rs13111989	5
chr4	1291112	rs13108904	5
chr4	101806531	rs13107740	6
chr3	56798494	rs13100723	5
chr3	46235200	rs13098911	3a
chr3	113290467	rs13092825	5
chr3	53147575	rs13088281	1f
chr3	122014565	rs13083990	6
chr3	27537908	rs13082711	4
chr3	12289799	rs13081389	6
chr3	120425330	rs13077101	6
chr3	126961147	rs13075436	6
chr3	174029044	rs13074924	6
chr3	18706857	rs13073817	6
chr3	66798949	rs13069000	4
chr3	143772246	rs13068298	5
chr3	188584678	rs13067593	6
chr3	156854741	rs13064954	4
chr3	54632546	rs13064588	6
chr20	31531543	rs13045180	3a
chr20	3756596	rs13043330	5
chr20	1924706	rs13042885	3a
chr20	39269073	rs13041247	6
chr20	46425575	rs13038095	6
chr2	190985679	rs13034723	6
chr2	192117237	rs13030978	6
chr2	57934054	rs13026414	5
chr2	236795342	rs13025591	6
chr2	27815509	rs13022873	5
chr2	160069755	rs13022357	5
chr2	20688518	rs13021401	5
chr2	40757790	rs13017846	6
chr2	61164330	rs13017599	5
chr2	202162810	rs13016963	5
chr2	22822158	rs13015955	5

chr2	102971864	rs13015714	6
chr2	181996044	rs13010713	6
chr2	231035744	rs13010639	6
chr2	8530255	rs13008689	5
chr2	5437542	rs13004938	6
chr2	61186828	rs13003464	5
chr2	663482	rs12999373	6
chr7	7302292	rs1299548	6
chr2	234173502	rs12994997	5
chr2	127887984	rs12989701	6
chr2	182323664	rs12988934	5
chr19	2170953	rs12986413	4
chr19	2177192	rs12982744	5
chr19	39731782	rs12980275	6
chr19	39738786	rs12979860	5
chr21	16817050	rs1297265	2b
chr18	57884749	rs12970134	5
chr18	67536495	rs12969657	6
chr18	76591634	rs12967884	6
chr18	57849022	rs12967135	6
chr18	52752016	rs12966547	5
chr8	11698746	rs1296028	1f
chr18	57751013	rs12957347	6
chr5	131995842	rs1295686	4
chr18	57872988	rs12955983	6
chr17	13733805	rs12949531	5
chr17	37912376	rs12946510	1b
chr17	43208120	rs12946454	5
chr6	6743148	rs1294421	5
chr17	40527543	rs12942547	6
chr17	14561015	rs12940030	6
chr17	55866286	rs12938916	5
chr16	11403892	rs12928822	4
chr16	11187782	rs12924729	1f
chr16	20367689	rs12917707	5
chr15	91114375	rs12915189	4
chr15	28365617	rs12913832	5
chr15	67467506	rs12913547	2b
chr15	38986367	rs12912251	1f
chr15	39315357	rs12907914	5
chr15	92883839	rs12905014	6
chr15	78833222	rs12901682	4
chr14	70517182	rs12883884	5
chr14	91442778	rs1286083	5
chr7	9266387	rs1285407	5
chr12	72724033	rs12831974	3a
chr12	4305882	rs12827476	6

chr12	12773520	rs12822507	5
chr12	89328334	rs12821256	4
chr12	49474604	rs12821008	6
chr11	39327958	rs12808199	5
chr11	102733162	rs12808148	5
chr11	124606284	rs12807809	5
chr11	103529631	rs12805875	6
chr5	180595137	rs1279750	4
chr10	127823150	rs1278329	2a
chr10	12328009	rs12779790	6
chr10	96405501	rs12777823	5
chr10	25918878	rs12775535	5
chr10	96405328	rs12772169	5
chr10	99294796	rs12767760	4
chr1	178471221	rs12760731	3a
chr1	216716536	rs12757165	6
chr1	76484013	rs12753569	4
chr1	66169678	rs12753193	3a
chr1	242955562	rs12751297	6
chr1	205676262	rs12748961	5
chr20	57164854	rs127430	5
chr1	10638603	rs12741973	6
chr19	15677709	rs1273516	5
chr1	221551538	rs12733856	6
chr1	8046671	rs12727642	6
chr1	60084515	rs12727131	6
chr1	155108166	rs12726330	4
chr1	16080170	rs12725198	6
chr10	6097282	rs12722495	6
chr1	186870071	rs12720541	5
chr15	99072904	rs12719740	5
chr7	50428444	rs12718598	4
chr7	50428427	rs12718597	3a
chr16	78188737	rs12716852	5
chr16	78187902	rs12716850	6
chr2	88315792	rs12714207	3a
chr2	55081375	rs12713280	6
chr2	46332168	rs12712969	6
chr16	57012378	rs12708980	6
chr16	11179872	rs12708716	1f
chr7	135479572	rs12707249	6
chr7	25901638	rs12700667	4
chr3	169481270	rs12696304	5
chr2	242262985	rs12694997	6
chr2	11255339	rs12692432	6
chrX	106244766	rs12688220	6
chr4	79280692	rs1268789	6

chr8	74274190	rs12679254	6
chr7	21607351	rs12670798	6
chr7	17309278	rs12670403	3a
chr7	96415054	rs12669076	6
chr7	78158471	rs12666870	4
chr7	2004420	rs12666575	5
chr6	21430727	rs12663356	6
chr14	50877982	rs1265879	5
chr5	77291939	rs12655917	6
chr12	111708457	rs1265564	5
chr12	114868137	rs1265507	5
chr5	176794190	rs12654812	4
chr5	74648602	rs12654264	6
chr5	1895828	rs12653946	5
chr6	31155784	rs1265181	1f
chr4	36079593	rs12651329	6
chr6	31118018	rs1265112	5
chr6	31107186	rs1265093	1f
chr4	174515681	rs12646107	6
chr4	88805207	rs12644436	6
chr4	154153999	rs12644284	4
chr3	162131407	rs12636148	6
chr3	16408488	rs12635698	5
chr3	112376307	rs12634229	6
chr11	116681007	rs1263173	1f
chr3	146632163	rs12629805	5
chr3	177095071	rs12629106	5
chr22	40654275	rs12628051	5
chr13	51066622	rs1262778	6
chr20	57790435	rs12625057	5
chr2	173311552	rs12621278	4
chr2	52703589	rs12619788	6
chr2	213824044	rs12619285	4
chr2	240843637	rs12618573	6
chr2	99083891	rs12617721	1f
chr19	19721721	rs12610185	5
chr18	40023781	rs12606301	5
chr18	53855524	rs12604483	5
chr17	2771511	rs12603284	4
chr16	54027970	rs12596210	6
chr15	63290644	rs12595433	4
chr15	45985070	rs12594515	5
chr15	68036851	rs12593813	5
chr15	61060706	rs12591650	6
chr15	33050422	rs1258763	6
chr14	26129565	rs12586774	6
chr12	56439208	rs12580100	3a

chr12	5797100	rs12579350	6
chr11	79077192	rs12576775	5
chr11	2502318	rs12576239	4
chr11	63979642	rs12575642	5
chr10	2424555	rs12571964	6
chr10	109569849	rs12570947	1f
chr1	241878546	rs12569163	6
chr1	22702230	rs12568930	5
chr1	156869046	rs12566888	5
chr1	61041874	rs12565755	5
chr1	11033081	rs12565727	5
chr14	65747758	rs1256531	2a
chr1	201345486	rs12564445	5
chr9	76157699	rs12555078	6
chr9	22842201	rs12554707	6
chr8	112976140	rs12549576	6
chr8	57313905	rs12545109	4
chr8	88868339	rs12543318	5
chr8	60623347	rs12541902	6
chr7	76762950	rs12540771	6
chr7	128717905	rs12537284	6
chr7	131287989	rs12534221	4
chr7	23502973	rs12534093	6
chr7	42325624	rs12532960	6
chr7	135329977	rs12530845	5
chr6	91015038	rs12529935	6
chr5	131784392	rs12521868	6
chr5	180170818	rs12517906	5
chr4	155451758	rs12511469	6
chr4	73645350	rs12507628	5
chr4	74319282	rs12506899	4
chr4	57334111	rs12505749	2b
chr10	81058026	rs1250552	5
chr10	81060316	rs1250550	5
chr10	81032531	rs1250546	5
chr10	81032884	rs1250544	5
chr10	81034669	rs1250542	2b
chr10	81036006	rs1250540	4
chr4	145436323	rs12504628	6
chr4	95514608	rs12500426	6
chr4	9944051	rs12498742	5
chr1	75770624	rs1249675	6
chr3	103397768	rs12485744	6
chr3	56865775	rs12485738	1f
chr22	40652872	rs12484776	6
chr21	38740823	rs12483205	3a
chr21	37013620	rs12483148	5

chr20	56428704	rs12481680	6
chr20	44761484	rs12480534	4
chr2	242502955	rs12479254	4
chr2	39101208	rs12479213	6
chr2	43721507	rs12478601	1f
chr2	239877147	rs12477314	5
chr2	46921284	rs12474201	6
chr6	161333936	rs1247318	6
chr2	142228508	rs12472911	6
chr2	239095421	rs12472274	6
chr2	219908368	rs12470505	6
chr2	43359060	rs12466022	4
chr19	33356890	rs12460876	5
chr18	13366861	rs12456874	5
chr18	40673379	rs12456492	6
chr18	56213389	rs12456021	6
chr10	73849638	rs1245541	4
chr16	58075281	rs12447804	5
chr16	88298123	rs12447690	5
chr14	85654018	rs12436689	2b
chr14	24381514	rs12436436	5
chr14	53837484	rs12434047	6
chr14	95848293	rs12432260	4
chr13	80644617	rs12431307	5
chr13	74742321	rs12429889	5
chr13	36441098	rs12428086	5
chr12	783483	rs12425791	4
chr12	66364508	rs12424086	6
chr12	125033932	rs12423712	2b
chr11	72250122	rs12418204	5
chr11	4563217	rs12416860	6
chr10	120278943	rs12413624	5
chr10	104719095	rs12413409	6
chr10	65315396	rs12411988	1f
chr1	205638221	rs12409639	4
chr11	116520526	rs1240773	6
chr13	51106554	rs1239947	6
chrX	2885722	rs12393627	6
chrX	10231690	rs12388359	6
chr12	28156080	rs12371778	6
chr12	58133255	rs12368653	1f
chr12	57204159	rs12367822	6
chr10	65121564	rs12355784	6
chr9	136723519	rs12344583	5
chr9	139121739	rs12338076	4
chr2	165540799	rs12328675	6
chr19	14139920	rs12327666	5

chr12	83164555	rs12317459	5
chr12	43160743	rs12316797	5
chr17	2125604	rs1231206	5
chr12	51357541	rs12304921	6
chr12	100820084	rs12296850	5
chr11	92920401	rs12296063	5
chr11	2489341	rs12296050	5
chr11	26605330	rs12295638	2b
chr11	79083619	rs12290811	5
chr11	45813580	rs12285276	6
chr11	125179852	rs12284594	5
chr11	116613659	rs12280753	4
chr10	71263263	rs1227969	1f
chr10	71588503	rs1227756	2b
chr11	125241045	rs12273350	4
chr11	116603723	rs12272004	4
chr10	11719009	rs12265836	6
chr10	35554053	rs12261843	6
chr10	55384742	rs12256830	5
chr10	6123494	rs12251307	6
chr10	9363876	rs12247397	6
chr10	35535694	rs12242110	6
chr1	55742836	rs12239436	4
chr1	247601594	rs12239046	1d
chr12	1856952	rs12230440	4
chr12	111414460	rs12229654	6
chr10	81788103	rs12220777	6
chr6	14587002	rs12213875	6
chr6	90996768	rs12212193	4
chr6	475488	rs12210050	6
chr6	26116981	rs12206204	5
chr6	396320	rs12203592	2b
chr6	17699321	rs12199222	4
chr6	32026807	rs12198173	5
chr6	77290798	rs12198063	6
chr6	2565751	rs12195826	4
chr6	31252924	rs12191877	6
chr5	158829526	rs12188300	4
chr5	428235	rs12188164	5
chr6	151957713	rs12173570	5
chr22	37982011	rs12157904	6
chr8	49812200	rs12155623	5
chr7	20994490	rs12155172	5
chr6	32074803	rs12153855	5
chr5	171203437	rs12153391	5
chr2	235210726	rs12151790	5
chr17	7521914	rs12150660	6

chr16	53842907	rs12149832	6
chr14	20957432	rs12147450	4
chr1	89146233	rs12145922	1f
chr1	204572070	rs12143943	5
chr1	162033889	rs12143842	2b
chr1	188383315	rs12142669	6
chr1	19839114	rs12138950	2b
chr1	197781197	rs12134279	5
chr1	63191776	rs12130333	1f
chr1	209727256	rs12130212	6
chr1	145725688	rs12129861	6
chr1	107463642	rs12125971	6
chr1	19386584	rs12123383	5
chr1	146508933	rs12122100	6
chr1	196973182	rs12116643	6
chr16	240279	rs1211375	5
chr6	122158269	rs12110693	5
chr19	3797099	rs12104221	6
chr21	40173527	rs1209950	4
chr11	93093136	rs12098946	5
chr1	107336300	rs12097821	5
chr1	159647987	rs12093699	5
chr2	206150593	rs1207421	6
chr1	2452978	rs12073504	5
chr1	192431997	rs12067906	2b
chr6	11943525	rs1205863	5
chr3	38593392	rs12053903	6
chr2	190607152	rs12053254	6
chr2	81872921	rs12052359	5
chr17	4683034	rs12051548	5
chr16	82663287	rs12051272	4
chr15	72169465	rs12050794	6
chr1	110031187	rs12049330	4
chr1	101331535	rs12048904	6
chr1	10799576	rs12046278	5
chr1	19765517	rs12045440	5
chr1	231844346	rs12044355	6
chr1	231816841	rs12042938	6
chr1	230922950	rs12038826	5
chr1	40064960	rs12037222	2b
chr1	70355980	rs12037173	6
chr1	172898376	rs12035082	4
chr1	207757514	rs12034598	6
chr1	207803594	rs12034383	5
chr1	88625635	rs12032672	6
chr1	249168435	rs12032643	2b
chr1	221617075	rs12032381	6

chr1	162133116	rs12029454	6
chr1	95053352	rs12029080	5
chr1	25775732	rs12027135	6
chr1	117038286	rs12025416	5
chr1	8759553	rs12025126	6
chr1	178491878	rs12023718	6
chr1	231270635	rs12023396	2b
chr1	219651132	rs12022722	6
chrX	152862637	rs12010175	5
chr10	37559579	rs1200821	6
chr9	15283096	rs12003180	6
chr8	122621908	rs11995252	4
chr8	121694649	rs11989782	5
chr8	118827838	rs11989122	5
chr8	119766193	rs11988997	6
chr2	10903411	rs1198872	5
chr8	117641609	rs11987235	4
chr8	1746949	rs11986414	5
chr8	127332656	rs11986011	6
chr1	98552831	rs1198588	5
chr7	38835034	rs11984145	6
chr7	105281187	rs11983798	3a
chr7	152669839	rs11981919	6
chr7	55159348	rs11979158	5
chr7	50466303	rs11978267	1f
chr7	143741579	rs11976180	6
chr7	126437896	rs11971186	6
chr6	41925289	rs11970772	4
chr6	41925303	rs11968166	3a
chr6	105612219	rs11962089	6
chr5	39397131	rs11959928	5
chr5	55001898	rs11958779	5
chr5	169950393	rs11957313	3a
chr5	173695777	rs11952171	6
chr4	167455492	rs11941399	6
chr4	150901756	rs11930273	5
chr3	195928853	rs11924930	6
chr1	92077096	rs1192415	4
chr3	170834558	rs11920719	5
chr3	170717520	rs11920090	4
chr3	60763255	rs11919041	3a
chr3	5188245	rs11918654	6
chr3	195809138	rs11915082	4
chrX	136055294	rs1190739	6
chr2	192587203	rs11903757	6
chr2	54684556	rs11898505	2b
chr2	5664007	rs11894081	6

chr2	234565282	rs11892031	3a
chr2	104808711	rs11890236	6
chr2	166943276	rs11890028	6
chr2	150697147	rs11889862	6
chr2	228481418	rs11889798	5
chr2	27083268	rs11887277	1f
chr2	96841792	rs11886999	5
chr2	206318592	rs11884476	6
chr19	31911128	rs11880316	5
chr19	3159768	rs11880198	5
chr19	10512910	rs11879191	1f
chr17	37813855	rs11869286	5
chr17	59239220	rs11868441	4
chr17	16933403	rs11867934	2b
chr16	11166687	rs11865121	4
chr16	31095170	rs11865038	4
chr16	77427768	rs11864477	5
chr16	84046714	rs11864146	5
chr16	82326804	rs11863065	6
chr16	70505358	rs11861805	5
chr15	78783276	rs11858836	6
chr15	67066562	rs11858577	4
chr15	58712202	rs11857380	5
chr15	29731443	rs11856574	5
chr15	68892988	rs11856323	5
chr15	101875122	rs11855415	5
chr14	25479541	rs11850957	5
chr14	96175977	rs11849538	6
chr14	71507600	rs11848070	6
chr14	22782376	rs11845134	6
chr13	113694508	rs11842874	5
chr13	79410573	rs11838918	6
chr13	73741148	rs11838472	5
chr12	123823545	rs11830103	6
chr11	93704048	rs11825709	5
chr7	2869984	rs1182188	6
chr10	130137104	rs11818629	6
chr1	93148376	rs11810217	6
chr1	103459536	rs11809524	6
chr1	67675515	rs11805303	5
chr1	38721662	rs11802770	4
chr1	233719983	rs11800854	2b
chr1	246661235	rs11800820	6
chr9	98429265	rs11790994	5
chr9	136925662	rs11789898	1f
chr7	72856429	rs1178979	5
chr7	72857048	rs1178977	6

chr9	121359285	rs11789399	5
chr8	24378740	rs11781622	6
chr8	123408090	rs11781551	5
chr8	10683928	rs11776767	6
chr7	86821979	rs11773103	6
chr7	143109138	rs11767557	5
chr7	48817964	rs11767191	6
chr7	69887084	rs11766624	5
chr7	71388849	rs11766496	6
chr7	28391141	rs11765845	4
chr7	65326820	rs11763147	6
chr17	7142850	rs117616209	2b
chr7	99118800	rs11761528	5
chr7	131370038	rs11761231	5
chr10	96058635	rs117607728	5
chr6	89183579	rs11757661	6
chr6	118993631	rs11756438	6
chr6	7118989	rs11755724	6
chr6	90958230	rs11755527	6
chr6	65635922	rs11754641	6
chr6	55473293	rs11754509	6
chr6	133663588	rs11753937	5
chr7	22024039	rs1175000	5
chr5	4029788	rs11748327	6
chr5	150258866	rs11747270	6
chr5	176798305	rs11746443	1b
chr5	131796921	rs11745587	6
chr5	40410583	rs11742570	4
chr5	150277908	rs11741861	5
chr5	156942284	rs11740562	1f
chr5	60950210	rs11738335	5
chr5	32815027	rs1173771	4
chr5	32804527	rs1173766	5
chr4	100713764	rs11735070	6
chr4	6891518	rs11734132	4
chr4	86683559	rs11732231	5
chr4	106463712	rs11730243	6
chr19	55819844	rs1172822	5
chr4	174602047	rs11727767	6
chr4	282498	rs11723261	6
chr1	205244952	rs1172130	4
chr3	135225248	rs11712655	6
chr3	119118795	rs11712165	6
chr3	182821274	rs11711441	4
chr3	5705051	rs11710433	5
chr3	38657898	rs11710077	5
chr3	38633922	rs11708996	5

chr3	123065777	rs11708067	4
chr3	55188272	rs11706236	6
chr2	124983055	rs1170612	6
chr22	40436972	rs11704416	2b
chr20	55683995	rs11699237	4
chr20	3185122	rs11697186	1f
chr20	43371319	rs11696845	5
chr20	44124522	rs11696501	5
chr12	121442669	rs1169313	1f
chr2	228584897	rs11691652	5
chr14	36738360	rs116909374	3a
chr14	36397679	rs1168987	6
chr2	218314252	rs11689435	5
chr2	25887557	rs11684202	2b
chr1	62996837	rs1168013	6
chr7	75173179	rs1167796	6
chr2	3841419	rs11677370	6
chr2	235900170	rs11676855	5
chr2	219010145	rs11676348	1b
chr2	204249398	rs11675251	6
chr19	55383050	rs11672983	6
chr19	41985586	rs11672691	3a
chr19	46172277	rs11671664	1b
chr19	10838485	rs11671653	5
chr19	35559473	rs11671010	4
chr19	3318509	rs11669592	5
chr19	11092138	rs11669133	4
chr19	47268372	rs11668878	4
chr19	11195029	rs11668477	5
chr19	55833663	rs11668344	4
chr19	17590280	rs11666579	5
chr19	17118432	rs11666377	5
chr18	74735134	rs11663697	5
chr18	68153619	rs11663206	6
chr18	19261412	rs11662721	6
chr18	75653522	rs11661856	6
chr18	20223694	rs11661542	5
chr17	4901981	rs11658587	2a
chr17	61763030	rs11658329	1f
chr17	10033678	rs11656696	5
chr17	43795432	rs11655470	6
chr17	27675225	rs11653144	2a
chr6	25870541	rs1165205	6
chr17	32287448	rs11650066	6
chr16	30918486	rs11649653	1f
chr16	792189	rs11648796	6
chr16	90084560	rs11648785	5

chr16	84685508	rs11647936	5
chr16	82642650	rs11646213	6
chr16	62377152	rs11645366	6
chr16	85991704	rs11642873	4
chr16	86381183	rs11641231	6
chr15	83352281	rs11638815	5
chr15	48633152	rs11637235	5
chr15	80432221	rs11634397	6
chr14	103040086	rs11628318	4
chr14	70365923	rs11627546	6
chr14	94972241	rs11627075	5
chr14	47454985	rs11627056	5
chr14	78786076	rs11624704	5
chr14	68986156	rs11624164	2b
chr14	103883632	rs11623869	3a
chr14	104509075	rs11622475	1f
chr13	48436924	rs11620399	6
chr12	6502741	rs11616188	5
chr12	73601131	rs11615274	5
chr12	57792579	rs11613352	6
chr12	118893247	rs11613092	5
chr12	12657512	rs11612508	6
chr12	47639525	rs11610206	4
chr19	45403411	rs1160985	6
chr11	63963946	rs11607165	6
chr11	15355245	rs11605083	3a
chr20	22050502	rs1160312	6
chr11	202855	rs11602954	5
chr11	131325245	rs11601906	6
chr11	28233767	rs11601602	5
chr10	101805441	rs11599750	5
chr10	101861434	rs11597390	1b
chr10	81015895	rs11593576	5
chr1	113313562	rs11590090	4
chr2	128177376	rs1158867	4
chr1	6631430	rs11587438	5
chr8	19912369	rs115849089	6
chr1	200935865	rs11584383	5
chr1	101407518	rs11581062	6
chr16	31368873	rs11574637	5
chr16	67971379	rs11574514	4
chr4	67733856	rs1155865	5
chr14	64680847	rs1152591	5
chr11	78992685	rs1151200	5
chr6	32050757	rs1150754	1f
chr4	180704917	rs11495908	6
chr4	131026487	rs114646238	6

chr12	32261238	rs1144713	2b
chr1	160630142	rs11265461	6
chr1	159700038	rs11265260	6
chr1	153939129	rs11264736	3a
chr1	155151492	rs11264341	4
chr1	155098904	rs11264330	2b
chr15	84580581	rs11259936	5
chr10	15129401	rs11259474	6
chr10	6407736	rs11258317	3a
chr10	12307893	rs11257655	4
chr3	101411977	rs112553552	6
chr10	17130692	rs11254363	4
chr10	566378	rs11252926	5
chr10	1406363	rs11250464	5
chr1	121280612	rs11249433	5
chr1	25297183	rs11249215	4
chr16	163597	rs11248850	1a
chr4	964358	rs11248060	1f
chr4	858331	rs11248051	5
chr1	26650549	rs11247915	6
chr15	51352248	rs1124769	5
chr10	128267639	rs11245052	6
chr3	13857968	rs1124480	6
chr9	135618282	rs11243897	5
chr3	20108545	rs1124376	1f
chr9	135096766	rs11243676	5
chr9	134450072	rs11243437	6
chr6	1535997	rs11242704	5
chr5	137599333	rs11242417	5
chr5	123119564	rs11241713	5
chr1	146560563	rs11239930	6
chr10	45123378	rs11239177	4
chr11	76332209	rs11236809	6
chr11	82723071	rs11233413	4
chr11	62771387	rs11231299	5
chr11	61795585	rs11230874	6
chr2	26228206	rs112288323	5
chr11	68978579	rs11228565	5
chr19	11163600	rs1122608	4
chr11	102691481	rs11225434	5
chr11	130273229	rs11222084	5
chr11	126243951	rs11220462	1f
chr16	53809246	rs1121980	4
chr11	98723215	rs11217223	5
chr11	116782973	rs11216185	5
chr11	114231254	rs11214966	6
chr11	108283160	rs11212617	6

chr11	97574983	rs11212364	6
chr1	68785212	rs11209261	6
chr18	54983553	rs1120787	6
chr1	56850685	rs11206801	6
chr1	149892871	rs11205277	6
chr8	14517293	rs11203649	6
chr21	43836185	rs11203203	5
chr10	90964613	rs11203032	2b
chr10	124220543	rs11200638	5
chr10	123851826	rs11200392	6
chr10	104939214	rs11191593	6
chr10	104906210	rs11191580	1f
chr10	104846177	rs11191548	1f
chr10	102979206	rs11190870	5
chr10	101365312	rs11190179	4
chr10	101292389	rs11190141	4
chr10	101291592	rs11190140	5
chr10	101282199	rs11190134	5
chr1	106430286	rs11184708	6
chr12	72125284	rs11178918	5
chr12	59259627	rs11172782	6
chr12	57527282	rs11172113	4
chr12	56518407	rs11171747	6
chr12	56470624	rs11171739	6
chr12	51155662	rs11169552	3a
chr12	48208367	rs11168249	2b
chr5	141479064	rs11167764	6
chr8	139504269	rs11166827	5
chr1	99348835	rs11166135	6
chr1	80734580	rs11162963	6
chr14	70347347	rs11158820	4
chr14	65240948	rs11158559	4
chr14	63953521	rs11158493	5
chrX	153006494	rs11156606	5
chr6	141169824	rs11155133	5
chr6	139660011	rs11155053	1f
chr6	135739354	rs11154801	1d
chr6	121748541	rs11154022	6
chr6	118667521	rs11153730	6
chr16	31334235	rs11150610	1f
chr16	30482493	rs11150589	2b
chr9	139860263	rs11145951	5
chr9	77499795	rs11144134	5
chr9	76158681	rs11143609	5
chr9	74887702	rs11143230	6
chr9	72998331	rs11142387	5
chr9	90235793	rs11141915	4

chr9	84372740	rs11139399	5
chr8	145043542	rs11136341	5
chr5	155202527	rs11134474	5
chr5	8983720	rs11134338	6
chr5	6834166	rs11134178	6
chr4	190641169	rs11132733	5
chr3	50352199	rs11130248	6
chr2	68652579	rs11126185	5
chr2	113978939	rs11123170	1f
chr2	121701288	rs11122834	5
chr1	9411468	rs11121382	6
chr1	9408958	rs11121380	5
chr10	94462881	rs1111875	5
chr1	221028507	rs11118620	6
chr1	219743718	rs11118346	3a
chr1	219657162	rs11118316	5
chr16	86019270	rs11117432	4
chr12	79775247	rs11114027	3a
chr12	96807916	rs11108495	6
chr12	93978503	rs11107116	6
chr12	88829293	rs11104870	5
chr4	96467502	rs11097470	3a
chr4	95146134	rs11097407	6
chr4	71329489	rs1109501	6
chr20	46779234	rs11086243	5
chr19	13001546	rs11085824	4
chr19	34322136	rs11084753	6
chr19	29736341	rs11083866	5
chr19	47207653	rs11083846	5
chr18	21365852	rs11082762	6
chr18	20720972	rs11082304	5
chr18	10899027	rs11080466	1f
chr17	44949099	rs11079740	1f
chr17	38064404	rs11078927	6
chr17	2145192	rs11078884	3a
chr17	7469228	rs11078697	5
chr17	1618362	rs11078597	4
chr7	2041431	rs1107592	5
chr16	15148645	rs11075253	5
chr16	10633164	rs11074889	5
chr15	76293970	rs11072566	6
chr15	69650052	rs11072089	6
chr15	63341995	rs11071720	2b
chr15	62433961	rs11071657	4
chr12	119163078	rs11069062	6
chr12	57809455	rs1106766	6
chr12	115094259	rs11067228	2b

chr5	173352762	rs1106693	3a
chr7	131453664	rs1106684	5
chr7	131453524	rs1106683	5
chr12	113365620	rs11066453	6
chr12	112871371	rs11066301	6
chr12	112168008	rs11066015	6
chr12	112072423	rs11065987	3a
chr12	110704486	rs11065611	4
chr12	120562973	rs11065028	1f
chr12	2091256	rs11062040	3a
chr12	130630934	rs11060736	6
chr12	9855957	rs11052552	6
chr12	23946145	rs11047102	6
chr11	8252852	rs110419	5
chr11	8243797	rs11041816	6
chr11	46379441	rs11038871	4
chr11	43728329	rs11037575	5
chr11	5225634	rs11036238	6
chr11	38288592	rs11034653	5
chr11	20378123	rs11025523	1f
chr11	17008604	rs11024102	2b
chr11	16917218	rs11024074	5
chr11	95992128	rs11021499	5
chr11	89875436	rs11018874	6
chr10	133330025	rs11018023	5
chr10	131500939	rs11016883	5
chr10	25111909	rs11014249	6
chr10	18708797	rs11014166	6
chr10	21830103	rs11012732	5
chr10	36060308	rs11010290	5
chr10	35295430	rs11010067	6
chr10	33294774	rs11009175	4
chr10	29289030	rs11007350	5
chr10	28504943	rs11006923	4
chr10	28150934	rs11006747	5
chr10	60555577	rs11006263	5
chr10	78315223	rs11001819	5
chr10	72332439	rs10999409	5
chr10	70939800	rs10998624	1d
chr10	65026857	rs10995485	5
chr10	64655912	rs10995356	6
chr10	64398465	rs10995251	6
chr10	64278681	rs10995190	4
chr10	64223382	rs10995170	6
chr10	62279123	rs10994397	6
chr10	62181127	rs10994338	2b
chr9	93633239	rs10993738	5

chr9	95559504	rs10992471	6
chr9	98376698	rs10988802	6
chr9	132370359	rs10988449	1f
chr9	119333682	rs10983238	6
chr9	117088063	rs10982156	5
chr9	4426630	rs10974531	6
chr9	29783988	rs10969375	6
chr9	27309658	rs10967875	6
chr9	22115104	rs10965235	5
chr9	14562313	rs10961577	5
chr8	40053604	rs10958605	3a
chr8	57095807	rs10958476	5
chr8	54410207	rs10958369	6
chr8	61194169	rs10957125	5
chr7	112911409	rs10953730	5
chr7	109152710	rs10953615	6
chr7	104503812	rs10953454	6
chr7	20647014	rs10950821	3a
chr6	44967489	rs10948197	6
chr6	30093363	rs10947055	6
chr6	26233386	rs10946808	4
chr6	20661033	rs10946398	5
chr6	170425520	rs10946292	5
chr5	45197778	rs10941694	6
chr5	57130817	rs10940579	3a
chr4	47563447	rs10938494	2b
chr3	191000807	rs10937470	4
chr3	189383182	rs10937405	5
chr3	188289696	rs10937355	4
chr3	187713717	rs10937329	6
chr3	186650789	rs10937275	4
chr3	137447484	rs10935268	6
chr3	134233091	rs10935120	6
chr3	128038372	rs10934853	5
chr2	221720087	rs10932886	5
chr2	191538561	rs10931468	5
chr2	183685561	rs10931041	5
chr2	12568995	rs10929808	5
chr5	10467701	rs1092913	1f
chr2	130468365	rs10928927	5
chr2	147372023	rs10928302	5
chr2	144428607	rs10928195	6
chr1	244173871	rs10927101	5
chr1	245733607	rs10924245	6
chr1	169099482	rs10919071	6
chr1	161915500	rs10918270	5
chr1	19843353	rs10917468	6

chr1	171949749	rs10914144	6
chr1	177913518	rs10913469	6
chr1	183081193	rs10911251	6
chr1	233643855	rs10910200	6
chr1	155126947	rs10908458	6
chr10	12438782	rs10906142	5
chr10	10073543	rs10905651	6
chr10	7089015	rs10905099	3a
chr1	25768936	rs10903129	1f
chr10	127699935	rs10901513	6
chr11	78095372	rs10899489	6
chr11	63592620	rs10897449	4
chr11	58339123	rs10896794	5
chr11	66551001	rs10896135	6
chr11	106330161	rs10895959	5
chr11	129698855	rs10894147	5
chr11	125178402	rs10893366	5
chr11	118611780	rs10892279	4
chr1	63118195	rs10889353	1f
chr1	62950857	rs10889332	6
chr1	56060950	rs10888935	6
chr1	152537953	rs10888501	4
chr10	89443309	rs10887741	5
chr10	121149402	rs10886471	5
chr10	116327549	rs10885582	6
chr10	101795360	rs10883437	5
chr10	101287763	rs10883365	4
chr12	72414562	rs10879357	5
chr12	40352995	rs10877840	5
chr12	58062666	rs10876993	5
chr12	56401084	rs10876864	1f
chr12	54712307	rs10876550	6
chr12	53731890	rs10876432	4
chr12	49676009	rs10875943	5
chr5	140700488	rs10875595	2b
chr1	93323970	rs10874746	6
chr1	76772327	rs10873876	6
chr18	57851762	rs10871777	6
chr16	74472695	rs10871290	3a
chr3	52718279	rs10865974	6
chr1	212237797	rs10863936	5
chr1	209988046	rs10863790	5
chr1	222245257	rs10863681	5
chr12	108767332	rs10861905	6
chr12	103912505	rs10861032	6
chr12	95711875	rs10859871	4
chr9	138275775	rs10858396	5

chr10	49985109	rs10857636	5
chr17	2143459	rs10852932	4
chr1	84761347	rs1085093	6
chr12	115381739	rs10850409	6
chr12	111333621	rs10849915	6
chr12	121938184	rs10849893	4
chr12	6435570	rs10849441	2b
chr12	4425121	rs10849033	5
chr12	3814237	rs10848911	5
chr12	2882543	rs10848704	5
chr12	125312424	rs10846744	4
chr6	161089816	rs1084651	6
chr12	12834893	rs10845606	5
chr12	28744640	rs10843215	6
chr12	20521653	rs10841496	4
chr12	20063967	rs10841397	5
chr11	48098279	rs10838801	6
chr11	47663048	rs10838738	6
chr11	47312891	rs10838687	1b
chr11	47275063	rs10838681	1f
chr11	23044591	rs10833905	5
chr11	94667963	rs10831284	6
chr11	92708709	rs10830963	3a
chr11	92698426	rs10830962	6
chr11	92681012	rs10830956	6
chr11	88891113	rs10830228	6
chr10	132747886	rs10829848	6
chr12	67628830	rs1082714	6
chr10	75421207	rs10824026	2b
chr10	64251976	rs10822013	4
chr10	63785088	rs10821944	4
chr10	63723576	rs10821936	5
chr9	97713458	rs10821415	2b
chr9	104223232	rs10819937	3a
chr9	126446777	rs10818854	4
chr9	99539137	rs10816533	6
chr9	8235632	rs10815798	5
chr9	4845519	rs10815094	6
chr9	4293149	rs10814916	5
chr9	33180361	rs10813960	6
chr9	331489	rs10813766	4
chr9	27533983	rs10812610	6
chr9	17895104	rs10810865	3a
chr9	1202370	rs10809650	6
chr8	65640259	rs10808739	5
chr7	132189688	rs10808265	3a
chr6	90926611	rs10806425	4

chr4	13914372	rs10805321	3a
chr1	196660260	rs10801555	6
chr1	191559355	rs10801047	6
chr1	161472157	rs10800309	6
chr1	165409094	rs10800098	5
chr1	21820989	rs10799701	1f
chr1	33983018	rs10798959	5
chr7	41470092	rs1079866	6
chr1	173309712	rs10798269	6
chr1	183852913	rs10797919	3a
chr1	2501337	rs10797432	1f
chr10	4151120	rs10795130	5
chr10	1156164	rs10794720	2b
chr11	85838807	rs10792830	6
chr11	61746290	rs10792320	1f
chr1	151895092	rs10788819	6
chr10	87740752	rs10788473	6
chr10	123033548	rs10788160	6
chr10	100300181	rs10786436	6
chr10	98135504	rs10786284	2b
chr12	66343809	rs10784502	5
chr12	66160970	rs10784496	6
chr19	31801129	rs1078373	6
chr1	85268242	rs10782529	6
chr16	30942624	rs10782001	5
chr9	139269337	rs10781500	6
chr9	79408143	rs10781380	2b
chr14	67975821	rs1077989	5
chr12	103495150	rs10778213	6
chr12	97689767	rs10777845	6
chr12	91473797	rs10777288	5
chr9	138029700	rs10776934	4
chr1	112107668	rs10776733	5
chr19	6668971	rs1077667	4
chr10	49735562	rs10776612	6
chr12	111910218	rs10774625	6
chr12	4368351	rs10774214	6
chr12	349297	rs10774021	6
chr12	132014205	rs10773920	6
chr12	9380858	rs10771431	5
chr12	20230638	rs10770612	5
chr11	8484088	rs10769908	6
chr11	7367094	rs10769780	6
chr11	32895663	rs10767971	3a
chr11	27725985	rs10767664	6
chr11	95866699	rs10765792	5
chr22	18439957	rs1076540	5

chr10	28388149	rs10763642	5
chr10	65274926	rs10761779	2b
chr10	65027609	rs10761731	4
chr9	135776033	rs1076160	6
chr9	102723691	rs10760706	6
chr9	4856876	rs10758658	6
chr9	4292082	rs10758593	5
chr9	32755027	rs10758161	5
chr9	22124476	rs10757278	4
chr9	22088259	rs10757272	4
chr9	22072263	rs10757269	6
chr12	69827657	rs10748128	5
chr1	99524046	rs10747502	6
chr9	78841400	rs10746997	6
chr1	232246026	rs10746514	5
chr12	114714265	rs10744816	5
chr12	129324937	rs10744391	6
chr12	10521388	rs10743889	1f
chr11	19599970	rs10741780	4
chr10	56790968	rs10740609	6
chr9	2691185	rs10738760	5
chr1	196679454	rs10737680	5
chr1	189723622	rs10737562	5
chr5	125319455	rs1073203	6
chr5	141522999	rs1062158	1f
chr12	6683286	rs1057510	6
chr7	25871108	rs1055144	5
chr17	73872947	rs1055129	4
chr2	219934347	rs1052483	6
chr16	86011336	rs10521318	5
chr17	13559079	rs10521233	6
chr17	13539803	rs10521232	6
chr15	96141866	rs10520789	6
chr15	36313964	rs10520045	6
chr4	149635024	rs10519980	5
chr15	49221590	rs10519201	5
chr15	62001131	rs10519131	6
chr15	40700021	rs10518693	1f
chr4	68064439	rs10518025	6
chr4	60748828	rs10517480	6
chr4	26085510	rs10517086	5
chr4	42453252	rs10517025	6
chr4	106688903	rs10516526	5
chr4	99337880	rs10516430	6
chr5	145038830	rs10515552	5
chr5	71716927	rs10515148	6
chr5	65739438	rs10514995	5

chr3	61413813	rs10514718	6
chr5	90424278	rs10514345	5
chr3	187543341	rs10513821	6
chr3	152535364	rs10513432	5
chr5	9623621	rs10513025	4
chr5	3558537	rs10512697	5
chr3	124340221	rs10512627	5
chr9	98259702	rs10512248	6
chr3	60289841	rs10510837	5
chr3	60123521	rs10510829	6
chr3	30346967	rs10510634	6
chr10	126438018	rs10510138	5
chr10	123625189	rs10510102	1f
chr10	90023032	rs10509540	3a
chr10	78157571	rs10509373	4
chr10	28025770	rs10508727	5
chr10	18388433	rs10508558	5
chr10	16299950	rs10508503	6
chr10	8972017	rs10508372	6
chr12	80496922	rs10506821	6
chr12	54687231	rs10506328	6
chr12	2056362	rs10505725	5
chr8	128407442	rs10505477	5
chr8	73778641	rs10504543	6
chr8	23534017	rs10503733	5
chr8	19847689	rs10503669	6
chr8	4214178	rs10503256	6
chr8	4180843	rs10503253	6
chr18	55454376	rs10503019	5
chr18	35198295	rs10502675	6
chr11	99493172	rs10501920	6
chr11	84417845	rs10501570	6
chr11	47293798	rs10501320	2b
chr16	72756100	rs10500569	2b
chr19	33750313	rs10500264	6
chr6	138002636	rs10499194	5
chr6	22743402	rs10498712	6
chr14	93103308	rs10498635	6
chr14	64899054	rs10498514	5
chr2	221899443	rs10498091	5
chr2	192914361	rs10497721	5
chr2	134005568	rs10496702	4
chr2	123126478	rs10496584	6
chr2	81751123	rs10496262	6
chr2	43806917	rs10495903	5
chr2	6694518	rs10495537	6
chr1	216632947	rs10495024	6

chr1	162085684	rs10494366	5
chr1	110352476	rs10494112	6
chr1	107830857	rs10494067	5
chr1	66310864	rs10493389	5
chr1	63591128	rs10493340	6
chr1	10353111	rs10492972	6
chr13	40807482	rs10492681	5
chr13	108816224	rs10492664	5
chr8	129246414	rs10492294	5
chr12	6580581	rs10492096	1f
chr10	117512587	rs10490919	6
chr3	62036723	rs10490775	6
chr1	234583617	rs10489896	6
chr1	159196764	rs10489849	6
chr1	189589338	rs10489759	4
chr1	230304987	rs10489615	6
chr4	13794415	rs10489087	5
chr7	128594182	rs10488631	3a
chr7	133335175	rs10488172	5
chr7	36952020	rs10488023	6
chr7	93713520	rs10487245	6
chr7	41478607	rs10486715	5
chr7	29184488	rs10486607	6
chr7	27976562	rs10486567	4
chr7	26892439	rs10486483	6
chr7	7938595	rs10486201	6
chr6	32665419	rs10484561	6
chr6	26031612	rs10484434	4
chr6	9451196	rs10484246	6
chr14	98642571	rs10484128	5
chr14	73756298	rs10483853	2b
chr14	65914866	rs10483776	6
chr14	61072874	rs10483727	6
chr8	124831833	rs10481151	5
chr5	173648437	rs10475598	6
chr5	32888817	rs10472828	5
chr15	58678511	rs10468017	5
chr12	40767361	rs10467147	6
chr12	9876090	rs10466829	5
chr10	76703005	rs10466033	6
chr5	56104307	rs10461617	6
chr18	2948028	rs10460009	5
chr10	4655564	rs10458787	6
chr1	70921172	rs10458561	3a
chr6	161010117	rs10455872	6
chr6	66598193	rs10455590	6
chr3	139073010	rs1044826	4

chr2	146105253	rs10445672	6
chr17	79261808	rs10445407	1f
chr16	88840461	rs10445033	4
chr1	99453286	rs10443196	6
chr6	20688120	rs10440833	6
chr5	40490789	rs10440635	4
chr4	118093136	rs10433903	5
chr2	150147277	rs10432496	5
chr19	29908106	rs10425935	5
chr19	46182303	rs10423928	5
chr19	54604821	rs10423754	6
chr19	18817902	rs10423674	1f
chr19	17762889	rs10416963	5
chr19	33532299	rs10411210	2a
chr19	13245967	rs10408126	2b
chr19	3944239	rs10406174	5
chr19	22608373	rs10404998	5
chr19	53284134	rs10404486	6
chr19	19407717	rs10401969	1f
chr12	121236257	rs1039302	3a
chr6	166155456	rs1039002	6
chr6	151933174	rs1038304	6
chr15	74012436	rs1038094	5
chr18	56752053	rs1037757	5
chr3	64886109	rs1036797	5
chr15	48914774	rs1036476	5
chr19	7199802	rs1035942	4
chr17	47564011	rs1035050	4
chr22	19984276	rs1034566	4
chr6	383545	rs1033180	4
chr19	54797847	rs103294	6
chr2	105910512	rs1030877	5
chr6	32856380	rs1029296	6
chr1	183170947	rs1028771	6
chr7	1285194	rs10277115	4
chr5	91893791	rs1027643	6
chr7	1273844	rs10275044	5
chr7	157387440	rs10274279	5
chr7	155768748	rs10267337	5
chr7	130900120	rs10265216	6
chr7	7848911	rs10259199	6
chr7	7268430	rs10259085	5
chr7	1039002	rs10256972	1f
chr7	50751089	rs10248619	6
chr7	88481441	rs10248351	6
chr7	116346602	rs10243024	4
chr2	204721751	rs1024161	6

chr4	131137760	rs1024020	6
chr7	43910881	rs10232743	4
chr1	11899032	rs1023252	1f
chr7	112628372	rs10229603	5
chr7	157294937	rs10227331	5
chr7	37938421	rs10226308	6
chr7	151415040	rs10224002	5
chr2	165577163	rs10221833	5
chr6	32857418	rs10214886	5
chr2	65208073	rs10211524	6
chr2	234158838	rs10210302	5
chr2	161757128	rs10208407	3a
chr2	127852020	rs10207628	5
chr2	111849658	rs10207392	5
chr2	240687288	rs10207060	5
chr2	176784137	rs1020410	6
chr5	55560026	rs1020388	6
chr2	238270893	rs10202497	5
chr2	60500143	rs10202231	5
chr2	105897739	rs1020064	6
chr2	2519512	rs10199521	5
chr2	12964496	rs10198628	5
chr2	152253917	rs10197940	6
chr2	151725791	rs10195263	2b
chr2	47240011	rs10194115	5
chr17	54278714	rs1019238	6
chr2	161380887	rs10192369	6
chr2	149387967	rs10191411	6
chr2	23124557	rs10189050	5
chr2	85794296	rs10187424	6
chr2	219514758	rs10187066	1f
chr2	238822330	rs10185142	5
chr2	158780203	rs10183640	3a
chr2	182007799	rs1018326	6
chr2	61224258	rs10181042	6
chr1	218705813	rs1018040	6
chr2	235960892	rs10174126	6
chr2	139278921	rs10170310	6
chr2	150457623	rs10170236	5
chr5	131744573	rs1016988	6
chr2	198881667	rs1016883	6
chr2	234825092	rs10166942	6
chr8	128093296	rs1016343	3a
chr11	4516575	rs10160399	3a
chr10	71099887	rs10159477	4
chr7	22756462	rs10155981	6
chr15	70048156	rs10152591	2b

chr3	46451679	rs1015164	6
chr14	56418118	rs10151037	5
chr14	79936963	rs10150332	5
chr22	39332622	rs1014971	5
chr15	95165579	rs1014922	5
chr14	79945161	rs10146997	5
chr14	23840032	rs10137082	5
chr18	31420009	rs1013696	5
chr14	106232584	rs10136766	5
chr14	95410528	rs10133840	5
chr14	21261732	rs10131141	5
chr21	35698389	rs1013063	5
chr11	18632983	rs10128711	5
chr1	230295788	rs10127775	5
chr9	81083896	rs10125737	6
chr9	97444037	rs10125337	4
chr9	114798036	rs10125054	5
chr9	35269818	rs10121009	5
chr9	108440541	rs10120372	5
chr9	96631133	rs10114408	6
chr2	144690116	rs1011397	6
chr8	20336572	rs10111661	6
chr2	26782620	rs1011108	4
chr8	8722377	rs10108954	5
chr8	123973067	rs10108684	5
chr8	61876043	rs10104895	5
chr8	61105198	rs10098647	6
chr8	19830920	rs10096633	6
chr8	71386903	rs10091374	6
chr8	29360304	rs10091038	5
chr8	128532136	rs10090154	2b
chr8	60178720	rs10089517	5
chr8	124765701	rs10088262	5
chr8	129543948	rs10088218	5
chr6	109626964	rs1008084	4
chr5	78756777	rs10078095	3b
chr5	128722695	rs10077875	6
chr11	46849359	rs1007738	1f
chr5	106982559	rs10074258	6
chr9	113662680	rs1007000	5
chr5	1279789	rs10069690	5
chr21	15845051	rs1006899	6
chr5	99342046	rs10067427	6
chr5	55438850	rs10065637	3a
chr5	32748636	rs10061804	5
chr5	148904091	rs10058728	5
chr5	127653021	rs10057405	5

chr5	91518720	rs10055544	6
chr5	32000482	rs10054504	6
chr5	56666499	rs10052004	5
chr4	87754418	rs10050311	6
chr17	64236317	rs10048158	5
chr11	116722040	rs10047462	5
chr6	32854696	rs10046257	4
chr10	104594506	rs1004467	5
chr11	2170142	rs1004446	5
chr5	148706298	rs10044242	5
chr5	63033717	rs10042968	6
chr21	38491094	rs1003719	6
chr5	150458145	rs10036748	3a
chr4	112611749	rs10034228	6
chr4	110659066	rs10033900	6
chr4	111720760	rs10033464	6
chr4	95643956	rs10027628	5
chr4	95856680	rs10021303	6
chr4	110964361	rs10012953	5
chr22	26403598	rs1001021	6
chr11	61655304	rs1000778	5
chr4	79589044	rs10007186	2a
chr4	142005572	rs10007052	6
chr7	30937177	rs1000597	2b
chr13	64141912	rs1000589	6
chr4	4719493	rs1000579	4
chr4	105844272	rs10005603	6
chr5	150240075	rs1000113	4

Table S2. Relocated GWAS SNPs to Ensembl genes

GWAS SNPID	Chr	Coordinate	Relocated region	Relocated Ensembl gene	Associated disease/trait	Original RefSeq region	PubmedID
rs9331888	8	27468862	UTR5	ENSG00000120885	Alzheimer's disease	intronic	19734903
rs2395185	6	32433167	ncRNA intronic	ENSG00000196301	Ulcerative colitis;Lung cancer;Hodgkin's lymphoma	intergenic	23143601, 22286212, 19915573, 19122664
rs6903608	6	32428285	ncRNA intronic	ENSG00000196301	Nodular sclerosis Hodgkin lymphoma;Hodgkin's lymphoma	intergenic	22086417, 21037568
rs12627933	22	32027449	intronic	ENSG00000241878	Intelligence	upstream	22449649
rs9621305	22	32049917	intronic	ENSG00000241878	Intelligence	intergenic	22449649
rs6088735	20	33745676	intronic	ENSG00000088298	Hemostatic factors and hematological phenotypes	intergenic	22443383
rs6060278	20	33753262	intronic	ENSG00000088298	Hemostatic factors and hematological phenotypes	intergenic	22443383
rs2348114	2	201582193	ncRNA exonic	ENSG00000244301	Intelligence	ncRNA intronic	22449649
rs9308945	2	34284852	ncRNA intronic	ENSG00000203386	Hypertension (young onset)	intergenic	19421330
rs6711736	2	34284984	ncRNA intronic	ENSG00000203386	Hypertension (young onset)	intergenic	19421330
rs6729869	2	34285194	ncRNA intronic	ENSG00000203386	Hypertension (young onset)	intergenic	19421330
rs10495809	2	34305257	ncRNA intronic	ENSG00000203386	Hypertension (young onset)	intergenic	19421330
rs7960483	12	45925755	ncRNA intronic	ENSG00000257657	Hypertension	intergenic	21626137
rs10785581	12	45930973	ncRNA intronic	ENSG00000257657	Hypertension	intergenic	21626137
rs11234027	11	71234107	UTR3	ENSG00000172890	Vitamin D levels	intergenic	20418485
rs999556	5	150473674	promoter	ENSG00000145901	Myopia (pathological)	intergenic	23049088
rs9995093	4	89220944	ncRNA intronic	ENSG00000246375	Bipolar disorder	intergenic	22925353
rs9984974	21	37809377	ncRNA intronic	ENSG00000230479	Myopia (pathological)	intergenic	23049088
rs9982601	21	35599128	ncRNA intronic	ENSG00000214955	Myocardial infarction (early	intergenic	21378990, 19198609

					onset);Coronary heart disease		
rs998124	18	42725662	ncRNA intronic	ENSG00000267101	Prostate cancer (gene x gene interaction)	intergenic	22219177
rs9978142	21	35652239	ncRNA intronic	ENSG00000214955	Pulmonary function	intergenic	21946350
rs9977253	21	26645083	ncRNA intronic	ENSG00000222042	Non-alcoholic fatty liver disease histology (other)	intergenic	20708005
rs9938149	16	88331640	ncRNA intronic	ENSG00000261273	Central corneal thickness;Cornea l structure	intergenic	22814818, 23291589
rs993804	3	25112171	ncRNA intronic	ENSG00000237838	Bipolar disorder and schizophrenia	intergenic	20889312
rs9923231	16	31107689	promote r	ENSG00000167397	Warfarin maintenance dose	intergenic	20833655, 19300499
rs9918668	7	93691744	ncRNA intronic	ENSG00000236453	Aging	intergenic	22773346
rs9913711	17	70098161	ncRNA intronic	ENSG00000234899	Liver enzyme levels (gamma-glutamy l transferase)	intergenic	22001757
rs9899891	17	14629746	ncRNA intronic	ENSG00000226130	Visceral adipose tissue/subcutane ous adipose tissue ratio	intergenic	22589738
rs9878522	3	165125159	ncRNA intronic	ENSG00000244128	Response to amphetamines	intergenic	22952603
rs9876781	3	48487338	ncRNA intronic	ENSG00000244380	Longevity	upstream	20834067
rs9874556	3	3356174	ncRNA intronic	ENSG00000223727	Pancreatic cancer	intergenic	23180869
rs9870146	3	117156255	intronic	ENSG00000185565	Obesity-related traits	intergenic	23251661
rs9866825	3	8250790	ncRNA intronic	ENSG00000227110	QT interval	intergenic	23166209
rs9866141	3	156950579	ncRNA intronic	ENSG00000243176	Diabetic retinopathy;Diab etic retinopathy	intergenic	21441570
rs9860340	3	87783976	ncRNA intronic	ENSG00000239572	Electroencephal ographic traits in alcoholism	intergenic	22554406
rs9844666	3	135974216	UTR5	ENSG00000114054	Height	intronic	20881960
rs984440	8	139077728	ncRNA	ENSG00000253288,	Obesity-related	intergenic	23251661

			intronic	ENSG00000253988	traits		
rs9837561	3	3650239	ncRNA intronic	ENSG00000223727	Response to amphetamines	intergenic	22952603
rs9835812	3	3375784	ncRNA intronic	ENSG00000223727	Immune response to smallpox vaccine (IL-6)	intergenic	22542470
rs9824150	3	162834301	ncRNA intronic	ENSG00000241168	Capecitabine sensitivity	intergenic	22864933
rs9815663	3	3614887	ncRNA intronic	ENSG00000223727	Asthma (childhood onset)	intergenic	22560479
rs9811423	3	112822322	ncRNA intronic	ENSG00000240057	Alzheimer's disease	intergenic	22005930
rs9810890	3	128652553	intronic	ENSG00000114656, ENSG00000187695	Dental caries	intergenic	23064961
rs9807989	2	102971200	intronic	ENSG00000115604	Asthma	intergenic	22561531
rs9807334	18	48524161	intronic	ENSG00000141646, ENSG00000267699	Immune reponse to smallpox (secreted IFN-alpha)	intergenic	22610502
rs9805786	13	24658356	intronic	ENSG00000182957	Depression and alcohol dependence	intergenic	22064162
rs9803659	1	167156500	ncRNA intronic	ENSG00000231605	Liver enzyme levels	intergenic	18940312
rs977396	8	105992696	intronic	ENSG00000253350	Response to antipsychotic treatment	intergenic	20195266
rs974819	11	103660567	ncRNA intronic	ENSG00000254987	Coronary heart disease	intergenic	21378988
rs972275	6	127391844	intronic	ENSG00000260527	Iron status biomarkers	intergenic	19084217
rs969088	5	26389262	ncRNA intronic	ENSG00000249099	Response to platinum-based chemotherapy in non-small-cell lung cancer	intergenic	22872573
rs9686661	5	55861786	intronic	ENSG00000225940	Triglycerides	intergenic	20686565
rs9657451	8	138905296	ncRNA intronic	ENSG00000253288, ENSG00000254361	Cognitive performance	intergenic	20125193
rs9653442	2	100825367	ncRNA intronic	ENSG00000232084	Type 1 diabetes	intergenic	17554260
rs9652490	15	77963887	intronic	ENSG00000169783	Essential tremor	intergenic	19182806
rs9650199	8	61346197	ncRNA intronic	ENSG00000251396	Response to amphetamines	intergenic	22952603
rs9642880	8	128718068	ncRNA intronic	ENSG00000249375	Urinary bladder cancer;Bladder	intergenic	20972438, 20348956,

					cancer		18794855
rs964184	11	116648917	UTR3	ENSG00000109917	Vitamin E levels;Triglycerides;Response to Vitamin E supplementation;Phospholipid levels (plasma);Metabolite levels;Metabolic syndrome;Lipoprotein-associated phospholipase A2 activity and mass;LDL cholesterol;Hypertriglyceridemia;HDL cholesterol;Coronary heart disease;"Cholesterol, total"	downstream	22916037, 22437554, 22399527, 22359512, 22003152, 21729881, 21378990, 20864672, 20686565, 20657596, 19060906
rs9635542	16	5001380	intronic	ENSG00000118898	Lung cancer-asbestos exposure interaction	intergenic	22637743
rs9619497	22	35371248	ncRNA intronic	ENSG00000227895	Metabolite levels (HVA-5-HIAA Factor score)	intergenic	23319000
rs96067	1	36571920	intronic	ENSG00000171812	Corneal structure	intergenic	23291589
rs9604529	13	114622597	ncRNA exonic	ENSG00000229373	Response to tocilizumab in rheumatoid arthritis	intergenic	22491018
rs958994	5	165063884	ncRNA intronic	ENSG00000253693	Carotid atherosclerosis in HIV infection	intergenic	20009918
rs958546	13	46833717	intronic	ENSG00000173988	Atrial fibrillation	intergenic	17903304
rs956225	8	122909687	ncRNA intronic	ENSG00000254018	Alzheimer's disease	intergenic	22159054
rs9533799	13	44808174	ncRNA intronic	ENSG00000227258, ENSG00000230731	Amyotrophic lateral sclerosis	intergenic	22959728
rs9512900	13	28429738	ncRNA intronic	ENSG00000247381	Attention deficit hyperactivity disorder and	intergenic	18951430

					conduct disorder		
rs9507041	13	23744379	ncRNA intronic	ENSG00000229483	Cannabis dependence	intergenic	21668797
rs9488363	6	114591986	ncRNA intronic	ENSG00000228624	Optic disc size (rim)	intergenic	20395239
rs9485372	6	149608874	intronic	ENSG00000055208	Breast cancer	intergenic	22383897
rs9469003	6	31407828	ncRNA intronic	ENSG00000206337	Stevens-Johnson syndrome and toxic epidermal necrolysis (SJS-TEN)	intergenic	21801394
rs9426935	1	153769400	ncRNA intronic	ENSG00000231827	Lentiform nucleus volume;Lentiform nucleus volume	intergenic	22903471
rs9399137	6	135419018	intronic	ENSG00000112339	Platelet counts;Hematology traits;HbA2 levels;F-cell distribution;Mean corpuscular volume;Mean corpuscular hemoglobin	intergenic	23043469, 22139419, 19853236, 17767159, 23263863
rs939207	4	69167671	ncRNA intronic	ENSG00000249531	Obesity-related traits	intergenic	23251661
rs9388489	6	126698719	intronic	ENSG00000260527	Type 1 diabetes	intergenic	19430480
rs9377063	6	147934947	intronic	ENSG00000203727	Obesity-related traits	intergenic	23251661
rs9375674	6	130216510	intronic	ENSG00000260527	Renal sinus fat;Renal sinus fat	intergenic	22044751
rs9373124	6	135423209	intronic	ENSG00000112339	White blood cell types;Other erythrocyte phenotypes	intergenic	21738478, 19862010
rs9368699	6	31802541	UTR5	ENSG00000204387	HIV-1 control	upstream	20041166
rs9364813	6	166128638	ncRNA intronic	ENSG00000256956	Economic and political preferences (immigration/crime)	intergenic	22566634
rs9321637	6	138266685	ncRNA intronic	ENSG00000226004, ENSG00000229922	Cardiac Troponin-T levels	intergenic	23247143
rs931608	19	22614122	intronic	ENSG00000197360	Response to statin therapy	intergenic	23064961, 22331829

					(LDL-C);Dental caries		
rs9312648	4	55241581	promoter	ENSG00000248518	Response to amphetamines	intergenic	22952603
rs9305354	21	29475196	ncRNA exonic	ENSG00000232079	Urinary albumin excretion	intergenic	17903292
rs930421	2	42981239	UTR3	ENSG00000057935	Attention deficit hyperactivity disorder	intergenic	18821565
rs9303196	17	5907443	intronic	ENSG00000179314	Non-small cell lung cancer	intergenic	20876614
rs9296949	6	14597982	ncRNA intronic	ENSG00000229646	Metabolite levels (HVA/MHPG ratio)	intergenic	23319000
rs9286879	1	172862234	ncRNA intronic	ENSG00000224228	Crohn's disease	intergenic	18587394, 23128233
rs9272535	6	32606756	exonic	ENSG00000196735	Chronic lymphocytic leukemia	intronic	21131588
rs9272346	6	32604372	intronic	ENSG00000196735	Type 1 diabetes;Asthma	upstream	18978792, 17554300, 23181788
rs9272219	6	32602269	intronic	ENSG00000196735	Schizophrenia;Rheumatoid arthritis	intergenic	21653640, 19571809
rs9272105	6	32599999	intronic	ENSG00000196735	Response to interferon beta therapy;Hepatocellular carcinoma	intergenic	22807686, 21502966
rs9271858	6	32595223	promoter	ENSG00000196735	Leishmaniasis (visceral)	intergenic	23291585
rs9268923	6	32432835	ncRNA intronic	ENSG00000196301	Ulcerative colitis	intergenic	20228798
rs9268905	6	32432077	ncRNA intronic	ENSG00000196301	Cystic fibrosis severity	intergenic	21602797
rs9268877	6	32431147	ncRNA intronic	ENSG00000196301	Ulcerative colitis	intergenic	19915572, 18836448
rs9268853	6	32429643	ncRNA intronic	ENSG00000196301	Ulcerative colitis;Rheumatoid arthritis;Lymphoma	intergenic	21653640, 21297633, 23349640
rs9267663	6	31867253	intronic	ENSG00000166278	Economic and political preferences (environmentalis	downstream	22566634

					m)		
rs9257809	6	29356331	intronic	ENSG00000243729	Barrett's esophagus	intergenic	22961001
rs925255	2	28614794	ncRNA intronic	ENSG00000229951	Inflammatory bowel disease	upstream	23128233
rs923175	11	12072503	ncRNA intronic	ENSG00000254486	Response to taxane treatment (docetaxel)	intergenic	23006423
rs922948	3	69442637	intronic	ENSG00000114541	Hip geometry	intergenic	17903296
rs921720	8	126534671	ncRNA intronic	ENSG00000253111	Obesity-related traits;Inflammatory bowel disease	intergenic	23251661, 23128233
rs918959	2	181513729	ncRNA intronic	ENSG00000225258	Systemic lupus erythematosus	intergenic	21408207
rs911119	20	23612737	ncRNA exonic	ENSG00000270001	Chronic kidney disease	intergenic	20383146
rs908327	1	235092600	promoter	ENSG00000237520	Immune reponse to smallpox (secreted IL-2)	intergenic	22610502
rs900400	3	156798775	promoter	ENSG00000241544	Birth weight	downstream	20372150, 23202124
rs895636	2	45188353	ncRNA exonic	ENSG00000225156	Metabolite levels	intergenic	21909109
rs894177	3	142894411	promoter	ENSG00000244171	Non-alcoholic fatty liver disease histology (other)	intergenic	20708005
rs893001	18	67516845	intronic	ENSG00000150637	Mean platelet volume	downstream	19820697
rs891835	8	130491752	ncRNA intronic	ENSG00000229140	Glioma	intergenic	19578367
rs890835	5	175956271	exonic	ENSG00000146083	Menopause (age at onset)	intronic	22267201
rs886424	6	30782002	ncRNA exonic	ENSG00000214894	Bipolar disorder and schizophrenia	intergenic	22688191
rs883924	9	93181531	ncRNA intronic	ENSG00000231107	Hepatitis C induced liver fibrosis	intergenic	22841784
rs882632	2	29280761	UTR3	ENSG00000189350	Major depressive disorder	intergenic	20125088
rs879882	6	31139452	intronic	ENSG00000204531	Height	intergenic	20881960
rs876537	1	159674933	ncRNA exonic	ENSG00000223603	C-reactive protein	intergenic	21647738
rs873549	1	222271767	ncRNA intronic	ENSG00000236230	Keloid	intergenic	20711176

rs872690	X	37854727	intronic	ENSG00000250349	Erectile dysfunction and prostate cancer treatment	intergenic	20932654
rs871606	4	54799245	intronic	ENSG00000145216	Blood pressure	intergenic	21909110
rs870288	16	5585852	ncRNA intronic	ENSG00000260411	Subcutaneous adipose tissue;Phospholipid levels (plasma)	intergenic	22589738, 22359512
rs857228	14	98670158	ncRNA intronic	ENSG00000259097	Sexual dysfunction (SSRI/SNRI-related)	intergenic	22445761
rs854572	7	94954696	intronic	ENSG00000005421	Paraoxonase activity	upstream	22982463
rs848353	7	108548660	promoter	ENSG00000229603	Smoking behavior	intergenic	22006218
rs840616	2	188196469	ncRNA intronic	ENSG00000224063	Coronary heart disease	intergenic	21378988
rs838880	12	125261593	UTR3	ENSG00000073060	HDL cholesterol	downstream	20686565
rs836132	11	34555191	ncRNA intronic	ENSG00000255271	β2-Glycoprotein I (β2-GPI) plasma levels	intergenic	23279374
rs835367	1	59762468	UTR5	ENSG00000172456	Obesity-related traits	upstream	23251661
rs830884	5	52020396	ncRNA intronic	ENSG00000248898	Response to platinum-based agents	intergenic	22020760
rs8127571	21	47165429	intronic	ENSG00000183570	Immune reponse to smallpox (secreted IFN-alpha)	intergenic	22610502
rs8112449	19	10520064	intronic	ENSG00000105401	Multiple sclerosis	intergenic	21833088
rs8102683	19	41363765	intronic	ENSG00000268797	Smoking behavior	intergenic	23049750
rs8102476	19	38735613	intronic	ENSG00000167642	Prostate cancer	intergenic	19767754
rs8101143	19	21956136	ncRNA intronic	ENSG00000268184	Capecitabine sensitivity	intergenic	22864933
rs8097348	18	1595021	ncRNA intronic	ENSG00000266602	Exercise (leisure time)	intergenic	19727025
rs8077059	17	55823552	promoter	ENSG00000166329	Sex hormone-binding globulin levels	intergenic	22675492
rs8076739	17	27714587	ncRNA intronic	ENSG00000264808, ENSG00000266111	Mean platelet volume	intergenic	22139419

rs8070473	17	33843512	intronic	ENSG00000205045	Depression (quantitative trait)	intergenic	20800221
rs806794	6	26200677	UTR3	ENSG00000197846	Height	downstream	20881960
rs8062326	16	19156016	intronic	ENSG00000261427	Alcohol dependence	intergenic	21876473
rs8049607	16	11691753	intronic	ENSG00000189067	QT interval	intergenic	23166209, 19305409, 19305408
rs8039808	15	49939846	intronic	ENSG00000140285	Response to antidepressant treatment	intergenic	22041458
rs802734	6	128278798	intronic	ENSG00000260527	Multiple sclerosis;Celiac disease	intergenic	21833088, 20190752
rs8023580	15	96708291	ncRNA intronic	ENSG00000247809	Sex hormone-bindin g globulin levels	intergenic	22829776
rs8017161	14	103563195	ncRNA intronic	ENSG00000259444	Primary biliary cirrhosis	intergenic	21399635
rs8016947	14	35832666	ncRNA intronic	ENSG00000258860	Psoriasis	intergenic	20953190
rs8016620	14	97393685	intronic	ENSG00000100749	Obesity-related traits	intergenic	23251661
rs8013477	14	49534425	ncRNA intronic	ENSG00000258868	Visceral adipose tissue/subcutane ous adipose tissue ratio	intergenic	22589738
rs800586	8	116813905	intronic	ENSG00000104447	Response to tocilizumab in rheumatoid arthritis	intergenic	22491018
rs7984869	13	86690527	ncRNA intronic	ENSG00000232252	Economic and political preferences (fairness)	intergenic	22566634
rs7971536	12	102373788	intronic	ENSG00000136048	Height	intergenic	20881960
rs7965584	12	90305779	ncRNA intronic	ENSG00000258216	Magnesium levels	intergenic	20700443
rs7964120	12	74087808	ncRNA intronic	ENSG00000258109	Obesity-related traits	intergenic	23251661
rs7963521	12	73828956	ncRNA intronic	ENSG00000258109	Chemerin levels	intergenic	20237162
rs7961581	12	71663102	intronic	ENSG00000127324	Type 2 diabetes	intergenic	18372903
rs7956193	12	113491784	promote r	ENSG00000242461	Visceral adipose tissue adjusted for BMI	intergenic	22589738
rs7950726	11	5225447	promote	ENSG00000224091	HbA2 levels	intergenic	23043469

			r				
rs7927997	11	76301375	promoter	ENSG00000254755	Crohn's disease	intergenic	21102463
rs7927894	11	76301316	promoter	ENSG00000254755	Crohn's disease;Atopic dermatitis	intergenic	19349984, 18587394
rs790116	3	122380923	ncRNA exonic	ENSG00000243918	Response to angiotensin II receptor blocker therapy (opposite direction w/ diuretic therapy)	intergenic	22566498
rs789560	12	70331827	ncRNA intronic	ENSG00000257815	Attention deficit hyperactivity disorder and conduct disorder	intergenic	18951430
rs7864204	9	71229179	ncRNA intronic	ENSG00000226337	Body mass index (interaction)	intergenic	23192594
rs785422	15	30173885	intronic	ENSG00000104067	Corneal structure	intergenic	23291589
rs7846606	8	77507613	ncRNA intronic	ENSG00000253661	Orofacial clefts	intergenic	22863734
rs7844723	8	122908503	ncRNA intronic	ENSG00000254018	Hemostatic factors and hematological phenotypes	intergenic	17903294
rs7838490	8	89585048	ncRNA intronic	ENSG00000253553	Body mass index and cholesterol (psychopharmacological treatment)	intergenic	22417934
rs7827290	8	142300315	ncRNA intronic	ENSG00000254197	Bipolar disorder	intergenic	21926972
rs7821565	8	52216762	ncRNA intronic	ENSG00000253664	Tuberculosis	intergenic	20694014
rs7816345	8	36846109	promoter	ENSG00000243503	Breast size	intergenic	22747683
rs7816032	8	19786891	intronic	ENSG00000175445	Hyperactive-impulsive symptoms	intergenic	18821565
rs7815944	8	129427518	ncRNA intronic	ENSG00000254275	Atopic dermatitis	intergenic	23042114
rs7814403	8	36459454	ncRNA intronic	ENSG00000253363	Obesity-related traits	intergenic	23251661
rs7808424	7	117067822	intronic	ENSG00000154438	Coronary heart disease	upstream	21378990
rs7800244	7	47779969	intronic	ENSG00000136273	Hepatitis C induced liver	intergenic	22841784

					fibrosis		
rs7780752	7	93241640	intronic	ENSG00000127928	Birth weight	intergenic	23202124
rs7776054	6	135418916	intronic	ENSG00000112339	Mean corpuscular hemoglobin	intergenic	19862010
rs7775698	6	135418635	intronic	ENSG00000112339	Red blood cell traits;Hematology traits;Mean corpuscular volume;Mean corpuscular hemoglobin	intergenic	20927387, 19853236, 23263863
rs7772131	6	30795171	ncRNA intronic	ENSG00000214894	Response to angiotensin II receptor blocker therapy	intergenic	22566498
rs7770731	6	14598820	ncRNA intronic	ENSG00000229646	Working memory	intergenic	21107309
rs776488	2	165890733	ncRNA intronic	ENSG00000236283	Common traits (Other)	intergenic	20585627
rs7756521	6	30848253	intronic	ENSG00000204580	HIV-1 control	intergenic	20041166
rs7739264	6	19785588	ncRNA intronic	ENSG00000226786	Endometriosis	intergenic	23104006
rs7735699	5	79099464	ncRNA intronic	ENSG00000250258	Bipolar disorder and schizophrenia	intergenic	20889312
rs7727102	5	4720585	ncRNA intronic	ENSG00000248973	Bipolar disorder and schizophrenia	intergenic	20889312
rs7717572	5	66833257	ncRNA intronic	ENSG00000249364	Prostate cancer (gene x gene interaction)	intergenic	22219177
rs7714584	5	150270420	intronic	ENSG00000237693	Crohn's disease	intergenic	21102463
rs7705924	5	101946798	ncRNA intronic	ENSG00000250682, ENSG00000250958	Crohn's disease	intergenic	22412388
rs7703051	5	74625487	ncRNA intronic	ENSG00000247372	LDL cholesterol	intergenic	18802019
rs7702331	5	72551134	ncRNA intronic	ENSG00000249743	Crohn's disease	intergenic	21102463, 23128233
rs7694379	4	88186509	ncRNA intronic	ENSG00000250572	Platelet counts	intergenic	22139419
rs76884941	3	111201785	ncRNA intronic	ENSG00000239311, ENSG00000241177	Chronic obstructive pulmonary disease	intergenic	21685187
rs7686660	4	144003159	ncRNA intronic	ENSG00000250326	Asthma	intergenic	21804548
rs7686384	4	189584353	ncRNA	ENSG00000249378	Obesity-related	intergenic	23251661

			intronic		traits		
rs768048	18	50285398	UTR3	ENSG00000187323	Alcoholism (alcohol use disorder factor score)	intronic	21529783
rs7679673	4	106061534	ncRNA intronic	ENSG00000251259	Prostate cancer	intergenic	19767753
rs7676999	4	116934079	ncRNA intronic	ENSG00000248173	Age-related macular degeneration	intergenic	23326517
rs7671266	4	10056376	intronic	ENSG00000109667	Cardiovascular disease risk factors	intergenic	21943158
rs7671189	4	11674661	ncRNA intronic	ENSG00000249631	Obesity-related traits	intergenic	23251661
rs7665939	4	190123318	ncRNA intronic	ENSG00000251310	Amyotrophic lateral sclerosis	intergenic	22959728
rs7665590	4	99796784	UTR3	ENSG00000151247	Primary biliary cirrhosis	intergenic	23000144
rs7662358	4	189590859	ncRNA intronic	ENSG00000249378	Cognitive performance	intergenic	20125193
rs7659062	4	189587079	ncRNA intronic	ENSG00000249378	Cognitive performance	intergenic	20125193
rs7656416	4	1254535	ncRNA intronic	ENSG00000196810	Type 2 diabetes	intergenic	22456796
rs7646881	3	158453279	ncRNA intronic	ENSG00000240207	Tetralogy of Fallot	intergenic	23297363
rs7638110	3	138903985	intronic	ENSG00000175110	Obesity and blood pressure	intergenic	22013104
rs7617219	3	148977314	ncRNA intronic	ENSG00000240216	Subcutaneous adipose tissue	intergenic	22589738
rs7616215	3	46205686	intronic	ENSG00000183625	Behcet's disease	intergenic	23291587
rs7612209	3	177596989	ncRNA intronic	ENSG00000231574	Cognitive performance	intergenic	19734545
rs7602460	2	182261869	ncRNA intronic	ENSG00000234663	Atrioventricular conduction	intergenic	21041692
rs7600502	2	22297003	ncRNA intronic	ENSG00000231200	C-reactive protein and white blood cell count	intergenic	22788528
rs7600259	2	35093293	ncRNA intronic	ENSG00000226994	Response to amphetamines	intergenic	22952603
rs7590720	2	216898658	intronic	ENSG00000115425, ENSG00000118242	Alcohol dependence	intergenic	19581569
rs7587205	2	39843197	ncRNA intronic	ENSG00000231312	Response to angiotensin II receptor blocker therapy	intergenic	22566498

					(opposite direction w/ diuretic therapy)		
rs7584262	2	42250549	ncRNA intronic	ENSG00000226398	Bone mineral density	intergenic	22504420
rs7583236	2	70182805	ncRNA exonic	ENSG00000239072	Obesity-related traits	intergenic	23251661
rs75825892	16	4257313	exonic	ENSG00000185739	Obesity-related traits	intronic	23251661
rs7578035	2	99382892	ncRNA intronic	ENSG00000226791	Bipolar disorder	intergenic	21926972
rs7571971	2	88895351	UTR5	ENSG00000172071	Progressive supranuclear palsy	intronic	21685912
rs7570469	2	79709354	intronic	ENSG00000066032	Response to antipsychotic treatment	intergenic	20195266
rs7561273	2	24247514	intronic	ENSG00000205639	Quantitative traits	downstream	19197348
rs7555668	1	112856570	ncRNA intronic	ENSG00000231246	Cognitive performance	intergenic	20125193
rs7552393	1	84254551	ncRNA intronic	ENSG00000233008	Select biomarker traits	intergenic	17903293
rs7550918	1	247675559	intronic	ENSG00000169224	Platelet counts	intergenic	22139419
rs7539409	1	84254735	ncRNA intronic	ENSG00000233008	Alzheimer's disease	intergenic	20061627
rs7529925	1	199007208	ncRNA intronic	ENSG00000235492	Red blood cell traits	intergenic	23222517
rs7526035	1	35289039	intronic	ENSG00000163866	Formal thought disorder in schizophrenia	intergenic	22648509
rs7524694	1	104019631	ncRNA intronic	ENSG00000224613	Obesity-related traits	intergenic	23251661
rs7517810	1	172853460	ncRNA intronic	ENSG00000224228	Crohn's disease	intergenic	21102463
rs746961	19	31099890	intronic	ENSG00000198597	Select biomarker traits	intergenic	17903293
rs746630	14	83255205	ncRNA exonic	ENSG00000258683	Obesity-related traits	intergenic	23251661
rs744016	22	27042106	promoter	ENSG00000225783, ENSG00000226912	RR interval (heart rate)	intergenic	20031603
rs743793	22	33561746	ncRNA intronic	ENSG00000232073	Visceral adipose tissue/subcutaneous adipose tissue ratio	intergenic	22589738
rs743777	22	37551607	intronic	ENSG00000100385	Type 1 diabetes autoantibodies;Rheumatoid	intergenic	21829393, 21653640, 17554300

					arthritis		
rs7396835	11	116684028	ncRNA exonic	ENSG00000236267	Quantitative traits	intergenic	19197348
rs739310	22	27051299	ncRNA intronic	ENSG00000225783	Obesity-related traits	intergenic	23251661
rs7387468	8	94358098	ncRNA intronic	ENSG00000246662	Temperament	downstream	22832960
rs7360412	20	2800801	UTR3	ENSG00000198326	White matter integrity	intergenic	23218918
rs7356884	6	22753047	ncRNA intronic	ENSG00000233358	Obesity-related traits	intergenic	23251661
rs734999	1	2513216	ncRNA intronic	ENSG00000228037	Ulcerative colitis	intergenic	21297633
rs733175	4	10050141	intronic	ENSG00000109667	Alzheimer's disease	intergenic	22005930
rs73317122	17	25320889	ncRNA intronic	ENSG00000264734	Response to amphetamines	intergenic	22952603
rs7307780	12	76220618	ncRNA intronic	ENSG00000258077	Sudden cardiac arrest	intergenic	21658281
rs730570	14	101142890	ncRNA intronic	ENSG00000258717	Type 2 diabetes	intergenic	21573907
rs730566	3	48487048	ncRNA intronic	ENSG00000244380	Prion diseases	intergenic	22210626
rs7302017	12	63004583	ncRNA intronic	ENSG00000257354	Waist circumference	intergenic	20966902
rs7299940	12	131390754	promoter	ENSG00000206850	Panic disorder	intergenic	19165232
rs7296418	12	123457619	UTR5	ENSG00000150967	Platelet counts	intergenic	21507922
rs72960926	6	75158266	ncRNA intronic	ENSG00000223786	Metabolite levels (MHPG)	intergenic	23319000
rs72829446	17	7552123	intronic	ENSG00000129244	Androgen levels	intergenic	22936694
rs72823592	17	46123004	ncRNA exonic	ENSG00000263412	Epilepsy (generalized)	intergenic	22949513
rs727979	6	149593920	intronic	ENSG00000055208	Hemostatic factors and hematological phenotypes	intergenic	17903294
rs727153	4	155654421	intronic	ENSG00000121207	Alzheimer's disease	intergenic	18823527
rs72669744	1	56116505	ncRNA intronic	ENSG00000234810	Lipid metabolism phenotypes	intergenic	22286219
rs7260598	19	24222786	ncRNA intronic	ENSG00000268362	Response to taxane treatment (placlitaxel)	intergenic	23006423
rs7258249	19	8271721	intronic	ENSG00000090661	Sphingolipid levels	intergenic	22359512, 19798445
rs7255066	19	45146103	ncRNA	ENSG00000266903	Multiple	upstream	21833088

			intronic		sclerosis		
rs7253363	19	11682495	intronic	ENSG00000198551	Multiple sclerosis (severity)	intergenic	19010793
rs724950	4	128342120	ncRNA intronic	ENSG00000248491	Obesity-related traits	intergenic	23251661
rs724016	3	141105570	UTR5	ENSG00000177311	Height	intronic	20881960, 18391950, 18193045
rs7217319	17	38924	ncRNA intronic	ENSG00000262836	AIDS progression	intergenic	21502085
rs7204371	16	67693797	exonic	ENSG00000102977	Obesity-related traits	intronic	23251661
rs7191888	16	73581058	ncRNA intronic	ENSG00000260848	Multiple sclerosis (severity)	intergenic	19010793
rs7191700	16	11406803	intronic	ENSG00000175643	Multiple sclerosis	intergenic	22190364
rs7188445	16	79734987	ncRNA intronic	ENSG00000260876	Urate levels	intergenic	23263486
rs7186852	16	30635659	ncRNA exonic	ENSG00000261680	Systemic lupus erythematosus	intergenic	19838193
rs718545	14	30489623	ncRNA intronic	ENSG00000248975	Obesity-related traits	intergenic	23251661
rs718314	12	26453283	ncRNA intronic	ENSG00000255750, ENSG00000256234	Waist-hip ratio;Renal cell carcinoma	intergenic	22010048, 20935629
rs7181753	15	96844727	ncRNA intronic	ENSG00000247809	Electroencephalographic traits in alcoholism	intergenic	22554406
rs7175404	15	94036688	ncRNA intronic	ENSG00000257060	Attention deficit hyperactivity disorder	intergenic	18839057
rs7159888	14	65758642	ncRNA intronic	ENSG00000258760	N-glycan levels	intergenic	21203500
rs7156960	14	76703351	ncRNA intronic	ENSG00000258454	Acute lymphoblastic leukemia (childhood)	intergenic	22076464
rs7152623	14	98588321	ncRNA intronic	ENSG00000259097	Aortic stiffness	intergenic	22068335
rs7141238	14	61734640	intronic	ENSG00000027075	Obesity-related traits	intergenic	23251661
rs713875	22	30592487	ncRNA intronic	ENSG00000225676	Crohn's disease	intergenic	21102463
rs7137869	12	119989646	ncRNA intronic	ENSG00000248636	Aging traits	intergenic	17903295
rs7136259	12	90081188	intronic	ENSG00000070961	Coronary heart	intergenic	22751097

					disease		
rs7134599	12	68500075	ncRNA intronic	ENSG00000255733	Ulcerative colitis;Inflammatory bowel disease	intergenic	21297633, 23128233
rs71327718	3	125592857	ncRNA intronic	ENSG00000241278, ENSG00000241288	Metabolite levels (HVA/5-HIAA ratio)	intergenic	23319000
rs7129220	11	10350538	intronic	ENSG00000133805	Systolic blood pressure;Diastolic blood pressure;Blood pressure	intergenic	21909115, 21909110
rs7121446	11	121954657	ncRNA intronic	ENSG00000255248	Cardiovascular disease risk factors	intergenic	20838585
rs712022	11	22843155	UTR3	ENSG00000198168	Dialysis-related mortality	downstream	21546767
rs7117858	11	15694462	ncRNA intronic	ENSG00000254695	Bone mineral density (hip)	intergenic	19801982
rs711355	15	30193468	intronic	ENSG00000104067	Response to antipsychotic treatment	intergenic	23241943
rs7108738	11	15710084	ncRNA intronic	ENSG00000254695	Bone mineral density	intergenic	22504420
rs7105934	11	69239741	promoter	ENSG00000255980	Renal cell carcinoma	intergenic	21131975
rs7105881	11	112456221	ncRNA intronic	ENSG00000254968	Response to antipsychotic treatment	intergenic	20195266
rs7085142	10	122939374	ncRNA intronic	ENSG00000227143	Visceral fat	intergenic	22589738
rs7081678	10	31990623	ncRNA intronic	ENSG00000237797	Waist-hip ratio	intergenic	20935629
rs7043482	9	85135915	ncRNA intronic	ENSG00000228430	Insulin resistance/response	intergenic	21901158
rs7014346	8	128424792	ncRNA intronic	ENSG00000246228	Colorectal cancer	intergenic	18372901
rs6990255	8	34126948	ncRNA intronic	ENSG00000253108	Bipolar disorder	intergenic	19416921
rs6987702	8	126504726	ncRNA intronic	ENSG00000253111	LDL cholesterol;"Cholesterol, total"	intergenic	19060911
rs6984305	8	9178268	ncRNA intronic	ENSG00000254235	Liver enzyme levels (alkaline phosphatase)	intergenic	22001757

rs6983267	8	128413305	ncRNA intronic	ENSG00000246228	Prostate cancer;Colorectal cancer	intergenic	21743057, 21242260, 18372905, 18264097, 18264096, 17618284, 17401363, 23266556
rs6951258	7	37757556	intronic	ENSG00000086289, ENSG00000187037	Obesity-related traits	intergenic	23251661
rs694739	11	64097233	promoter	ENSG00000236935	Crohn's disease;Alopecia areata	intergenic	21102463, 20596022
rs6941421	6	15089151	ncRNA intronic	ENSG00000234261	Multiple sclerosis (severity)	intergenic	19010793
rs6938239	6	34683635	ncRNA exonic	ENSG00000186328	Height	intergenic	20881960
rs6924995	6	16161425	ncRNA exonic	ENSG00000218073	Response to statin therapy (LDL-C)	intergenic	22331829
rs6912405	6	74871231	ncRNA intronic	ENSG00000223786	Obesity-related traits	intergenic	23251661
rs6907728	6	132228769	ncRNA intronic	ENSG00000236673	Obesity-related traits	intergenic	23251661
rs6890853	5	35852311	promoter	ENSG00000168685	Primary biliary cirrhosis	intergenic	23000144
rs6867983	5	55854153	intronic	ENSG00000225940	Triglycerides	intergenic	20864672
rs6846071	4	102402215	intronic	ENSG00000153064	C-reactive protein and white blood cell count	intergenic	22788528
rs6844339	4	14144208	ncRNA intronic	ENSG00000251679	Obesity-related traits	intergenic	23251661
rs6843082	4	111718067	ncRNA exonic	ENSG00000249519	Stroke (ischemic);Atrial fibrillation	intergenic	23041239, 20173747
rs6834483	4	35983111	ncRNA intronic	ENSG00000251160	Obesity-related traits	intergenic	23251661
rs6809854	3	18784423	ncRNA intronic	ENSG00000228956	Psoriasis	intergenic	20953190
rs6798928	3	151579873	ncRNA intronic	ENSG00000242908	Immunoglobulin A;Immunoglobulin A	intergenic	20694011
rs6782299	3	180550702	intronic	ENSG00000145075	Schizophrenia	intergenic	19571811
rs6781182	3	34212166	ncRNA intronic	ENSG00000226320	Visceral adipose tissue adjusted for BMI	intergenic	22589738

rs6778194	3	153184656	ncRNA intronic	ENSG00000240456	Immune reponse to smallpox (secreted IFN-alpha)	intergenic	22610502
rs6775745	3	131813900	intronic	ENSG00000196353	Neutrophil count	intergenic	21507922
rs6774852	3	196078427	UTR3	ENSG00000163960	Obesity-related traits	intergenic	23251661
rs6774280	3	6255997	ncRNA intronic	ENSG00000226022	Response to tocilizumab in rheumatoid arthritis	intergenic	22491018
rs67418890	1	161540857	intronic	ENSG00000203747	Lipid metabolism phenotypes	intergenic	22286219
rs6740462	2	65667272	ncRNA intronic	ENSG00000204929	Inflammatory bowel disease	intergenic	23128233
rs6735786	2	103770238	ncRNA intronic	ENSG00000229209	Bone mineral density (hip)	intergenic	19079262
rs6733379	2	34480075	ncRNA intronic	ENSG00000203386	Attention deficit hyperactivity disorder and conduct disorder	intergenic	18951430
rs6721654	2	121301911	promoter	ENSG00000237614	Age-related macular degeneration	intergenic	23326517
rs6720394	2	111989372	ncRNA intronic	ENSG00000172965	Primary sclerosing cholangitis	intergenic	21151127
rs6718520	2	43325570	ncRNA intronic	ENSG00000230587	Multiple sclerosis	intergenic	22190364
rs6716455	2	151126403	ncRNA intronic	ENSG00000230645	Alcoholism (alcohol use disorder factor score);Alcoholism (alcohol dependence factor score)	intergenic	21529783
rs6710823	2	135592381	ncRNA intronic	ENSG00000224043	Parkinson's disease	intergenic	21292315
rs6708166	2	30526780	intronic	ENSG00000213626	vWF and FVIII levels	intergenic	21810271
rs6705628	2	74208362	ncRNA exonic	ENSG00000237883	Systemic lupus erythematosus	intergenic	23273568
rs6699417	1	89123443	ncRNA intronic	ENSG00000237505	Height	intergenic	20881960
rs6693882	1	96145968	ncRNA intronic	ENSG00000228971	Pain	intergenic	19207018
rs6687840	1	159342439	ncRNA	ENSG00000228560	Chemerin levels	intergenic	20237162

			intronic				
rs6651252	8	129567181	ncRNA intronic	ENSG00000254275	Crohn's disease	intergenic	21102463, 23128233
rs6607284	17	36026335	ncRNA intronic	ENSG00000267668	Bipolar disorder and schizophrenia	intergenic	20889312
rs6604026	1	93303603	ncRNA exonic	ENSG00000251795	Multiple sclerosis	intronic	19525955, 17660530
rs6603109	19	7424528	intronic	ENSG00000263264	Sexual dysfunction (SSRI/SNRI-related)	intergenic	22445761
rs6600671	1	121200490	ncRNA intronic	ENSG00000227082	Hip geometry	intergenic	17903296
rs6592284	11	86055901	UTR3	ENSG00000149196	Cognitive performance	intronic	19734545
rs6586513	1	17216331	promoter	ENSG00000238142	Allergic rhinitis	intergenic	22036096
rs6585436	10	119210375	ncRNA intronic	ENSG00000258114	Cardiac repolarization	intergenic	22683750
rs6584283	10	101290301	ncRNA intronic	ENSG00000257582	Ulcerative colitis	intergenic	21297633, 20228798, 19915572
rs6574988	14	88559992	ncRNA intronic	ENSG00000258867	Bipolar disorder	intergenic	21254220
rs6574433	14	78786159	intronic	ENSG00000021645	Cognitive performance	intergenic	20125193
rs6560517	9	79038170	intronic	ENSG00000187210	Dialysis-related mortality	intergenic	21546767
rs6556756	5	163889280	ncRNA intronic	ENSG00000241956, ENSG00000253236	Breast cancer	intergenic	17903305
rs6546886	2	74245777	intronic	ENSG00000187605	Dialysis-related mortality	intergenic	21546767
rs6543833	2	34631032	ncRNA intronic	ENSG00000226785	Ovarian reserve	intergenic	22116950
rs6503659	17	39897264	intronic	ENSG00000173801	Esophageal cancer (squamous cell)	intergenic	22960999
rs6503525	17	38095174	ncRNA intronic	ENSG00000264968	Asthma	intergenic	21150878
rs650258	11	60832282	ncRNA intronic	ENSG00000256733	Multiple sclerosis	intergenic	21833088
rs6496932	15	85825567	ncRNA intronic	ENSG00000218052	Central corneal thickness;Corneal structure	intergenic	20719862, 23291589
rs6486986	12	19801595	intronic	ENSG00000139154	Cognitive performance	intergenic	19734545
rs6484218	11	10390581	intronic	ENSG00000133805	Schizophrenia,	intergenic	20713499

					bipolar disorder and depression (combined)		
rs6444931	3	170163474	intronic	ENSG00000013297	Bipolar disorder and schizophrenia	intergenic	20889312
rs6442925	3	5013876	ncRNA intronic	ENSG00000235831	Corneal structure	intergenic	21979947
rs6442522	3	15440556	intronic	ENSG00000206562	Uric acid levels	intergenic	18759275
rs6441975	3	46428190	ncRNA intronic	ENSG00000223552	AIDS progression	intergenic	21502085
rs6441961	3	46352384	ncRNA exonic	ENSG00000236736	Celiac disease	intergenic	18311140
rs644148	19	44970935	promoter	ENSG00000176761	Personality dimensions	intergenic	18957941
rs6441286	3	159728878	ncRNA intronic	ENSG00000244040	Primary biliary cirrhosis	intergenic	20639880, 19458352
rs6438424	3	117574822	ncRNA intronic	ENSG00000239268	Menarche (age at onset)	intergenic	21102462
rs6431731	2	15863002	intronic	ENSG00000231031	Chronic kidney disease	intergenic	22479191
rs6430538	2	135539967	ncRNA intronic	ENSG00000224043	Parkinson's disease	intergenic	22451204
rs6428370	1	196844593	intronic	ENSG00000080910	Acute lymphoblastic leukemia (childhood)	intergenic	19684603
rs62314947	4	75502487	ncRNA intronic	ENSG00000249942	Breast size	intergenic	22747683
rs62057608	16	34682193	ncRNA exonic	ENSG00000214581	Obesity-related traits	intergenic	23251661
rs61744862	17	17068182	exonic	ENSG00000133030	Obesity-related traits	intronic	23251661
rs6088765	20	33799280	intronic	ENSG00000088298	Ulcerative colitis	intergenic	23128233
rs6085920	20	7180056	ncRNA intronic	ENSG00000228888	Uric acid levels	intergenic	18759275
rs6045676	20	1941171	ncRNA intronic	ENSG00000233896	Aortic root size	intergenic	21223598
rs6027511	20	58898209	ncRNA intronic	ENSG00000228340	Lentiform nucleus volume;Lentiform nucleus volume	downstream	22903471
rs601339	12	123174743	ncRNA intronic	ENSG00000256249	Adiponectin levels	intergenic	22479202
rs600550	11	59997666	intronic	ENSG00000214787	Lipoprotein-associated phospholipase	intergenic	23118302

					A2 activity and mass		
rs593982	11	65513107	promoter	ENSG00000255557	Atopic dermatitis	intergenic	23042114
rs5934953	X	11048692	ncRNA intronic	ENSG00000234129	Cognitive performance	intergenic	19734545
rs5934683	X	9751474	intronic	ENSG00000101850	Colorectal cancer	intergenic	22634755
rs58263042	5	95521777	ncRNA intronic	ENSG00000250551, ENSG00000251314	Breast size	intergenic	22747683
rs5767992	22	48188253	ncRNA intronic	ENSG00000224271	Obesity-related traits	intergenic	23251661
rs5762311	22	28090207	ncRNA intronic	ENSG00000227838	Sudden cardiac arrest	intergenic	21658281
rs5753037	22	30581722	ncRNA intronic	ENSG00000225676	Type 1 diabetes	intergenic	19430480
rs573872	3	153472163	ncRNA intronic	ENSG00000240456	Infantile hypertrophic pyloric stenosis	intergenic	22306654
rs569688	8	60961821	promoter	ENSG00000254775	Myopia (pathological)	intergenic	23049088
rs565229	11	122189465	ncRNA intronic	ENSG00000255090	Hemostatic factors and hematological phenotypes	intergenic	17903294
rs56238310	3	111233239	ncRNA intronic	ENSG00000241177	Chronic obstructive pulmonary disease	intergenic	21685187
rs55681231	20	58946489	ncRNA intronic	ENSG00000228340	Metabolite levels (MHPG)	intergenic	23319000
rs555212	13	113756540	intronic	ENSG00000268130	End-stage coagulation	intergenic	23381943
rs543686	15	35068309	ncRNA intronic	ENSG00000250007	Prostate cancer (gene x gene interaction)	intergenic	22219177
rs539901	15	58674669	intronic	ENSG00000128918	Non-small cell lung cancer	intergenic	23144319
rs529866	16	11373320	intronic	ENSG00000175643	Inflammatory bowel disease	intergenic	23128233
rs528301	2	45154908	ncRNA intronic	ENSG00000259439	Alcohol and nictotine co-dependence	intergenic	22488850
rs525455	10	13103285	intronic	ENSG00000151468	Platelet aggregation	intergenic	20526338
rs508487	11	117075566	UTR3	ENSG00000160613	Cardiovascular disease risk factors	downstream	21943158

rs4985167	16	15082865	ncRNA exonic	ENSG00000261819	Phospholipid levels (plasma)	intronic	21829377
rs4972755	2	176239849	ncRNA intronic	ENSG00000229066	Bipolar disorder and major depressive disorder (combined)	intergenic	20351715
rs496300	21	44779680	ncRNA intronic	ENSG00000237989	Metabolic syndrome	intergenic	20694148
rs4955755	3	170494409	intronic	ENSG00000013297	Menopause (age at onset)	intergenic	19448619
rs4954218	2	135803425	intronic	ENSG00000176601	Corneal structure	intergenic	21979947
rs4950806	1	201952574	UTR5	ENSG00000176393	Butyrylcholinesterase levels	intronic	21862451
rs4948088	7	51027194	ncRNA intronic	ENSG00000228204	Type 1 diabetes	intergenic	19430480
rs494459	11	118574675	promoter	ENSG00000255422	Height	intergenic	20881960
rs494453	1	112192122	ncRNA exonic	ENSG00000215867	Osteoporosis	intronic	20548944
rs4938534	11	111275133	intronic	ENSG00000110777	Primary biliary cirrhosis	intergenic	23000144
rs493258	15	58687880	intronic	ENSG00000128918	Age-related macular degeneration	intergenic	20385826, 20385819
rs4924935	17	18753870	intronic	ENSG00000141127	Pancreatic cancer	intergenic	20686608
rs4923705	15	36293605	ncRNA intronic	ENSG00000259639	Attention deficit hyperactivity disorder	intergenic	20732626
rs4910742	11	5306509	intronic	ENSG00000196565, ENSG00000213931	Inflammatory biomarkers;Fetal hemoglobin levels	intergenic	22291609, 18245381
rs4901869	14	59334128	ncRNA intronic	ENSG00000258583	Panic disorder	intergenic	19165232
rs4894410	3	139043236	intronic	ENSG00000175110	Kawasaki disease	intergenic	22446961
rs488628	3	118121335	ncRNA intronic	ENSG00000243276	White matter integrity (interaction)	intergenic	23218918
rs488333	7	83911271	intronic	ENSG00000075213	Prion diseases	intergenic	22210626
rs4872511	8	22400989	ncRNA exonic	ENSG00000251034	HIV-1 control	intergenic	20205591
rs4871750	8	127902003	ncRNA intronic	ENSG00000253438	Obesity-related traits	intergenic	23251661
rs4871611	8	126537570	ncRNA	ENSG00000253111	Crohn's disease	intergenic	21102463

			intronic				
rs4871297	8	123706155	ncRNA exonic	ENSG00000253819	Type 1 diabetes nephropathy	intergenic	23028342
rs4864201	4	130731284	ncRNA intronic	ENSG00000249618	Obesity	intergenic	22484627
rs485499	3	159745863	ncRNA intronic	ENSG00000244040	Primary biliary cirrhosis	intergenic	21399635
rs4852324	2	74202578	ncRNA intronic	ENSG00000237883	Systemic lupus erythematosus	intergenic	23273568
rs4845812	1	5707816	ncRNA intronic	ENSG00000236948	Metabolite levels (MHPG)	intergenic	23319000
rs4838320	9	128831097	ncRNA intronic	ENSG00000232413	Economic and political preferences (immigration/crime)	intergenic	22566634
rs482329	1	234816554	ncRNA intronic	ENSG00000228044, ENSG00000230628	Life threatening arrhythmia	intergenic	22247754
rs4821132	22	33566587	ncRNA intronic	ENSG00000244472	Visceral adipose tissue adjusted for BMI	intergenic	22589738
rs4819143	21	47124178	intronic	ENSG00000183570	Insulin resistance/response	intergenic	21901158
rs4811971	20	56796784	intronic	ENSG00000124227	Height	intergenic	19893584
rs4803480	19	42066279	intronic	ENSG00000007129	Schizophrenia	intergenic	21682944
rs4800279	18	25070355	ncRNA intronic	ENSG00000264151	White matter integrity	intergenic	23218918
rs4799088	18	77573548	ncRNA intronic	ENSG00000267780	Amyotrophic lateral sclerosis	intergenic	20801717
rs4794820	17	38089344	ncRNA intronic	ENSG00000264968	Asthma	intergenic	22561531
rs4792394	17	13684917	ncRNA intronic	ENSG00000236088	Conduct disorder (symptom count)	intergenic	20585324
rs4783227	16	82594383	ncRNA intronic	ENSG00000261176	Response to antipsychotic treatment	intergenic	20195266
rs4780355	16	11347858	intronic	ENSG00000175643	Crohn's disease and psoriasis	downstream	22482804
rs4777845	15	93877425	ncRNA intronic	ENSG00000257060	Adiponectin levels	intergenic	22479202
rs4775413	15	61840103	ncRNA intronic	ENSG00000259616	Schizophrenia	intergenic	21926974
rs4775041	15	58674695	intronic	ENSG00000128918	Triglycerides;Metabolite levels;HDL cholesterol	intergenic	19043545, 18193043

rs4775031	15	58635583	intronic	ENSG00000128918	Cognitive performance	intergenic	20125193
rs4762767	12	19866129	intronic	ENSG00000139154	Pulmonary function	intergenic	21946350
rs4760790	12	71634794	intronic	ENSG00000127324	Type 2 diabetes	intergenic	20581827
rs4750316	10	6393260	UTR3	ENSG00000212743	Rheumatoid arthritis	intergenic	20453842, 18794853
rs4746003	10	71538292	promoter	ENSG00000230469	Alzheimer's disease	intergenic	22005930
rs4745062	9	73784264	intronic	ENSG00000083067	Longevity	intergenic	20834067
rs4743820	9	93928416	ncRNA intronic	ENSG00000229694	Inflammatory bowel disease	intergenic	23128233
rs472913	1	61095558	ncRNA intronic	ENSG00000226476	Bipolar disorder	intergenic	19416921
rs4722672	7	27231762	ncRNA intronic	ENSG00000253508	Ulcerative colitis	intergenic	23128233
rs4716055	6	9853919	intronic	ENSG00000181355	Adiponectin levels	intergenic	22479202
rs4713226	6	29434414	ncRNA intronic	ENSG00000225797	Immune reponse to smallpox (secreted IFN-alpha)	intergenic	22610502
rs4704970	5	155500992	intronic	ENSG00000170624	Multiple sclerosis (age of onset)	intergenic	19010793
rs4702982	5	99991648	ncRNA intronic	ENSG00000250806	Panic disorder	intergenic	19165232
rs4702435	5	7155612	ncRNA intronic	ENSG00000250974	Obesity-related traits	intergenic	23251661
rs4698412	4	15737348	intronic	ENSG00000109743	Parkinson's disease	intergenic	22451204, 21084426
rs4693646	4	84721814	ncRNA intronic	ENSG00000250546	Obesity-related traits	intergenic	23251661
rs4686914	3	187717540	ncRNA intronic	ENSG00000225058	Metabolite levels	intergenic	21909109
rs4684585	3	883851	ncRNA intronic	ENSG00000224957	Response to statin therapy	intergenic	20339536
rs4680534	3	159698945	ncRNA intronic	ENSG00000244040	Multiple sclerosis	intergenic	19525953
rs4670779	2	38044344	ncRNA exonic	ENSG00000225402	Bone mineral density (spine)	intergenic	19079262
rs4670766	2	37940542	intronic	ENSG00000163171	Total ventricular volume	intergenic	21116278
rs4660531	1	41839822	intronic	ENSG00000204060	Bipolar disorder	intergenic	21926972
rs4657616	1	158971086	intronic	ENSG00000163565	Hematology traits	intergenic	23263863
rs4656784	1	159326880	ncRNA intronic	ENSG00000228560	IgE levels;IgE levels	intergenic	22075330

rs4648356	1	2709164	intronic	ENSG00000215912	Multiple sclerosis	intergenic	21833088
rs4646949	6	34845449	intronic	ENSG00000065060	Fasting insulin-related traits (interaction with BMI)	downstream	22581228
rs4643574	2	315215	promoter	ENSG00000235779	Cognitive performance	intergenic	20125193
rs4622329	12	102321935	intronic	ENSG00000136048	Systemic lupus erythematosus	intergenic	23273568
rs4615179	4	38465471	ncRNA intronic	ENSG00000249534	Obesity-related traits	intergenic	23251661
rs4581712	16	80497601	ncRNA intronic	ENSG00000259867	Liver enzyme levels (gamma-glutamyl transferase)	intergenic	22001757
rs456867	5	55811092	intronic	ENSG00000225940	Urate levels	intergenic	23263486
rs4549631	6	126966308	intronic	ENSG00000260527	Height	intergenic	18391952
rs4538475	4	15737937	intronic	ENSG00000109743	Parkinson's disease	intergenic	19915576
rs4516970	6	160137687	intronic	ENSG00000112096	Iron status biomarkers	intergenic	19084217
rs4509693	10	102501571	intronic	ENSG00000075891	Alzheimer's disease	intergenic	20061627
rs4503880	18	56084054	ncRNA exonic	ENSG00000267675	Liver enzyme levels (gamma-glutamyl transferase)	intergenic	22001757
rs4489787	12	48811100	ncRNA intronic	ENSG00000257735	Prostate cancer (gene x gene interaction)	intergenic	22219177
rs4479806	5	26723783	ncRNA intronic	ENSG00000251033	Anorexia nervosa	intergenic	21079607
rs4470914	7	19616522	ncRNA intronic	ENSG00000223838	Height	intergenic	20881960
rs4469412	8	33565378	ncRNA intronic	ENSG00000253642	Immune response to smallpox vaccine (IL-6)	intergenic	22542470
rs445114	8	128323181	ncRNA intronic	ENSG00000246228	Prostate cancer	intergenic	21743057, 19767754
rs4446959	1	159357684	ncRNA intronic	ENSG00000228560	Chemerin levels	intergenic	20237162
rs4434872	1	153774276	ncRNA intronic	ENSG00000231827	Conduct disorder (symptom count)	intergenic	20585324
rs4418214	6	31391401	ncRNA intronic	ENSG00000206337	HIV-1 control	intergenic	21051598

rs4410871	8	128815029	ncRNA intronic	ENSG00000249859	Multiple sclerosis	intergenic	21833088
rs4380028	15	79111093	intronic	ENSG00000185787	Coronary heart disease	intergenic	21378988
rs4349147	16	3124920	intronic	ENSG00000008517	HIV-1 susceptibility	intergenic	22174851
rs4322600	14	88295600	ncRNA intronic	ENSG00000258807, ENSG00000259077	Breast cancer	intergenic	22923054
rs4313034	6	29973925	ncRNA exonic	ENSG00000237669	Graves' disease	ncRNA intronic	21900946
rs430	7	19106539	intronic	ENSG00000122691	Obesity-related traits	intergenic	23251661
rs4295627	8	130685457	ncRNA intronic	ENSG00000229140	Glioma	intergenic	21531791, 19578367
rs4293393	16	20364588	intronic	ENSG00000169344	Chronic kidney disease and serum creatinine levels	upstream	20686651
rs4281086	8	10352308	ncRNA intronic	ENSG00000253649	Obesity-related traits	intergenic	23251661
rs4273712	6	126964510	intronic	ENSG00000260527	Intracranial volume	intergenic	22504418
rs4262150	5	152288453	ncRNA intronic	ENSG00000249484	Bipolar disorder and schizophrenia	intergenic	22688191
rs4256159	3	18767404	ncRNA intronic	ENSG00000228956	Inflammatory bowel disease	intergenic	23128233
rs4246905	9	117553249	exonic	ENSG00000181634	Ulcerative colitis;Inflammatory bowel disease	intronic	21297633, 23128233
rs420017	15	89617081	ncRNA intronic	ENSG00000260123	Height;Obesity-related traits	intergenic	21998595, 23251661
rs416603	16	11364079	intronic	ENSG00000175643	Type 1 diabetes	upstream	18978792
rs416350	12	68779413	ncRNA intronic	ENSG00000251301	Temperament (bipolar disorder)	intergenic	22365631
rs415890	6	167406633	ncRNA intronic	ENSG00000227598	Crohn's disease	intergenic	21102463
rs4149178	6	43272188	UTR3	ENSG00000137204	Urate levels	intronic	23263486
rs4147141	1	69579252	ncRNA intronic	ENSG00000223883	Inattentive symptoms;Attention deficit hyperactivity disorder	intergenic	18821565
rs4142248	19	44888276	UTR3	ENSG00000267508	Myopia (pathological)	intergenic	23049088
rs4141819	2	67864675	ncRNA	ENSG00000235495	Endometriosis	intergenic	23104006

			intronic				
rs4128725	1	159405959	ncRNA intronic	ENSG00000228560	Select biomarker traits	intergenic	17903293
rs4105144	19	41358624	intronic	ENSG00000268797	Smoking behavior	intergenic	20418888
rs405460	14	59366315	ncRNA intronic	ENSG00000258583	Obesity-related traits	intergenic	23251661
rs4027132	2	12037492	ncRNA intronic	ENSG00000224184	Bipolar disorder	intergenic	21254220
rs400028	5	113849294	ncRNA intronic	ENSG00000246316	Obesity-related traits	intergenic	23251661
rs399885	2	79687252	intronic	ENSG00000066032	Response to antipsychotic treatment	intergenic	20195266
rs3930234	3	184216130	intronic	ENSG00000145191	Alcoholism (alcohol use disorder factor score)	intergenic	21529783
rs3923564	10	81735981	intronic	ENSG00000133661	Chronic obstructive pulmonary disease-related biomarkers	intergenic	23144326
rs3919627	3	42909160	intronic	ENSG00000180432, ENSG00000240747	Obesity-related traits	downstream	23251661
rs3916765	6	32685550	promoter	ENSG00000232080	Type 2 diabetes	intergenic	22693455
rs3913363	3	171653903	intronic	ENSG00000186329	Response to angiotensin II receptor blocker therapy	intergenic	22566498
rs3903239	1	170569317	ncRNA intronic	ENSG00000235303	Atrial fibrillation	intergenic	22544366
rs3892630	19	33181484	ncRNA intronic	ENSG00000267475	Red blood cell traits	intergenic	23222517
rs3884558	15	61702779	ncRNA intronic	ENSG00000259616	Response to amphetamines;Breast cancer (prognosis)	intergenic	22952603, 23319801
rs3864639	7	76738591	ncRNA intronic	ENSG00000214439, ENSG00000259628	Obesity-related traits	intergenic	23251661
rs3849491	3	76484183	ncRNA exonic	ENSG00000240809	Sex hormone-binding globulin levels	intergenic	22675492
rs3848445	17	14294021	ncRNA intronic	ENSG00000230647	Protein quantitative trait loci	intergenic	18464913
rs3845817	2	65758525	ncRNA	ENSG00000204929	Bipolar disorder	intergenic	21926972

			intronic				
rs3828890	6	31440669	ncRNA intronic	ENSG00000206337	Renal function-related traits (sCR);Renal function-related traits (eGRFcrea)	downstream	22797727
rs3820201	1	53581670	ncRNA exonic	ENSG00000235563	Hippocampal atrophy	intronic	22745009
rs38152	7	20047575	ncRNA intronic	ENSG00000243004	HIV-1 control	intergenic	20041166
rs3813582	16	79749353	ncRNA intronic	ENSG00000260876	Thyroid function	intergenic	22494929
rs3813579	16	79749276	ncRNA intronic	ENSG00000260876	Thyroid volume	intergenic	21565293
rs3810265	19	50223266	ncRNA exonic	ENSG00000243829	Panic disorder	intergenic	19165232
rs3806932	5	110405675	promoter	ENSG00000145777	Eosinophilic esophagitis (pediatric)	intergenic	20208534
rs3806308	1	20142866	ncRNA intronic	ENSG00000235434	Ulcerative colitis	intergenic	19122664
rs3803064	12	113173494	intronic	ENSG00000089169	Platelet counts	intergenic	21507922
rs3796619	4	1095281	ncRNA exonic	ENSG00000251639	Recombination rate (males)	intronic	18239089
rs3771180	2	102953617	UTR5	ENSG00000115602	Asthma	intronic	21804549
rs3764563	19	15724203	ncRNA exonic	ENSG00000266951	Inflammatory biomarkers	intergenic	22228203
rs3764400	17	46123932	ncRNA exonic	ENSG00000263412	Body mass index	intergenic	20935630
rs3761847	9	123690239	UTR5	ENSG00000056558	Rheumatoid arthritis	intronic	20453842, 17804836
rs3761218	20	3776175	intronic	ENSG00000101224	Bipolar disorder	upstream	21254220, 17554300
rs3760318	17	29247715	promoter	ENSG00000184060	Height	intergenic	18391951
rs3744728	17	6093951	promoter	ENSG00000262231	Bipolar disorder (mood-incongruent)	intergenic	23092984
rs3738443	1	247348189	ncRNA exonic	ENSG00000215795	Alcohol dependence	intergenic	21314694
rs371915	16	84578241	intronic	ENSG00000140950	Eosinophilic esophagitis (pediatric)	intergenic	20208534
rs361433	7	142104571	promoter	ENSG00000211732	Capecitabine sensitivity	intergenic	22864933
rs359965	2	219840891	ncRNA	ENSG00000236445	Immune	upstream	22542470

			exonic		response to smallpox vaccine (IL-6)		
rs35964523	3	898661	promoter	ENSG00000224239	Response to statin therapy	intergenic	20339536
rs35853021	15	58680643	intronic	ENSG00000128918	Lipid metabolism phenotypes	intergenic	22286219
rs35675666	1	8021973	UTR5	ENSG00000116288	Ulcerative colitis;Inflammatory bowel disease	intronic	21297633, 23128233
rs356220	4	90641340	ncRNA intronic	ENSG00000251095	Parkinson's disease	intergenic	22451204, 21738487, 21084426, 21044948, 20711177
rs356219	4	90637601	ncRNA intronic	ENSG00000251095	Parkinson's disease	intergenic	22438815, 21292315
rs35600665	15	24741291	ncRNA intronic	ENSG00000259905	Obesity-related traits	intergenic	23251661
rs34924084	16	5003074	intronic	ENSG00000118898	Metabolite levels (HVA/MHPG ratio)	intergenic	23319000
rs346291	6	80564836	ncRNA exonic	ENSG00000220918	Partial epilepsies	intronic	20522523
rs342296	7	106372903	ncRNA intronic	ENSG00000243797	Mean platelet volume	intergenic	22423221
rs342293	7	106372219	ncRNA intronic	ENSG00000243797	Mean platelet volume	intergenic	22423221, 22139419, 19820697, 19221038
rs342275	7	106359216	ncRNA intronic	ENSG00000243797	Platelet counts	intergenic	22139419
rs340874	1	214159256	ncRNA intronic	ENSG00000230461	Fasting glucose-related traits (interaction with BMI);Fasting glucose-related traits	intergenic	22581228, 20081858
rs340849	1	214118090	ncRNA intronic	ENSG00000230461	Alzheimer's disease	intergenic	22159054
rs333960	1	110439480	ncRNA exonic	ENSG00000261055	Obesity-related traits	intergenic	23251661
rs3130320	6	32223258	promoter	ENSG00000225914	Systemic lupus erythematosus	intergenic	21408207

rs3127573	6	160681393	UTR5	ENSG00000112499	Creatinine levels	intergenic	20383145
rs312691	17	68326338	ncRNA intronic	ENSG00000267109	Thyrotoxic hypokalemic periodic paralysis	intergenic	22863731
rs3126085	1	152300817	ncRNA intronic	ENSG00000237975	Atopic dermatitis	intergenic	21666691
rs3120665	1	152316590	ncRNA intronic	ENSG00000237975	Personality dimensions	intergenic	20691247
rs3117035	6	33086249	ncRNA exonic	ENSG00000224557	RR interval (heart rate)	ncRNA intronic	20031603
rs3113494	4	87832601	intronic	ENSG00000163633	Amyotrophic lateral sclerosis	intergenic	22470424
rs3110496	17	27917771	ncRNA intronic	ENSG00000264031	Height	intergenic	20881960
rs3094548	6	29355202	intronic	ENSG00000243729	Pulmonary function	intergenic	21946350
rs3093030	19	10397403	ncRNA exonic	ENSG00000267607	Soluble levels of adhesion molecules	upstream;downstream	20167578
rs3025343	9	136478355	ncRNA exonic	ENSG00000261018	Smoking behavior	intergenic	20418890
rs3018362	18	60082093	promoter	ENSG00000241088	Paget's disease;Bone mineral density (hip)	intergenic	21623375, 20436471, 18445777
rs2989476	1	61059259	ncRNA intronic	ENSG00000226476	Bipolar disorder	intergenic	21738484, 21254220
rs2980879	8	126481475	ncRNA intronic	ENSG00000253111	Adiponectin levels	intergenic	22479202
rs297941	12	50319086	ncRNA exonic	ENSG00000257771	Obsessive-compulsive disorder	intergenic	22889921
rs296547	1	200892137	ncRNA intronic	ENSG00000233217	Celiac disease	intergenic	20190752
rs2954038	8	126507389	ncRNA intronic	ENSG00000253111	Response to statin therapy	intergenic	20339536
rs2954033	8	126493746	ncRNA intronic	ENSG00000253111	Triglycerides-Blood Pressure (TG-BP)	intergenic	21386085
rs2954029	8	126490972	ncRNA intronic	ENSG00000253111	Triglycerides;LDL cholesterol;HDL cholesterol;"Cholesterol, total"	intergenic	20864672, 20686565, 19060906
rs2954026	8	126484526	ncRNA intronic	ENSG00000253111	HDL Cholesterol - Triglycerides (HDLC-TG)	intergenic	21386085
rs2954021	8	126482077	ncRNA	ENSG00000253111	Liver enzyme	intergenic	22001757,

			intronic		levels (alkaline phosphatase);Liver enzyme levels (alanine transaminase);LDL cholesterol		20864672
rs2935776	8	109629903	ncRNA intronic	ENSG00000253949	T-tau	intergenic	20932310
rs2923084	11	10388782	intronic	ENSG00000133805	HDL cholesterol	intergenic	20686565
rs2905072	9	135845035	intronic	ENSG00000165702	Bipolar disorder	intergenic	19416921
rs2903698	4	76332975	ncRNA intronic	ENSG00000248646, ENSG00000250735	Prion diseases	intergenic	22210626
rs2894207	6	31263751	ncRNA intronic	ENSG00000256166	Nasopharyngeal carcinoma	intergenic	20512145
rs28890483	15	59719169	intronic	ENSG00000157470	Bipolar disorder and schizophrenia	intergenic	20889312
rs2887571	12	1638171	promoter	ENSG00000111186	Bone mineral density	intergenic	22504420
rs2877832	14	27800177	ncRNA intronic	ENSG00000258932	Diabetes related insulin traits	intergenic	17903298
rs2860975	10	96766934	ncRNA exonic	ENSG00000228460	Immune response to smallpox vaccine (IL-6)	intergenic	22542470
rs2858884	6	32700083	promoter	ENSG00000226030	Narcolepsy	intergenic	20711174
rs2854160	17	61977248	promoter	ENSG00000259333	Height	intergenic	21998595
rs285406	8	87025013	intronic	ENSG00000147614	Adverse response to lamotrigine and phenytoin	intergenic	22379998
rs2847476	11	114191016	ncRNA intronic	ENSG00000256947	Volumetric brain MRI	intergenic	17903297
rs284489	8	105958020	intronic	ENSG00000253350	Glaucoma (primary open-angle)	intergenic	22570617
rs2836823	21	40380249	ncRNA intronic	ENSG00000237721	Nicotine dependence	intergenic	17158188
rs2836770	21	40314704	ncRNA intronic	ENSG00000205622	Immune response to smallpox vaccine (IL-6)	intergenic	22542470
rs2836754	21	40291740	ncRNA intronic	ENSG00000205622	Crohn's disease	intergenic	17554261
rs283610	5	73248512	promoter	ENSG00000259968	Obesity-related traits	intergenic	23251661

rs2834442	21	35690786	ncRNA intronic	ENSG00000214955	Height	intergenic	20881960
rs2833693	21	33552512	ncRNA intronic	ENSG00000230323	Temperament	intergenic	22832960
rs2833610	21	33385186	intronic	ENSG00000142149	Type 2 diabetes	intergenic	21490949
rs2833607	21	33381040	intronic	ENSG00000142149	Vitiligo	intergenic	19890347
rs2827312	21	23631676	ncRNA intronic	ENSG00000226043	Alcoholism (heaviness of drinking)	intergenic	21529783
rs2823455	21	17069166	promoter	ENSG00000226298	Panic disorder	intergenic	19165232
rs2820037	1	239436542	ncRNA intronic	ENSG00000227185	Hypertension	intergenic	17554300
rs2816316	1	192536813	promoter	ENSG00000232498	Celiac disease	intergenic	20190752, 18311140
rs279612	8	122706291	ncRNA intronic	ENSG00000248478	Metabolite levels (5-HIAA)	intergenic	23319000
rs2785173	11	35155732	ncRNA intronic	ENSG00000255521	Response to Vitamin E supplementation	intergenic	22437554
rs2766692	14	100684192	ncRNA intronic	ENSG00000258522, ENSG00000258982	Electroencephalographic traits in alcoholism	intergenic	22554406
rs2764980	10	3284007	ncRNA intronic	ENSG00000226762	Attention deficit hyperactivity disorder and conduct disorder	intergenic	18951430
rs275437	5	6879978	ncRNA intronic	ENSG00000250060	Obesity-related traits	intergenic	23251661
rs2742417	3	45731451	UTR5	ENSG00000211456	Response to antidepressant treatment	intronic	22041458
rs2739330	22	24295286	ncRNA intronic	ENSG00000231271	Liver enzyme levels (gamma-glutamyl transferase)	intergenic	22001757
rs2735839	19	51364623	promoter	ENSG00000167751	Prostate cancer;Prostate-specific antigen levels	downstream	18264097, 23269536
rs2731672	5	176842474	intronic	ENSG00000198055	Platelet function and related traits;Metabolite levels;Activated partial thromboplastin time	intergenic	22916037, 21546496, 20303064
rs2727405	11	13090942	ncRNA	ENSG00000255558	Obesity-related	intergenic	23251661

			intronic		traits		
rs2726807	4	183137398	intronic	ENSG00000218336	Schizophrenia	intergenic	22885689
rs272594	8	81470120	promoter	ENSG00000188856	Neutrophil count	intergenic	21507922
rs2718812	3	133399702	ncRNA intronic	ENSG00000242337	Iron status biomarkers	intergenic	19084217
rs2716816	4	180383312	ncRNA intronic	ENSG00000250993	Breast size	intergenic	22747683
rs271066	3	6377695	ncRNA intronic	ENSG00000226022	Alzheimer's disease (age of onset)	intergenic	22005931
rs270545	5	38051593	ncRNA intronic	ENSG00000250003	Major depressive disorder	intergenic	20125088
rs2705293	8	138918945	ncRNA intronic	ENSG00000253288, ENSG00000254361	Neuroticism	intergenic	18762592
rs2669010	12	77009060	ncRNA intronic	ENSG00000257526	Systemic lupus erythematosus	intergenic	21408207
rs266849	19	51349090	ncRNA intronic	ENSG00000267968	Prostate-specific antigen levels	intergenic	21160077
rs266717	3	186530484	ncRNA intronic	ENSG00000231724, ENSG00000232233	Adiponectin levels	intergenic	20011104
rs2657195	8	92559915	ncRNA intronic	ENSG00000253901	Disc degeneration (lumbar)	intergenic	22993228
rs2652834	15	63396867	ncRNA intronic	ENSG00000259672	HDL cholesterol	intergenic	20686565
rs2651244	1	70995562	promoter	ENSG00000233020	Inflammatory bowel disease	intergenic	23128233
rs261967	5	95850250	ncRNA intronic	ENSG00000251314	Body mass index	intergenic	22344219
rs260461	19	58770883	ncRNA exonic	ENSG00000268516	Attention deficit hyperactivity disorder	intronic	18821565
rs2592394	2	176991779	ncRNA intronic	ENSG00000237380	Magnesium levels	intergenic	20700443
rs2586502	17	48289070	ncRNA intronic	ENSG00000249406	Response to taxane treatment (docetaxel)	intergenic	23006423
rs2585417	20	52843799	intronic	ENSG00000101132	Obesity-related traits	intergenic	23251661
rs2575029	8	31174830	ncRNA intronic	ENSG00000253377	Obesity-related traits	intergenic	23251661
rs2571391	6	29923838	promoter	ENSG00000235290	IgE levels;IgE levels	intergenic	22075330
rs2562456	19	21666210	ncRNA exonic	ENSG00000268658	Pain	upstream	19207018
rs2545801	5	176841339	intronic	ENSG00000198055	Metabolite	intergenic	22703881,

					levels;Activated partial thromboplastin time		22286219
rs2540917	2	60608759	ncRNA intronic	ENSG00000223929	Mean corpuscular volume	intergenic	19862010
rs2526932	14	73081068	intronic	ENSG00000205683	C-reactive protein and white blood cell count	intergenic	22788528
rs2524276	6	31408265	ncRNA intronic	ENSG00000206337	Capecitabine sensitivity	intergenic	22864933
rs2523393	6	29705659	ncRNA exonic	ENSG00000214922	Multiple sclerosis	ncRNA intronic	19525953
rs251253	5	172480336	promote r	ENSG00000207210	PR interval	intergenic	20062060
rs2507838	11	58472799	intronic	ENSG00000149124	Body mass (lean)	intergenic	23108985
rs2501677	10	6946752	ncRNA intronic	ENSG00000234248	Fasting insulin-related traits (interaction with BMI)	intergenic	22581228
rs2499604	1	238103501	intronic	ENSG00000198626	Non-alcoholic fatty liver disease histology (AST)	intergenic	20708005
rs247617	16	56990716	promote r	ENSG00000263441	Metabolic syndrome	intergenic	22399527
rs2461751	2	176289319	ncRNA intronic	ENSG00000229066	Electrocardiogra phic conduction measures	intergenic	19389651
rs245914	7	29218159	intronic	ENSG00000106066, ENSG00000106069	Obesity-related traits	intergenic	23251661
rs245201	5	127169212	ncRNA intronic	ENSG00000230561	Brain imaging in schizophrenia (interaction)	intergenic	19023125
rs2444240	11	120040442	intronic	ENSG00000137699, ENSG00000176984	Corneal curvature	intergenic	22969067
rs2412980	22	30592069	ncRNA intronic	ENSG00000225676	Dialysis-related mortality	intergenic	21546767
rs2411984	17	47445751	ncRNA intronic	ENSG00000248714	Sex hormone-bindin g globulin levels	intergenic	22829776
rs2407103	8	36951914	promote r	ENSG00000253650	Insulin resistance/respon se	intergenic	21901158
rs240657	8	15665612	promote	ENSG00000250483	Intelligence	intergenic	22449649

			r				
rs240444	21	11002011	ncRNA intronic	ENSG00000187172	Bipolar disorder and schizophrenia	intergenic	20889312
rs2400997	14	101727107	ncRNA intronic	ENSG00000258497	Prostate cancer (gene x gene interaction)	intergenic	22219177
rs2398162	15	96830550	ncRNA intronic	ENSG00000247809	Hypertension	intergenic	17554300
rs2395528	10	80100861	ncRNA intronic	ENSG00000230417	Conduct disorder (interaction)	intergenic	18846501
rs2388896	10	8954224	ncRNA intronic	ENSG00000234752	Tetralogy of Fallot	intergenic	23297363
rs2382817	2	219151218	UTR5	ENSG00000135926	Inflammatory bowel disease	intronic	23128233
rs2369304	14	96206070	ncRNA intronic	ENSG00000257275	Neutrophil count	intergenic	21507922
rs2361502	2	234698790	intronic	ENSG00000185038	Bilirubin levels	intergenic	21646302
rs2360997	14	76812491	intronic	ENSG00000119715	Attention deficit hyperactivity disorder symptoms (interaction)	intergenic	18846501
rs2338104	12	109895168	UTR5	ENSG00000110906	HDL cholesterol	intronic	19060906, 18193043
rs2333163	4	176270885	ncRNA intronic	ENSG00000248551	Obesity-related traits	intergenic	23251661
rs233100	1	85772009	ncRNA intronic	ENSG00000223653	Multiple sclerosis	intergenic	21833088
rs2326017	17	46720565	ncRNA intronic	ENSG00000233283	Cognitive performance	intergenic	19734545
rs2312147	2	58222928	intronic	ENSG00000028116	Schizophrenia	intergenic	19571808
rs2303745	19	17420289	intronic	ENSG00000127220	Systemic lupus erythematosus	upstream	23273568
rs2293941	13	28491198	ncRNA intronic	ENSG00000247381	Fasting glucose-related traits (interaction with BMI)	intergenic	22581228
rs2292303	12	102513531	intronic	ENSG00000075188	Height	upstream	19893584
rs2292096	1	200826769	UTR3	ENSG00000118200	Epilepsy	intronic	22116939
rs228437	6	134898456	ncRNA intronic	ENSG00000232310	Melanoma	intergenic	21983787
rs2281388	6	33060118	ncRNA exonic	ENSG00000231461	Graves' disease	intergenic	21841780
rs2278702	15	80694922	intronic	ENSG00000259495	Bipolar disorder	intergenic	18711365
rs2278170	11	4496846	ncRNA exonic	ENSG00000225101	Amyotrophic lateral sclerosis	intergenic	22470424

rs2277862	20	34152782	ncRNA exonic	ENSG00000088340	Cholesterol, total	intergenic	20686565
rs2275606	6	146918950	ncRNA intronic	ENSG00000237468	Leprosy	intergenic	22019778
rs2275215	6	129861392	ncRNA intronic	ENSG00000226149	Body mass index	intergenic	20397748
rs2271404	2	112003867	ncRNA intronic	ENSG00000172965	Atopic dermatitis	intergenic	23042114
rs2267138	22	29793641	intronic	ENSG00000100280	Myopia (pathological)	intergenic	23049088
rs2259816	12	121435587	UTR3	ENSG00000135100	C-reactive protein;Coronary heart disease	intronic	22939635, 19198612
rs225848	14	30594657	ncRNA intronic	ENSG00000248975	Sexual dysfunction (SSRI/SNRI-related)	intergenic	22445761
rs2251301	8	11119037	ncRNA intronic	ENSG00000236827	Response to antipsychotic therapy (extrapyramidal side effects)	intergenic	19875103
rs2247056	6	31265490	ncRNA intronic	ENSG00000256166	Triglycerides	intergenic	20686565
rs2244067	1	187412042	promoter	ENSG00000231599	Metabolite levels (5-HIAA)	intergenic	23319000
rs2240466	7	72856269	UTR3	ENSG00000009954	Triglycerides;Caffeine consumption	intronic	21490707, 19060911
rs2233152	19	41281016	intronic	ENSG00000261857, ENSG00000268975	Kawasaki disease	upstream	22446962
rs223116	14	23977010	intronic	ENSG00000129460	Resting heart rate	intergenic	20639392
rs2220321	8	127911596	ncRNA intronic	ENSG00000253438	Obesity-related traits	intergenic	23251661
rs2213169	11	5303063	intronic	ENSG00000196565, ENSG00000213931	Mean corpuscular hemoglobin concentration;Hematology traits	intergenic	23263863
rs2199936	1	160864221	promoter	ENSG00000213080	Urate levels;Lipoprotein-associated phospholipase A2 activity change in response to statin	intergenic	23118302, 21943158, 20884846

					therapy;Cardiovascular disease risk factors		
rs219553	2	21577743	ncRNA intronic	ENSG00000231204, ENSG00000233005	Erectile dysfunction and prostate cancer treatment	intergenic	20932654
rs2193071	2	8036260	ncRNA exonic	ENSG00000226506	Obesity-related traits	intergenic	23251661
rs2173063	15	93131632	ncRNA intronic	ENSG00000258676	Subcutaneous adipose tissue	intergenic	22589738
rs217181	16	72114002	intronic	ENSG00000140830	Metabolite levels	intergenic	22916037
rs2168303	18	58375934	ncRNA intronic	ENSG00000267098	Gout	intergenic	23263486
rs216345	9	33799370	ncRNA intronic	ENSG00000235481	Bipolar disorder	downstream	18711365
rs2159324	19	45695738	intronic	ENSG00000007047, ENSG00000266958, ENSG00000267545	Quantitative traits	intergenic	19197348
rs2154319	1	41745770	ncRNA intronic	ENSG00000235358	Height	intergenic	20881960
rs2153299	10	28752106	ncRNA intronic	ENSG00000237128	Obesity-related traits	intergenic	23251661
rs2138852	17	27703349	ncRNA intronic	ENSG00000264808, ENSG00000266111	Mean platelet volume	intergenic	19820697, 19110211
rs2130017	11	89502376	promoter	ENSG00000204456	Response to taxane treatment (docetaxel)	intergenic	23006423
rs2119704	14	88487689	ncRNA intronic	ENSG00000258826	Multiple sclerosis	intergenic	21833088
rs2117032	12	21074122	intronic	ENSG00000111700, ENSG00000205754, ENSG00000257046	Bilirubin levels	intergenic	19419973
rs2114039	4	55092626	intronic	ENSG00000145216	Corneal curvature	intergenic	21665993
rs2112347	5	75015242	intronic	ENSG00000269850	Body mass index	intergenic	20935630
rs210135	20	31277094	ncRNA exonic	ENSG00000175730	Hematological parameters	intergenic	19820697
rs2093210	14	60957279	intronic	ENSG00000179008	Height	intergenic	20881960
rs2084898	11	120026748	intronic	ENSG00000137699	Stroke (pediatric)	intergenic	22990015
rs2080401	2	171540823	ncRNA intronic	ENSG00000213981	Coronary heart disease	intergenic	21347282
rs2075672	7	100240296	intronic	ENSG00000106327	Red blood cell traits	downstream	23222517
rs2073233	20	12874585	ncRNA intronic	ENSG00000233048	Brain structure	intergenic	20171287

rs2072590	2	177042633	ncRNA exonic	ENSG00000224189, ENSG00000226363	Ovarian cancer	ncRNA intronic	20852632
rs2058350	12	3913427	intronic	ENSG00000111224	Cognitive performance	intergenic	20125193
rs2057178	11	32364187	ncRNA intronic	ENSG00000227160	Tuberculosis	intergenic	22306650
rs2048485	18	42090667	ncRNA intronic	ENSG00000267337	Schizophrenia	intergenic	22885689
rs2043085	15	58680954	intronic	ENSG00000128918	Metabolic syndrome (bivariate traits)	intergenic	21386085
rs2040406	6	32603007	intronic	ENSG00000196735	Multiple sclerosis	intergenic	20453840
rs2034588	16	8329327	ncRNA intronic	ENSG00000260003	Response to antidepressant treatment	intergenic	22041458
rs2032794	5	86432617	ncRNA intronic	ENSG00000249061	Personality dimensions	intergenic	21173776
rs2032366	18	59266371	ncRNA intronic	ENSG00000267175, ENSG00000267279	Obesity-related traits	intergenic	23251661
rs2022309	1	95052476	ncRNA intronic	ENSG00000223675	End-stage coagulation	intergenic	23381943
rs1991866	8	130624105	ncRNA intronic	ENSG00000229140	Inflammatory bowel disease	intergenic	23128233
rs1978503	18	53664282	ncRNA intronic	ENSG00000267327	Breast cancer	intergenic	17903305
rs1967689	1	208039471	intronic	ENSG00000203709	Age-related macular degeneration	intergenic	23326517
rs1953600	10	81911725	UTR3	ENSG00000122359	Sarcoidosis	intergenic	22936702
rs1951082	14	27260043	ncRNA intronic	ENSG00000257842, ENSG00000257845	Attention deficit hyperactivity disorder and conduct disorder	intergenic	18951430
rs1949733	4	8503359	ncRNA intronic	ENSG00000205959	Response to antineoplastic agents	intergenic	21659360
rs1944582	18	45815417	intronic	ENSG00000184828	Response to taxane treatment (docetaxel)	intergenic	23006423
rs1936800	6	127436064	intronic	ENSG00000260527	Renal function-related traits (BUN)	intergenic	22797727
rs1930961	22	25875265	ncRNA intronic	ENSG00000100058	Bipolar disorder (mood-incongruent)	intergenic	23092984
rs1923539	10	81694950	ncRNA intronic	ENSG00000242600	Chronic obstructive	intergenic	23144326

					pulmonary disease-related biomarkers		
rs1917445	11	103589094	ncRNA intronic	ENSG00000254987	Neutrophil count	intergenic	21507922
rs1912453	1	162821291	UTR3	ENSG00000185860	Colorectal cancer	intergenic	23266556
rs1900004	10	70000881	ncRNA intronic	ENSG00000233590	Vertical cup-disc ratio;Optic disc parameters	intergenic	20548946
rs1898036	15	36349846	ncRNA intronic	ENSG00000259639	Response to tocilizumab in rheumatoid arthritis	intergenic	22491018
rs1897031	15	54341465	ncRNA exonic	ENSG00000259619	Cognitive performance	intronic	19734545
rs1895320	5	159849586	UTR5	ENSG00000164611	Insulin-related traits	intronic	22791750
rs1893154	18	905125	ncRNA exonic	ENSG00000265179	Obesity-related traits	intronic	23251661
rs1892534	1	66105944	UTR3	ENSG00000116678	C-reactive protein	intergenic	18439548
rs1884537	14	101251989	ncRNA intronic	ENSG00000214548	Optic disc size (disc)	intergenic	20395239
rs1884302	20	7106289	ncRNA intronic	ENSG00000232271	Sagittal craniosynostosis	intergenic	23160099
rs1883414	6	33086448	ncRNA exonic	ENSG00000224557	Nephropathy	ncRNA intronic	21399633
rs1879248	3	180551214	intronic	ENSG00000145075	Schizophrenia	intergenic	21926974
rs1878047	19	51773802	ncRNA intronic	ENSG00000268595	Body mass index	intergenic	19851299
rs1875517	3	117307567	intronic	ENSG00000185565	Waist circumference	intergenic	17903300
rs1867504	3	133410661	ncRNA exonic	ENSG00000242337	Iron status biomarkers	intergenic	19084217
rs1867485	16	86681031	ncRNA intronic	ENSG00000261161	Obesity-related traits	intergenic	23251661
rs1859962	17	69108753	ncRNA intronic	ENSG00000260785	Prostate cancer	intergenic	21743057, 19767753, 18264097, 17603485
rs1853665	6	150298842	ncRNA exonic	ENSG00000219298	Radiation response	intergenic	20923822
rs1845344	4	120962385	promoter	ENSG00000253825	F-cell distribution	intergenic	21326311
rs1800588	15	58723675	intronic	ENSG00000128918, ENSG00000166035	HDL cholesterol	upstream	18193044
rs17824620	12	113100994	intronic	ENSG00000089169	Platelet counts	intergenic	22139419

rs17810546	3	159665050	ncRNA intronic	ENSG00000244040	Celiac disease;Behcet's disease	intergenic	20190752, 18311140, 23291587
rs17788937	12	77899510	ncRNA intronic	ENSG00000231121	Myopia (pathological)	intergenic	23049088
rs17780304	17	30535856	UTR3	ENSG00000126858	Obesity-related traits	intronic	23251661
rs17780086	17	30343282	intronic	ENSG00000185158	Height	intergenic	20881960
rs17773430	18	57963117	ncRNA intronic	ENSG00000267401	Obesity and blood pressure	intergenic	22013104
rs17767419	16	79744548	ncRNA intronic	ENSG00000260876	Thyroid volume	intergenic	21565293
rs17767225	14	71666297	ncRNA intronic	ENSG00000257692	Alzheimer's disease (late onset)	intergenic	22881374
rs17718828	13	75128114	ncRNA exonic	ENSG00000236678	Phospholipid levels (plasma)	ncRNA intronic	22359512
rs17679567	16	76890126	ncRNA intronic	ENSG00000259995	Response to TNF-alpha inhibitors in rheumatoid arthritis	intergenic	22569225
rs17666963	10	125207501	ncRNA intronic	ENSG00000230131	Cardiac Troponin-T levels	intergenic	23247143
rs17663555	5	72432036	ncRNA intronic	ENSG00000251599	Renal function-related traits (BUN)	intergenic	22797727
rs17635075	4	167343939	ncRNA intronic	ENSG00000249675	Obesity-related traits	intergenic	23251661
rs17632159	5	72431482	ncRNA intronic	ENSG00000251599	Urate levels	intergenic	23263486
rs17608059	17	13910549	ncRNA intronic	ENSG00000236088	Temperament	intergenic	22832960
rs17605562	2	12037179	ncRNA intronic	ENSG00000224184	Hair morphology	intergenic	19896111
rs17589290	4	112923237	ncRNA intronic	ENSG00000249815	Coronary heart disease	intergenic	21347282
rs17586843	4	116924184	ncRNA intronic	ENSG00000248173	Age-related macular degeneration	intergenic	23326517
rs17581368	8	21662445	intronic	ENSG00000168546	Entorhinal cortical thickness	intergenic	21116278
rs17577085	5	141863604	ncRNA intronic	ENSG00000231185	Coronary heart disease	intergenic	21347282
rs1757171	6	37487044	ncRNA intronic	ENSG00000204110	Cognitive performance	intergenic	19734545

rs17511627	13	26724328	ncRNA intronic	ENSG00000233963	Alzheimer's disease	intergenic	22159054
rs17511102	2	37960613	intronic	ENSG00000163171	Height	intergenic	20881960
rs17491951	2	2926013	ncRNA intronic	ENSG00000234423	White matter integrity (interaction)	intergenic	23218918
rs17468244	5	124848906	ncRNA intronic	ENSG00000260192	Obesity-related traits	intergenic	23251661
rs174550	11	61571478	UTR5	ENSG00000149485	Phospholipid levels (plasma);Fasting glucose-related traits (interaction with BMI);Fasting glucose-related traits	intronic	22581228, 21829377, 20081858
rs174549	11	61571382	UTR5	ENSG00000149485	Comprehensive strength and appendicular lean mass;Metabolite levels	intronic	22960237, 23281178
rs174548	11	61571348	UTR5	ENSG00000149485	Triglycerides;Metabolite levels;HDL cholesterol;Hematology traits	intronic	20864672, 19043545, 23303382
rs17445836	16	86017663	ncRNA intronic	ENSG00000269667	Multiple sclerosis	intergenic	19525953
rs17439299	11	15620586	ncRNA intronic	ENSG00000254789	Obesity-related traits	intergenic	23251661
rs17431357	12	120880434	intronic	ENSG00000111780	Insulin resistance/response	intergenic	21901158
rs17429217	12	117295333	UTR3	ENSG00000135116	Alzheimer's disease (age of onset)	intergenic	22005931
rs17419851	6	24749413	ncRNA intronic	ENSG00000224164	Neutrophil count	intergenic	21507922
rs17410015	1	101551926	ncRNA intronic	ENSG00000233184	Response to antipsychotic treatment	intergenic	20195266
rs17398575	7	106409452	ncRNA intronic	ENSG00000243797	Carotid intima media thickness	intergenic	21909108
rs17356664	19	45740771	intronic	ENSG00000007047	Platelet counts	intergenic	22139419
rs1733724	10	54223977	ncRNA intronic	ENSG00000231131	Ventricular conduction;Elect	intergenic	21076409, 20062063

					rocardiographic traits		
rs17331332	4	106808107	intronic	ENSG00000138785	Pulmonary function (interaction)	intergenic	23284291
rs17321515	8	126486409	ncRNA intronic	ENSG00000253111	Triglycerides	intergenic	18193044, 18193043
rs17299841	6	128021803	intronic	ENSG00000260527	Immune response to smallpox vaccine (IL-6)	intergenic	22610502
rs17291045	4	161506897	ncRNA intronic	ENSG00000249425	HIV-1 control	intergenic	20041166
rs17290922	16	57024317	intronic	ENSG00000140853	Schizophrenia	intergenic	23212062
rs17275498	2	231420401	ncRNA intronic	ENSG00000235419	Cognitive performance	intergenic	20125193
rs17249754	12	90060586	intronic	ENSG00000070961	Blood pressure;Biomedical quantitative traits	intergenic	21909110, 21572416, 19396169
rs17229285	2	199523122	ncRNA intronic	ENSG00000225421	Ulcerative colitis	intergenic	23128233
rs1719271	15	65183801	intronic	ENSG00000249240	Platelet counts	intergenic	22139419
rs17159640	7	112086333	ncRNA exonic	ENSG00000180019	Mortality among heart failure patients	intronic	20400778
rs17129289	1	84232748	ncRNA intronic	ENSG00000233008	Obesity-related traits	intergenic	23251661
rs17121944	12	59440232	ncRNA intronic	ENSG00000257443	Temperament (bipolar disorder)	intergenic	22365631
rs17111920	14	27406892	ncRNA intronic	ENSG00000258081	Bipolar disorder and schizophrenia	intergenic	20889312
rs17100498	5	143003605	ncRNA intronic	ENSG00000249881	Response to antipsychotic treatment	intergenic	20195266
rs17086172	18	70227021	intronic	ENSG00000141668	Airflow obstruction;Airflow obstruction	intergenic	22837378
rs17084051	4	55087581	ncRNA exonic	ENSG00000242670	Corneal astigmatism;Corneal curvature	intergenic	22144915, 23401653
rs17079928	13	24654228	intronic	ENSG00000182957	Orofacial clefts	intergenic	22419666
rs17079773	13	24598384	ncRNA intronic	ENSG00000228741	Inattentive symptoms	intergenic	18821565
rs17057678	6	130031493	intronic	ENSG00000260527	Antineutrophil cytoplasmic	upstream	22808956

					antibody-associated vasculitis;Antineutrophil cytoplasmic antibody-associated vasculitis		
rs17057381	8	27416801	promoter	ENSG00000234770	Dental caries	intergenic	23259602
rs17054265	9	91260562	ncRNA intronic	ENSG00000228189	Obesity-related traits	intergenic	23251661
rs17053082	5	155394230	intronic	ENSG00000170624	Type 2 diabetes	intergenic	23300278
rs17050782	4	140423134	intronic	ENSG00000145391	Smoking behavior	intergenic	19247474
rs17043947	2	22736987	ncRNA intronic	ENSG00000231200	Self-rated health	intergenic	20707712
rs17040773	2	112500035	promoter	ENSG00000228615	Bone mineral density	intergenic	22504420
rs17036170	3	12330411	UTR5	ENSG00000132170	Drug-induced liver injury;Drug-induced liver injury	intronic	22968431
rs17031508	4	102560290	intronic	ENSG00000153064	Blood pressure	intergenic	22763476
rs17025426	1	110645327	ncRNA intronic	ENSG00000258673	Visceral adipose tissue/subcutaneous adipose tissue ratio	intergenic	22589738
rs17022027	4	95614278	ncRNA intronic	ENSG00000249951	Visceral fat	intergenic	22589738
rs1701704	12	56412487	intronic	ENSG00000123411	Type 1 diabetes autoantibodies;Type 1 diabetes;Asthma;Alopecia areata	intergenic	21829393, 21804548, 20596022, 18198356
rs170020	5	151344558	ncRNA intronic	ENSG00000254226	Interstitial lung disease;Interstitial lung disease	intergenic	21787189
rs17000918	22	49812502	intronic	ENSG00000188511	Immune reponse to smallpox (secreted IL-1beta)	intergenic	22610502
rs16966460	15	38511983	ncRNA intronic	ENSG00000259380	Bipolar disorder	intergenic	18711365
rs16965039	16	57047299	ncRNA exonic	ENSG00000259922	Coronary heart disease	intergenic	21347282
rs16961543	16	59940188	ncRNA intronic	ENSG00000261807	Obesity-related traits	intergenic	23251661
rs16948048	17	47440466	ncRNA	ENSG00000248714	Diastolic blood	upstream	19430483

			intronic		pressure		
rs16940212	15	58694020	intronic	ENSG00000128918	HDL cholesterol	intergenic	21909109
rs16939046	8	76147954	ncRNA intronic	ENSG00000249395	Information processing speed	intergenic	21130836
rs16922670	9	106024220	ncRNA intronic	ENSG00000225564	Alzheimer's disease	intergenic	22005930
rs16921914	11	31210771	intronic	ENSG00000170959	Bone mineral density (spine)	intergenic	19801982
rs16912238	9	125573030	intronic	ENSG00000136940	Obesity-related traits	intergenic	23251661
rs16910421	11	12070665	ncRNA intronic	ENSG00000254486	Visceral fat	intergenic	22589738
rs16909318	8	82445224	ncRNA exonic	ENSG00000253374	Visceral fat	intergenic	22589738
rs16906916	10	56848985	intronic	ENSG00000150275	Response to tocilizumab in rheumatoid arthritis	intergenic	22491018
rs16902094	8	128320346	ncRNA intronic	ENSG00000246228	Prostate cancer	intergenic	19767754
rs1689800	1	182168885	ncRNA intronic	ENSG00000228918	HDL cholesterol	intergenic	20686565
rs16872248	4	22021500	ncRNA intronic	ENSG00000250039	Obesity-related traits	intergenic	23251661
rs16872085	8	105957540	intronic	ENSG00000253350	Sudden cardiac arrest	intergenic	21658281
rs16869652	6	33851173	ncRNA intronic	ENSG00000249346	Schizophrenia	intergenic	22885689
rs16862782	3	187687890	ncRNA intronic	ENSG00000224187	Myopia (pathological)	intergenic	23049088
rs16861531	2	14398052	ncRNA intronic	ENSG00000230448	Response to antidepressant treatment	intergenic	22041458
rs16858228	2	12212445	ncRNA intronic	ENSG00000224184	Obesity-related traits	intergenic	23251661
rs16849225	2	164906820	ncRNA intronic	ENSG00000237844	Blood pressure	intergenic	21572416
rs16844716	1	199356516	ncRNA exonic	ENSG00000236468	Dialysis-related mortality	intergenic	21546767
rs16839626	2	204089730	intronic	ENSG00000144426	Obesity-related traits	intergenic	23251661
rs16827293	2	150509332	ncRNA intronic	ENSG00000231969	Waist-to-hip circumference ratio (interaction)	intergenic	23192594
rs167769	12	57503775	UTR5	ENSG00000166888	Eosinophilic esophagitis (pediatric)	intronic	20208534

rs1668357	7	38004406	intronic	ENSG00000106483	Myopia (pathological)	intergenic	23049088
rs1656966	3	186466252	ncRNA intronic	ENSG00000197099	Myopia (pathological)	intergenic	23049088
rs161645	5	104069917	ncRNA intronic	ENSG00000251574	Depression (quantitative trait)	intergenic	23290196
rs1605834	2	22576100	ncRNA intronic	ENSG00000231200	Bipolar disorder and schizophrenia	intergenic	20889312
rs160441	8	90656988	ncRNA intronic	ENSG00000251136	Tuberculosis	intergenic	20694014
rs1601875	3	3658840	ncRNA intronic	ENSG00000223727	Bipolar disorder	intergenic	18711365
rs1593	4	187195551	UTR3	ENSG00000088926	Activated partial thromboplastin time	intronic	22703881
rs1568889	11	28009463	ncRNA intronic	ENSG00000255094	Bipolar disorder	intergenic	21254220
rs1566039	5	6821914	ncRNA intronic	ENSG00000249734	Sphingolipid levels	intergenic	22359512
rs1564892	12	104445742	intronic	ENSG00000120820	Corneal structure	intergenic	23291589
rs1562430	8	128387852	ncRNA intronic	ENSG00000246228, ENSG00000253929	Breast cancer	intergenic	21263130, 20453838
rs1558744	12	68504592	ncRNA intronic	ENSG00000255733	Ulcerative colitis	intergenic	20228799, 19122664
rs1551398	8	126540051	ncRNA intronic	ENSG00000253111	Crohn's disease	intergenic	18587394
rs1550057	4	142117424	intronic	ENSG00000170153	Conduct disorder (case status)	intergenic	20585324
rs1536827	10	135306158	ncRNA intronic	ENSG00000214279	Visceral adipose tissue/subcutaneous adipose tissue ratio	intergenic	22589738
rs1534422	2	12640741	ncRNA intronic	ENSG00000224184	Type 1 diabetes	intergenic	19430480
rs1533665	15	78530940	intronic	ENSG00000103740	Personality dimensions	intergenic	20691247
rs1532815	1	165180089	ncRNA exonic	ENSG00000224702	Response to acetaminophen (hepatotoxicity)	intronic	21177773
rs1532085	15	58683366	intronic	ENSG00000128918	Triglycerides;Metabolite levels;Metabolic syndrome;HDL cholesterol;"Cho	intergenic	22916037, 22399527, 20686565, 19060911, 19060910,

					lesterol, total";Red blood cell traits		23222517
rs1529093	2	177909595	ncRNA intronic	ENSG00000236501	Non-alcoholic fatty liver disease histology (other)	intergenic	20708005
rs1523288	3	165280209	ncRNA intronic	ENSG00000244128	Heart failure	intergenic	20445134
rs1521	6	31350704	promoter	ENSG00000225851	Graves' disease	intergenic	21841780
rs1520333	8	79401038	ncRNA intronic	ENSG00000254266	Multiple sclerosis	intergenic	21833088
rs1514178	1	61205469	ncRNA intronic	ENSG00000231252	Phospholipid levels (plasma)	intergenic	21829377
rs1498095	3	42093422	promoter	ENSG00000229339	Visceral adipose tissue/subcutaneous adipose tissue ratio	intergenic	22589738
rs1497546	3	98034526	ncRNA intronic	ENSG00000251088	Drug-induced liver injury (flucloxacillin)	intergenic	19483685
rs1495377	12	71577101	intronic	ENSG00000127324	Type 2 diabetes;Creutzfeldt-Jakob disease (variant)	intergenic	22137330, 17554300
rs1493682	4	121041286	ncRNA intronic	ENSG00000250938	HIV-1 viral setpoint	intergenic	22174851
rs1491818	11	29195137	ncRNA intronic	ENSG00000254526	Amyotrophic lateral sclerosis (age of onset)	intergenic	22959728
rs1490388	6	126835655	intronic	ENSG00000260527	Height	intergenic	18391951
rs1490384	6	126851160	intronic	ENSG00000260527	Height	intergenic	20881960
rs1490075	3	176422652	ncRNA intronic	ENSG00000232461	Response to amphetamines	intergenic	22952603
rs1488665	11	45528116	ncRNA intronic	ENSG00000254746, ENSG00000255041	Obesity-related traits	intergenic	23251661
rs1484948	11	41852664	ncRNA intronic	ENSG00000255171	RR interval (heart rate)	intergenic	20031603
rs1476442	7	91272344	ncRNA intronic	ENSG00000223665	Obesity-related traits	intergenic	23251661
rs1464108	12	131022010	intronic	ENSG00000060709	Alzheimer's disease	intergenic	22005930
rs1457451	2	65862378	ncRNA intronic	ENSG00000204929, ENSG00000235725	Iron status biomarkers	intergenic	19084217
rs1455244	18	11494199	ncRNA intronic	ENSG00000267252	Schizophrenia	intergenic	23212062
rs1449984	2	23414651	ncRNA	ENSG00000232451	Depression	intergenic	20800221

			intronic		(quantitative trait)		
rs1446468	2	164963486	ncRNA intronic	ENSG00000237844	Blood pressure	intergenic	21909110
rs1444418	10	64560470	ncRNA intronic	ENSG00000238280	Atopic dermatitis	intergenic	23042114
rs1443170	4	128320908	ncRNA intronic	ENSG00000248491	Obesity-related traits	intergenic	23251661
rs1440581	4	89226422	ncRNA intronic	ENSG00000246375	Metabolite levels	intergenic	22916037, 22286219
rs1438949	5	152504929	ncRNA intronic	ENSG00000249484	Response to amphetamines	intergenic	22952603
rs1432723	5	155302582	intronic	ENSG00000170624	Obesity-related traits	intergenic	23251661
rs1432295	2	61066666	ncRNA intronic	ENSG00000228414	Hodgkin's lymphoma	intergenic	21037568
rs1427593	2	137555224	intronic	ENSG00000144229	Pancreatic cancer	intergenic	20686608
rs1421746	5	127151976	ncRNA intronic	ENSG00000230561	Amyotrophic lateral sclerosis (age of onset)	intergenic	22959728
rs1420956	18	25167945	ncRNA intronic	ENSG00000264151	Obesity-related traits	intergenic	23251661
rs1420101	2	102957716	exonic	ENSG00000115602	Eosinophil counts	intronic	19198610
rs1417437	1	70154441	intronic	ENSG00000033122	Orofacial clefts	intergenic	22419666
rs1408272	6	25842951	intronic	ENSG00000124564	Mean corpuscular hemoglobin;Iron status biomarkers;Hematology traits;Red blood cell traits	intergenic	21149283, 19862010, 19853236, 23222517
rs1406961	20	61895920	intronic	ENSG00000101198	Cardiovascular disease risk factors	intergenic	21779381
rs1406428	2	51736994	ncRNA intronic	ENSG00000231918	Response to tocilizumab in rheumatoid arthritis	intergenic	22491018
rs1405262	2	6134940	ncRNA intronic	ENSG00000232044	HIV-1 control	intergenic	20041166
rs1402837	2	169757354	intronic	ENSG00000152253	Glycated hemoglobin levels	upstream	19096518
rs1402279	12	77729385	ncRNA intronic	ENSG00000231121	Smoking behavior	intergenic	19247474

rs1398217	18	44752238	intronic	ENSG00000215474	Menarche (age at onset)	intergenic	21102462
rs1386330	11	87819427	ncRNA intronic	ENSG00000255102	Multiple sclerosis (age of onset)	intergenic	19010793
rs13831	20	57475191	UTR3	ENSG00000087460	Event-related brain oscillations	intronic	21184583
rs1380131	14	54072858	ncRNA intronic	ENSG00000225680, ENSG00000237356	Alcoholism (heaviness of drinking)	intergenic	21529783
rs1373004	10	54427825	ncRNA intronic	ENSG00000228651	Bone mineral density	intergenic	22504420
rs1371867	8	101330209	intronic	ENSG00000034677	Atrioventricular conduction	intergenic	21041692
rs1371737	7	9189541	ncRNA intronic	ENSG00000260543	Obesity-related traits	intergenic	23251661
rs1366594	5	88376061	ncRNA intronic	ENSG00000248309	Bone mineral density (hip);Bone mineral density	intergenic	21533022, 19801982, 22504420
rs1361108	6	126767600	intronic	ENSG00000260527	Menarche (age at onset);Height	intergenic	21998595, 21102462
rs1351394	12	66351826	UTR3	ENSG00000149948	Height	intronic	20881960
rs134882	22	42670965	ncRNA intronic	ENSG00000182057	Bipolar disorder and schizophrenia	downstream	20889312
rs1344694	2	216893637	intronic	ENSG00000115425, ENSG00000118242	Alcohol dependence	intergenic	22004471
rs13438712	7	105542580	intronic	ENSG00000128536	Obesity-related traits	intergenic	23251661
rs1341239	6	22304204	ncRNA intronic	ENSG00000260049	Paget's disease	intergenic	21623375
rs13409348	2	79539988	intronic	ENSG00000066032	Bipolar disorder	intergenic	19416921
rs13407662	2	53782559	intronic	ENSG00000115239	Stroke (ischemic)	intergenic	23041239
rs13398206	2	199169096	ncRNA intronic	ENSG00000236653	Prostate cancer (gene x gene interaction)	intergenic	22219177
rs13383928	2	209578600	intronic	ENSG00000144407	Lung cancer-asbestos exposure interaction	intergenic	22637743
rs13381277	18	74318610	intronic	ENSG00000263812	Dental caries	intergenic	23064961
rs13333226	16	20365654	intronic	ENSG00000169344	Hypertension	intergenic	21082022
rs13330107	16	76878862	ncRNA intronic	ENSG00000259995	Inattentive symptoms	intergenic	18821565
rs13281615	8	128355618	ncRNA intronic	ENSG00000246228, ENSG00000253929	Breast cancer	intergenic	17529967

rs13273073	8	23584226	ncRNA intronic	ENSG00000253471, ENSG00000254002	Treatment response for severe sepsis;Treatment response for severe sepsis	intergenic	22310353
rs1326986	10	19929513	intronic	ENSG00000204740	Ankylosing spondylitis	intergenic	20062062
rs13252298	8	128095156	promoter	ENSG00000253264	Prostate cancer	intergenic	21743057
rs13228694	7	99940307	intronic	ENSG00000121716	Obesity-related traits	intergenic	23251661
rs1320976	1	169073346	promoter	ENSG00000237707	QT interval	intergenic	23166209
rs13204965	6	127167072	intronic	ENSG00000260527	Bone mineral density	intergenic	21533022, 22504420
rs13204742	6	128245765	intronic	ENSG00000260527	Crohn's disease	intergenic	23128233
rs1320333	2	679179	ncRNA intronic	ENSG00000233296	Obesity-related traits	intergenic	23251661
rs13195786	6	10163968	intronic	ENSG00000181355	Calcium levels	intergenic	20705733
rs13179048	5	95542726	ncRNA intronic	ENSG00000250551, ENSG00000251314	Fasting glucose-related traits (interaction with BMI)	intergenic	22581228
rs13172324	5	7368845	ncRNA intronic	ENSG00000249865	Myopia (pathological)	intergenic	23049088
rs1317209	1	20140036	promoter	ENSG00000235434	Ulcerative colitis	downstream	20228799
rs1317082	3	169497585	ncRNA exonic	ENSG00000269984	Telomere length	intronic	23001564
rs13166814	5	117337753	ncRNA intronic	ENSG00000249797	Subcutaneous adipose tissue	intergenic	22589738
rs13098911	3	46235201	intronic	ENSG00000183625	Celiac disease	intergenic	20190752
rs13073817	3	18706858	ncRNA intronic	ENSG00000228956	Crohn's disease	intergenic	21102463
rs13053817	22	29847722	ncRNA intronic	ENSG00000225465	Carotid atherosclerosis in HIV infection	intergenic	20009918
rs13034723	2	190985680	intronic	ENSG00000187699	Body mass index	intergenic	22344221
rs1302019	6	147971574	intronic	ENSG00000203727	Myopia (pathological)	intergenic	23049088
rs13017846	2	40757791	intronic	ENSG00000183023	QT interval	intergenic	22726844
rs13017599	2	61164331	ncRNA exonic	ENSG00000237522	Rheumatoid arthritis;Psoriatic arthritis	intergenic	22170493, 19503088
rs13015714	2	102971865	intronic	ENSG00000115604	Celiac disease;Atopic dermatitis	intergenic	23042114, 18311140

rs13012266	2	37957480	intronic	ENSG00000163171	Metabolite levels (HVA/MHPG ratio)	intergenic	23319000
rs13010713	2	181996045	ncRNA intronic	ENSG00000234663	Celiac disease	intergenic	20190752
rs13002573	2	164915208	ncRNA intronic	ENSG00000237844	Blood pressure	intergenic	21909110
rs1299548	7	7302293	ncRNA intronic	ENSG00000230825	Visceral adipose tissue adjusted for BMI	intergenic	22589738
rs12949531	17	13733806	ncRNA intronic	ENSG00000236088	Systemic lupus erythematosus	intergenic	19165918
rs12941150	17	70363395	ncRNA intronic	ENSG00000227036	Pulmonary function decline	intergenic	22424883
rs12938916	17	55866287	ncRNA intronic	ENSG00000265542	Bipolar disorder	intergenic	21254220
rs12928822	16	11403893	intronic	ENSG00000175643	Celiac disease	intergenic	20190752
rs12917707	16	20367690	promoter	ENSG00000169344	Renal function and chronic kidney disease;Chronic kidney disease	intergenic	20383146, 19430482
rs12907914	15	39315358	ncRNA intronic	ENSG00000259278, ENSG00000259345	Cardiac hypertrophy	intergenic	21348951
rs12901682	15	78833223	UTR5	ENSG00000041357	Response to tocilizumab in rheumatoid arthritis	intronic	22491018
rs12753569	1	76484014	ncRNA intronic	ENSG00000225605	Personality dimensions	intergenic	20691247
rs12742923	1	83489844	ncRNA intronic	ENSG00000230817	HIV-associated dementia;HIV-associated dementia	intergenic	22628157
rs1273516	19	15677710	ncRNA intronic	ENSG00000269516	Response to tocilizumab in rheumatoid arthritis	intergenic	22491018
rs12731740	1	208024820	intronic	ENSG00000203709	Biomedical quantitative traits	intergenic	19396169
rs12726330	1	155108167	intronic	ENSG00000169241	Parkinson's disease	upstream;downstream	22451204
rs12679254	8	74274191	ncRNA intronic	ENSG00000253339	Inattentive symptoms	intergenic	18821565
rs12653946	5	1895829	ncRNA intronic	ENSG00000249326	Prostate cancer	intergenic	22923026, 20676098

rs12646107	4	174515682	ncRNA exonic	ENSG00000261672	Response to amphetamines	intergenic	22952603
rs12636651	3	46282391	intronic	ENSG00000183625	Monocyte chemoattractant protein-1;Obesity-related traits;Monocyte chemoattractant protein-1	intergenic	23017229, 23251661
rs12629805	3	146632164	ncRNA intronic	ENSG00000243620	Subcutaneous adipose tissue	intergenic	22589738
rs12619285	2	213824045	ncRNA intronic	ENSG00000225332	Eosinophil counts	intergenic	19198610
rs12618573	2	240843638	intronic	ENSG00000130414	Response to amphetamines	intergenic	22952603
rs12617311	2	199632565	ncRNA intronic	ENSG00000225421	Menarche (age at onset)	intergenic	21102462
rs12604483	18	53855525	ncRNA exonic	ENSG00000206129	HIV-1 susceptibility	intergenic	21160409
rs12602978	17	25567080	ncRNA intronic	ENSG00000265246	Dental caries	intergenic	23064961
rs12592967	15	61870943	ncRNA intronic	ENSG00000259616	Schizophrenia	intergenic	22688191
rs12571093	10	70019371	promoter	ENSG00000234102	Optic disc size (disc)	intergenic	20395239
rs12570947	10	109569850	ncRNA intronic	ENSG00000229981	Prion diseases	intergenic	22210626
rs12565755	1	61041875	ncRNA intronic	ENSG00000226476	Response to tocilizumab in rheumatoid arthritis	intergenic	22491018
rs1256531	14	65747759	ncRNA exonic	ENSG00000259078	Conduct disorder (symptom count)	intergenic	20585324
rs12526186	6	30736151	ncRNA intronic	ENSG00000228022	Response to antipsychotic treatment	intergenic	19721433
rs12518099	5	89546109	ncRNA intronic	ENSG00000248555	Type 2 diabetes and other traits	intergenic	19734900
rs1247318	6	161333937	ncRNA intronic	ENSG00000220913, ENSG00000224371	Aging	intergenic	22773346
rs12447690	16	88298124	ncRNA intronic	ENSG00000261273	Central corneal thickness	intergenic	20719862, 20485516
rs12434047	14	53837485	ncRNA intronic	ENSG00000237356	Economic and political preferences (fairness)	intergenic	22566634
rs12431733	14	54290830	ncRNA	ENSG00000235269	Parkinson's	intergenic	19915575

			intronic		disease		
rs12423247	12	96858362	ncRNA intronic	ENSG00000258272	Phospholipid levels (plasma)	intergenic	22359512
rs1240773	11	116520527	ncRNA intronic	ENSG00000237937	HIV-1 susceptibility	intergenic	21160409
rs12393627	X	2885723	intronic	ENSG00000157399	Height	intergenic	22021425, 21998595
rs12388359	X	10231691	ncRNA intronic	ENSG00000227042	Alcohol dependence	intergenic	19581569
rs12328675	2	165540800	UTR3	ENSG00000082438	HDL cholesterol	downstream	20686565
rs12321565	12	68532077	ncRNA intronic	ENSG00000255733	Bipolar disorder and schizophrenia	intergenic	20889312
rs12274302	11	90586900	ncRNA exonic	ENSG00000261645	HIV-1 control	intergenic	20041166
rs12269901	11	116973929	ncRNA intronic	ENSG00000224077	Coronary heart disease	intergenic	21347282
rs12261589	10	79540835	ncRNA exonic	ENSG00000213513	Obesity-related traits	intergenic	23251661
rs1223271	20	13296912	intronic	ENSG00000089123	Parkinson's disease	intergenic	19915575
rs12206204	6	26116982	intronic	ENSG00000180596	Bilirubin levels	intergenic	19414484
rs12199775	6	143898894	intronic	ENSG00000112419	Inflammatory bowel disease	intergenic	23128233
rs12198063	6	77290799	ncRNA intronic	ENSG00000236635	Capecitabine sensitivity	intergenic	22864933
rs12155172	7	20994491	ncRNA intronic	ENSG00000232790	Prostate cancer	intergenic	19767753
rs12150660	17	7521915	intronic	ENSG00000129214	Testosterone levels;Sex hormone-binding globulin levels	intergenic	22829776, 21998597
rs12145922	1	89146234	ncRNA intronic	ENSG00000237505	Liver enzyme levels (gamma-glutamyl transferase)	intergenic	22001757
rs12143943	1	204572071	promoter	ENSG00000240710	Cognitive performance	intergenic	19734545
rs12130212	1	209727257	ncRNA intronic	ENSG00000224260	Obesity (extreme)	intergenic	21935397
rs12109285	5	21749348	ncRNA intronic	ENSG00000253766	Response to tocilizumab in rheumatoid arthritis	intergenic	22491018
rs12073837	1	221010205	ncRNA intronic	ENSG00000257551	F-cell distribution	intergenic	21326311
rs12073504	1	2452979	UTR3	ENSG00000157881	Obesity-related traits	intronic	23251661

rs1206397	2	51667739	ncRNA intronic	ENSG00000231918	Cognitive performance	intergenic	19734545
rs12035082	1	172898377	ncRNA intronic	ENSG00000224228	Crohn's disease	intergenic	17554261
rs12029080	1	95053353	ncRNA intronic	ENSG00000223675	D-dimer levels	intergenic	21502573
rs12025416	1	117038287	ncRNA intronic	ENSG00000224950, ENSG00000230381	Multiple sclerosis	intergenic	21244703
rs1200821	10	37559580	ncRNA intronic	ENSG00000240800	Hemostatic factors and hematological phenotypes	intergenic	17903294
rs11931074	4	90639515	ncRNA intronic	ENSG00000251095	Parkinson's disease	intergenic	19915576
rs1190739	X	136055295	ncRNA intronic	ENSG00000234062, ENSG00000241150	Coronary heart disease	intergenic	21347282
rs11889862	2	150697148	ncRNA intronic	ENSG00000162947, ENSG00000231969	Menopause (age at onset)	intergenic	19448619
rs11880316	19	31911129	ncRNA intronic	ENSG00000267553	Estradiol levels	intergenic	22675492
rs11870477	17	67802352	ncRNA intronic	ENSG00000227517	Response to TNF-alpha inhibitors in rheumatoid arthritis	intergenic	22569225
rs11865038	16	31095171	UTR3	ENSG00000167395	Parkinson's disease	intronic	22451204
rs11857380	15	58712203	UTR5	ENSG00000128918	Obesity-related traits	intergenic	23251661
rs11847697	14	30515112	ncRNA intronic	ENSG00000248975	Body mass index	intergenic	20935630
rs11845134	14	22782377	promoter	ENSG00000211819	Obesity-related traits	intergenic	23251661
rs11823543	11	116649135	UTR3	ENSG00000109917	Triglycerides-Blood Pressure (TG-BP)	downstream	21386085
rs11809789	1	82074852	ncRNA intronic	ENSG00000236676	Bilirubin levels	intergenic	22085899
rs11790994	9	98429266	ncRNA intronic	ENSG00000228142	Inattentive symptoms	intergenic	18821565
rs1178979	7	72856430	UTR3	ENSG00000009954	Triglycerides	intronic	20864672
rs11786458	8	40252701	ncRNA intronic	ENSG00000253354	Inattentive symptoms	intergenic	18821565
rs11786194	8	33673011	ncRNA intronic	ENSG00000253642	Immune response to smallpox vaccine (IL-6)	intergenic	22542470
rs11782819	8	10334781	ncRNA	ENSG00000253641	Alzheimer's	intergenic	20452100

			exonic		disease		
rs11781622	8	24378741	ncRNA intronic	ENSG00000253535	White matter integrity	intergenic	23218918
rs117616209	17	7142851	intronic	ENSG00000040633	Metabolite levels	upstream;downstream	22286219
rs11757063	6	96884886	ncRNA intronic	ENSG00000233797	Migraine	intergenic	22683712
rs11747270	5	150258867	intronic	ENSG00000237693	Crohn's disease	intergenic	18587394
rs11745587	5	131796922	UTR3	ENSG00000197536	Asthma	ncRNA intronic	22561531
rs11730243	4	106463713	ncRNA intronic	ENSG00000250522	Economic and political preferences (time)	intergenic	22566634
rs11724635	4	15737101	intronic	ENSG00000109743	Parkinson's disease	intergenic	21292315
rs11711441	3	182821275	intronic	ENSG00000078070	Parkinson's disease	intergenic	21292315
rs11696845	20	43371320	ncRNA intronic	ENSG00000244558	Obesity-related traits	intergenic	23251661
rs1168987	14	36397680	ncRNA intronic	ENSG00000258342	Response to antineoplastic agents	intergenic	21659360
rs11674248	2	24154192	intronic	ENSG00000173960	Obesity-related traits	intergenic	23251661
rs11662763	18	5857091	ncRNA intronic	ENSG00000261738	Immunoglobulin A;Immunoglobulin A	intergenic	20694011
rs11654749	17	69125606	ncRNA intronic	ENSG00000260785	Pulmonary function (interaction)	intergenic	23284291
rs11649653	16	30918487	ncRNA intronic	ENSG00000230447	Triglycerides	intergenic	20686565
rs11648796	16	792190	promoter	ENSG00000103245	Height	intergenic	20881960
rs11641231	16	86381184	ncRNA intronic	ENSG00000168367, ENSG00000269826	Response to statin therapy	intergenic	20339536
rs1163656	12	81337458	intronic	ENSG00000111058	Dental caries	intergenic	23259602
rs11626056	14	52233276	ncRNA exonic	ENSG00000258535	Hippocampal atrophy	intergenic	19668339
rs11624704	14	78786077	intronic	ENSG00000021645	Obesity	intergenic	21552555
rs11618202	13	31113379	intronic	ENSG00000189403	MRI atrophy measures	intergenic	21116278
rs11616188	12	6502742	promoter	ENSG00000256433	Ankylosing spondylitis	intergenic	21743469
rs11615916	12	62610860	intronic	ENSG00000198673	Pulmonary function decline	intergenic	22424883
rs11615274	12	73601132	ncRNA	ENSG00000258123	Body mass index	intergenic	22417934

			intronic		and cholesterol (psychopharmacological treatment)		
rs11613352	12	57792580	intronic	ENSG00000179912	Triglycerides;HDL cholesterol	intergenic	20686565
rs11610206	12	47639526	ncRNA intronic	ENSG00000257925	Alzheimer's disease	intergenic	19118814
rs11602954	11	202856	intronic	ENSG00000177951	Mean platelet volume;Bone mineral density	downstream	19820697, 22504420
rs1158867	2	128177377	UTR5	ENSG00000115718	Protein C levels	intronic	20802025
rs11581062	1	101407519	ncRNA exonic	ENSG00000230946	Multiple sclerosis	intronic	21833088
rs11564258	12	40792300	ncRNA intronic	ENSG00000258167	Crohn's disease;Inflammatory bowel disease	intergenic	21102463, 23128233
rs1154865	12	73989837	ncRNA intronic	ENSG00000258109	Breast cancer	intergenic	17903305
rs1148186	10	28617635	intronic	ENSG00000150054	Pulmonary function decline	intergenic	22424883
rs1144713	12	32261239	UTR3	ENSG00000151746	Obesity-related traits	intronic	23251661
rs11249215	1	25297184	ncRNA exonic	ENSG00000261025	Ankylosing spondylitis	intergenic	21743469
rs11245052	10	128267640	intronic	ENSG00000154493	Metabolite levels (MHPG)	intergenic	23319000
rs1124480	3	13857969	UTR3	ENSG00000154764	Chronic obstructive pulmonary disease-related biomarkers	intergenic	23144326
rs11239930	1	146560564	ncRNA intronic	ENSG00000227242	AIDS progression	intergenic	21502085
rs11226373	11	104334239	ncRNA intronic	ENSG00000256422	Matrix metalloproteinase levels	intergenic	20031604
rs11223996	11	134626887	ncRNA intronic	ENSG00000251226	Myopia (pathological)	intergenic	23049088
rs11191548	10	104846178	UTR3	ENSG00000076685	Systolic blood pressure;Blood pressure	intergenic	21909115, 21909110, 21572416, 19430483
rs11190140	10	101291593	promoter	ENSG00000257582	Ulcerative colitis;Crohn's disease	intergenic	20228799, 18587394
rs11175593	12	40601940	ncRNA	ENSG00000225342	Crohn's disease	intergenic	18587394

			intronic				
rs1117324	2	21840093	ncRNA intronic	ENSG00000233005	Response to antipsychotic treatment	intergenic	20195266
rs11171747	12	56518408	ncRNA intronic	ENSG00000258317	Systemic sclerosis	intergenic	21779181
rs11168351	12	48403765	ncRNA intronic	ENSG00000258203	Bipolar disorder and schizophrenia	intergenic	20889312
rs111649495	5	118356415	ncRNA intronic	ENSG00000249494	Response to amphetamines	intergenic	22952603
rs11163372	1	82247248	intronic	ENSG00000117114	Temperament	intergenic	22832960
rs11155133	6	141169825	ncRNA intronic	ENSG00000234147	Acute lymphoblastic leukemia (childhood)	intergenic	19684603
rs11155053	6	139660012	ncRNA exonic	ENSG00000218565	Obesity-related traits	intergenic	23251661
rs11150589	16	30482494	promoter	ENSG00000222701	Ulcerative colitis	intergenic	23128233
rs11139399	9	84372741	ncRNA intronic	ENSG00000233926	Hippocampal atrophy	intergenic	22745009
rs11118620	1	221028508	ncRNA intronic	ENSG00000257551	Heart failure	intergenic	20445134
rs11113894	12	108841863	ncRNA intronic	ENSG00000247213	Obesity-related traits	intergenic	23251661
rs1106766	12	57809456	intronic	ENSG00000179912	Urate levels	intergenic	20884846
rs11066453	12	113365621	ncRNA intronic	ENSG00000257452	Gamma glutamyl transpeptidase	intergenic	21909109
rs11059374	12	128304418	ncRNA intronic	ENSG00000256922	Response to amphetamines	intergenic	22952603
rs11052552	12	9855958	promoter	ENSG00000256582	Type 1 diabetes	intergenic	17554300
rs11036238	11	5225635	promoter	ENSG00000224091	Malaria	intergenic	19465909
rs11009175	10	33294775	ncRNA intronic	ENSG00000229656	Depression (quantitative trait)	intergenic	20800221
rs10988449	9	132370360	promoter	ENSG00000148335	Response to antidepressant treatment	intergenic	22041458
rs10984561	9	122258577	intronic	ENSG00000260970	Pulmonary function decline	intergenic	22424883
rs10956483	8	130572110	ncRNA intronic	ENSG00000229140	White blood cell types	intergenic	21738478
rs10948172	6	44777691	intronic	ENSG00000196284	Osteoarthritis	intergenic	22763110
rs10946808	6	26233387	ncRNA	ENSG00000218281	Height	intergenic	19343178,

			exonic				18391951, 18391950
rs10937275	3	186650790	ncRNA exonic	ENSG00000203632	Drug-induced liver injury (flucloxacillin)	intronic	19483685
rs10936797	3	174194243	intronic	ENSG00000177694	Obesity-related traits	intergenic	23251661
rs10936632	3	170130102	ncRNA intronic	ENSG00000242578	Prostate cancer	intergenic	21743467
rs10929808	2	12568996	ncRNA intronic	ENSG00000224184	Bipolar disorder and schizophrenia	intergenic	20889312
rs10915864	1	225901006	ncRNA intronic	ENSG00000226349, ENSG00000227496	Obesity-related traits	intergenic	23251661
rs10905868	10	10974256	ncRNA intronic	ENSG00000229240	Obesity-related traits	intergenic	23251661
rs10900020	10	44827197	intronic	ENSG00000107562	Schizophrenia	intergenic	23212062
rs10892279	11	118611781	ncRNA intronic	ENSG00000255422	Celiac disease and Rheumatoid arthritis	intergenic	21383967
rs10888935	1	56060951	ncRNA intronic	ENSG00000234810	Inflammatory biomarkers	intergenic	22228203
rs10883365	10	101287764	ncRNA exonic	ENSG00000228778, ENSG00000257582	Crohn's disease	intergenic	17554300, 17554261
rs10876864	12	56401085	promoter	ENSG00000123411	Vitiligo	intergenic	22951725
rs10876550	12	54712308	ncRNA intronic	ENSG00000258344	Mean platelet volume	intergenic	22139419
rs10876432	12	53731891	intronic	ENSG00000170374	Bone mineral density (spine)	intergenic	19079262
rs10865035	2	100835734	ncRNA intronic	ENSG00000232084	Rheumatoid arthritis	intergenic	20453842
rs10852932	17	2143460	UTR5	ENSG00000070366	Aortic root size	intronic	19584346
rs10849605	12	1064438	intronic	ENSG00000002016	Lung cancer	intergenic	22899653
rs1084651	6	161089817	ncRNA intronic	ENSG00000243831	HDL cholesterol	intergenic	20686565
rs10844154	12	32380501	ncRNA exonic	ENSG00000258134	Weight;Emphysema-related traits	intronic	20709820, 19851299
rs10841496	12	20521654	ncRNA intronic	ENSG00000256879	Male infertility	upstream	19478329
rs10841397	12	20063968	ncRNA intronic	ENSG00000255910	Response to amphetamines	intergenic	22952603
rs10841287	12	19779793	intronic	ENSG00000139154	Obesity-related traits	intergenic	23251661
rs10830962	11	92698427	promoter	ENSG00000254874	Metabolite levels;Metabolic syndrome;Diabetes (gestational)	intergenic	22399527, 22233651, 21909109

rs10812428	9	26614847	promoter	ENSG00000266429	Drug-induced liver injury (flucloxacillin)	intergenic	19483685
rs10805321	4	13914373	ncRNA intronic	ENSG00000250634	Response to antipsychotic therapy (extrapyramidal side effects)	intergenic	21990027
rs10797432	1	2501338	ncRNA intronic	ENSG00000225931	Ulcerative colitis	intergenic	23128233
rs10792665	11	82649768	intronic	ENSG00000137509, ENSG00000165490	Obesity-related traits	intergenic	23251661
rs10778213	12	103495151	ncRNA intronic	ENSG00000257703	C-reactive protein	intergenic	18439548
rs10776733	1	112107669	intronic	ENSG00000116473	Obesity-related traits	intergenic	23251661
rs10774214	12	4368352	ncRNA intronic	ENSG00000255920, ENSG00000256164	Colorectal cancer	intergenic	23263487
rs10745954	12	103483094	ncRNA intronic	ENSG00000257703	C-reactive protein	intergenic	21300955
rs10744304	12	127813741	ncRNA intronic	ENSG00000256362	Depression (quantitative trait)	intergenic	20800221
rs10743889	12	10521389	ncRNA intronic	ENSG00000245648	Obesity-related traits	intergenic	23251661
rs10740609	10	56790969	intronic	ENSG00000150275	Weight	intergenic	20966902
rs1055129	17	73872948	ncRNA exonic	ENSG00000267801	White matter hyperintensity burden	intronic	21681796
rs10520045	15	36313965	ncRNA intronic	ENSG00000259639	Major depressive disorder	intergenic	20673876
rs10519131	15	62001132	ncRNA intronic	ENSG00000259675	Parkinson's disease	intergenic	22451204
rs10516541	4	108115222	intronic	ENSG00000155011	Mean forced vital capacity from 2 exams	intergenic	17903307
rs10513025	5	9623622	ncRNA exonic	ENSG00000248525	Autism	intergenic	19812673
rs10511378	3	118162623	ncRNA intronic	ENSG00000243276	Obesity-related traits	intergenic	23251661
rs10510634	3	30346968	promoter	ENSG00000199927	Fasting plasma glucose	intergenic	17903298
rs10510138	10	126438019	intronic	ENSG00000203791, ENSG00000258539	Obesity-related traits	intergenic	23251661
rs10506821	12	80496923	ncRNA exonic	ENSG00000230291	Hip geometry	intergenic	17903296
rs10505477	8	128407443	ncRNA	ENSG00000246228	Colorectal	intergenic	17618283

			intronic		cancer		
rs10503951	8	33665680	ncRNA intronic	ENSG00000253642	Immune response to smallpox vaccine (IL-6)	intergenic	22542470
rs10501320	11	47293799	UTR5	ENSG00000110514	Proinsulin levels	intronic	21873549
rs10500569	16	72756101	ncRNA intronic	ENSG00000259768	Metabolite levels	intergenic	22916037
rs10499504	7	17561583	ncRNA intronic	ENSG00000226598	Response to antipsychotic treatment	intergenic	20195266
rs10496166	2	69063909	ncRNA exonic	ENSG00000228329	RR interval (heart rate)	intergenic	20031603
rs10490113	2	59499347	ncRNA intronic	ENSG00000222030, ENSG00000233891	Breast cancer	intergenic	17903305
rs10489087	4	13794416	ncRNA intronic	ENSG00000250634	Hemostatic factors and hematological phenotypes	intergenic	17903294
rs10485165	6	89112817	ncRNA intronic	ENSG00000234426	Select biomarker traits	intergenic	17903293
rs10484128	14	98642572	ncRNA intronic	ENSG00000259097	Hemostatic factors and hematological phenotypes	intergenic	17903294
rs10483727	14	61072875	promoter	ENSG00000253014	Vertical cup-disc ratio;Optic disc size (rim);Glaucoma (primary open-angle)	intergenic	22570617, 22419738, 20548946, 20395239
rs10475598	5	173648438	intronic	ENSG00000170091	Information processing speed	intergenic	21130836
rs10472828	5	32888818	promoter	ENSG00000251062	Height	intergenic	19570815, 19343178
rs10468017	15	58678512	intronic	ENSG00000128918	Phospholipid levels (plasma);Metabolic syndrome (bivariate traits);HDL cholesterol;Cardiovascular disease risk factors;Age-related macular degeneration	intergenic	22359512, 21943158, 21665990, 21386085, 20385826, 19060906
rs10455657	6	68984930	ncRNA	ENSG00000226497	Cannbis use	intergenic	22823124

			intronic		(initiation)		
rs10423754	19	54604822	intronic	ENSG00000170909	Response to taxane treatment (docetaxel)	upstream	23006423
rs10411161	19	52372976	intronic	ENSG00000161551	Breast cancer	intergenic	21424380
rs10404998	19	22608374	ncRNA intronic	ENSG00000269796	Dental caries	intergenic	23259602
rs1039002	6	166155457	ncRNA intronic	ENSG00000256956	Bipolar disorder	intergenic	22205951
rs1037757	18	56752054	ncRNA exonic	ENSG00000267215	Alzheimer's disease (age of onset)	intergenic	22005931
rs1027643	5	91893792	ncRNA intronic	ENSG00000249776	Wilms tumor	intergenic	22544364
rs1023252	1	11899033	exonic	ENSG00000011021	Natriuretic peptide levels	intronic	21273288
rs10229603	7	112628373	ncRNA intronic	ENSG00000234520	Attention deficit hyperactivity disorder and conduct disorder	intergenic	18951430
rs10227331	7	157294938	ncRNA intronic	ENSG00000223872	Inattentive symptoms	intergenic	18821565
rs10212363	3	101848561	intronic	ENSG00000170044	Waist-to-hip circumference ratio (interaction)	intergenic	23192594
rs10210302	2	234158839	intronic	ENSG00000085978	Crohn's disease	intergenic	17554300
rs10187424	2	85794297	intronic	ENSG00000118640	Prostate cancer	intergenic	21743467
rs1018326	2	182007800	ncRNA intronic	ENSG00000234663	Ankylosing spondylitis	intergenic	20062062
rs10172965	2	195871819	ncRNA intronic	ENSG00000235056	Temperament	intergenic	22832960
rs10170236	2	150457624	ncRNA intronic	ENSG00000231969	Acute lymphoblastic leukemia (childhood)	intergenic	23007406
rs1016343	8	128093297	ncRNA intronic	ENSG00000253264	Prostate cancer	intergenic	21743057, 18264097
rs10162002	13	24042510	ncRNA intronic	ENSG00000232977	Hypothyroidism	intergenic	22493691
rs1015213	8	52887541	ncRNA intronic	ENSG00000253844	Glaucoma (primary open-angle)	intergenic	22922875
rs1015164	3	46451680	ncRNA intronic	ENSG00000240310	AIDS progression	downstream	21502085
rs1013063	21	35698390	ncRNA intronic	ENSG00000214955	Body mass index (interaction)	intergenic	23192594
rs10125054	9	114798037	ncRNA	ENSG00000259953	Obesity-related	ncRNA	23251661

			exonic		traits	intronic	
rs10104895	8	61876044	ncRNA intronic	ENSG00000254777	Pulmonary function decline	intergenic	22424883
rs10092658	8	130980472	intronic	ENSG00000153310	Protein quantitative trait loci	intergenic	18464913
rs10091374	8	71386904	ncRNA intronic	ENSG00000253143, ENSG00000253967	Cardiac Troponin-T levels	intergenic	23247143
rs10088218	8	129543949	ncRNA intronic	ENSG00000254275	Ovarian cancer	intergenic	20852632
rs10055544	5	91518721	ncRNA intronic	ENSG00000250049	Immune reponse to smallpox (secreted IL-10)	intergenic	22610502
rs10048158	17	64236318	intronic	ENSG00000091583	β2-Glycoprotein I (β2-GPI) plasma levels	intergenic	23279374
rs10037512	5	88354675	ncRNA intronic	ENSG00000248309	Height	intergenic	20881960
rs10030601	4	150725212	ncRNA intronic	ENSG00000234828	Epilepsy (generalized)	intergenic	22949513
rs1000597	7	30937178	intronic	ENSG00000240583, ENSG00000250424	Nephrolithiasis	intergenic	22396660
rs10005603	4	105844273	ncRNA intronic	ENSG00000248242, ENSG00000248373	Tuberculosis	intergenic	20694014
rs1000113	5	150240076	intronic	ENSG00000237693	Crohn's disease	intergenic	17554300

Table S3. Relocated GWAS SNPs to UCSC genes

GWAS SNPID	Chr	Coordinate	Relocated region	Relocated UCSC gene	Associated disease/trait	Original RefSeq region	PubmedID
rs9331888	8	27468862	UTR5	CLU	Alzheimer's disease	intronic	19734903
rs12627933	22	32027449	intronic	PISD	Intelligence	upstream	22449649
rs9621305	22	32049917	intronic	PISD	Intelligence	intergenic	22449649
rs6088735	20	33745676	intronic	EDEM2	Hemostatic factors and hematological phenotypes	intergenic	22443383
rs6060278	20	33753262	intronic	EDEM2	Hemostatic factors and hematological phenotypes	intergenic	22443383
rs12091564	1	145395604	promoter	TRNA Lys	Coronary heart disease	intergenic	21626137
rs9995093	4	89220944	ncRNA intronic	BC027846	Bipolar disorder	intergenic	22925353
rs9913711	17	70098161	ncRNA intronic	AK094963, AL833139	Liver enzyme levels (gamma-glutamyl transferase)	intergenic	22001757
rs9866825	3	8250790	ncRNA intronic	LOC100288428	QT interval	intergenic	23166209
rs9844666	3	135974216	UTR5	PCCB	Height	intronic	20881960
rs9824150	3	162834301	ncRNA intronic	BC073807	Capecitabine sensitivity	intergenic	22864933
rs9810890	3	128652553	intronic	AK125726, KIAA1257	Dental caries	intergenic	23064961
rs9807989	2	102971200	intronic	IL18R1	Asthma	intergenic	22561531
rs9805786	13	24658356	intronic	SPATA13	Depression and alcohol dependence	intergenic	22064162
rs9803659	1	167156500	ncRNA intronic	RBSG4	Liver enzyme levels	intergenic	18940312
rs972275	6	127391844	ncRNA intronic	AK127472	Iron status biomarkers	intergenic	19084217
rs9653442	2	100825367	ncRNA intronic	BC105019	Type 1 diabetes	intergenic	17554260
rs9652490	15	77963887	intronic	LINGO1	Essential tremor	intergenic	19182806
rs9642880	8	128718068	ncRNA intronic	BC042052	Urinary bladder cancer;Bladder cancer	intergenic	20972438, 20348956, 18794855
rs9604529	13	114622597	exonic	FLJ44054	Response to tocilizumab in rheumatoid arthritis	intergenic	22491018
rs9596270	13	50842440	ncRNA intronic	BCMS,DLEU1	Multiple sclerosis	intergenic	22190364
rs958546	13	46833717	intronic	LRRC63	Atrial fibrillation	intergenic	17903304
rs954820	10	133957761	UTR5	JAKMIP3	Chronic obstructive pulmonary disease-related biomarkers	intronic	23144326

rs9488363	6	114591986	ncRNA intronic	BC042098	Optic disc size (rim)	intergenic	20395239
rs9485372	6	149608874	intronic	TAB2	Breast cancer	intergenic	22383897
rs9468925	6	31258837	intronic	HLA-B	Vitiligo	intergenic	20526339
rs9461688	6	31271716	intronic	HLA-B	Protein quantitative trait loci	intergenic	18464913
rs9402515	6	133860345	ncRNA intronic	BC041459	Protein quantitative trait loci	intergenic	18464913
rs9387478	6	117786180	intronic	GOPC	Lung cancer	intergenic	23143601
rs9375969	6	133867802	ncRNA intronic	BC041459	Visceral fat	intergenic	22589738
rs9368677	6	31272321	intronic	HLA-B	Atopic dermatitis	intergenic	23042114
rs9357155	6	32809848	UTR3	PSMB8	Nephropathy	intronic	21399633
rs9316500	13	51094114	ncRNA intronic	BCMS,DLEU1	Pulmonary function decline	intergenic	22424883
rs9305354	21	29475196	ncRNA intronic	AX747935	Urinary albumin excretion	intergenic	17903292
rs930421	2	42981239	UTR3	MTA3	Attention deficit hyperactivity disorder	intergenic	18821565
rs9302001	13	95463392	ncRNA intronic	BC045767	Panic disorder	intergenic	19165232
rs9267663	6	31867253	intronic	C2	Economic and political preferences (environmentalism)	downstream	22566634
rs9264942	6	31274380	intronic	HLA-B	HIV-1 control;Crohn's disease	intergenic	21051598, 20041166, 23128233
rs9262632	6	31024808	ncRNA exonic	HCG22	HIV-1 control	ncRNA intronic	21051598
rs9260489	6	29920332	intronic	HLA-G,HLA-J	Multiple sclerosis	intergenic	22190364
rs925255	2	28614794	ncRNA intronic	AK055918	Inflammatory bowel disease	upstream	23128233
rs911119	20	23612737	intronic	CST3	Chronic kidney disease	intergenic	20383146
rs890835	5	175956271	exonic	RNF44	Menopause (age at onset)	intronic	22267201
rs886424	6	30782002	exonic	AK098012	Bipolar disorder and schizophrenia	intergenic	22688191
rs871606	4	54799245	intronic	PDGFRA	Blood pressure	intergenic	21909110
rs870288	16	5585852	intronic	BC108660	Subcutaneous adipose tissue;Phospholipid levels (plasma)	intergenic	22589738, 22359512
rs854572	7	94954696	intronic	PON1	Paraoxonase activity	upstream	22982463
rs823156	1	205764640	UTR5	SLC41A1	Parkinson's disease	intronic	21738487
rs8127571	21	47165429	intronic	PCBP3	Immune reponse to smallpox (secreted IFN-alpha)	intergenic	22610502
rs8070473	17	33843512	intronic	SLFN12L	Depression (quantitative	intergenic	20800221

					trait)		
rs8068318	17	59483766	UTR3	TBX2	Creatinine levels	intronic	20383145
rs806321	13	50841323	ncRNA intronic	BCMS,DLEU1	Multiple sclerosis	intergenic	21833088
rs8033165	15	29006093	ncRNA intronic	WHAMMP2	Black vs. red hair color;Black vs. blond hair color	intergenic	18483556
rs7961581	12	71663102	intronic	TSPAN8	Type 2 diabetes	intergenic	18372903
rs789560	12	70331827	intronic	C12orf28	Attention deficit hyperactivity disorder and conduct disorder	intergenic	18951430
rs7861820	9	108936674	ncRNA intronic	BC039487	Menarche and menopause (age at onset)	intergenic	19448621
rs7815944	8	129427518	ncRNA intronic	BC009730	Atopic dermatitis	intergenic	23042114
rs7801190	7	100458093	UTR5	SLC12A9	Coronary heart disease	intronic	21347282
rs7772131	6	30795171	intronic	AK098012	Response to angiotensin II receptor blocker therapy	intergenic	22566498
rs7705924	5	101946798	ncRNA intronic	AX747345	Crohn's disease	intergenic	22412388
rs7665590	4	99796784	promoter	AK098333	Primary biliary cirrhosis	intergenic	23000144
rs7656416	4	1254535	ncRNA intronic	C4orf42	Type 2 diabetes	intergenic	22456796
rs7616215	3	46205686	intronic	CCR3	Behcet's disease	intergenic	23291587
rs7612209	3	177596989	ncRNA intronic	AK056252	Cognitive performance	intergenic	19734545
rs7602460	2	182261869	ncRNA intronic	AK125001	Atrioventricular conduction	intergenic	21041692
rs7570469	2	79709354	intronic	CTNNA2	Response to antipsychotic treatment	intergenic	20195266
rs7552393	1	84254551	ncRNA intronic	BC036594	Select biomarker traits	intergenic	17903293
rs7539409	1	84254735	ncRNA intronic	BC036594	Alzheimer's disease	intergenic	20061627
rs740363	10	118575606	intronic	HSPA12A	Heart failure	intergenic	17903304
rs7359397	16	28885659	intronic	NPIPL1	Body mass index	downstream	20935630
rs7337573	13	61513669	ncRNA intronic	AK097816	Phospholipid levels (plasma)	intergenic	22359512
rs7318731	13	22709670	ncRNA intronic	AK054845	RR interval (heart rate)	intergenic	20031603
rs7296418	12	123457619	intronic	ABCB9	Platelet counts	intergenic	21507922
rs72960926	6	75158266	ncRNA intronic	AF086303	Metabolite levels (MHPG)	intergenic	23319000
rs727979	6	149593920	intronic	TAB2	Hemostatic factors and hematological phenotypes	intergenic	17903294
rs727153	4	155654421	ncRNA	DQ266889	Alzheimer's disease	intergenic	18823527

			intronic				
rs72669744	1	56116505	intronic	AK127270	Lipid metabolism phenotypes	intergenic	22286219
rs7260598	19	24222786	ncRNA intronic	AK092080, AK092150	Response to taxane treatment (placlitaxel)	intergenic	23006423
rs7253363	19	11682495	ncRNA intronic	BC039523	Multiple sclerosis (severity)	intergenic	19010793
rs7181753	15	96844727	ncRNA intronic	AK000872, AK307134	Electroencephalographic traits in alcoholism	intergenic	22554406
rs7175404	15	94036688	ncRNA intronic	AK094352	Attention deficit hyperactivity disorder	intergenic	18839057
rs7141238	14	61734640	intronic	PRKCH	Obesity-related traits	intergenic	23251661
rs7137869	12	119989646	ncRNA intronic	AF086288	Aging traits	intergenic	17903295
rs7121446	11	121954657	ncRNA intronic	BC089451	Cardiovascular disease risk factors	intergenic	20838585
rs7105934	11	69239741	promoter	AK094674	Renal cell carcinoma	intergenic	21131975
rs7105056	11	112064431	UTR5	BCO2	Immune reponse to smallpox (secreted IL-12p40)	intronic	22610502
rs705471	10	3667726	ncRNA intronic	BC037918	Capecitabine sensitivity	intergenic	22864933
rs705469	10	3658919	ncRNA intronic	BC037918	Capecitabine sensitivity	intergenic	22864933
rs7014346	8	128424792	ncRNA intronic	DQ515898, DQ515899, LOC727677	Colorectal cancer	intergenic	18372901
rs6983267	8	128413305	ncRNA intronic	DQ515898, DQ515899, LOC727677	Prostate cancer;Colorectal cancer	intergenic	21743057, 21242260, 18372905, 18264097, 18264096, 17618284, 17401363, 23266556
rs6972204	7	2582452	UTR3	BRAT1	Obesity-related traits	intronic	23251661
rs6952808	7	1886535	ncRNA exonic	AK127048	Bipolar disorder and schizophrenia	intronic	20889312
rs6951258	7	37757556	ncRNA intronic	BC043356	Obesity-related traits	intergenic	23251661
rs6932590	6	27248931	promoter	TRNA Val	Schizophrenia	intergenic	19571808
rs6927022	6	32612397	UTR3	HLA-DQA1	Ulcerative colitis	downstream	23128233
rs6912405	6	74871231	ncRNA intronic	AF086303	Obesity-related traits	intergenic	23251661
rs6846071	4	102402215	intronic	BANK1	C-reactive protein and white blood cell count	intergenic	22788528

rs6782299	3	180550702	ncRNA intronic	DKFZp434 A128	Schizophrenia	intergenic	19571811
rs6775745	3	131813900	intronic	CPNE4	Neutrophil count	intergenic	21507922
rs6751715	2	56363377	ncRNA intronic	BC043355	HIV-1 control	intergenic	20041166
rs6740462	2	65667272	ncRNA intronic	FLJ16124	Inflammatory bowel disease	intergenic	23128233
rs6720394	2	111989372	ncRNA intronic	LOC54147 1	Primary sclerosing cholangitis	intergenic	21151127
rs6699417	1	89123443	ncRNA intronic	AK123834	Height	intergenic	20881960
rs6687840	1	159342439	ncRNA intronic	BC038194	Chemerin levels	intergenic	20237162
rs6604026	1	93303603	ncRNA exonic	SNORA66	Multiple sclerosis	intronic	19525955, 17660530
rs6586513	1	17216331	intronic	CROCC	Allergic rhinitis	intergenic	22036096
rs6584283	10	101290301	ncRNA intronic	DQ372722	Ulcerative colitis	intergenic	21297633, 20228798, 19915572
rs6560517	9	79038170	intronic	GCNT1	Dialysis-related mortality	intergenic	21546767
rs6556756	5	163889280	ncRNA intronic	BC011998	Breast cancer	intergenic	17903305
rs6544997	2	47844505	intronic	MSH2	Vitiligo	intergenic	19890347
rs6503659	17	39897264	intronic	JUP	Esophageal cancer (squamous cell)	intergenic	22960999
rs6484218	11	10390581	ncRNA intronic	EF537580	Schizophrenia, bipolar disorder and depression (combined)	intergenic	20713499
rs6479891	10	65006456	ncRNA exonic	AX747628	Arthritis (juvenile idiopathic)	intronic	22354554
rs6444931	3	170163474	intronic	CLDN11	Bipolar disorder and schizophrenia	intergenic	20889312
rs6442925	3	5013876	intronic	LOC10050 7582	Corneal structure	intergenic	21979947
rs6441286	3	159728878	ncRNA intronic	AK097161	Primary biliary cirrhosis	intergenic	20639880, 19458352
rs6428370	1	196844593	intronic	CFHR4	Acute lymphoblastic leukemia (childhood)	intergenic	19684603
rs623323	17	700020	ncRNA exonic	AX748314	Type 2 diabetes	intergenic	23300278
rs61744862	17	17068182	UTR5	MPRIP	Obesity-related traits	intronic	23251661
rs6088765	20	33799280	intronic	EDEM2	Ulcerative colitis	intergenic	23128233
rs6045676	20	1941171	ncRNA intronic	AK090681	Aortic root size	intergenic	21223598
rs6027511	20	58898209	ncRNA intronic	AK309218	Lentiform nucleus volume;Lentiform nucleus volume	downstream	22903471

rs600550	11	59997666	promoter	AB231729, AB231731, MS4A4E	Lipoprotein-associated phospholipase A2 activity and mass	intergenic	23118302
rs5934953	X	11048692	ncRNA intronic	AY660577, AY660578	Cognitive performance	intergenic	19734545
rs5767992	22	48188253	ncRNA intronic	AK093107	Obesity-related traits	intergenic	23251661
rs565229	11	122189465	ncRNA intronic	MIR100HG	Hemostatic factors and hematological phenotypes	intergenic	17903294
rs55681231	20	58946489	ncRNA intronic	AK309218	Metabolite levels (MHPG)	intergenic	23319000
rs543686	15	35068309	ncRNA intronic	AK092087	Prostate cancer (gene x gene interaction)	intergenic	22219177
rs527409	1	58757915	intronic	DAB1	Kawasaki disease	intergenic	21221998
rs525455	10	13103285	ncRNA intronic	AK311458	Platelet aggregation	intergenic	20526338
rs4955755	3	170494409	intronic	CLDN11	Menopause (age at onset)	intergenic	19448619
rs4954218	2	135803425	intronic	YSK4	Corneal structure	intergenic	21979947
rs4938534	11	111275133	ncRNA intronic	BC022056	Primary biliary cirrhosis	intergenic	23000144
rs4910742	11	5306509	intronic	HBE1	Inflammatory biomarkers;Fetal hemoglobin levels	intergenic	22291609, 18245381
rs4872511	8	22400989	ncRNA exonic	AK125860	HIV-1 control	intergenic	20205591
rs4864201	4	130731284	ncRNA intronic	BC041448	Obesity	intergenic	22484627
rs485499	3	159745863	ncRNA intronic	AK097161	Primary biliary cirrhosis	intergenic	21399635
rs4845812	1	5707816	ncRNA intronic	AK125078	Metabolite levels (MHPG)	intergenic	23319000
rs4819143	21	47124178	intronic	PCBP3	Insulin resistance/response	intergenic	21901158
rs4800279	18	25070355	ncRNA intronic	AK127888	White matter integrity	intergenic	23218918
rs4792394	17	13684917	ncRNA intronic	AK123263	Conduct disorder (symptom count)	intergenic	20585324
rs4788084	16	28539848	intronic	NPIPL1	Type 1 diabetes autoantibodies;Type 1 diabetes	intergenic	21829393, 19430480
rs4777845	15	93877425	ncRNA intronic	AK094352	Adiponectin levels	intergenic	22479202
rs4760790	12	71634794	intronic	TSPAN8	Type 2 diabetes	intergenic	20581827
rs4745062	9	73784264	intronic	TRPM3	Longevity	intergenic	20834067
rs4716055	6	9853919	intronic	MRDS1,OFCC1	Adiponectin levels	intergenic	22479202

rs4704970	5	155500992	intronic	SGCD	Multiple sclerosis (age of onset)	intergenic	19010793
rs4693646	4	84721814	ncRNA intronic	BC005018	Obesity-related traits	intergenic	23251661
rs4684585	3	883851	ncRNA intronic	AK126307	Response to statin therapy	intergenic	20339536
rs4680534	3	159698945	ncRNA intronic	AK097161	Multiple sclerosis	intergenic	19525953
rs4657616	1	158971086	intronic	IFI16	Hematology traits	intergenic	23263863
rs4656784	1	159326880	ncRNA intronic	BC038194	IgE levels;IgE levels	intergenic	22075330
rs4549631	6	126966308	ncRNA intronic	AK127472	Height	intergenic	18391952
rs445114	8	128323181	ncRNA intronic	DQ515898, DQ515899, LOC727677	Prostate cancer	intergenic	21743057, 19767754
rs4446959	1	159357684	ncRNA intronic	BC038194	Chemerin levels	intergenic	20237162
rs4410871	8	128815029	ncRNA intronic	PVT1	Multiple sclerosis	intergenic	21833088
rs4324798	6	28776117	promoter	TRNA Phe	Lung adenocarcinoma	intergenic	19836008
rs4281086	8	10352308	ncRNA intronic	AK307207	Obesity-related traits	intergenic	23251661
rs4273712	6	126964510	ncRNA intronic	AK127472	Intracranial volume	intergenic	22504418
rs4262150	5	152288453	ncRNA intronic	AK123816	Bipolar disorder and schizophrenia	intergenic	22688191
rs4236601	7	116162729	intronic	CAV1	Glaucoma (primary open-angle)	intergenic	20835238
rs4149178	6	43272188	UTR3	SLC22A7	Urate levels	intronic	23263486
rs4142248	19	44888276	intronic	ZFP112	Myopia (pathological)	intergenic	23049088
rs4128725	1	159405959	ncRNA intronic	BC038194	Select biomarker traits	intergenic	17903293
rs404860	6	32184345	UTR3	NOTCH4	Asthma	intronic	21804548
rs400028	5	113849294	ncRNA intronic	AK097686	Obesity-related traits	intergenic	23251661
rs399885	2	79687252	intronic	CTNNA2	Response to antipsychotic treatment	intergenic	20195266
rs399604	6	32975014	exonic	HLA-DOA	Platelet counts	intronic	22139419
rs389884	6	31940897	UTR3	STK19	Hematology traits	intronic	23263863
rs3893464	6	29935250	intronic	HLA-G,HLA-J	Graves' disease	intergenic	21900946
rs386000	19	54792761	intronic	LILRA6	HDL cholesterol	intergenic	20686565
rs3849491	3	76484183	intronic	ROBO2	Sex hormone-binding globulin levels	intergenic	22675492
rs3845817	2	65758525	ncRNA intronic	FLJ16124	Bipolar disorder	intergenic	21926972

rs3820201	1	53581670	ncRNA exonic	AX748428	Hippocampal atrophy	intronic	22745009
rs3803064	12	113173494	intronic	RPH3A	Platelet counts	intergenic	21507922
rs3761218	20	3776175	intronic	CDC25B	Bipolar disorder	upstream	21254220, 17554300
rs3741920	12	6938872	UTR5	LEPREL2	Response to Vitamin E supplementation	intronic	22437554
rs37060	16	58566304	UTR5	CNOT1	QT interval	intronic	22726844
rs361433	7	142104571	intronic	TCRB,TCRBV22S1A2N1T,TCRBV5S1A1T	Capecitabine sensitivity	intergenic	22864933
rs342296	7	106372903	ncRNA intronic	AF086203	Mean platelet volume	intergenic	22423221
rs342293	7	106372219	ncRNA intronic	AF086203	Mean platelet volume	intergenic	22423221, 22139419, 19820697, 19221038
rs342275	7	106359216	ncRNA intronic	AF086203	Platelet counts	intergenic	22139419
rs340849	1	214118090	ncRNA intronic	PROX1-AS1	Alzheimer's disease	intergenic	22159054
rs314370	7	100453208	UTR5	SLC12A9	Resting heart rate	intronic	20639392
rs3134792	6	31312326	intronic	HLA-B	Psoriasis	intergenic	18364390
rs3130320	6	32223258	promoter	AK123889	Systemic lupus erythematosus	intergenic	21408207
rs3126085	1	152300817	ncRNA intronic	AK056431	Atopic dermatitis	intergenic	21666691
rs3123078	10	51524971	intronic	PARG,TIMM23,TIMM23B	Prostate cancer	intergenic	19767753
rs3120665	1	152316590	ncRNA intronic	AK056431	Personality dimensions	intergenic	20691247
rs3118914	13	51116901	ncRNA intronic	BCMS	Height	intergenic	19343178
rs3118905	13	51105334	ncRNA intronic	BCMS	Height	intergenic	20881960
rs3117035	6	33086249	exonic	HLA-DPB2	RR interval (heart rate)	ncRNA intronic	20031603
rs3116602	13	51111355	ncRNA intronic	BCMS	Height	intergenic	18391952
rs3110496	17	27917771	intronic	ANKRD13B	Height	intergenic	20881960
rs297941	12	50319086	ncRNA exonic	BC034605	Obsessive-compulsive disorder	intergenic	22889921
rs2969775	2	47885476	intronic	MSH2	Alzheimer's disease	intergenic	22005930
rs2923084	11	10388782	ncRNA intronic	EF537580	HDL cholesterol	intergenic	20686565

rs2904804	10	5009759	UTR3	AKR1C1	Economic and political preferences (immigration/crime)	intronic	22566634
rs2894207	6	31263751	intronic	HLA-B	Nasopharyngeal carcinoma	intergenic	20512145
rs2877832	14	27800177	ncRNA intronic	BC148262	Diabetes related insulin traits	intergenic	17903298
rs2860580	6	29906691	ncRNA intronic	AK097625	Nasopharyngeal carcinoma	intergenic	20512145
rs2762051	13	50835715	ncRNA intronic	BCMS,DLEU1	Celiac disease	intergenic	20190752
rs2755237	13	41109429	intronic	FOXO1	Central corneal thickness	intergenic	20719862
rs2739330	22	24295286	intronic	DDT	Liver enzyme levels (gamma-glutamyl transferase)	intergenic	22001757
rs2726807	4	183137398	intronic	ODZ3	Schizophrenia	intergenic	22885689
rs2721051	13	41110884	intronic	FOXO1	Central corneal thickness;Corneal structure	intergenic	20485516, 23291589
rs260461	19	58770883	ncRNA exonic	BC063675	Attention deficit hyperactivity disorder	intronic	18821565
rs2596542	6	31366595	promoter	MICA	Hepatocellular carcinoma	intergenic	21499248
rs2571391	6	29923838	intronic	HLA-G,HLA-J	IgE levels;IgE levels	intergenic	22075330
rs2562456	19	21666210	ncRNA exonic	LOC400680	Pain	upstream	19207018
rs2524054	6	31252396	intronic	HLA-B	CD4:CD8 lymphocyte ratio	intergenic	20045101
rs2524005	6	29899677	ncRNA intronic	AK097625	Bipolar disorder and schizophrenia	intergenic	22688191
rs2523946	6	29941943	intronic	HLA-G,HLA-J	IgA nephropathy	upstream	22197929
rs2523822	6	29828660	intronic	HLA-G,HLA-H	Drug-induced liver injury (amoxicillin-clavulanate)	intergenic	21570397
rs2523809	6	29849619	intronic	HLA-G,HLA-H	IgE levels;IgE levels	intergenic	22075330
rs2517713	6	29918099	intronic	HLA-G,HLA-J	Nasopharyngeal carcinoma	intergenic	19664746
rs251253	5	172480336	promoter	Y RNA	PR interval	intergenic	20062060
rs245914	7	29218159	intronic	CHN2,CPVL	Obesity-related traits	intergenic	23251661
rs2398162	15	96830550	ncRNA intronic	AK000872, AK307134	Hypertension	intergenic	17554300
rs2395528	10	80100861	ncRNA intronic	AK126491, LOC100132987	Conduct disorder (interaction)	intergenic	18846501

rs2383024	9	17487945	UTR3	CNTLN	Pulmonary function decline	intronic	22424883
rs2382817	2	219151218	UTR5	TMBIM1	Inflammatory bowel disease	intronic	23128233
rs2369304	14	96206070	ncRNA intronic	BX247990	Neutrophil count	intergenic	21507922
rs233100	1	85772009	ncRNA intronic	LOC646626	Multiple sclerosis	intergenic	21833088
rs2312147	2	58222928	intronic	VRK2	Schizophrenia	intergenic	19571808
rs2297644	10	99359739	UTR3	HOGA1	Metabolite levels	intronic	22286219
rs2292303	12	102513531	intronic	NUP37	Height	upstream	19893584
rs2287921	19	49228272	UTR5	RASIP1	Retinal vascular caliber;Bipolar disorder	intronic	21926972, 21060863
rs228437	6	134898456	ncRNA intronic	AJ606331	Melanoma	intergenic	21983787
rs2277862	20	34152782	exonic	FER1L4	Cholesterol, total	intergenic	20686565
rs2275215	6	129861392	ncRNA intronic	BC035400	Body mass index	intergenic	20397748
rs2271404	2	112003867	ncRNA intronic	LOC541471	Atopic dermatitis	intergenic	23042114
rs2259816	12	121435587	exonic	HNF1A	C-reactive protein;Coronary heart disease	intronic	22939635, 19198612
rs2247056	6	31265490	intronic	HLA-B	Triglycerides	intergenic	20686565
rs2240466	7	72856269	ncRNA exonic	BC050599	Triglycerides;Caffeine consumption	intronic	21490707, 19060911
rs2239815	22	29192670	UTR5	XBP1	Esophageal cancer (squamous cell)	intronic	22960999
rs2233152	19	41281016	ncRNA intronic	MIA-RAB4B	Kawasaki disease	upstream	22446962
rs2213169	11	5303063	intronic	HBE1	Mean corpuscular hemoglobin concentration;Hematology traits	intergenic	23263863
rs2173063	15	93131632	promoter	DQ580421, DQ591781	Subcutaneous adipose tissue	intergenic	22589738
rs2165468	10	3516105	ncRNA intronic	BC037918	Bone mineral density	intergenic	17903296
rs211718	1	76106675	ncRNA exonic	CR936677	Metabolite levels;Metabolic traits	intergenic	21886157, 20037589
rs2117032	12	21074122	intronic	SLCO1B3, SLCO1B7	Bilirubin levels	intergenic	19419973
rs2114039	4	55092626	intronic	PDGFRA	Corneal curvature	intergenic	21665993
rs2090409	9	108967088	ncRNA intronic	BC039487	Menarche (age at onset)	intergenic	21102462, 19448620
rs2080401	2	171540823	ncRNA intronic	AK127400	Coronary heart disease	intergenic	21347282
rs2078267	11	64334114	UTR3	SLC22A11	Urate levels	intronic	20884846,

							23263486
rs2075672	7	100240296	intronic	TFR2	Red blood cell traits	downstream	23222517
rs2074488	6	31240431	intronic	HLA-B	Chronic obstructive pulmonary disease-related biomarkers	upstream	23144326
rs2072590	2	177042633	ncRNA exonic	LOC401022	Ovarian cancer	ncRNA intronic	20852632
rs2070488	3	38442490	exonic	XYLB	Electrocardiographic conduction measures	intronic	19389651
rs2048485	18	42090667	ncRNA intronic	BC051727	Schizophrenia	intergenic	22885689
rs2032794	5	86432617	ncRNA intronic	BC034940	Personality dimensions	intergenic	21173776
rs201789	13	50986118	ncRNA intronic	BCMS,DLEU1	Anthropometric traits	intergenic	19260139
rs1967689	1	208039471	UTR3	AK123177	Age-related macular degeneration	intergenic	23326517
rs1944582	18	45815417	intronic	ZBTB7C	Response to taxane treatment (docetaxel)	intergenic	23006423
rs1936800	6	127436064	ncRNA intronic	AK127472	Renal function-related traits (BUN)	intergenic	22797727
rs1930961	22	25875265	ncRNA intronic	CRYBB2P1	Bipolar disorder (mood-incongruent)	intergenic	23092984
rs1918172	2	156888500	ncRNA intronic	BC032407	Attention deficit hyperactivity disorder	intergenic	18821565
rs1883414	6	33086448	UTR3	HLA-DPB2	Nephropathy	ncRNA intronic	21399633
rs1879248	3	180551214	ncRNA intronic	DKFZp434A128	Schizophrenia	intergenic	21926974
rs1859962	17	69108753	ncRNA intronic	BC039327	Prostate cancer	intergenic	21743057, 19767753, 18264097, 17603485
rs1800588	15	58723675	intronic	LIPC	HDL cholesterol	upstream	18193044
rs17824620	12	113100994	intronic	RPH3A	Platelet counts	intergenic	22139419
rs17810546	3	159665050	ncRNA intronic	AK097161	Celiac disease;Behcet's disease	intergenic	20190752, 18311140, 23291587
rs17780304	17	30535856	UTR3	RHOT1	Obesity-related traits	intronic	23251661
rs17780086	17	30343282	intronic	LRRC37B	Height	intergenic	20881960
rs17674580	18	43309911	UTR5	SLC14A1	Bladder cancer	intronic	21750109
rs17651978	3	71020490	UTR3	FOXP1	Attention deficit hyperactivity disorder	intronic	18821565
rs17581368	8	21662445	intronic	DOK2	Entorhinal cortical thickness	intergenic	21116278
rs17511627	13	26724328	intronic	RNF6	Alzheimer's disease	intergenic	22159054
rs17491951	2	2926013	ncRNA	AK095310	White matter integrity	intergenic	23218918

			intronic		(interaction)		
rs1741344	20	4101800	ncRNA exonic	BC027448	Height	intergenic	20881960
rs17410015	1	101551926	ncRNA intronic	AK021551, BC045807, BX538249	Response to antipsychotic treatment	intergenic	20195266
rs17398575	7	106409452	ncRNA intronic	AF086203	Carotid intima media thickness	intergenic	21909108
rs17290922	16	57024317	intronic	NLRC5	Schizophrenia	intergenic	23212062
rs17188434	2	157096776	ncRNA intronic	BC032407	Menarche (age at onset)	intergenic	21102462
rs17129289	1	84232748	ncRNA intronic	BC036594	Obesity-related traits	intergenic	23251661
rs1712517	10	105033015	ncRNA intronic	BC040734	Migraine	intergenic	22683712
rs17084051	4	55087581	intronic	PDGFRA	Corneal astigmatism;Corneal curvature	intergenic	22144915, 23401653
rs17079928	13	24654228	intronic	SPATA13	Orofacial clefts	intergenic	22419666
rs17079773	13	24598384	ncRNA intronic	BC043582	Inattentive symptoms	intergenic	18821565
rs17057678	6	130031493	intronic	ARHGAP18	Antineutrophil cytoplasmic antibody-associated vasculitis;Antineutrophil cytoplasmic antibody-associated vasculitis	upstream	22808956
rs17053082	5	155394230	intronic	SGCD	Type 2 diabetes	intergenic	23300278
rs17031508	4	102560290	intronic	BANK1	Blood pressure	intergenic	22763476
rs1701704	12	56412487	intronic	IKZF4	Type 1 diabetes autoantibodies;Type 1 diabetes;Asthma;Alopecia areata	intergenic	21829393, 21804548, 20596022, 18198356
rs17002342	4	77955205	ncRNA exonic	FW340024	Waist-to-hip circumference ratio (interaction)	intronic	23192594
rs170020	5	151344558	ncRNA intronic	AK001582	Interstitial lung disease;Interstitial lung disease	intergenic	21787189
rs17000918	22	49812502	ncRNA intronic	BC033837	Immune reponse to smallpox (secreted IL-1beta)	intergenic	22610502
rs16996151	4	89978800	intronic	FAM13A	Response to antipsychotic therapy (extrapyramidal side effects)	upstream	19875103
rs16966460	15	38511983	ncRNA	BC037952	Bipolar disorder	intergenic	18711365

			intronic				
rs16965039	16	57047299	intronic	NLRC5	Coronary heart disease	intergenic	21347282
rs16951095	18	7042911	UTR5	LAMA1	Non-small cell lung cancer	intronic	20876614
rs16939046	8	76147954	ncRNA intronic	BC062758	Information processing speed	intergenic	21130836
rs16922670	9	106024220	ncRNA intronic	BC035187	Alzheimer's disease	intergenic	22005930
rs16921914	11	31210771	intronic	DCDC1	Bone mineral density (spine)	intergenic	19801982
rs16906916	10	56848985	intronic	PCDH15	Response to tocilizumab in rheumatoid arthritis	intergenic	22491018
rs16902094	8	128320346	ncRNA intronic	DQ515898, DQ515899, LOC727677	Prostate cancer	intergenic	19767754
rs16869652	6	33851173	promoter	DQ572560	Schizophrenia	intergenic	22885689
rs16861531	2	14398052	ncRNA intronic	BC035112	Response to antidepressant treatment	intergenic	22041458
rs16858228	2	12212445	ncRNA intronic	AK001558	Obesity-related traits	intergenic	23251661
rs16850885	4	75170140	ncRNA intronic	BC016361	Immune reponse to smallpox (secreted IL-1beta)	intergenic	22610502
rs16839962	2	156925286	ncRNA intronic	BC032407	Response to statin therapy	intergenic	20339536
rs16839626	2	204089730	intronic	NBEAL1	Obesity-related traits	intergenic	23251661
rs16830359	1	43596384	ncRNA intronic	AK309744	Cardiac hypertrophy	intergenic	21348951
rs1625895	17	7578115	UTR3	TP53	Sex hormone-binding globulin levels	intronic	22829776
rs1593	4	187195551	UTR3	F11	Activated partial thromboplastin time	intronic	22703881
rs1570854	10	3418124	ncRNA intronic	BC037918	Economic and political preferences (environmentalism)	intergenic	22566634
rs1564892	12	104445742	intronic	GLT8D2	Corneal structure	intergenic	23291589
rs1562430	8	128387852	ncRNA intronic	DQ515898, DQ515899, LOC727677	Breast cancer	intergenic	21263130, 20453838
rs1534422	2	12640741	ncRNA intronic	AK001558	Type 1 diabetes	intergenic	19430480
rs1514178	1	61205469	ncRNA intronic	AK097193	Phospholipid levels (plasma)	intergenic	21829377
rs1495377	12	71577101	intronic	TSPAN8	Type 2 diabetes;Creutzfeldt-Jakob disease (variant)	intergenic	22137330, 17554300

rs1490388	6	126835655	ncRNA intronic	AK127472	Height	intergenic	18391951
rs1490384	6	126851160	ncRNA intronic	AK127472	Height	intergenic	20881960
rs1474476	14	22607502	intronic	AV2S1A1,T-CellReceptorV-alpharegion,TCR-alpha,TCRA,TRA,TRAC,TRD	Obesity-related traits	intergenic	23251661
rs1464108	12	131022010	intronic	RIMBP2	Alzheimer's disease	intergenic	22005930
rs1457451	2	65862378	ncRNA intronic	FLJ16124	Iron status biomarkers	intergenic	19084217
rs1440581	4	89226422	ncRNA intronic	BC027846	Metabolite levels	intergenic	22916037, 22286219
rs1432723	5	155302582	intronic	SGCD	Obesity-related traits	intergenic	23251661
rs1425609	3	162681995	ncRNA intronic	BC073807	Aging (time to death)	intergenic	21782286
rs1420956	18	25167945	ncRNA intronic	AK127888	Obesity-related traits	intergenic	23251661
rs1417437	1	70154441	intronic	LRRC7	Orofacial clefts	intergenic	22419666
rs1371737	7	9189541	ncRNA intronic	RBSG3	Obesity-related traits	intergenic	23251661
rs1361108	6	126767600	ncRNA intronic	AK127472	Menarche (age at onset);Height	intergenic	21998595, 21102462
rs1351394	12	66351826	UTR3	HMGA2	Height	intronic	20881960
rs13438712	7	105542580	intronic	CDHR3	Obesity-related traits	intergenic	23251661
rs13409348	2	79539988	intronic	CTNNA2	Bipolar disorder	intergenic	19416921
rs13398206	2	199169096	ncRNA intronic	AK096499	Prostate cancer (gene x gene interaction)	intergenic	22219177
rs1329189	10	129998372	ncRNA intronic	AK124226	Orofacial clefts	intergenic	22419666
rs13281615	8	128355618	ncRNA intronic	DQ515898, DQ515899, LOC727677	Breast cancer	intergenic	17529967
rs1326986	10	19929513	intronic	AK297683	Ankylosing spondylitis	intergenic	20062062
rs13204965	6	127167072	ncRNA intronic	AK127472	Bone mineral density	intergenic	21533022, 22504420
rs13191343	6	31241109	intronic	HLA-B	Psoriatic arthritis	intergenic	20953186
rs13098911	3	46235201	intronic	CCR3	Celiac disease	intergenic	20190752
rs13015714	2	102971865	intronic	IL18R1	Celiac disease;Atopic dermatitis	intergenic	23042114, 18311140
rs13010713	2	181996045	ncRNA intronic	AK125001	Celiac disease	intergenic	20190752
rs12938916	17	55866287	promoter	7SK	Bipolar disorder	intergenic	21254220
rs12901682	15	78833223	UTR5	PSMA4	Response to tocilizumab	intronic	22491018

					in rheumatoid arthritis		
rs1265879	14	50877983	intronic	CDKL1	Cognitive performance	intergenic	20125193
rs12636651	3	46282391	intronic	CCR3	Monocyte chemoattractant protein-1;Obesity-related traits;Monocyte chemoattractant protein-1	intergenic	23017229, 23251661
rs1262778	13	51066623	ncRNA intronic	BCMS,DLEU1	Bipolar disorder and major depressive disorder (combined)	intergenic	20351715
rs12524865	6	134196674	ncRNA intronic	BC041459	Coronary heart disease	intergenic	22751097
rs1239954	13	51119137	ncRNA intronic	BCMS	Obesity-related traits	intergenic	23251661
rs1239947	13	51106555	ncRNA intronic	BCMS	Height	intergenic	18391951
rs12393627	X	2885723	intronic	ARSE	Height	intergenic	22021425, 21998595
rs12274302	11	90586900	ncRNA intronic	HP11113	HIV-1 control	intergenic	20041166
rs12261589	10	79540835	promoter	DQ570533, DQ575045, DQ576634, DQ580595, DQ582762, DQ584676, DQ586086, DQ593224, DQ593302, DQ594020, DQ594771	Obesity-related traits	intergenic	23251661
rs12206204	6	26116982	intronic	HIST1H2BC	Bilirubin levels	intergenic	19414484
rs12191877	6	31252925	intronic	HLA-B	Psoriasis	intergenic	20953188, 19169254
rs12150660	17	7521915	intronic	SHBG	Testosterone levels;Sex hormone-binding globulin levels	intergenic	22829776, 21998597
rs12145922	1	89146234	ncRNA intronic	AK123834	Liver enzyme levels (gamma-glutamyl transferase)	intergenic	22001757
rs12109285	5	21749348	ncRNA intronic	BC038535	Response to tocilizumab in rheumatoid arthritis	intergenic	22491018
rs12032643	1	249168436	promoter	TRNA Glu	Myopia (pathological)	intergenic	23049088
rs1190739	X	136055295	ncRNA intronic	AK055694	Coronary heart disease	intergenic	21347282
rs11857380	15	58712203	intronic	LIPC	Obesity-related traits	intergenic	23251661

rs11845134	14	22782377	intronic	AV4S1,T-CellReceptorV-alpharegion,TCR-alpha,TCRA,TRA,TRA@,TRAC,TRD,hADV29S1,hADV36S1,hADV38S2	Obesity-related traits	intergenic	23251661
rs11809789	1	82074852	intronic	LPHN2	Bilirubin levels	intergenic	22085899
rs1178979	7	72856430	ncRNA exonic	BC050599	Triglycerides	intronic	20864672
rs11781622	8	24378741	intronic	ADAM7	White matter integrity	intergenic	23218918
rs11711441	3	182821275	intronic	MCCC1	Parkinson's disease	intergenic	21292315
rs11674248	2	24154192	intronic	UBXN2A	Obesity-related traits	intergenic	23251661
rs11654749	17	69125606	ncRNA intronic	BC039327	Pulmonary function (interaction)	intergenic	23284291
rs11618202	13	31113379	intronic	HMGB1	MRI atrophy measures	intergenic	21116278
rs11615916	12	62610860	intronic	FAM19A2	Pulmonary function decline	intergenic	22424883
rs11613352	12	57792580	ncRNA intronic	KIAA1002	Triglycerides;HDL cholesterol	intergenic	20686565
rs11574637	16	31368874	UTR3	ITGAX	Systemic lupus erythematosus	intronic	18204098
rs11564258	12	40792300	intronic	MUC19	Crohn's disease;Inflammatory bowel disease	intergenic	21102463, 23128233
rs1154155	14	23002684	ncRNA intronic	AV8S2A1N1T	Narcolepsy	intergenic	20711174, 19412176
rs11245052	10	128267640	intronic	C10orf90	Metabolite levels (MHPG)	intergenic	23319000
rs11223996	11	134626887	ncRNA intronic	AK125040	Myopia (pathological)	intergenic	23049088
rs11190140	10	101291593	promoter	DQ372722	Ulcerative colitis;Crohn's disease	intergenic	20228799, 18587394
rs111649495	5	118356415	ncRNA intronic	BC036311	Response to amphetamines	intergenic	22952603
rs11163372	1	82247248	intronic	LPHN2	Temperament	intergenic	22832960
rs11139399	9	84372741	ncRNA intronic	BC036431	Hippocampal atrophy	intergenic	22745009
rs11134474	5	155202528	intronic	SGCD	Smoking behavior	intergenic	22006218
rs11078697	17	7469229	UTR3	SENP3	IgM levels	ncRNA intronic	23118916
rs11070098	13	95505262	ncRNA intronic	BC045767	Obesity-related traits	intergenic	23251661
rs1106766	12	57809456	ncRNA	KIAA1002	Urate levels	intergenic	20884846

			intronic				
rs11066453	12	113365621	intronic	OAS1	Gamma glutamyl transpeptidase	intergenic	21909109
rs10993994	10	51549496	intronic	PARG,TIMM23,TIMM23B	Prostate-specific antigen levels;Prostate cancer	upstream	21743057, 21160077, 20676098, 18264097, 18264096, 23269536
rs10961577	9	14562314	intronic	ZDHHC21	Visceral adipose tissue adjusted for BMI	intergenic	22589738
rs10948172	6	44777691	intronic	SUPT3H	Osteoarthritis	intergenic	22763110
rs10947055	6	30093364	ncRNA exonic	DQ580846	Cardiac hypertrophy	intergenic	21348951
rs10945919	6	164186677	ncRNA intronic	AK093114	Response to TNF antagonist treatment	intergenic	18615156
rs10929808	2	12568996	ncRNA intronic	AK001558	Bipolar disorder and schizophrenia	intergenic	20889312
rs10915864	1	225901006	ncRNA intronic	AK124056	Obesity-related traits	intergenic	23251661
rs10897449	11	63592621	ncRNA exonic	AK092098	Electroencephalographic traits in alcoholism	intronic	22554406
rs10888935	1	56060951	intronic	AK127270	Inflammatory biomarkers	intergenic	22228203
rs10883365	10	101287764	ncRNA exonic	DQ372722, chromosome10openreadingframe139	Crohn's disease	intergenic	17554300, 17554261
rs10865035	2	100835734	ncRNA intronic	BC105019	Rheumatoid arthritis	intergenic	20453842
rs10849605	12	1064438	intronic	RAD52	Lung cancer	intergenic	22899653
rs10805321	4	13914373	ncRNA intronic	AK091889	Response to antipsychotic therapy (extrapyramidal side effects)	intergenic	21990027
rs10792665	11	82649768	intronic	C11orf82	Obesity-related traits	intergenic	23251661
rs10776733	1	112107669	intronic	RAP1A	Obesity-related traits	intergenic	23251661
rs10743889	12	10521389	ncRNA intronic	AK096314	Obesity-related traits	intergenic	23251661
rs10740609	10	56790969	intronic	PCDH15	Weight	intergenic	20966902
rs10510138	10	126438019	intronic	FAM53B	Obesity-related traits	intergenic	23251661
rs10509540	10	90023033	intronic	RNLS	Type 1 diabetes	intergenic	19430480
rs10505477	8	128407443	ncRNA intronic	DQ515898, DQ515899, LOC727677	Colorectal cancer	intergenic	17618283
rs10500569	16	72756101	ncRNA	AK021563	Metabolite levels	intergenic	22916037

			intronic				
rs10489087	4	13794416	ncRNA intronic	AK091889	Hemostatic factors and hematological phenotypes	intergenic	17903294
rs10484554	6	31274555	intronic	HLA-B	Psoriasis;AIDS progression	intergenic	20953190, 19115949, 18369459
rs10458787	10	4655565	ncRNA intronic	LOC100216001	Body mass index	intergenic	20397748
rs10423754	19	54604822	intronic	OSCAR	Response to taxane treatment (docetaxel)	upstream	23006423
rs103294	19	54797848	intronic	LILRA6	Prostate cancer	intergenic	23023329
rs10273639	7	142456928	ncRNA intronic	TCRVB	Pancreatitis	upstream	23143602
rs10212363	3	101848561	intronic	ZPLD1	Waist-to-hip circumference ratio (interaction)	intergenic	23192594
rs1018326	2	182007800	ncRNA intronic	AK125001	Ankylosing spondylitis	intergenic	20062062
rs10136766	14	106232585	ncRNA intronic	FLJ00385,IGH@,abParts	IgG levels	intergenic	23225573
rs10129255	14	107176213	ncRNA intronic	abParts	Kawasaki disease	intergenic	21326860
rs10092658	8	130980472	intronic	FAM49B	Protein quantitative trait loci	intergenic	18464913
rs10055544	5	91518721	ncRNA intronic	AK056485	Immune reponse to smallpox (secreted IL-10)	intergenic	22610502
rs10030601	4	150725212	ncRNA intronic	BC031092	Epilepsy (generalized)	intergenic	22949513
rs1000597	7	30937178	intronic	AQP1	Nephrolithiasis	intergenic	22396660

Table S4. Relocated GWAS SNPs to AceView genes

GWAS SNPID	Chr	Coordinate	Relocated region	Relocated AceView gene	Associated disease/trait	Original RefSeq region	PubmedID
rs35079168	9	137280939	exonic	latybu	Intelligence	intronic	22449649
rs2279590	8	27456253	UTR3	CLU	Alzheimer's disease	intronic	19734903
rs11136000	8	27464519	exonic	smawjarby	Alzheimer's disease	intronic	19734903, 19734902
rs9331888	8	27468862	UTR5	CLU	Alzheimer's disease	intronic	19734903
rs10262915	7	94000473	ncRNA intronic	yohuya	Intelligence	intergenic	22449649
rs6465411	7	94003260	ncRNA intronic	yohuya	Intelligence	intergenic	22449649
rs13221576	7	94006994	ncRNA intronic	yohuya	Intelligence	intergenic	22449649
rs4729127	7	94008535	ncRNA intronic	yohuya	Intelligence	intergenic	22449649
rs12627933	22	32027449	ncRNA exonic	lafar	Intelligence	upstream	22449649
rs9621305	22	32049917	intronic	PISD	Intelligence	intergenic	22449649
rs6088735	20	33745676	intronic	EIF6andEDEM2	Hemostatic factors and hematological phenotypes	intergenic	22443383
rs6060278	20	33753262	intronic	EIF6andEDEM2	Hemostatic factors and hematological phenotypes	intergenic	22443383
rs2235617	20	48554977	ncRNA exonic	beeku	Psoriasis	intronic	20953190
rs897200	2	192017771	intronic	STAT4	Behcet's disease	intergenic	23001997
rs9308945	2	34284852	ncRNA intronic	sleelo	Hypertension (young onset)	intergenic	19421330
rs6711736	2	34284984	ncRNA intronic	sleelo	Hypertension (young onset)	intergenic	19421330
rs6729869	2	34285194	ncRNA intronic	sleelo	Hypertension (young onset)	intergenic	19421330
rs10495809	2	34305257	ncRNA intronic	sleelo	Hypertension (young onset)	intergenic	19421330
rs11234027	11	71234107	UTR3	NADSYN1andKRTAP5-7	Vitamin D levels	intergenic	20418485
rs999556	5	150473674	promoter	TNIP1,snyvubo	Myopia (pathological)	intergenic	23049088
rs9995093	4	89220944	intronic	HERC6	Bipolar disorder	intergenic	22925353
rs998584	6	43757896	ncRNA intronic	cherbloy	Adiponectin levels	intergenic	22479202

rs9984974	21	37809377	intronic	teetaw	Myopia (pathological)	intergenic	23049088
rs9982601	21	35599128	intronic	MRPS6andSLC5A3andC21orf82	Myocardial infarction (early onset);Coronary heart disease	intergenic	21378990, 19198609
rs998124	18	42725662	intronic	morsar	Prostate cancer (gene x gene interaction)	intergenic	22219177
rs9978142	21	35652239	intronic	MRPS6andSLC5A3andC21orf82	Pulmonary function	intergenic	21946350
rs993925	1	218860068	ncRNA intronic	hisuyo	Pulmonary function	intergenic	21946350
rs993804	3	25112171	ncRNA intronic	makeme	Bipolar disorder and schizophrenia	intergenic	20889312
rs9923231	16	31107689	promoter	VKORC1andPRSS53	Warfarin maintenance dose	intergenic	20833655, 19300499
rs9922516	16	18024850	ncRNA exonic	yotema	Obesity-related traits	intergenic	23251661
rs9918079	4	15545410	UTR3	CC2D2A	Obesity-related traits	intronic	23251661
rs9913711	17	70098161	intronic	yanima	Liver enzyme levels (gamma-glutamyl transferase)	intergenic	22001757
rs9899891	17	14629746	ncRNA intronic	klawaw	Visceral adipose tissue/subcutaneous adipose tissue ratio	intergenic	22589738
rs9895661	17	59456589	UTR5	BCAS3	Renal function-related traits (sCR);Renal function-related traits (eGRFcrea);Chronic kidney disease	intronic	22797727, 20383146
rs9878522	3	165125159	ncRNA intronic	ferjabu	Response to amphetamines	intergenic	22952603
rs9876781	3	48487338	intronic	CCDC51	Longevity	upstream	20834067
rs987525	8	129946154	intronic	fokey	Orofacial clefts;Cleft lip	intergenic	22863734, 20436469, 19656524, 19270707
rs9874556	3	3356174	ncRNA intronic	stufar	Pancreatic cancer	intergenic	23180869

rs9866141	3	156950579	intronic	skasweyby	Diabetic retinopathy;Diabetic retinopathy	intergenic	21441570
rs9860340	3	87783976	ncRNA intronic	reedubo	Electroencephalographic traits in alcoholism	intergenic	22554406
rs9850225	3	80865764	ncRNA intronic	smysweyby,suswerby	Erectile dysfunction	intergenic	22704111
rs9844666	3	135974216	UTR5	PCCB	Height	intronic	20881960
rs984440	8	139077728	ncRNA intronic	barzyby	Obesity-related traits	intergenic	23251661
rs9836484	3	78044689	ncRNA intronic	reeswerby	Brain imaging in schizophrenia (interaction)	intergenic	19023125
rs9835812	3	3375784	ncRNA intronic	stufar	Immune response to smallpox vaccine (IL-6)	intergenic	22542470
rs9824150	3	162834301	ncRNA intronic	zyskawby	Capecitabine sensitivity	intergenic	22864933
rs9822268	3	49719729	UTR5	APEH	Ulcerative colitis	intronic	21297633
rs9811423	3	112822322	intronic	rorsweyby	Alzheimer's disease	intergenic	22005930
rs9810890	3	128652553	intronic	KIAA1257	Dental caries	intergenic	23064961
rs9807989	2	102971200	intronic	IL1RL1andIL18R1	Asthma	intergenic	22561531
rs9807334	18	48524161	intronic	ELAC1andSMAD4	Immune reponse to smallpox (secreted IFN-alpha)	intergenic	22610502
rs9805786	13	24658356	intronic	SPATA13andC1QTNF9	Depression and alcohol dependence	intergenic	22064162
rs9803659	1	167156500	intronic	rawbar	Liver enzyme levels	intergenic	18940312
rs974819	11	103660567	ncRNA intronic	keruro	Coronary heart disease	intergenic	21378988
rs972275	6	127391844	ncRNA intronic	klubloyby,sowame	Iron status biomarkers	intergenic	19084217
rs969088	5	26389262	ncRNA intronic	berplawby	Response to platinum-based chemotherapy in non-small-cell lung cancer	intergenic	22872573
rs9686661	5	55861786	intronic	snogee	Triglycerides	intergenic	20686565
rs9657451	8	138905296	ncRNA intronic	nereya	Cognitive performance	intergenic	20125193

rs9653442	2	100825367	intronic	LOC150577	Type 1 diabetes	intergenic	17554260
rs9652490	15	77963887	intronic	LINGO1	Essential tremor	intergenic	19182806
rs964184	11	116648917	UTR3	ZNF259	Vitamin E levels;Triglycerides;Response to Vitamin E supplementation;Phospholipid levels (plasma);Metabolite levels;Metabolic syndrome;Lipoprotein-associated phospholipase A2 activity and mass;LDL cholesterol;Hypertriglyceridemia;HDL cholesterol;Coronary	downstream	22916037, 22437554, 22399527, 22359512, 22003152, 21729881, 21378990, 20864672, 20686565, 20657596, 19060906
rs9636231	2	139671955	ncRNA intronic	puklee	Alcohol dependence	intergenic	21956439
rs9635542	16	5001380	intronic	PPL	Lung cancer-asbestos exposure interaction	intergenic	22637743
rs961831	9	22362104	UTR3	tosiro	Personality dimensions	intergenic	20691247
rs960902	2	37731665	ncRNA intronic	jerdaw	Response to TNF-alpha inhibitors in rheumatoid arthritis	intergenic	22569225
rs9608102	22	23644794	exonic	BCR	Immune response to smallpox vaccine (IL-6)	intronic	22542470
rs9604529	13	114622597	exonic	FLJ44054	Response to tocilizumab in rheumatoid arthritis	intergenic	22491018
rs960246	2	223937559	intronic	KCNE4	C-reactive protein and white blood cell count	intergenic	22788528

rs9596270	13	50842440	intronic	DLEU1	Multiple sclerosis	intergenic	22190364
rs9594759	13	43032593	ncRNA intronic	feeseyby	Bone mineral density (spine)	intergenic	19079262, 18445777
rs958546	13	46833717	intronic	LRRC63	Atrial fibrillation	intergenic	17903304
rs956237	4	109046960	ncRNA exonic	smarsnarby	Systemic lupus erythematosus	intronic	22291604
rs956225	8	122909687	ncRNA intronic	leyterbo	Alzheimer's disease	intergenic	22159054
rs954820	10	133957761	UTR5;UTR3	JAKMIP3;JAKMIP3	Chronic obstructive pulmonary disease-related biomarkers	intronic	23144326
rs9548119	13	38531581	ncRNA intronic	marzo	Self-rated health	intergenic	20707712
rs9533799	13	44808174	ncRNA intronic	swafuby	Amyotrophic lateral sclerosis	intergenic	22959728
rs9507041	13	23744379	ncRNA intronic	numimi,tunor	Cannabis dependence	intergenic	21668797
rs9500256	6	58308955	ncRNA intronic	forkleyby	Eosinophilic esophagitis (pediatric)	intergenic	20208534
rs9497975	6	148547802	ncRNA intronic	muvybo	HIV-1 control	intergenic	20041166
rs9485372	6	149608874	intronic	TAB2	Breast cancer	intergenic	22383897
rs9480865	6	108916573	ncRNA exonic	spawvabo	Normalized brain volume	intronic	19010793
rs9468925	6	31258837	intronic	HLA-BandHLA-C	Vitiligo	intergenic	20526339
rs9461688	6	31271716	ncRNA intronic	rosiya	Protein quantitative trait loci	intergenic	18464913
rs9442235	1	16393357	exonic	teymorbu	Cognitive performance	intronic	19734545
rs943072	6	43795968	ncRNA intronic	cherbloy	Ulcerative colitis	intergenic	21297633
rs941853	10	116199175	UTR5	tukimi	Obesity-related traits	intronic	23251661
rs9402515	6	133860345	ncRNA intronic	pajer	Protein quantitative trait loci	intergenic	18464913
rs9399137	6	135419018	intronic	HBS1LandALDH8A1	Platelet counts;Hematology traits;HbA2 levels;F-cell distribution;Mea	intergenic	23043469, 22139419, 19853236, 17767159, 23263863

					n corpuscular volume;Mean corpuscular hemoglobin		
rs9398652	6	122146034	ncRNA intronic	skoytoybo	Resting heart rate	intergenic	20639392
rs9393366	6	23077311	intronic	shawpoyby	Tourette syndrome	intergenic	22889924
rs9388489	6	126698719	intronic	CENPW	Type 1 diabetes	intergenic	19430480
rs9387478	6	117786180	intronic	GOPCandROS1	Lung cancer	intergenic	23143601
rs9377063	6	147934947	intronic	SAMD5	Obesity-related traits	intergenic	23251661
rs9375969	6	133867802	ncRNA intronic	pajer	Visceral fat	intergenic	22589738
rs9373124	6	135423209	intronic	HBS1LandALDH8A1	White blood cell types;Other erythrocyte phenotypes	intergenic	21738478, 19862010
rs9368677	6	31272321	ncRNA intronic	rosiya	Atopic dermatitis	intergenic	23042114
rs9357155	6	32809848	UTR3	PSMB8	Nephropathy	intronic	21399633
rs934299	2	137338940	ncRNA intronic	noygybo	Cognitive test performance	intergenic	17903297
rs9321637	6	138266685	ncRNA intronic	weejer	Cardiac Troponin-T levels	intergenic	23247143
rs9320841	6	122114451	ncRNA intronic	skoytoybo	Resting heart rate	intergenic	23183192
rs9317284	13	63634350	intronic	wersta	Bone mineral density	intergenic	17903296
rs9316500	13	51094114	intronic	samimi	Pulmonary function decline	intergenic	22424883
rs9313772	5	157804457	ncRNA intronic	slogey	Blood pressure	intergenic	21909110
rs9312648	4	55241581	ncRNA intronic	moysweeby	Response to amphetamines	intergenic	22952603
rs930811	9	2696555	intronic	norrorby	Obesity-related traits	intergenic	23251661
rs9305354	21	29475196	ncRNA exonic	memera	Urinary albumin excretion	intergenic	17903292
rs930421	2	42981239	UTR3	MTA3	Attention deficit hyperactivity disorder	intergenic	18821565
rs9302001	13	95463392	ncRNA intronic	swonor	Panic disorder	intergenic	19165232
rs9296015	6	32218989	intronic	RNF5	Systemic sclerosis;Rheumatoid arthritis	intergenic	21779181, 21505073

rs9295536	6	22131929	exonic	klojar	Neuroblastoma	ncRNA intronic	22941191
rs9287638	2	239694631	ncRNA intronic	hayoyo	Male-pattern baldness	intergenic	22693459
rs9286879	1	172862234	ncRNA intronic	ferplo	Crohn's disease	intergenic	18587394, 23128233
rs9273349	6	32625869	UTR3	HLA-DQB1	Asthma	intergenic	20860503
rs9272346	6	32604372	intronic	HLA-DQA1	Type 1 diabetes;Asthma	upstream	18978792, 17554300, 23181788
rs9272219	6	32602269	intronic	HLA-DQA1	Schizophrenia;Rheumatoid arthritis	intergenic	21653640, 19571809
rs9272105	6	32599999	intronic	HLA-DQA1	Response to interferon beta therapy;Hepatocellular carcinoma	intergenic	22807686, 21502966
rs9271858	6	32595223	promoter	HLA-DQA1	Leishmaniasis (visceral)	intergenic	23291585
rs9267911	6	32205110	intronic	RNF5	Crohn's disease	intergenic	23266558
rs9267663	6	31867253	UTR3	ZBTB12	Economic and political preferences (environmentalism)	downstream	22566634
rs9266406	6	31336418	promoter	DHFRP2	Behcet's disease	intergenic	23001997
rs9264942	6	31274380	ncRNA intronic	rosiya	HIV-1 control;Crohn's disease	intergenic	21051598, 20041166, 23128233
rs9262632	6	31024808	UTR3	HCG22	HIV-1 control	ncRNA intronic	21051598
rs9260489	6	29920332	ncRNA exonic	susnoybu	Multiple sclerosis	intergenic	22190364
rs9257809	6	29356331	intronic	OR11A1andOR5V1	Barrett's esophagus	intergenic	22961001
rs925255	2	28614794	UTR3	glarjawbu	Inflammatory bowel disease	upstream	23128233
rs923175	11	12072503	intronic	suriro	Response to taxane treatment (docetaxel)	intergenic	23006423
rs922948	3	69442637	intronic	FRMD4B	Hip geometry	intergenic	17903296
rs921720	8	126534671	ncRNA intronic	kavoyby	Obesity-related traits;Inflammatory bowel disease	intergenic	23251661, 23128233
rs911119	20	23612737	intronic	CST3	Chronic kidney disease	intergenic	20383146

rs900400	3	156798775	promoter	glorswoybu	Birth weight	downstream	20372150, 23202124
rs895636	2	45188353	ncRNA exonic	pujerbo	Metabolite levels	intergenic	21909109
rs894177	3	142894411	promoter	PBX2P1	Non-alcoholic fatty liver disease histology (other)	intergenic	20708005
rs890835	5	175956271	exonic	RNF44	Menopause (age at onset)	intronic	22267201
rs887864	16	11158885	UTR3	feywarbu	Allergic rhinitis	intronic	22036096
rs886424	6	30782002	intronic	yuko	Bipolar disorder and schizophrenia	intergenic	22688191
rs883924	9	93181531	ncRNA intronic	siyamu,zoyley	Hepatitis C induced liver fibrosis	intergenic	22841784
rs881858	6	43806609	ncRNA intronic	cherbloy	Chronic kidney disease	intergenic	20383146
rs881375	9	123652898	UTR3	dorsher	Rheumatoid arthritis	intergenic	19503088
rs879882	6	31139452	intronic	POU5F1	Height	intergenic	20881960
rs875622	19	16467759	UTR3	wawsoy	White matter integrity	intronic	23218918
rs873549	1	222271767	intronic	pawber	Keloid	intergenic	20711176
rs870288	16	5585852	intronic	LOC100287538	Subcutaneous adipose tissue;Phospholipid levels (plasma)	intergenic	22589738, 22359512
rs861318	1	159002222	UTR5	IFI16	Obesity-related traits	intronic	23251661
rs857228	14	98670158	ncRNA intronic	vydyby	Sexual dysfunction (SSRI/SNRI-related)	intergenic	22445761
rs854572	7	94954696	intronic	PON3andPON1	Paraoxonase activity	upstream	22982463
rs853356	6	14173428	intronic	CD83	Height	intergenic	21998595
rs848353	7	108548660	ncRNA intronic	nahuya	Smoking behavior	intergenic	22006218
rs840016	1	167408670	UTR5	CD247	Rheumatoid arthritis	intronic	20453842
rs838880	12	125261593	UTR3	UBCandSCARB1	HDL cholesterol	downstream	20686565
rs836589	2	173493078	UTR5	guferbu	Erectile dysfunction	intergenic	22704111
rs836132	11	34555191	ncRNA	sheyglaw	β2-	intergenic	23279374

			intronic		Glycoprotein I (β2-GPI) plasma levels		
rs835367	1	59762468	UTR5	FGGY	Obesity-related traits	upstream	23251661
rs830884	5	52020396	ncRNA intronic	ruroyu	Response to platinum-based agents	intergenic	22020760
rs824931	2	222801699	ncRNA intronic	kawgabo	Body mass index	intergenic	19851299
rs8176704	9	136135552	exonic	foytoyby	Coagulation factor levels	intronic	23267103
rs816488	3	39553788	UTR5	RPSAandSNORA62andMOBP	Cognitive performance	intronic	19734545
rs815815	2	47399064	UTR5	CALM2andC2orf61	Dialysis-related mortality	intronic	21546767
rs8127571	21	47165429	intronic	PCBP3	Immune reponse to smallpox (secreted IFN-alpha)	intergenic	22610502
rs8112449	19	10520064	intronic	CDC37	Multiple sclerosis	intergenic	21833088
rs8109288	19	16185559	UTR5	TPM4	Platelet counts;Mean platelet volume	intronic	22423221, 22139419
rs8102683	19	41363765	intronic	MIA	Smoking behavior	intergenic	23049750
rs8102476	19	38735613	intronic	SPINT2andC19orf33	Prostate cancer	intergenic	19767754
rs8097348	18	1595021	intronic	royso	Exercise (leisure time)	intergenic	19727025
rs8077059	17	55823552	ncRNA intronic	neysley	Sex hormone-binding globulin levels	intergenic	22675492
rs8076739	17	27714587	intronic	TAOK1,shylee	Mean platelet volume	intergenic	22139419
rs8070473	17	33843512	intronic	SLFN12L	Depression (quantitative trait)	intergenic	20800221
rs8068318	17	59483766	UTR3	TBX2	Creatinine levels	intronic	20383145
rs806794	6	26200677	UTR3	HIST1H2BF	Height	downstream	20881960
rs806321	13	50841323	intronic	DLEU1	Multiple sclerosis	intergenic	21833088
rs8062326	16	19156016	intronic	ITPRIPL2andSYT17	Alcohol dependence	intergenic	21876473
rs805303	6	31616366	exonic	BAT3	Systolic blood pressure;Hypert	intronic	21909115

					ension;Diastolic blood pressure		
rs8052560	16	88777242	UTR3	CTU2	Height	intronic	20881960
rs8049607	16	11691753	intronic	LITAF	QT interval	intergenic	23166209, 19305409, 19305408
rs8033165	15	29006093	intronic	WHAMML2	Black vs. red hair color;Black vs. blond hair color	intergenic	18483556
rs8023580	15	96708291	intronic	lorswo	Sex hormone-binding globulin levels	intergenic	22829776
rs8017161	14	103563195	ncRNA intronic	forva	Primary biliary cirrhosis	intergenic	21399635
rs8016947	14	35832666	intronic	berswoy	Psoriasis	intergenic	20953190
rs8016620	14	97393685	intronic	VRK1	Obesity-related traits	intergenic	23251661
rs8014204	14	75322794	UTR3	YLPM1	Caffeine consumption	intronic	21490707
rs8013477	14	49534425	ncRNA intronic	flawpu	Visceral adipose tissue/subcutaneous adipose tissue ratio	intergenic	22589738
rs8004664	14	90034972	UTR5	fartabo	Fasting glucose-related traits (interaction with BMI)	intronic	22581228
rs7992643	13	100555038	ncRNA intronic	rordeeby	Attention deficit hyperactivity disorder	intergenic	18821565
rs798544	7	2763102	UTR5	GNA12,fuchuby	Height	intergenic	18391951
rs798489	7	2801803	splicing	GNA12(GNA12.iAug10:exon3:c.115+1G>A)	Height	intronic	20881960
rs7981942	13	80623346	UTR3	kleeferby	Fasting insulin-related traits (interaction with BMI)	intergenic	22581228
rs7971536	12	102373788	intronic	DRAM1	Height	intergenic	20881960
rs7965584	12	90305779	intronic	monare	Magnesium levels	intergenic	20700443
rs7965445	12	131862903	ncRNA intronic	ruhere	Mortality among heart failure patients	intergenic	20400778
rs7961581	12	71663102	intronic	TSPAN8	Type 2 diabetes	intergenic	18372903
rs7953249	12	121403724	intronic	NCRNA00262	N-glycan levels;Chronic	intergenic	23144326, 21203500

					obstructive pulmonary disease-related biomarkers		
rs7950811	11	92651002	ncRNA intronic	LOC642791	Conduct disorder (symptom count);Conduct disorder (case status)	intergenic	20585324
rs7950726	11	5225447	promoter	smufee	HbA2 levels	intergenic	23043469
rs7929679	11	34805849	ncRNA intronic	zyflorbu	Chronic obstructive pulmonary disease-related biomarkers	intergenic	23144326
rs7923609	10	65133822	ncRNA exonic	voysha	Liver enzyme levels (alkaline phosphatase)	intronic	22001757
rs79156074	9	16000235	intronic	C9orf93	Metabolite levels (5-HIAA/ MHPG Ratio)	intergenic	23319000
rs7913069	10	105714399	ncRNA intronic	swumorby	Uterine fibroids	intergenic	21460842
rs7900909	10	66448472	ncRNA intronic	nohero	Response to statin therapy	intergenic	20339536
rs7897194	10	20792334	ncRNA intronic	kahero	Bipolar disorder	intergenic	22925353
rs789560	12	70331827	intronic	C12orf28	Attention deficit hyperactivity disorder and conduct disorder	intergenic	18951430
rs7864204	9	71229179	ncRNA intronic	narlar	Body mass index (interaction)	intergenic	23192594
rs7861820	9	108936674	ncRNA intronic	glerspar	Menarche and menopause (age at onset)	intergenic	19448621
rs78546022	6	150380884	promoter	plujee	Metabolite levels (HVA/5-HIAA ratio)	intergenic	23319000
rs785422	15	30173885	intronic	TJP1	Corneal structure	intergenic	23291589
rs7846606	8	77507613	ncRNA intronic	spykey	Orofacial clefts	intergenic	22863734
rs7844723	8	122908503	ncRNA intronic	leyterbo	Hemostatic factors and hematological	intergenic	17903294

					phenotypes		
rs7838490	8	89585048	intronic	korskawbu	Body mass index and cholesterol (psychopharmacological treatment)	intergenic	22417934
rs7827290	8	142300315	intronic	SLC45A4	Bipolar disorder	intergenic	21926972
rs7821565	8	52216762	ncRNA intronic	sloykor	Tuberculosis	intergenic	20694014
rs7815944	8	129427518	intronic	fykey	Atopic dermatitis	intergenic	23042114
rs7814403	8	36459454	ncRNA intronic	rorbla	Obesity-related traits	intergenic	23251661
rs7801190	7	100458093	UTR5	SLC12A9	Coronary heart disease	intronic	21347282
rs7792596	7	94009634	ncRNA exonic	yohuya	Intelligence	intergenic	22449649
rs7781370	7	96133531	intronic	SHFM1	Bone mineral density (hip)	upstream	19801982
rs7780752	7	93241640	intronic	GNGT1	Birth weight	intergenic	23202124
rs7776054	6	135418916	intronic	HBS1LandALDH8A1	Mean corpuscular hemoglobin	intergenic	19862010
rs7775698	6	135418635	intronic	HBS1LandALDH8A1	Red blood cell traits;Hematology traits;Mean corpuscular volume;Mean corpuscular hemoglobin	intergenic	20927387, 19853236, 23263863
rs7772697	6	149435111	intronic	jajo	Diabetic retinopathy;Diabetic retinopathy	intergenic	21441570
rs7772131	6	30795171	intronic	yuko	Response to angiotensin II receptor blocker therapy	intergenic	22566498
rs776488	2	165890733	ncRNA intronic	zakla	Common traits (Other)	intergenic	20585627
rs7758616	6	14200147	ncRNA intronic	jorglorbu	Obesity-related traits	intergenic	23251661
rs7758128	6	32345283	intronic	RNF5	Vitiligo	intergenic	21326295
rs7756521	6	30848253	intronic	DDR1	HIV-1 control	intergenic	20041166
rs77443149	7	97924554	UTR3	BAIAP2L1	Obesity-related traits	intronic	23251661
rs7743761	6	31336100	promoter	DHFRP2	Ankylosing spondylitis	intergenic	20062062

rs7735699	5	79099464	ncRNA intronic	swawjor	Bipolar disorder and schizophrenia	intergenic	20889312
rs7717572	5	66833257	ncRNA intronic	leyger	Prostate cancer (gene x gene interaction)	intergenic	22219177
rs7714584	5	150270420	intronic	slowuby	Crohn's disease	intergenic	21102463
rs7705924	5	101946798	ncRNA intronic	beysloby	Crohn's disease	intergenic	22412388
rs7703051	5	74625487	ncRNA intronic	horoyu	LDL cholesterol	intergenic	18802019
rs7702057	5	115727838	intronic	COMMD10	Amyotrophic lateral sclerosis	intergenic	19451621
rs769449	19	45410002	exonic	APOE	C-reactive protein	intronic	18439548
rs7694379	4	88186509	ncRNA intronic	torflor	Platelet counts	intergenic	22139419
rs76884941	3	111201785	ncRNA intronic	hakume,weyswoybu	Chronic obstructive pulmonary disease	intergenic	21685187
rs7681423	4	155542248	intronic	FGG	Fibrinogen	intergenic	21757653
rs768048	18	50285398	UTR3	DCC	Alcoholism (alcohol use disorder factor score)	intronic	21529783
rs7679673	4	106061534	ncRNA intronic	tunayu	Prostate cancer	intergenic	19767753
rs767210	13	111470038	intronic	shoyserby	Cognitive performance	intergenic	19734545
rs7671266	4	10056376	ncRNA intronic	dygo	Cardiovascular disease risk factors	intergenic	21943158
rs7667	1	19718824	ncRNA exonic	kawkorbu,zaplu	Crohn's disease and psoriasis	intronic	22482804
rs7665939	4	190123318	ncRNA intronic	woygly	Amyotrophic lateral sclerosis	intergenic	22959728
rs7665590	4	99796784	promoter	hisare	Primary biliary cirrhosis	intergenic	23000144
rs7657746	4	123161619	UTR3	KIAA1109	Inflammatory bowel disease	intronic	23128233
rs7656416	4	1254535	intronic	C4orf42	Type 2 diabetes	intergenic	22456796
rs7646881	3	158453279	intronic	MFSD1	Tetralogy of Fallot	intergenic	23297363
rs7640543	3	30462403	intronic	flawfybo	Migraine	intergenic	22683712
rs7638110	3	138903985	intronic	MRPS22	Obesity and blood pressure	intergenic	22013104
rs7634533	3	30372053	intronic	flawfybo	Dental caries	intergenic	23259602

rs7634528	3	177766464	promoter	faswerby	Myopia (pathological)	intergenic	23049088
rs7616215	3	46205686	intronic	CCR3	Behcet's disease	intergenic	23291587
rs7612209	3	177596989	ncRNA intronic	kemura	Cognitive performance	intergenic	19734545
rs7605378	2	200676926	UTR3	LOC348751	Osteoporosis	ncRNA intronic	21573128
rs7600502	2	22297003	ncRNA intronic	spyjobu	C-reactive protein and white blood cell count	intergenic	22788528
rs7600259	2	35093293	ncRNA intronic	kimiyo	Response to amphetamines	intergenic	22952603
rs7595103	2	78075244	intronic	veydor	Attention deficit hyperactivity disorder and conduct disorder	intergenic	18951430
rs7590720	2	216898658	intronic	PECRandMREG	Alcohol dependence	intergenic	19581569
rs7588567	2	134363032	intronic	NCKAP5	Glaucoma (primary open-angle)	intergenic	22419738
rs7587205	2	39843197	ncRNA intronic	homomo	Response to angiotensin II receptor blocker therapy (opposite direction w/ diuretic therapy)	intergenic	22566498
rs7584262	2	42250549	ncRNA intronic	remuyo	Bone mineral density	intergenic	22504420
rs75825892	16	4257313	UTR5	SRL	Obesity-related traits	intronic	23251661
rs7578035	2	99382892	ncRNA intronic	jawjybu	Bipolar disorder	intergenic	21926972
rs7571971	2	88895351	exonic	EIF2AK3	Progressive supranuclear palsy	intronic	21685912
rs7570469	2	79709354	intronic	CTNNA2	Response to antipsychotic treatment	intergenic	20195266
rs7566934	2	176654738	ncRNA intronic	slunorbo	Information processing speed	intergenic	21130836
rs7561273	2	24247514	intronic	MFSD2BandFKBP1B	Quantitative traits	downstream	19197348
rs7555668	1	112856570	ncRNA intronic	smerley	Cognitive performance	intergenic	20125193
rs7552393	1	84254551	ncRNA	plerber	Select biomarker	intergenic	17903293

			intronic		traits		
rs7550918	1	247675559	ncRNA intronic	nurobo	Platelet counts	intergenic	22139419
rs7542900	1	95070041	ncRNA intronic	yoseyo	Type 2 diabetes	intergenic	22238593
rs753955	13	24293859	intronic	kerzo	Lung cancer	intergenic	21725308
rs7539409	1	84254735	ncRNA intronic	plerber	Alzheimer's disease	intergenic	20061627
rs7529925	1	199007208	ncRNA intronic	gobor	Red blood cell traits	intergenic	23222517
rs7526034	1	63586973	ncRNA intronic	sorbor	Brain imaging	intergenic	20100581
rs7525133	1	156341494	UTR3	RHBG	Visceral adipose tissue adjusted for BMI	intronic	22589738
rs7524694	1	104019631	intronic	teyfawbu	Obesity-related traits	intergenic	23251661
rs7517810	1	172853460	ncRNA intronic	ferplo	Crohn's disease	intergenic	21102463
rs746630	14	83255205	exonic	fava	Obesity-related traits	intergenic	23251661
rs7445832	5	62586301	intronic	garger	Alcohol and nictotine co-dependence	intergenic	23216389
rs744016	22	27042106	intronic	risa	RR interval (heart rate)	intergenic	20031603
rs743793	22	33561746	ncRNA intronic	bary	Visceral adipose tissue/subcutaneous adipose tissue ratio	intergenic	22589738
rs743777	22	37551607	intronic	C1QTNF6andIL2RB	Type 1 diabetes autoantibodies; Rheumatoid arthritis	intergenic	21829393, 21653640, 17554300
rs740363	10	118575606	intronic	HSPA12A	Heart failure	intergenic	17903304
rs739310	22	27051299	intronic	MIAT	Obesity-related traits	intergenic	23251661
rs7387468	8	94358098	ncRNA intronic	LOC642924	Temperament	downstream	22832960
rs7360412	20	2800801	UTR3	C20orf141	White matter integrity	intergenic	23218918
rs735860	6	53123118	intronic	ELOVL5andRPS16P5	Glaucoma	intergenic	20363506
rs735854	22	36679058	UTR5	MYH9	Optic disc size (rim)	intronic	20395239
rs7356884	6	22753047	ncRNA intronic	savabo	Obesity-related traits	intergenic	23251661
rs735665	11	123361397	intronic	GRAMD1B	Follicular	intergenic	22700719,

					lymphoma;Chronic lymphocytic leukemia		20639881, 18758461
rs735396	12	121438844	exonic	C12orf43	N-glycan levels	intronic	21203500
rs734999	1	2513216	ncRNA intronic	geyvoybo	Ulcerative colitis	intergenic	21297633
rs733175	4	10050141	ncRNA intronic	dygo	Alzheimer's disease	intergenic	22005930
rs73317122	17	25320889	intronic	gurer	Response to amphetamines	intergenic	22952603
rs732505	19	5582535	intronic	SAFB2andPLAC2	vWF and FVIII levels	intergenic	21810271
rs7324557	13	24296862	intronic	kerzo	Visceral adipose tissue adjusted for BMI	intergenic	22589738
rs730570	14	101142890	ncRNA intronic	jeydoy	Type 2 diabetes	intergenic	21573907
rs730566	3	48487048	intronic	CCDC51	Prion diseases	intergenic	22210626
rs7305618	12	121402932	intronic	NCRNA00262	Type 2 diabetes;C-reactive protein	intergenic	21647738, 21573907
rs7302017	12	63004583	ncRNA intronic	kirori	Waist circumference	intergenic	20966902
rs72991	11	121243716	ncRNA intronic	flofer	Response to tocilizumab in rheumatoid arthritis	intergenic	22491018
rs729761	6	43804571	ncRNA intronic	cherbloy,husiya	Urate levels	intergenic	23263486
rs7296418	12	123457619	UTR5	ABCB9	Platelet counts	intergenic	21507922
rs729397	10	2532019	intronic	meemy	Response to statin therapy	intergenic	20339536
rs729302	7	128568960	promoter	kawflawbu	Systemic lupus erythematosus	intergenic	23053960, 23273568
rs728616	10	81847914	ncRNA exonic	blersho	Chronic obstructive pulmonary disease-related biomarkers	intronic	23144326
rs72829446	17	7552123	intronic	ATP1B2	Androgen levels	intergenic	22936694
rs72823592	17	46123004	intronic	COPZ2	Epilepsy (generalized)	intergenic	22949513
rs72820627	10	96852690	intronic	CYP2C8	Response to amphetamines	intergenic	22952603
rs727979	6	149593920	intronic	TAB2	Hemostatic factors and hematological phenotypes	intergenic	17903294

rs72669744	1	56116505	intronic	yusimo	Lipid metabolism phenotypes	intergenic	22286219
rs7260598	19	24222786	intronic	ZNF254	Response to taxane treatment (placlitaxel)	intergenic	23006423
rs7255066	19	45146103	ncRNA intronic	basee	Multiple sclerosis	upstream	21833088
rs7253363	19	11682495	intronic	ZNF627	Multiple sclerosis (severity)	intergenic	19010793
rs724950	4	128342120	intronic	wableyby	Obesity-related traits	intergenic	23251661
rs7243299	18	7755771	UTR5	PTPRM	Dialysis-related mortality	intronic	21546767
rs724016	3	141105570	UTR5	ZBTB38	Height	intronic	20881960, 18391950, 18193045
rs7235440	18	22106776	ncRNA intronic	remiri	Obesity-related traits	intergenic	23251661
rs7217319	17	38924	ncRNA intronic	glasoy	AIDS progression	intergenic	21502085
rs7204371	16	67693797	exonic	ACD	Obesity-related traits	intronic	23251661
rs7203193	16	11641180	intronic	LITAF	Coronary heart disease	downstrea m	21347282
rs719593	2	42004997	ncRNA intronic	lawfa	Conduct disorder (interaction)	intergenic	18846501
rs7191888	16	73581058	ncRNA intronic	sisuri	Multiple sclerosis (severity)	intergenic	19010793
rs7191700	16	11406803	intronic	C16orf75	Multiple sclerosis	intergenic	22190364
rs7188445	16	79734987	ncRNA intronic	narar	Urate levels	intergenic	23263486
rs7186852	16	30635659	promoter	ZNF689	Systemic lupus erythematosus	intergenic	19838193
rs718545	14	30489623	intronic	PRKD1	Obesity-related traits	intergenic	23251661
rs718314	12	26453283	intronic	sheestaw	Waist-hip ratio;Renal cell carcinoma	intergenic	22010048, 20935629
rs7181753	15	96844727	intronic	lorswo	Electroencephal ographic traits in alcoholism	intergenic	22554406
rs7175404	15	94036688	ncRNA intronic	nosoru	Attention deficit hyperactivity	intergenic	18839057

					disorder		
rs7159888	14	65758642	ncRNA intronic	mohoru	N-glycan levels	intergenic	21203500
rs7156960	14	76703351	intronic	C14orf118	Acute lymphoblastic leukemia (childhood)	intergenic	22076464
rs7152623	14	98588321	ncRNA intronic	vydyby	Aortic stiffness	intergenic	22068335
rs713875	22	30592487	intronic	slardoy	Crohn's disease	intergenic	21102463
rs7137869	12	119989646	intronic	CCDC60andTMEM233	Aging traits	intergenic	17903295
rs7136259	12	90081188	intronic	ATP2B1	Coronary heart disease	intergenic	22751097
rs7134599	12	68500075	ncRNA intronic	LOC341333	Ulcerative colitis;Inflammatory bowel disease	intergenic	21297633, 23128233
rs71327718	3	125592857	intronic	nayura,toybeybu	Metabolite levels (HVA/5-HIAA ratio)	intergenic	23319000
rs7129220	11	10350538	intronic	AMPD3	Systolic blood pressure;Diastolic blood pressure;Blood pressure	intergenic	21909115, 21909110
rs712022	11	22843155	UTR3	SVIP	Dialysis-related mortality	downstream	21546767
rs7117858	11	15694462	intronic	sherskerby	Bone mineral density (hip)	intergenic	19801982
rs711355	15	30193468	intronic	TJP1	Response to antipsychotic treatment	intergenic	23241943
rs7112365	11	125277494	UTR3	kekora	Obesity-related traits	intronic	23251661
rs7108738	11	15710084	intronic	sherskerby	Bone mineral density	intergenic	22504420
rs7107217	11	129473690	ncRNA intronic	serero	Type 2 diabetes;Breast cancer	intergenic	22383897, 22238593
rs7105934	11	69239741	promoter	sayare	Renal cell carcinoma	intergenic	21131975
rs7105881	11	112456221	intronic	korna	Response to antipsychotic treatment	intergenic	20195266
rs7105056	11	112064431	exonic	SDHD	Immune reponse to smallpox (secreted IL-	intronic	22610502

					12p40)		
rs7102705	11	69143284	intronic	MYEOV	Breast size	intergenic	22747683
rs7094131	10	22847439	UTR5	hukomi	Obesity-related traits	intronic	23251661
rs7090512	10	6110829	exonic	kakloybu	Multiple sclerosis	intergenic	21833088
rs708547	4	57821309	intronic	slorspeeby	Bleomycin sensitivity	intergenic	21106707
rs7085142	10	122939374	ncRNA intronic	permu	Visceral fat	intergenic	22589738
rs7081678	10	31990623	ncRNA intronic	voloy	Waist-hip ratio	intergenic	20935629
rs7077335	10	6315794	ncRNA intronic	munaro	Myopia (pathological)	intergenic	23049088
rs7064929	X	64367019	ncRNA intronic	zarbaw	Erectile dysfunction and prostate cancer treatment	intergenic	20932654
rs7059886	X	3410669	intronic	geyby	Antineutrophil cytoplasmic antibody-associated vasculitis;Antineutrophil cytoplasmic antibody-associated vasculitis	intergenic	22808956
rs705471	10	3667726	ncRNA intronic	yunaro	Capecitabine sensitivity	intergenic	22864933
rs705469	10	3658919	ncRNA intronic	yunaro	Capecitabine sensitivity	intergenic	22864933
rs7044529	9	137568051	exonic	mortarby	Central corneal thickness;Corneal structure	intronic	20719862, 23291589
rs7032940	9	112945405	promoter	skawserby	Height	intergenic	19893584
rs7023329	9	21816528	UTR5	MTAPandCDKN2BAS	Melanoma	intronic	21983787, 19578364
rs7014346	8	128424792	ncRNA intronic	LOC727677	Colorectal cancer	intergenic	18372901
rs701428	22	20228542	promoter	noybo	Obesity-related traits	downstream	23251661
rs700752	7	46753553	intronic	flaku	Insulin-like growth factors	intergenic	21216879
rs700750	7	46753491	intronic	flaku	Obesity-related traits	intergenic	23251661
rs7000734	8	96127030	intronic	swubawby	Radiation response	intergenic	20923822

rs6993813	8	120052238	intronic	COLEC10	Bone mineral density (hip)	intergenic	18445777
rs6990255	8	34126948	ncRNA intronic	shyvuby	Bipolar disorder	intergenic	19416921
rs6987702	8	126504726	ncRNA intronic	kavoyby	LDL cholesterol;"Cholesterol, total"	intergenic	19060911
rs6984305	8	9178268	intronic	woyblerby	Liver enzyme levels (alkaline phosphatase)	intergenic	22001757
rs6983267	8	128413305	ncRNA intronic	LOC727677	Prostate cancer;Colorectal cancer	intergenic	21743057, 21242260, 18372905, 18264097, 18264096, 17618284, 17401363, 23266556
rs6972204	7	2582452	UTR5;UTR3	C7orf27;C7orf27	Obesity-related traits	intronic	23251661
rs6952808	7	1886535	UTR5	LOC100128374	Bipolar disorder and schizophrenia	intronic	20889312
rs6951258	7	37757556	intronic	GPR141andTXNDC3andEPDR1	Obesity-related traits	intergenic	23251661
rs694739	11	64097233	ncRNA intronic	jawnu	Crohn's disease;Alopecia areata	intergenic	21102463, 20596022
rs6942328	6	4512979	ncRNA intronic	spajey	Uric acid levels	intergenic	21294900
rs6927022	6	32612397	UTR3	HLA-DQA1	Ulcerative colitis	downstream	23128233
rs6918981	6	34238514	intronic	glawspyby	Height	intergenic	19893584, 19396169
rs6911490	6	106522027	intronic	PRDM1	Ulcerative colitis	intergenic	21297633
rs6907728	6	132228769	intronic	kawame	Obesity-related traits	intergenic	23251661
rs6905288	6	43758873	ncRNA intronic	cherbloy	Waist-hip ratio;Coronary heart disease	intergenic	22319020, 20935629
rs6904029	6	29943067	exonic	HCG9	Vitiligo	ncRNA exonic	20410501
rs6903823	6	28322296	UTR5	ZNF323	Pulmonary function	intronic	21946350
rs6890853	5	35852311	promoter	IL7R	Primary biliary cirrhosis	intergenic	23000144
rs6887695	5	158822645	ncRNA	smargey	Crohn's disease	intergenic	17554261

			exonic				
rs6871626	5	158826792	ncRNA exonic	smargey	Ulcerative colitis;Inflammatory bowel disease	intergenic	21297633, 23128233
rs6867983	5	55854153	intronic	snogee	Triglycerides	intergenic	20864672
rs6846071	4	102402215	intronic	BANK1	C-reactive protein and white blood cell count	intergenic	22788528
rs6844339	4	14144208	ncRNA intronic	glogu	Obesity-related traits	intergenic	23251661
rs6843082	4	111718067	ncRNA exonic	titime	Stroke (ischemic);Atrial fibrillation	intergenic	23041239, 20173747
rs6834555	4	10062326	ncRNA intronic	dygo	Alzheimer's disease	intergenic	22005930
rs6834483	4	35983111	intronic	ARAP2	Obesity-related traits	intergenic	23251661
rs6825911	4	111381638	intronic	ENPEP	Blood pressure	intergenic	21572416
rs6809854	3	18784423	ncRNA intronic	spardorbo	Psoriasis	intergenic	20953190
rs6806528	3	69252899	UTR3	smawporbu	Celiac disease	intronic	20190752
rs6789987	3	187763398	ncRNA intronic	slerswoybu	Visceral adipose tissue/subcutaneous adipose tissue ratio;Visceral adipose tissue adjusted for BMI	intergenic	22589738
rs6782299	3	180550702	intronic	CCDC39	Schizophrenia	intergenic	19571811
rs6781182	3	34212166	ncRNA intronic	farbabo	Visceral adipose tissue adjusted for BMI	intergenic	22589738
rs6778194	3	153184656	ncRNA intronic	chorfeebu,keyura	Immune reponse to smallpox (secreted IFN-alpha)	intergenic	22610502
rs6775745	3	131813900	ncRNA intronic	nukume	Neutrophil count	intergenic	21507922
rs6774852	3	196078427	ncRNA exonic	lorsweyby	Obesity-related traits	intergenic	23251661
rs6753473	2	26526419	intronic	EPT1	Non-small cell lung cancer	intergenic	23144319
rs6751715	2	56363377	intronic	CCDC85A	HIV-1 control	intergenic	20041166
rs6740462	2	65667272	intronic	FLJ16124,varkobu	Inflammatory bowel disease	intergenic	23128233

rs6739054	2	11220562	ncRNA intronic	stokla	Cognitive performance	intergenic	20125193
rs6736997	2	235615197	ncRNA intronic	nygloy	Pancreatic cancer	intergenic	20686608
rs6735786	2	103770238	ncRNA intronic	himiyo	Bone mineral density (hip)	intergenic	19079262
rs6733379	2	34480075	ncRNA intronic	sleelo	Attention deficit hyperactivity disorder and conduct disorder	intergenic	18951430
rs6723108	2	135479980	intronic	TMEM163	Type 2 diabetes	intergenic	23209189
rs6721654	2	121301911	promoter	kumemo	Age-related macular degeneration	intergenic	23326517
rs6720394	2	111989372	intronic	LOC541471	Primary sclerosing cholangitis	intergenic	21151127
rs6718520	2	43325570	intronic	norfa	Multiple sclerosis	intergenic	22190364
rs6716455	2	151126403	ncRNA intronic	larly	Alcoholism (alcohol use disorder factor score);Alcoholism (alcohol dependence factor score)	intergenic	21529783
rs6708166	2	30526780	intronic	LBH	vWF and FVIII levels	intergenic	21810271
rs6705628	2	74208362	UTR5	flarklar	Systemic lupus erythematosus	intergenic	23273568
rs6703335	1	243608967	ncRNA exonic	dumerbu	Schizophrenia	intronic	21926974
rs6699417	1	89123443	ncRNA intronic	borbar	Height	intergenic	20881960
rs669607	3	28071444	intronic	mikeme	Multiple sclerosis	intergenic	21833088
rs6693882	1	96145968	intronic	rorda	Pain	intergenic	19207018
rs6677208	1	239601969	intronic	CHRM3	Platelet counts	intergenic	21507922
rs665440	11	128582557	ncRNA exonic	jarchor	Myopia (pathological)	intronic	23049088
rs6651252	8	129567181	intronic	sluskobu	Crohn's disease	intergenic	21102463, 23128233
rs6607284	17	36026335	intronic	rinari	Bipolar disorder and schizophrenia	intergenic	20889312
rs6604026	1	93303603	UTR3	RPL5andSNORD21andSNORA66	Multiple sclerosis	intronic	19525955, 17660530
rs6603109	19	7424528	intronic	ARHGEF18	Sexual	intergenic	22445761

					dysfunction (SSRI/SNRI-related)		
rs6600671	1	121200490	ncRNA intronic	klorbey	Hip geometry	intergenic	17903296
rs6596140	5	133021851	intronic	FSTL4	Hypertension	intergenic	22384028
rs6586513	1	17216331	promoter	glupla	Allergic rhinitis	intergenic	22036096
rs6586282	21	44478497	exonic	CBS	Homocysteine levels	intronic	20031578
rs6585436	10	119210375	ncRNA intronic	kehoro	Cardiac repolarization	intergenic	22683750
rs6584283	10	101290301	ncRNA intronic	terpeeby	Ulcerative colitis	intergenic	21297633, 20228798, 19915572
rs6584202	10	100170383	UTR5	HPS1andPYROXD2	Obesity-related traits	intronic	23251661
rs6583826	10	94347830	intronic	KIF11	Type 2 diabetes	intergenic	21490949
rs6576878	1	87705031	intronic	serfawbu	White blood cell types	intergenic	21738478
rs6574988	14	88559992	intronic	reysla	Bipolar disorder	intergenic	21254220
rs6574433	14	78786159	intronic	NRXN3	Cognitive performance	intergenic	20125193
rs6568433	6	106829537	intronic	AIM1	Longevity	intergenic	20834067
rs6565681	17	78348494	UTR3	RNF213	Moyamoya disease	ncRNA intronic	21048783
rs6563210	13	36476447	UTR3	lersherbu	Height	ncRNA intronic	21998595
rs6560517	9	79038170	intronic	RPSAP9andGCNT1	Dialysis-related mortality	intergenic	21546767
rs6558295	8	145139522	UTR3	GPAA1	Metabolic traits	intronic	21886157
rs6556756	5	163889280	ncRNA intronic	stagoy	Breast cancer	intergenic	17903305
rs6556416	5	158818745	ncRNA exonic	smargey	Ankylosing spondylitis	intergenic	21743469
rs6550435	3	36864489	promoter	pluswerby	Bipolar disorder	intergenic	21926972
rs6534347	4	123198435	UTR5	tergla	Type 1 diabetes	intronic	17554300
rs6532023	4	88773849	ncRNA intronic	ninayu	Bone mineral density	intergenic	22504420
rs6526555	X	26369899	intronic	blaku	Metabolite levels (HVA/MHPG ratio);Metabolite levels (5-HIAA/ MHPG Ratio)	intergenic	23319000
rs652520	6	93713805	ncRNA intronic	tasaya	Chronic obstructive pulmonary	intergenic	23144326

					disease-related biomarkers		
rs6511720	19	11202306	UTR5	bloflor	Metabolite levels;Lipoprotein-associated phospholipase A2 activity and mass;LDL cholesterol;"Cholesterol, total";Carotid intima media thickness;Cardiovascular disease risk factors	intronic	22916037, 22003152, 21943158, 21909108, 20686565, 19060906, 18193044, 18193043
rs6503525	17	38095174	intronic	wanima	Asthma	intergenic	21150878
rs650258	11	60832282	ncRNA intronic	torchor	Multiple sclerosis	intergenic	21833088
rs6499188	16	68674788	intronic	ZFP90andCDH3	Ulcerative colitis	intergenic	21297633
rs6490294	12	112190438	UTR5	ACAD10	Mean platelet volume	intronic	22423221
rs6486986	12	19801595	ncRNA intronic	shoyzeeby	Cognitive performance	intergenic	19734545
rs6484218	11	10390581	intronic	AMPD3	Schizophrenia, bipolar disorder and depression (combined)	intergenic	20713499
rs6469804	8	120044829	intronic	COLEC10	Bone mineral density (spine)	intergenic	18445777
rs6468852	8	103975989	intronic	chorkey	Alzheimer's disease (late onset)	intergenic	22881374
rs6468544	8	98329765	ncRNA intronic	yuhare	Antipsychotic-induced QTc interval prolongation	intergenic	20921969
rs6458307	6	42731115	intronic	KIAA0240	Bipolar disorder	intergenic	17554300
rs645040	3	135926622	promoter	RPL31P23	Triglycerides	intergenic	20686565
rs6444931	3	170163474	intronic	CLDN11	Bipolar disorder and schizophrenia	intergenic	20889312
rs6442925	3	5013876	intronic	kirora	Corneal structure	intergenic	21979947
rs6442522	3	15440556	intronic	METTL6	Uric acid levels	intergenic	18759275
rs644234	9	136142217	ncRNA exonic	starteybu	Protein biomarker	intronic	23056639
rs6438424	3	117574822	intronic	swabarbu	Menarche (age	intergenic	21102462

					at onset)		
rs643434	9	136142355	ncRNA exonic	starteybu	Inflammatory biomarkers;End-stage coagulation	intronic	22291609, 23381943
rs6431731	2	15863002	intronic	nekere	Chronic kidney disease	intergenic	22479191
rs642803	11	65560620	UTR5	OVOL1	Urate levels	intronic	23263486
rs623323	17	700020	UTR5	klarroy	Type 2 diabetes	intergenic	23300278
rs62296075	4	1039215	intronic	suga	Obesity-related traits	intergenic	23251661
rs62209	10	11000339	intronic	CELF2	Alzheimer's disease (late onset)	intergenic	21379329
rs61744862	17	17068182	exonic	MPRIP	Obesity-related traits	intronic	23251661
rs6103489	20	42487204	ncRNA intronic	gysabu	Response to amphetamines	intergenic	22952603
rs6088765	20	33799280	intronic	EIF6andEDEM2, PROCR	Ulcerative colitis	intergenic	23128233
rs6085920	20	7180056	ncRNA intronic	neyvor	Uric acid levels	intergenic	18759275
rs6065904	20	44534651	UTR3	PLTP	Lipid metabolism phenotypes	intronic	22286219
rs606458	11	64546391	UTR5	SF1	Urate levels	upstream	21768215
rs606452	11	75276178	UTR5	SERPINH1	Height	intronic	22021425
rs6052699	20	4611877	intronic	RPS4XP2,boyto	Platelet aggregation	intergenic	20526338
rs6051520	20	351944	intronic	TRIB3	Information processing speed	intergenic	21130836
rs6050267	20	25019099	ncRNA exonic	pleyzy	Cognitive performance	intronic	20125193
rs6046396	20	19852503	intronic	RIN2	Bipolar disorder and schizophrenia	intergenic	20889312
rs6046346	20	19802188	intronic	RIN2	Obesity-related traits	intergenic	23251661
rs6045676	20	1941171	intronic	namori	Aortic root size	intergenic	21223598
rs6027755	20	59268710	ncRNA intronic	rarkey	Non-alcoholic fatty liver disease histology (other)	intergenic	20708005
rs6027511	20	58898209	intronic	dersubu,swyvey	Lentiform nucleus volume;Lentiform nucleus volume	downstream	22903471

rs6026584	20	57469073	UTR5	GNAS	Renal function-related traits (BUN)	intronic	22797727
rs6025590	20	56070505	intronic	CTCFLandHMGB1L1	Obesity-related traits	downstream	23251661
rs6024938	20	55109304	UTR3	C20orf107	Obesity-related traits	intronic	23251661
rs6017342	20	43065028	UTR5	staty	Ulcerative colitis	intergenic	21297633, 19915572, 23128233
rs601339	12	123174743	ncRNA intronic	weechy	Adiponectin levels	intergenic	22479202
rs600550	11	59997666	intronic	MS4A4E	Lipoprotein-associated phospholipase A2 activity and mass	intergenic	23118302
rs600231	11	65260646	ncRNA intronic	meeferby	Bone mineral density	intergenic	22504420
rs5945619	X	51241672	intronic	NUDT11	Prostate cancer	intergenic	18264097
rs5944185	X	25853614	ncRNA intronic	sawvar	Erectile dysfunction and prostate cancer treatment	intergenic	20932654
rs593982	11	65513107	promoter	mayaro	Atopic dermatitis	intergenic	23042114
rs5937496	X	75347434	ncRNA intronic	shormo	Amyotrophic lateral sclerosis	intergenic	19734901
rs5934953	X	11048692	intronic	perva	Cognitive performance	intergenic	19734545
rs5934683	X	9751474	intronic	GPR143	Colorectal cancer	intergenic	22634755
rs576523	1	160746076	ncRNA exonic	LOC100128858	Capecitabine sensitivity	intergenic	22864933
rs5762311	22	28090207	ncRNA intronic	shoyter	Sudden cardiac arrest	intergenic	21658281
rs5759167	22	43500212	intronic	TTLL1	Prostate cancer	intergenic	19767753
rs5753037	22	30581722	intronic	slardoy	Type 1 diabetes	intergenic	19430480
rs5749446	22	32880585	UTR3	FBXO7	Red blood cell traits	intronic	23222517
rs573872	3	153472163	ncRNA intronic	chorfeebu	Infantile hypertrophic pyloric stenosis	intergenic	22306654
rs569688	8	60961821	promoter	skerkor	Myopia (pathological)	intergenic	23049088
rs563694	2	169774071	intronic	ABCB11	Fasting plasma glucose	intergenic	18521185
rs56238310	3	111233239	ncRNA	hakume	Chronic	intergenic	21685187

			intronic		obstructive pulmonary disease		
rs560887	2	169763148	UTR5	G6PC2	Metabolite levels;Metabolic traits;Metabolic syndrome;Fasting plasma glucose;Fasting glucose-related traits (interaction with BMI);Fasting glucose-related traits	intronic	22916037, 22581228, 22508271, 22399527, 22286219, 20081858, 19060910, 19060907, 18451265
rs548726	1	5913621	promoter	lobey	Response to taxane treatment (placlitaxel)	intergenic	23006423
rs548181	11	125461709	intronic	FEZ1	Schizophrenia	intergenic	21926974
rs543686	15	35068309	ncRNA intronic	nerspey	Prostate cancer (gene x gene interaction)	intergenic	22219177
rs529866	16	11373320	intronic	C16orf75	Inflammatory bowel disease	intergenic	23128233
rs528301	2	45154908	intronic	LOC100130502	Alcohol and nictotine co-dependence	intergenic	22488850
rs527409	1	58757915	ncRNA intronic	sparrubo	Kawasaki disease	intergenic	21221998
rs525455	10	13103285	intronic	CCDC3	Platelet aggregation	intergenic	20526338
rs519113	19	45376284	UTR3	sawber	HDL cholesterol	intronic	21909109
rs516246	19	49206172	UTR3	MAMSTR	Liver enzyme levels (gamma-glutamyl transferase);Obesity-related traits;Crohn's disease	intronic	22001757, 23251661, 23128233
rs514659	9	136142203	ncRNA exonic	starteybu	Coronary heart disease	intronic	21239051
rs513349	6	33541719	UTR3	BAK1	Platelet counts	intronic	23263863
rs508487	11	117075566	UTR3	TAGLN	Cardiovascular disease risk factors	downstream	21943158
rs4996815	13	106651661	promoter	kawderby	Bipolar disorder and schizophrenia	intergenic	20889312

rs4985167	16	15082865	exonic	LOC728138	Phospholipid levels (plasma)	intronic	21829377
rs4972755	2	176239849	ncRNA intronic	wadar	Bipolar disorder and major depressive disorder (combined)	intergenic	20351715
rs4968031	16	23765774	UTR3	raseru	Platelet counts	upstream	21507922
rs4963128	11	589564	UTR3	flawmorby	Systemic lupus erythematosus	intronic	21408207, 18204446
rs496300	21	44779680	intronic	slotaw	Metabolic syndrome	intergenic	20694148
rs4955755	3	170494409	intronic	CLDN11	Menopause (age at onset)	intergenic	19448619
rs4954218	2	135803425	intronic	YSK4	Corneal structure	intergenic	21979947
rs4950806	1	201952574	UTR5	RNPEP	Butyrylcholinesterase levels	intronic	21862451
rs4938534	11	111275133	intronic	POU2AF1	Primary biliary cirrhosis	intergenic	23000144
rs4937126	11	126281897	UTR3	ST3GAL4	Coronary heart disease	intronic	21378990
rs4932217	15	89902032	promoter	LOC100288864,seyswy	Height	intergenic	18193045
rs4931594	12	32150243	promoter	jarshy	Obesity-related traits	intergenic	23251661
rs4924935	17	18753870	intronic	PRPSAP2	Pancreatic cancer	intergenic	20686608
rs4910742	11	5306509	intronic	OR51B5andHBG2andHBG1	Inflammatory biomarkers;Fetal hemoglobin levels	intergenic	22291609, 18245381
rs4907240	2	97228039	intronic	KIAA1310	Event-related brain oscillations	intergenic	21184583
rs4903031	14	73019236	UTR3	RGS6	C-reactive protein	intronic	21300955
rs4902647	14	69254191	UTR3	C14orf181andZFP36L1	Multiple sclerosis	downstream	21833088
rs4901869	14	59334128	intronic	kerpo	Panic disorder	intergenic	19165232
rs4894410	3	139043236	intronic	MRPS22	Kawasaki disease	intergenic	22446961
rs489332	9	78028346	ncRNA intronic	syteeby	Schizophrenia	intergenic	23212062
rs4886707	15	75755467	promoter	moystor	Height	intergenic	20189936
rs4886088	13	59128526	ncRNA intronic	sweezoy	Subcutaneous adipose tissue	intergenic	22589738
rs4872511	8	22400989	ncRNA	morkoy	HIV-1 control	intergenic	20205591

			exonic				
rs4871750	8	127902003	ncRNA intronic	bleyvyby	Obesity-related traits	intergenic	23251661
rs4871611	8	126537570	ncRNA intronic	kavoyby,ryla	Crohn's disease	intergenic	21102463
rs4871297	8	123706155	UTR5	mureya	Type 1 diabetes nephropathy	intergenic	23028342
rs4870684	7	57699151	ncRNA intronic	LOC100128575	Bipolar disorder and schizophrenia	intergenic	20889312
rs4861096	4	41284711	ncRNA intronic	floyblerby	Cognitive performance	intergenic	19734545
rs485499	3	159745863	intronic	voyskawby	Primary biliary cirrhosis	intergenic	21399635
rs4852324	2	74202578	intronic	flarklar	Systemic lupus erythematosus	intergenic	23273568
rs4849887	2	121245122	ncRNA exonic	semayo	Breast size	intergenic	22747683
rs4845812	1	5707816	ncRNA intronic	sheyrerbo	Metabolite levels (MHPG)	intergenic	23319000
rs4839680	3	143902027	intronic	C3orf58	Myopia (pathological)	intergenic	23049088
rs4827947	X	97864354	ncRNA intronic	seedy	Immune reponse to smallpox (secreted IL-1beta)	intergenic	22610502
rs482329	1	234816554	ncRNA exonic	sami	Life threatening arrhythmia	intergenic	22247754
rs4821469	22	36616445	ncRNA exonic	beezer	End-stage renal disease (non-diabetic)	intergenic	20532800
rs4819143	21	47124178	intronic	PCBP3	Insulin resistance/response	intergenic	21901158
rs4815617	20	3827309	exonic	C20orf29andMAVS	Asthma	upstream	20159242
rs4813802	20	6699595	intronic	pygar	Colorectal cancer	intergenic	23266556
rs4812466	20	39439483	ncRNA intronic	kotera	Metabolite levels (HVA-5-HIAA Factor score)	intergenic	23319000
rs4809324	20	62318220	UTR5	RTEL1andTNFRSF6B	Glioma (high-grade)	ncRNA intronic	19578366
rs4806073	19	35555190	UTR5	HPN	Serum albumin level	ncRNA intronic	23022100
rs4803480	19	42066279	intronic	CEACAM21	Schizophrenia	intergenic	21682944
rs4800279	18	25070355	intronic	wahima	White matter	intergenic	23218918

					integrity		
rs4799088	18	77573548	ncRNA intronic	skarsu	Amyotrophic lateral sclerosis	intergenic	20801717
rs479777	11	64107477	ncRNA exonic	jawnu	Sarcoidosis	upstream	22837380
rs4792394	17	13684917	ncRNA intronic	moteru	Conduct disorder (symptom count)	intergenic	20585324
rs4790881	17	2068932	ncRNA exonic	kyskey	Bone mineral density	intronic	22504420
rs4785763	16	90066936	exonic	AFG3L1	Melanoma	ncRNA exonic	19578364
rs4785204	16	50103734	ncRNA exonic	foyspey	Esophageal cancer (squamous cell)	intronic	22960999
rs4784165	16	52347819	intronic	perworbu	Polycystic ovary syndrome	intergenic	22885925
rs4783227	16	82594383	ncRNA intronic	chorry	Response to antipsychotic treatment	intergenic	20195266
rs4780355	16	11347858	intronic	C16orf75	Crohn's disease and psoriasis	downstrea m	22482804
rs4777845	15	93877425	ncRNA intronic	nosoru	Adiponectin levels	intergenic	22479202
rs4777542	15	73082366	UTR3	glydee	Urate levels	ncRNA intronic	23263486
rs4773144	13	110960712	exonic	slozoy	Coronary heart disease	intronic	21378990
rs4760790	12	71634794	intronic	TSPAN8	Type 2 diabetes	intergenic	20581827
rs4760636	12	48173352	UTR3	SLC48A1	Urate levels	intronic	23263486
rs4751178	10	131992245	intronic	GLRX3	Speech perception in dyslexia	intergenic	19786962
rs4750316	10	6393260	UTR3	DKFZp667F0711	Rheumatoid arthritis	intergenic	20453842, 18794853
rs4749791	10	8633861	intronic	sweega	Obesity-related traits	intergenic	23251661
rs4745062	9	73784264	intronic	flaspar	Longevity	intergenic	20834067
rs4743820	9	93928416	intronic	lawsuby	Inflammatory bowel disease	intergenic	23128233
rs4732038	7	134250322	UTR5	zukee	Longevity	intronic	20834067
rs472913	1	61095558	intronic	gorbey	Bipolar disorder	intergenic	19416921
rs4722672	7	27231762	ncRNA intronic	bucherby	Ulcerative colitis	intergenic	23128233
rs4716055	6	9853919	intronic	OFCC1	Adiponectin levels	intergenic	22479202
rs4711336	6	33659046	UTR5	ITPR3	Height	intronic	20881960

rs4705952	5	131839618	intronic	C5orf56andRAD50	C-reactive protein	intergenic	21300955
rs4704970	5	155500992	intronic	pucheyby	Multiple sclerosis (age of onset)	intergenic	19010793
rs4702982	5	99991648	intronic	swoger	Panic disorder	intergenic	19165232
rs4702435	5	7155612	ncRNA intronic	tireyu	Obesity-related traits	intergenic	23251661
rs4698412	4	15737348	intronic	FAM200BandBST1	Parkinson's disease	intergenic	22451204, 21084426
rs4698169	4	17135343	ncRNA intronic	slorgu	Temperament (bipolar disorder)	intergenic	22365631
rs4693089	4	84373622	UTR3	FAM175AandHELQ	Menopause (age at onset)	intronic	22267201
rs4689388	4	6270056	promoter	WFS1	Type 2 diabetes and other traits	intergenic	19734900
rs4686914	3	187717540	ncRNA intronic	slerswoybu	Metabolite levels	intergenic	21909109
rs4684585	3	883851	ncRNA intronic	sneydubo	Response to statin therapy	intergenic	20339536
rs4670779	2	38044344	exonic	STRAP.1	Bone mineral density (spine)	intergenic	19079262
rs4665058	2	160190209	UTR5	BAZ2B	Sudden cardiac arrest	intronic	21738491
rs4663476	2	235601064	ncRNA intronic	nygloy	Response to angiotensin II receptor blocker therapy	intergenic	22566498
rs4660293	1	40028180	UTR5;UTR3	PABPC4andSNORA55;PABPC4andSNORA55	HDL cholesterol	intronic	20686565
rs4657616	1	158971086	intronic	IFI16	Hematology traits	intergenic	23263863
rs4656958	1	160856964	intronic	ITLN1	Inflammatory bowel disease	intergenic	23128233
rs4654748	1	21786068	ncRNA exonic	LOC767853	Folate pathway vitamin levels	intronic	19303062
rs46522	17	46988597	UTR5	UBE2Z	Coronary heart disease	intronic	21378990
rs4646949	6	34845449	UTR3	UHRF1BP1	Fasting insulin-related traits (interaction with BMI)	downstream	22581228
rs4624519	3	36862980	intronic	pluswerby	Schizophrenia	intergenic	21926974
rs4622329	12	102321935	intronic	DRAM1	Systemic lupus erythematosus	intergenic	23273568
rs4621553	5	113030164	ncRNA	boystorby	Sudden cardiac	intergenic	21658281

			intronic		arrest		
rs4615179	4	38465471	ncRNA intronic	teki	Obesity-related traits	intergenic	23251661
rs456867	5	55811092	intronic	snogee	Urate levels	intergenic	23263486
rs4557101	3	73540618	ncRNA exonic	peeswerby	QT interval	intronic	23166209
rs4538475	4	15737937	intronic	FAM200BandBST1	Parkinson's disease	intergenic	19915576
rs4529888	11	127081591	ncRNA intronic	smuglybu	Amyotrophic lateral sclerosis	intergenic	22959728
rs4516970	6	160137687	ncRNA intronic	darvoyby	Iron status biomarkers	intergenic	19084217
rs4489787	12	48811100	intronic	swojeyby	Prostate cancer (gene x gene interaction)	intergenic	22219177
rs4479806	5	26723783	intronic	fystybu	Anorexia nervosa	intergenic	21079607
rs4470914	7	19616522	ncRNA intronic	yahiya	Height	intergenic	20881960
rs445114	8	128323181	ncRNA intronic	LOC727677	Prostate cancer	intergenic	21743057, 19767754
rs4434872	1	153774276	exonic	stoyrubo	Conduct disorder (symptom count)	intergenic	20585324
rs4410871	8	128815029	intronic	PVT1	Multiple sclerosis	intergenic	21833088
rs4392868	8	96120072	intronic	swubawby	Radiation response	intergenic	20923822
rs4382726	1	209334217	ncRNA intronic	klawbee	Response to amphetamines	intergenic	22952603
rs4380028	15	79111093	intronic	MORF4L1	Coronary heart disease	intergenic	21378988
rs435746	16	77916191	UTR3	VAT1L	Bipolar disorder and schizophrenia	intronic	20889312
rs4352210	2	37750980	ncRNA intronic	jerdaw	RR interval (heart rate)	intergenic	20031603
rs4349859	6	31365787	intronic	noyjey	Ankylosing spondylitis	intergenic	21743469
rs4349147	16	3124920	intronic	IL32,LOC100132433	HIV-1 susceptibility	intergenic	22174851
rs4325129	1	159462381	ncRNA intronic	bleekoybu	Obesity-related traits	intergenic	23251661
rs4324798	6	28776117	intronic	fawjaw	Lung adenocarcinoma	intergenic	19836008
rs4322600	14	88295600	intronic	deymaby,tawpar	Breast cancer	intergenic	22923054
rs430727	3	41128564	ncRNA	jerfawbo	Bone mineral	intergenic	22504420

			intronic		density		
rs4281086	8	10352308	intronic	LOC346702	Obesity-related traits	intergenic	23251661
rs4273712	6	126964510	promoter	LOC728666	Intracranial volume	intergenic	22504418
rs4269383	6	156197502	ncRNA intronic	stajee	Pancreatic cancer	intergenic	22158540
rs4256159	3	18767404	ncRNA intronic	spardorbo	Inflammatory bowel disease	intergenic	23128233
rs425215	21	43707101	exonic	stato	Common traits (Other)	intronic	20585627
rs4246905	9	117553249	exonic	TNFSF15	Ulcerative colitis;Inflammatory bowel disease	intronic	21297633, 23128233
rs4236601	7	116162729	intronic	CAV2andCAV1	Glaucoma (primary open-angle)	intergenic	20835238
rs422421	5	176517326	UTR5	FGFR4	Height	intronic	20881960
rs420017	15	89617081	intronic	lersky	Height;Obesity-related traits	intergenic	21998595, 23251661
rs416603	16	11364079	intronic	C16orf75	Type 1 diabetes	upstream	18978792
rs416350	12	68779413	intronic	yuhumi	Temperament (bipolar disorder)	intergenic	22365631
rs415890	6	167406633	exonic	sheysnaby	Crohn's disease	intergenic	21102463
rs4149178	6	43272188	UTR3	SLC22A7	Urate levels	intronic	23263486
rs4147141	1	69579252	ncRNA intronic	bawkley	Inattentive symptoms;Attention deficit hyperactivity disorder	intergenic	18821565
rs4143832	5	131862977	intronic	C5orf56andRAD50	Eosinophil counts	intergenic	19198610
rs4142248	19	44888276	UTR3	ZNF229andZNF285andZFP112	Myopia (pathological)	intergenic	23049088
rs4133289	1	159453937	ncRNA intronic	bleekoybu	Hemostatic factors and hematological phenotypes	intergenic	17903294
rs4105144	19	41358624	intronic	MIA	Smoking behavior	intergenic	20418888
rs405460	14	59366315	intronic	kerpo	Obesity-related traits	intergenic	23251661
rs404860	6	32184345	UTR3	NOTCH4andGPSM3	Asthma	intronic	21804548
rs402710	5	1320722	UTR3	CLPTM1L	Lung cancer	intronic	18978790
rs4006360	17	39250639	intronic	skarvybu	Bipolar disorder	intergenic	20889312

					and schizophrenia		
rs399885	2	79687252	intronic	CTNNA2	Response to antipsychotic treatment	intergenic	20195266
rs399604	6	32975014	exonic	HLA-DOA	Platelet counts	intronic	22139419
rs397969	17	19804247	promoter	skeywey	Platelet counts	intergenic	22139419
rs3930234	3	184216130	intronic	EIF2B5	Alcoholism (alcohol use disorder factor score)	intergenic	21529783
rs3925075	16	31347748	promoter	lospaw	IgA nephropathy	intergenic	22197929
rs3923564	10	81735981	intronic	SFTPD	Chronic obstructive pulmonary disease-related biomarkers	intergenic	23144326
rs3919627	3	42909160	UTR3	CCBP2andZNF662andFAM198A	Obesity-related traits	downstream	23251661
rs3916765	6	32685550	promoter	plartoybo	Type 2 diabetes	intergenic	22693455
rs3913363	3	171653903	ncRNA intronic	skeeswoybu	Response to angiotensin II receptor blocker therapy	intergenic	22566498
rs3903239	1	170569317	ncRNA intronic	kime	Atrial fibrillation	intergenic	22544366
rs389884	6	31940897	UTR3	STK19andC4A	Hematology traits	intronic	23263863
rs3893464	6	29935250	intronic	ZNRD1	Graves' disease	intergenic	21900946
rs3892630	19	33181484	intronic	flawwa	Red blood cell traits	intergenic	23222517
rs3864639	7	76738591	intronic	FAM185B	Obesity-related traits	intergenic	23251661
rs3853601	6	31499603	UTR3	ATP6V1G2andBAT1andSNORD84	Atopic dermatitis	ncRNA intronic	22197932
rs3850370	14	78534906	ncRNA intronic	soyshabu	Response to platinum-based chemotherapy in non-small-cell lung cancer	intergenic	22872573
rs3849491	3	76484183	intronic	ROBO2	Sex hormone-binding globulin levels	intergenic	22675492
rs3845817	2	65758525	intronic	FLJ16124	Bipolar disorder	intergenic	21926972
rs3827886	12	14660184	ncRNA exonic	weeblybu	Response to amphetamines	intronic	22952603
rs3825776	15	58746830	ncRNA	supee	Amyotrophic	intronic	18084291

			exonic		lateral sclerosis		
rs3820201	1	53581670	UTR3	seyuro	Hippocampal atrophy	intronic	22745009
rs3819299	6	31322367	UTR3	HLA-BandHLA-C	Platelet counts	intronic	22139419
rs3816587	4	25417244	UTR3	ANAPC4	Rheumatoid arthritis	intronic	17554300
rs3814219	10	105647095	ncRNA exonic	nawsho	Endothelial function traits	intronic	17903301
rs3813948	1	207269858	UTR3	C4BPB	C4b binding protein levels	intronic	20212171
rs3813582	16	79749353	ncRNA intronic	narar	Thyroid function	intergenic	22494929
rs3813579	16	79749276	ncRNA intronic	narar	Thyroid volume	intergenic	21565293
rs3812163	6	7725760	promoter	BMP6	Height	intergenic	20881960
rs3810265	19	50223266	UTR5	RPS9P4	Panic disorder	intergenic	19165232
rs3809566	15	63333724	promoter	TPM1	Platelet counts	intergenic	22139419
rs3809346	13	110960943	exonic	slozoy	Coronary artery calcification	intronic	22144573
rs3806932	5	110405675	promoter	TSLP	Eosinophilic esophagitis (pediatric)	intergenic	20208534
rs3806308	1	20142866	intronic	choybo	Ulcerative colitis	intergenic	19122664
rs3803662	16	52586341	exonic	LOC643714	Breast cancer (male);Breast cancer;Breast cancer (male)	ncRNA exonic	23001122, 20872241, 20453838, 19330030, 17529974, 17529967
rs3803064	12	113173494	intronic	RPH3A	Platelet counts	intergenic	21507922
rs3796804	4	82009707	ncRNA exonic	laflor	HIV-1 susceptibility	downstream	22174851
rs3795578	1	204112984	exonic	mimo	Response to acetaminophen (hepatotoxicity)	intronic	21177773
rs3790455	1	156456301	ncRNA exonic	woypla	Migraine	intronic	22683712
rs378108	21	40469520	intronic	PSMG1	Ankylosing spondylitis	intergenic	21743469
rs3779195	7	97993362	ncRNA exonic	zorkar	Sex hormone-binding globulin levels	intronic	22829776
rs3771180	2	102953617	UTR5	IL1RL1andIL18R1	Asthma	intronic	21804549
rs3764913	2	211074909	exonic	ACADL	Metabolite levels	intronic	23281178

rs3764640	19	1207238	UTR3	STK11	Alzheimer's disease	intronic	22005930
rs3764628	19	18747605	UTR5	KLHL26	Orofacial clefts	upstream	22419666
rs3764563	19	15724203	UTR5	rakora	Inflammatory biomarkers	intergenic	22228203
rs3764400	17	46123932	intronic	COPZ2	Body mass index	intergenic	20935630
rs3761847	9	123690239	UTR5	TRAF1	Rheumatoid arthritis	intronic	20453842, 17804836
rs3761218	20	3776175	intronic	CDC25B	Bipolar disorder	upstream	21254220, 17554300
rs3760776	19	5839746	UTR5	FUT6	Vitamin B12 levels;N-glycan levels;Tumor biomarkers	upstream	22367966, 21203500, 23300138
rs3760318	17	29247715	intronic	ADAP2	Height	intergenic	18391951
rs3758354	9	75764565	ncRNA intronic	slashee	Schizophrenia, bipolar disorder and depression (combined)	intergenic	20713499
rs3757354	6	16127407	promoter	koreme	LDL cholesterol;"Cholesterol, total"	intergenic	20686565
rs3753394	1	196620917	UTR5	CFH	Complement C3 and C4 levels	upstream	23028341
rs3747767	6	80257281	UTR3	najo	Hoarding	intergenic	21302353
rs3744728	17	6093951	promoter	sherdee	Bipolar disorder (mood-incongruent)	intergenic	23092984
rs3744028	17	73888672	exonic	TRIM65	White matter hyperintensity burden	intronic	21681796
rs3741920	12	6938872	UTR5	LEPREL2	Response to Vitamin E supplementation	intronic	22437554
rs3738443	1	247348189	ncRNA intronic	meyplo	Alcohol dependence	intergenic	21314694
rs3734905	6	169958982	UTR3	WDR27	HIV-1 control	intronic	20041166
rs3729931	3	12626516	UTR3	MKRN2	Cardiac hypertrophy	intronic	21348951
rs371915	16	84578241	intronic	KIAA1609	Eosinophilic esophagitis (pediatric)	intergenic	20208534
rs37060	16	58566304	UTR5	CNOT1	QT interval	intronic	22726844
rs367615	5	108948937	ncRNA intronic	sayayu	Colorectal cancer	intergenic	23300701
rs365836	7	101809851	UTR3	glyblee	Response to antidepressants	intronic	22584459

rs361433	7	142104571	exonic	ritemu	Capecitabine sensitivity	intergenic	22864933
rs35964523	3	898661	promoter	snoydubo	Response to statin therapy	intergenic	20339536
rs35675666	1	8021973	UTR5	PARK7	Ulcerative colitis;Inflammatory bowel disease	intronic	21297633, 23128233
rs356220	4	90641340	ncRNA intronic	saso	Parkinson's disease	intergenic	22451204, 21738487, 21084426, 21044948, 20711177
rs356219	4	90637601	ncRNA intronic	saso	Parkinson's disease	intergenic	22438815, 21292315
rs35600665	15	24741291	ncRNA intronic	PWRN1	Obesity-related traits	intergenic	23251661
rs34924084	16	5003074	intronic	PPL	Metabolite levels (HVA/MHPG ratio)	intergenic	23319000
rs342296	7	106372903	intronic	sokaw	Mean platelet volume	intergenic	22423221
rs342293	7	106372219	intronic	sokaw	Mean platelet volume	intergenic	22423221, 22139419, 19820697, 19221038
rs342275	7	106359216	intronic	sokaw	Platelet counts	intergenic	22139419
rs340874	1	214159256	UTR3	skerbo	Fasting glucose-related traits (interaction with BMI);Fasting glucose-related traits	intergenic	22581228, 20081858
rs322458	3	120585315	ncRNA intronic	tusuyu	Aging (facial)	intergenic	23223146
rs314370	7	100453208	UTR5	SLC12A9	Resting heart rate	intronic	20639392
rs314280	6	105400837	intronic	LIN28B	Menarche (age at onset)	intergenic	19448622
rs3134792	6	31312326	intronic	HLA-BandHLA-C	Psoriasis	intergenic	18364390
rs3132613	6	30537606	intronic	GNL1	Graves' disease	intergenic	21900946
rs3130573	6	31106268	UTR3	PSORS1C1	Systemic sclerosis	intronic	21750679
rs3130340	6	32244627	intronic	RNF5	Bone mineral density (spine)	intergenic	18445777
rs3130320	6	32223258	intronic	RNF5	Systemic lupus	intergenic	21408207

					erythematosus		
rs312691	17	68326338	ncRNA intronic	zuwey	Thyrotoxic hypokalemic periodic paralysis	intergenic	22863731
rs3126085	1	152300817	intronic	ruyo	Atopic dermatitis	intergenic	21666691
rs3123078	10	51524971	intronic	LOC10431	Prostate cancer	intergenic	19767753
rs3120665	1	152316590	intronic	ruyo	Personality dimensions	intergenic	20691247
rs3119939	13	63638329	intronic	wersta	Asthma	intergenic	21907864
rs3117582	6	31620520	UTR5	APOM	Lung cancer;Lung adenocarcinoma	intronic	19836008, 19654303, 18978787
rs3117242	6	33069893	intronic	HLA-DPB1	Antineutrophil cytoplasmic antibody-associated vasculitis;Antineutrophil cytoplasmic antibody-associated vasculitis	intergenic	22808956
rs3117099	6	32358270	UTR3	RNF5	Bipolar disorder and schizophrenia	upstream	22688191
rs3117035	6	33086249	exonic	HLA-DPB2	RR interval (heart rate)	ncRNA intronic	20031603
rs3115573	6	32218843	intronic	RNF5	Nephropathy	intergenic	20595679
rs3113494	4	87832601	promoter	feegy	Amyotrophic lateral sclerosis	intergenic	22470424
rs3110496	17	27917771	intronic	ANKRD13B,GIT1	Height	intergenic	20881960
rs3099844	6	31448976	ncRNA exonic	nanure	Neonatal lupus;Metabolic syndrome	intergenic	22399527, 20662065
rs3094548	6	29355202	intronic	OR11A1andOR5V1	Pulmonary function	intergenic	21946350
rs3093030	19	10397403	ncRNA exonic	jupo	Soluble levels of adhesion molecules	upstream;downstream	20167578
rs3025343	9	136478355	UTR3	kleeskabu	Smoking behavior	intergenic	20418890
rs2995271	10	30519832	ncRNA intronic	suloy	Pancreatitis	intergenic	23143602
rs2981579	10	123337335	exonic	myraby	Breast cancer	intronic	20453838, 19330030

rs2980879	8	126481475	ncRNA intronic	kavoyby	Adiponectin levels	intergenic	22479202
rs2979481	8	30262786	ncRNA exonic	mayaya	Heart rate variability traits	intronic	22174390
rs297941	12	50319086	ncRNA exonic	zynee	Obsessive-compulsive disorder	intergenic	22889921
rs2967951	5	10464107	exonic	ROPN1L	Body mass index	intronic	22446040
rs296547	1	200892137	intronic	C1orf81	Celiac disease	intergenic	20190752
rs2958154	12	57065713	UTR5	PTGES3	Age-related macular degeneration	intronic	20385819
rs2954038	8	126507389	ncRNA intronic	kavoyby	Response to statin therapy	intergenic	20339536
rs2954033	8	126493746	ncRNA intronic	kavoyby	Triglycerides-Blood Pressure (TG-BP)	intergenic	21386085
rs2954029	8	126490972	ncRNA intronic	kavoyby	Triglycerides;LDL cholesterol;HDL cholesterol;"Cholesterol, total"	intergenic	20864672, 20686565, 19060906
rs2954026	8	126484526	ncRNA intronic	kavoyby	HDL Cholesterol - Triglycerides (HDLC-TG)	intergenic	21386085
rs2954021	8	126482077	ncRNA intronic	kavoyby	Liver enzyme levels (alkaline phosphatase);Liver enzyme levels (alanine transaminase);LDL cholesterol	intergenic	22001757, 20864672
rs2953145	2	241515596	UTR5	RNPEPL1	Bipolar disorder	intronic	17554300
rs2935776	8	109629903	ncRNA intronic	mawzuby,pleekey	T-tau	intergenic	20932310
rs293428	4	69591782	intronic	LOC728811	Sex hormone-binding globulin levels	intergenic	22829776
rs2932538	1	113216543	intronic	MOV10	Systolic blood pressure;Hypertension;Diastolic blood pressure;Blood pressure	upstream	21909115, 21909110
rs29232	6	29611431	intronic	GABBR1andUBD	Nasopharyngeal carcinoma	intergenic	19664746

rs2923084	11	10388782	intronic	AMPD3	HDL cholesterol	intergenic	20686565
rs290986	9	93563536	intronic	SYK	Multiple sclerosis	upstream	21833088
rs2908835	12	11648418	intronic	PRB2	Information processing speed	intergenic	21130836
rs2905072	9	135845035	intronic	GFI1B	Bipolar disorder	intergenic	19416921
rs2904804	10	5009759	UTR3	AKR1E2andAKR1C1andAKR1C3	Economic and political preferences (immigration/crime)	intronic	22566634
rs2903698	4	76332975	ncRNA intronic	dorgar	Prion diseases	intergenic	22210626
rs290258	9	93555739	intronic	SYK	Prostate cancer (gene x gene interaction)	intergenic	22219177
rs2901964	1	15792426	UTR5	CELA2AandCELA2B	Erectile dysfunction and prostate cancer treatment	intronic	20932654
rs2900333	12	14653867	ncRNA exonic	sohemi	Testicular germ cell cancer	intergenic	20543847
rs2894207	6	31263751	ncRNA intronic	rosiya	Nasopharyngeal carcinoma	intergenic	20512145
rs2893923	10	65261184	intronic	soymaby	Platelet aggregation	intergenic	20526338
rs28890483	15	59719169	intronic	FAM81A	Bipolar disorder and schizophrenia	intergenic	20889312
rs2888830	7	90915354	ncRNA intronic	smysherby	Dental caries	intergenic	23259602
rs2887571	12	1638171	promoter	nerblar	Bone mineral density	intergenic	22504420
rs2877832	14	27800177	intronic	LOC728755	Diabetes related insulin traits	intergenic	17903298
rs2860580	6	29906691	intronic	HLA ,ZNRD1	Nasopharyngeal carcinoma	intergenic	20512145
rs2860031	1	30525714	ncRNA intronic	deeda	Bipolar disorder (mood-incongruent)	intergenic	23092984
rs2854160	17	61977248	intronic	GH1 ,wawlee	Height	intergenic	21998595
rs28493229	19	41224204	exonic	ITPKC	Kawasaki disease	intronic	22081228
rs2847297	18	12797694	UTR3	PTPN2	Rheumatoid arthritis	intronic	22446963
rs28417902	9	140088630	UTR5	TPRN	Obesity-related traits	intronic	23251661
rs2841498	9	87930045	ncRNA	snaterby	Partial epilepsies	intergenic	20522523

			intronic					
rs2836878	21	40465534	intronic	PSMG1	Ulcerative colitis;Inflammatory bowel disease;C-reactive protein	intergenic	21300955, 21297633, 18758464, 23128233
rs2836823	21	40380249	ncRNA intronic	blortaw	Nicotine dependence	intergenic	17158188
rs2836754	21	40291740	ncRNA intronic	FLJ45139	Crohn's disease	intergenic	17554261
rs2836326	21	39721764	ncRNA intronic	flaley	Metabolite levels (HVA)	intergenic	23319000
rs2834655	21	36238507	UTR5	swerser	Immune response to smallpox vaccine (IL-6)	intronic	22542470
rs2834442	21	35690786	intronic	MRPS6andSLC5A3andC21orf82	Height	intergenic	20881960
rs2833610	21	33385186	intronic	HUNK	Type 2 diabetes	intergenic	21490949
rs2833607	21	33381040	intronic	HUNK	Vitiligo	intergenic	19890347
rs2823743	21	17667720	ncRNA exonic	katira	Chronic obstructive pulmonary disease-related biomarkers	ncRNA intronic	23144326
rs2816316	1	192536813	ncRNA intronic	risimo	Celiac disease	intergenic	20190752, 18311140
rs2814944	6	34552797	promoter	spaspyby	HDL cholesterol	intergenic	20686565
rs2814828	9	90811182	intronic	snarsher	Height	intergenic	18391951
rs2814707	9	27536397	ncRNA intronic	lolee	Amyotrophic lateral sclerosis	intergenic	19734901
rs2814021	10	63361987	intronic	reesterby	Obesity-related traits	intergenic	23251661
rs281380	19	49214470	intronic	MAMSTR	Multiple sclerosis	intergenic	21833088
rs281379	19	49214274	intronic	MAMSTR	Crohn's disease	intergenic	21102463
rs2812378	9	34710260	intronic	fleyplobu	Rheumatoid arthritis	upstream	18794853
rs2797685	1	7879063	UTR5	PER3	Crohn's disease	intronic	21102463
rs2785197	11	35093070	intronic	bloglaw	Systemic lupus erythematosus	intergenic	23273568
rs2785173	11	35155732	ncRNA intronic	sweemaw	Response to Vitamin E supplementation	intergenic	22437554
rs2778031	9	90835726	intronic	snarsher	Height	intergenic	20881960
rs2774920	1	94611300	ncRNA intronic	glawroybo	D-dimer levels	intergenic	21502573
rs2766692	14	100684192	intronic	raniru	Electroencephal	intergenic	22554406

					ographic traits in alcoholism		
rs2764980	10	3284007	ncRNA intronic	beerarby	Attention deficit hyperactivity disorder and conduct disorder	intergenic	18951430
rs2762051	13	50835715	intronic	DLEU1	Celiac disease	intergenic	20190752
rs275437	5	6879978	ncRNA intronic	derskuby	Obesity-related traits	intergenic	23251661
rs274546	5	131699867	exonic	glarstorby	Height	ncRNA intronic	20881960
rs2742417	3	45731451	UTR5	SACM1L	Response to antidepressant treatment	intronic	22041458
rs2739330	22	24295286	intronic	GSTTP1andDDT	Liver enzyme levels (gamma-glutamyl transferase)	intergenic	22001757
rs2735839	19	51364623	intronic	KLK3andKLK2	Prostate cancer;Prostate-specific antigen levels	downstream	18264097, 23269536
rs2731672	5	176842474	intronic	GRK6	Platelet function and related traits;Metabolite levels;Activated partial thromboplastin time	intergenic	22916037, 21546496, 20303064
rs2730245	7	158724789	exonic	smarkee	Height	intronic	18391950
rs272889	5	131665378	UTR5	tawslerby	Metabolic traits	ncRNA intronic	21886157
rs2727405	11	13090942	intronic	choyskerby	Obesity-related traits	intergenic	23251661
rs2726807	4	183137398	intronic	ODZ3	Schizophrenia	intergenic	22885689
rs272594	8	81470120	promoter	RPSAP47	Neutrophil count	intergenic	21507922
rs2721051	13	41110884	UTR3	smerdoby	Central corneal thickness;Corneal structure	intergenic	20485516, 23291589
rs2718812	3	133399702	intronic	TFP1	Iron status biomarkers	intergenic	19084217
rs2716816	4	180383312	intronic	gleyspubu	Breast size	intergenic	22747683
rs2716734	2	39947721	ncRNA exonic	sotimo	Erectile dysfunction and prostate cancer treatment	intergenic	20932654
rs2712381	3	128338600	UTR3	soramo	Monocyte count	downstream	23314186

rs2711721	12	47372270	ncRNA intronic	rehumi	Prostate cancer (gene x gene interaction)	intergenic	22219177
rs270545	5	38051593	ncRNA intronic	norja	Major depressive disorder	intergenic	20125088
rs2705293	8	138918945	ncRNA intronic	nereya	Neuroticism	intergenic	18762592
rs2677744	15	91450441	exonic	MAN2A2	Attention deficit hyperactivity disorder	intronic	18839057
rs2675163	3	11075014	UTR5	SLC6A1	Response to Vitamin E supplementation	intronic	22437554
rs2669010	12	77009060	ncRNA intronic	flano	Systemic lupus erythematosus	intergenic	21408207
rs266849	19	51349090	intronic	slasee	Prostate-specific antigen levels	intergenic	21160077
rs2665838	17	61966465	intronic	GH1	Height	intergenic	20881960
rs2660753	3	87110674	ncRNA intronic	toseyu	Prostate cancer	intergenic	18264097
rs2657195	8	92559915	ncRNA intronic	seruya	Disc degeneration (lumbar)	intergenic	22993228
rs2652834	15	63396867	ncRNA intronic	skerpee	HDL cholesterol	intergenic	20686565
rs2652822	15	63422772	ncRNA exonic	jostaw	Metabolic traits	intronic	21886157
rs2650000	12	121388962	intronic	NCRNA00262	Metabolic traits;LDL cholesterol	intergenic	19060910, 19060906
rs2647264	4	106268594	intronic	PPA2	Immune response to anthrax vaccine;Immune response to anthrax vaccine	intergenic	22658931
rs261967	5	95850250	intronic	zerswabu	Body mass index	intergenic	22344219
rs260461	19	58770883	UTR5	ZNF544,yuwaru	Attention deficit hyperactivity disorder	intronic	18821565
rs259919	6	30025503	UTR3	ZNRD1	HIV-1 control	ncRNA intronic	20041166
rs2596542	6	31366595	intronic	noyjey	Hepatocellular carcinoma	intergenic	21499248
rs2593321	3	22122396	intronic	ZNF385D	HIV-1 control	intergenic	20205591
rs2586502	17	48289070	ncRNA	mihori,vygla	Response to	intergenic	23006423

			intronic		taxane treatment (docetaxel)		
rs2585417	20	52843799	intronic	PFDN4	Obesity-related traits	intergenic	23251661
rs258322	16	89755903	UTR5	CDK10	Melanoma;Black vs. red hair color	intronic	21983787, 19578364, 18483556
rs2575029	8	31174830	intronic	tyshor	Obesity-related traits	intergenic	23251661
rs2571391	6	29923838	intronic	ZNRD1	IgE levels;IgE levels	intergenic	22075330
rs255414	5	62817900	intronic	garger	Body mass index	intergenic	20935630
rs2545801	5	176841339	intronic	GRK6	Metabolite levels;Activated partial thromboplastin time	intergenic	22703881, 22286219
rs2540917	2	60608759	ncRNA intronic	skeykybu	Mean corpuscular volume	intergenic	19862010
rs2540226	2	39959060	intronic	glystuby	Personality dimensions	intergenic	18957941
rs2524054	6	31252396	intronic	HLA-BandHLA-C	CD4:CD8 lymphocyte ratio	intergenic	20045101
rs2524005	6	29899677	intronic	HLA ,ZNRD1	Bipolar disorder and schizophrenia	intergenic	22688191
rs2523946	6	29941943	intronic	ZNRD1	IgA nephropathy	upstream	22197929
rs2523809	6	29849619	ncRNA intronic	shersloyby	IgE levels;IgE levels	intergenic	22075330
rs2523608	6	31322559	UTR3	HLA-BandHLA-C	HIV-1 control	intronic	21051598, 20205591
rs2517713	6	29918099	intronic	ZNRD1	Nasopharyngeal carcinoma	intergenic	19664746
rs2516739	16	2097158	UTR3	NTHL1	Longevity	intronic	20834067
rs2507838	11	58472799	promoter	skawglubu	Body mass (lean)	intergenic	23108985
rs2501677	10	6946752	ncRNA intronic	sawlor	Fasting insulin-related traits (interaction with BMI)	intergenic	22581228
rs2501276	1	22373624	intronic	HSPC157andCDC42	Immune response to smallpox vaccine (IL-6)	intergenic	22610502
rs2484992	10	31512098	intronic	beefoy	Disc degeneration	intergenic	22993228

					(lumbar)		
rs2484990	10	31515132	intronic	beefoy	Disc degeneration (lumbar)	intergenic	22993228
rs2479409	1	55504650	intronic	PCSK9	LDL cholesterol;"Cholesterol, total"	upstream	20686565
rs2477686	1	2392648	intronic	PLCH2	Non-obstructive azoospermia	intergenic	22197933
rs2472297	15	75027880	intronic	CYP1A1	Coffee consumption	intergenic	21357676
rs2470893	15	75019449	intronic	CYP1A1	Coffee consumption;Caffeine consumption	intergenic	21876539, 21490707
rs2463822	11	62103420	promoter	ASRGL1	Chronic obstructive pulmonary disease-related biomarkers	intergenic	23144326
rs2461751	2	176289319	ncRNA intronic	wadar	Electrocardiographic conduction measures	intergenic	19389651
rs2459210	10	126144179	intronic	LHPP	Hemostatic factors and hematological phenotypes	intergenic	22443383
rs245914	7	29218159	intronic	CHN2,CPVL	Obesity-related traits	intergenic	23251661
rs2444240	11	120040442	intronic	LOC729173,TRIM29	Corneal curvature	intergenic	22969067
rs2423279	20	7812350	ncRNA exonic	SRSF10P2	Colorectal cancer	intergenic	23263487
rs2412980	22	30592069	intronic	slardoy	Dialysis-related mortality	intergenic	21546767
rs2411984	17	47445751	intronic	warey	Sex hormone-binding globulin levels	intergenic	22829776
rs240444	21	11002011	intronic	BAGE3andBAGE2andTPTE	Bipolar disorder and schizophrenia	intergenic	20889312
rs2400997	14	101727107	ncRNA intronic	teheri	Prostate cancer (gene x gene interaction)	intergenic	22219177
rs2398162	15	96830550	UTR3	lorswo	Hypertension	intergenic	17554300
rs2395528	10	80100861	intronic	LOC100132987	Conduct disorder (interaction)	intergenic	18846501

rs2395029	6	31431780	exonic	MICAandHCP5	Psoriasis;HIV-1 control;Drug-induced liver injury (flucloxacillin); AIDS progression	ncRNA exonic	21051598, 20041166, 19483685, 19115949, 18369459
rs2388896	10	8954224	ncRNA intronic	valor	Tetralogy of Fallot	intergenic	23297363
rs2383024	9	17487945	UTR3	CNTLN	Pulmonary function decline	intronic	22424883
rs2382817	2	219151218	exonic	TMBIM1	Inflammatory bowel disease	intronic	23128233
rs237743	20	47903019	UTR3	NCRNA00275andSNORD12CandSNORD12B	Height	ncRNA intronic	20881960
rs2370759	10	32634972	UTR3	EPC1	Sexual dysfunction (female)	intronic	22509378
rs2369304	14	96206070	intronic	stupar	Neutrophil count	intergenic	21507922
rs2361502	2	234698790	intronic	narstuby	Bilirubin levels	intergenic	21646302
rs2338104	12	109895168	UTR5	KCTD10	HDL cholesterol	intronic	19060906, 18193043
rs2338	6	1573613	ncRNA intronic	skeejor	Response to platinum-based agents	intergenic	22020760
rs2333163	4	176270885	intronic	faborbo	Obesity-related traits	intergenic	23251661
rs2326017	17	46720565	intronic	rulee	Cognitive performance	intergenic	19734545
rs2312147	2	58222928	intronic	VRK2	Schizophrenia	intergenic	19571808
rs2303745	19	17420289	intronic	ABHD8	Systemic lupus erythematosus	upstream	23273568
rs2303369	2	27715416	exonic	FNDC4	Menopause (age at onset)	intronic	22267201
rs2297644	10	99359739	UTR3	DHDPSLandPI4K2A	Metabolite levels	intronic	22286219
rs229541	22	37591318	intronic	C1QTNF6andIL2RB	Type 1 diabetes	intergenic	19430480, 18978792
rs2293941	13	28491198	splicing	flydoy(flydoy.aAug10:exon3:c.197-2G>G)	Fasting glucose-related traits (interaction with BMI)	intergenic	22581228
rs2293889	8	116599199	UTR3	TRPS1	HDL cholesterol	intronic	20686565
rs2293152	17	40481529	UTR5	STAT3	Multiple sclerosis	intronic	22190364
rs2292303	12	102513531	UTR5	NUP37	Height	upstream	19893584
rs2290159	3	12628920	UTR3	RAF1	Cholesterol,	intronic	20686565

					total		
rs2289700	15	79224683	UTR5	CTSH	Bipolar disorder	intronic	21771265
rs2286276	7	72987354	exonic	TBL2	Triglycerides	intronic	21909109
rs2285947	7	21584088	UTR3	DNAH11	Multiple cancers (lung cancer, gastric cancer, and squamous cell carcinoma)	intronic	23103227
rs2284746	1	17306675	exonic	jorplo	Pulmonary function;Height; Pulmonary function (interaction)	intronic	21946350, 20881960, 23284291
rs228437	6	134898456	ncRNA intronic	kusiya	Melanoma	intergenic	21983787
rs2282335	9	71865932	UTR5	TJP2	Renal sinus fat;Renal sinus fat	intronic	22044751
rs2281808	20	1610551	UTR3	SIRPG	Type 1 diabetes	intronic	19430480
rs2281680	14	24033070	UTR5;UTR3	JPH4andAP1G2;JPH4andAP1G2	Sudden cardiac arrest	intronic	21658281
rs2281388	6	33060118	intronic	HLA-DPB1	Graves' disease	intergenic	21841780
rs2280890	8	22404840	intronic	SORBS3	HIV-1 control	intergenic	20205591
rs2278702	15	80694922	intronic	hekama	Bipolar disorder	intergenic	18711365
rs2278170	11	4496846	exonic	OR52K3P	Amyotrophic lateral sclerosis	intergenic	22470424
rs2277862	20	34152782	exonic	FER1L4	Cholesterol, total	intergenic	20686565
rs2275606	6	146918950	ncRNA intronic	slyjer	Leprosy	intergenic	22019778
rs227458	6	165466351	ncRNA intronic	cheesnerbu	Visceral adipose tissue adjusted for BMI	intergenic	22589738
rs2271404	2	112003867	UTR3	klader	Atopic dermatitis	intergenic	23042114
rs2259816	12	121435587	exonic	HNF1A	C-reactive protein;Coronary heart disease	intronic	22939635, 19198612
rs225848	14	30594657	intronic	PRKD1	Sexual dysfunction (SSRI/SNRI-related)	intergenic	22445761
rs2255221	6	31431691	exonic	MICAandHCP5	HIV-1 control	ncRNA exonic	21051598
rs2252865	1	8422676	UTR3	RERE	Schizophrenia	intronic	21926974
rs2251301	8	11119037	ncRNA intronic	spyker	Response to antipsychotic therapy	intergenic	19875103

					(extrapyramidal side effects)		
rs2248462	6	31446796	promoter	nanure	Hodgkin's lymphoma	intergenic	22286212
rs2248359	20	52791518	promoter	CYP24A1	Multiple sclerosis	intergenic	21833088
rs2247056	6	31265490	ncRNA intronic	rosiya	Triglycerides	intergenic	20686565
rs2244067	1	187412042	promoter	storkeybo	Metabolite levels (5-HIAA)	intergenic	23319000
rs2242944	21	40465178	intronic	PSMG1	Ankylosing spondylitis	intergenic	20062062
rs224278	10	64580575	intronic	EGR2	Ewing sarcoma	intergenic	22327514
rs2242663	11	66335308	UTR5	CTSF	Bipolar disorder	intronic	19416921
rs2241423	15	68086838	ncRNA exonic	slazo	Body mass index	intronic	20935630
rs2240466	7	72856269	UTR3	BAZ1B	Triglycerides;Caffeine consumption	intronic	21490707, 19060911
rs2239815	22	29192670	UTR5;UTR3	XBP1;XBP1	Esophageal cancer (squamous cell)	intronic	22960999
rs2239557	14	74551970	exonic	LIN52	Common traits (Other)	intronic	20585627
rs2236653	11	126283785	UTR3	ST3GAL4	Liver enzyme levels (alkaline phosphatase)	intronic	22001757
rs2233152	19	41281016	intronic	MIA	Kawasaki disease	upstream	22446962
rs223116	14	23977010	intronic	NGDN	Resting heart rate	intergenic	20639392
rs2220321	8	127911596	ncRNA intronic	bleyvyby	Obesity-related traits	intergenic	23251661
rs2213169	11	5303063	intronic	HBE1,OR51B5andHBG2andHBG1	Mean corpuscular hemoglobin concentration;Hematology traits	intergenic	23263863
rs2205960	1	173191475	intronic	TNFSF4	Systemic lupus erythematosus	intergenic	19838193, 23273568
rs2199936	1	160864221	intronic	ITLN1	Urate levels;Lipoprotein-associated phospholipase A2 activity change in response to statin	intergenic	23118302, 21943158, 20884846

					therapy;Cardiovascular disease risk factors		
rs219553	2	21577743	intronic	flordor	Erectile dysfunction and prostate cancer treatment	intergenic	20932654
rs2193071	2	8036260	ncRNA exonic	sheedar	Obesity-related traits	intergenic	23251661
rs2185570	10	96751270	UTR3	plarnoyby	Dehydroepiandrosterone sulphate levels	intergenic	21533175
rs217181	16	72114002	intronic	TXNL4B	Metabolite levels	intergenic	22916037
rs2168889	4	75223711	intronic	skerflor	Metabolite levels	intergenic	22916037
rs2168303	18	58375934	ncRNA intronic	rimuri	Gout	intergenic	23263486
rs2165468	10	3516105	intronic	loylor	Bone mineral density	intergenic	17903296
rs216345	9	33799370	intronic	bloyplabu	Bipolar disorder	downstream	18711365
rs2159324	19	45695738	intronic	BLOC1S3andMARK4	Quantitative traits	intergenic	19197348
rs2158836	7	107580839	UTR5	LAMB1	Ulcerative colitis	intronic	19122664
rs2153299	10	28752106	ncRNA intronic	charklybu	Obesity-related traits	intergenic	23251661
rs2144134	20	13148152	promoter	teeto	Body mass index (interaction)	downstream	23192594
rs2138852	17	27703349	intronic	shylee	Mean platelet volume	intergenic	19820697, 19110211
rs2136093	1	90875888	ncRNA intronic	roseyo	Response to antidepressants	intergenic	20360315
rs2130017	11	89502376	promoter	daglee	Response to taxane treatment (docetaxel)	intergenic	23006423
rs2120991	12	54270228	intronic	skuno	Biliary atresia	intergenic	20460270
rs2119704	14	88487689	ncRNA intronic	gomaby	Multiple sclerosis	intergenic	21833088
rs2117032	12	21074122	intronic	SLCO1B3andLST-3TM12andSLCO1B1	Bilirubin levels	intergenic	19419973
rs2115763	11	112051169	UTR3	SDHD	Interleukin-18 levels	intronic	20150558
rs2114646	2	170624221	intronic	PHOSPHO2andK	Obesity-related	intergenic	23251661

				LHL23	traits		
rs2110001	7	150517022	intronic	TMEM176B	Height	intergenic	20881960
rs210937	6	135540380	intronic	rawjer	Obesity-related traits	downstream	23251661
rs2108225	7	107453103	ncRNA intronic	LOC100128307	Ulcerative colitis	intergenic	19915573
rs2104362	6	33825358	ncRNA intronic	borbloy	Alzheimer's disease (age of onset)	intergenic	22005931
rs210359	14	54167472	ncRNA intronic	siheru	Immune reponse to smallpox (secreted IFN-alpha)	intergenic	22610502
rs2102808	2	169117025	exonic	skawsterby	Parkinson's disease	intergenic	21292315
rs210142	6	33546837	UTR5	BAK1	Chronic lymphocytic leukemia	intronic	22700719
rs210138	6	33542538	UTR3	BAK1	Testicular germ cell tumor	intronic	19483681
rs210135	20	31277094	UTR3	BAK1P1	Hematological parameters	intergenic	19820697
rs2097677	7	22732839	ncRNA intronic	yuhiya	C-reactive protein	intergenic	21196492
rs2093210	14	60957279	intronic	C14orf39	Height	intergenic	20881960
rs2090409	9	108967088	ncRNA intronic	glerspar	Menarche (age at onset)	intergenic	21102462, 19448620
rs2087160	4	111334730	intronic	ENPEP	Metabolic traits	intergenic	21886157
rs2084898	11	120026748	intronic	TRIM29	Stroke (pediatric)	intergenic	22990015
rs2080401	2	171540823	intronic	modar	Coronary heart disease	intergenic	21347282
rs2079742	17	59465697	exonic	rekiri	Urate levels	intronic	23263486
rs2078267	11	64334114	UTR3	SLC22A11	Urate levels	intronic	20884846, 23263486
rs2075876	21	45709153	UTR5;UTR3	AIRE;AIRE	Rheumatoid arthritis	intronic	21505073
rs2075672	7	100240296	intronic	TFR2	Red blood cell traits	downstream	23222517
rs2074518	17	33324382	UTR5	LIG3	QT interval	intronic	19305408
rs2074488	6	31240431	intronic	HLA-BandHLA-C	Chronic obstructive pulmonary disease-related biomarkers	upstream	23144326
rs2074238	11	2484803	UTR3	KCNQ1	QT interval	intronic	19305408
rs2073233	20	12874585	ncRNA intronic	torvor	Brain structure	intergenic	20171287

rs2073080	22	44394402	ncRNA intronic	wopa	Nonalcoholic fatty liver disease	upstream	22719876
rs2072590	2	177042633	exonic	LOC401022	Ovarian cancer	ncRNA intronic	20852632
rs2070729	5	131819921	UTR3	IRF1	Platelet counts	intronic	22139419
rs2069084	1	234984988	ncRNA intronic	husiyo	HIV-1 control	intergenic	20041166
rs2062377	8	120007420	promoter	COLEC10	Bone mineral density (spine);Bone mineral density	intergenic	19801982, 22504420
rs2062305	13	43052880	ncRNA intronic	feeseyby	Crohn's disease	intergenic	21102463
rs2058710	2	207121782	intronic	nehura	Bipolar disorder and schizophrenia	intergenic	22688191
rs2058350	12	3913427	intronic	PARP11	Cognitive performance	intergenic	20125193
rs2057178	11	32364187	intronic	smawmaw	Tuberculosis	intergenic	22306650
rs204993	6	32155581	exonic	loyseeby	Asthma	intronic	21804548
rs2048485	18	42090667	intronic	rahima	Schizophrenia	intergenic	22885689
rs2047267	5	25087725	intronic	spywyby	Uric acid levels	intergenic	21294900
rs2040406	6	32603007	intronic	HLA-DQA1	Multiple sclerosis	intergenic	20453840
rs2034764	9	2742771	intronic	KIAA0020	Alzheimer's disease (age of onset)	intergenic	22005931
rs2033195	5	153509596	intronic	MFAP3andGALNT10	Body mass index	intergenic	21701570
rs2032794	5	86432617	ncRNA intronic	nawflee	Personality dimensions	intergenic	21173776
rs2032366	18	59266371	intronic	deesey	Obesity-related traits	intergenic	23251661
rs2022309	1	95052476	intronic	tiyaro	End-stage coagulation	intergenic	23381943
rs2014355	12	121175524	UTR5	ACADS	Metabolite levels	intronic	20037589
rs1999930	6	116387134	intronic	FRK	Age-related macular degeneration	intergenic	21665990
rs1990193	17	68828089	ncRNA intronic	forto	Obesity-related traits	intergenic	23251661
rs1981483	16	630665	exonic	NHLRC4andPIGQ	Disc degeneration (lumbar)	intronic	22993228
rs1978503	18	53664282	ncRNA intronic	persee	Breast cancer	intergenic	17903305

rs1976403	1	21766453	intronic	NBPF3	Liver enzyme levels (alkaline phosphatase)	upstream	22001757
rs1967689	1	208039471	UTR3	C1orf132	Age-related macular degeneration	intergenic	23326517
rs1967017	1	145723645	intronic	PDZK1	Urate levels	intergenic	20884846
rs1959947	14	41523462	intronic	blardey	Hemostatic factors and hematological phenotypes	intergenic	22443383
rs1953600	10	81911725	UTR3	ANXA11	Sarcoidosis	intergenic	22936702
rs1953126	9	123640500	intronic	PHF19	Celiac disease and Rheumatoid arthritis	upstream	21383967
rs1951082	14	27260043	ncRNA intronic	kodey	Attention deficit hyperactivity disorder and conduct disorder	intergenic	18951430
rs1949733	4	8503359	intronic	yotime	Response to antineoplastic agents	intergenic	21659360
rs1944582	18	45815417	intronic	ZBTB7C	Response to taxane treatment (docetaxel)	intergenic	23006423
rs1936800	6	127436064	ncRNA intronic	klubloyby,sowam e	Renal function-related traits (BUN)	intergenic	22797727
rs1930961	22	25875265	UTR3	namaru	Bipolar disorder (mood-incongruent)	intergenic	23092984
rs1927702	9	15986716	intronic	C9orf93	Body mass index	intergenic	19851299
rs1918172	2	156888500	ncRNA intronic	geedo	Attention deficit hyperactivity disorder	intergenic	18821565
rs1917445	11	103589094	ncRNA intronic	keruro	Neutrophil count	intergenic	21507922
rs1900004	10	70000881	ncRNA intronic	gawlor	Vertical cup-disc ratio;Optic disc parameters	intergenic	20548946
rs189798	8	8990577	promoter	smoyzarby	Myopia (pathological)	intergenic	23049088
rs1895320	5	159849586	UTR5	PTTG1	Insulin-related traits	intronic	22791750
rs1893767	11	123956466	ncRNA intronic	plomey	Obesity-related traits	intergenic	23251661
rs1893154	18	905125	UTR5;UT	ADCYAP1;gersa	Obesity-related	intronic	23251661

			R3	w	traits		
rs1888414	21	21777730	ncRNA intronic	romura	Hippocampal atrophy	intergenic	19668339
rs1884302	20	7106289	ncRNA intronic	jerto	Sagittal craniosynostosis	intergenic	23160099
rs1884136	20	10850464	ncRNA intronic	wasara	Information processing speed	intergenic	21130836
rs1883415	6	24491475	intronic	GPLD1	Liver enzyme levels (alkaline phosphatase)	intergenic	22001757
rs1883414	6	33086448	UTR3	HLA-DPB2	Nephropathy	ncRNA intronic	21399633
rs1879248	3	180551214	intronic	CCDC39	Schizophrenia	intergenic	21926974
rs1878047	19	51773802	ncRNA intronic	tukera	Body mass index	intergenic	19851299
rs1876040	2	19134748	promoter	klygeybu	Cognitive performance	intergenic	20125193
rs1875620	9	91540059	ncRNA intronic	kasuro	Response to statin therapy (LDL-C)	intergenic	22331829
rs1867504	3	133410661	intronic	TFP1	Iron status biomarkers	intergenic	19084217
rs1867485	16	86681031	ncRNA intronic	jyzy	Obesity-related traits	intergenic	23251661
rs1859962	17	69108753	intronic	lorrey	Prostate cancer	intergenic	21743057, 19767753, 18264097, 17603485
rs1853665	6	150298842	promoter	RAET1J	Radiation response	intergenic	20923822
rs1850744	4	9790712	intronic	SLC2A9	Economic and political preferences	intergenic	22566634
rs1845344	4	120962385	promoter	geegu	F-cell distribution	intergenic	21326311
rs1835740	8	98166913	ncRNA intronic	yuhare	Migraine	intergenic	20802479
rs1834481	11	112023827	UTR5	IL18	Interleukin-18 levels	intronic	20150558
rs1819333	6	167373547	intronic	sherfleeby	Inflammatory bowel disease	intergenic	23128233
rs1806864	9	90031669	ncRNA intronic	LOC100287242	Bipolar disorder and schizophrenia	intergenic	20889312
rs1800588	15	58723675	intronic	LIPC	HDL cholesterol	upstream	18193044
rs1795708	12	58750680	ncRNA exonic	LOC100127973	Obesity-related traits	intergenic	23251661
rs1789891	4	100250419	intronic	ADH1CandADH	Conduct	intergenic	22004471,

				1A	disorder (interaction);Alcohol dependence		18846501
rs17826255	17	29333516	promoter	plarsmu	Myopia (pathological)	intergenic	23049088
rs17824620	12	113100994	intronic	RPH3A	Platelet counts	intergenic	22139419
rs17818399	2	46826026	ncRNA exonic	zufa	Height	intronic	20966902
rs17817600	11	85677471	UTR3	borchoy	Alzheimer's disease	intronic	22832961
rs17798991	18	47121316	intronic	LIPG	Obesity-related traits	intergenic	23251661
rs17788937	12	77899510	ncRNA intronic	gluno	Myopia (pathological)	intergenic	23049088
rs17780304	17	30535856	UTR3	RHOT1	Obesity-related traits	intronic	23251661
rs17780086	17	30343282	intronic	LRRC37B	Height	intergenic	20881960
rs17773430	18	57963117	ncRNA intronic	spysho	Obesity and blood pressure	intergenic	22013104
rs17767419	16	79744548	ncRNA intronic	narar	Thyroid volume	intergenic	21565293
rs17695092	5	173337853	UTR3	CPEB4	Crohn's disease	intronic	23128233
rs17679567	16	76890126	ncRNA intronic	bodar	Response to TNF-alpha inhibitors in rheumatoid arthritis	intergenic	22569225
rs17666963	10	125207501	ncRNA intronic	zamu	Cardiac Troponin-T levels	intergenic	23247143
rs176095	6	32158319	intronic	RNF5	Atopic dermatitis	upstream;downstream	23042114
rs17608059	17	13910549	ncRNA intronic	moteru	Temperament	intergenic	22832960
rs17594362	13	42139245	ncRNA intronic	rorire	Multiple sclerosis	intergenic	21833088
rs17589290	4	112923237	intronic	torsweebu	Coronary heart disease	intergenic	21347282
rs17586674	5	2335581	promoter	fluplaby	Cognitive performance	intergenic	20125193
rs1757171	6	37487044	intronic	huwame	Cognitive performance	intergenic	19734545
rs17511627	13	26724328	intronic	RNF6	Alzheimer's disease	intergenic	22159054
rs17491951	2	2926013	ncRNA intronic	yoraro	White matter integrity (interaction)	intergenic	23218918
rs17468244	5	124848906	ncRNA	klugor	Obesity-related	intergenic	23251661

			intronic		traits		
rs17466684	8	27452847	ncRNA intronic	kluskobu	Panic disorder	intergenic	19165232
rs17465637	1	222823529	UTR3	MIA3	Myocardial infarction (early onset);Coronary heart disease	intronic	21378990, 19198609, 17634449
rs174601	11	61623140	UTR5	FEN1andFADS2	Liver enzyme levels (alkaline phosphatase)	intronic	22001757
rs17445836	16	86017663	intronic	gerzy	Multiple sclerosis	intergenic	19525953
rs17439299	11	15620586	ncRNA intronic	stawfaw	Obesity-related traits	intergenic	23251661
rs17431357	12	120880434	intronic	COX6A1andGATC	Insulin resistance/response	intergenic	21901158
rs17429217	12	117295333	UTR3	kune	Alzheimer's disease (age of onset)	intergenic	22005931
rs17419851	6	24749413	intronic	peefloyby	Neutrophil count	intergenic	21507922
rs17410015	1	101551926	ncRNA intronic	keyo	Response to antipsychotic treatment	intergenic	20195266
rs17398575	7	106409452	intronic	sokaw	Carotid intima media thickness	intergenic	21909108
rs17356664	19	45740771	intronic	BLOC1S3andMARK4	Platelet counts	intergenic	22139419
rs1735151	21	41123301	UTR5	IGSF5	Coronary heart disease	intronic	21347282
rs1734907	7	100315517	promoter	vorskaby	Inflammatory bowel disease	intergenic	23128233
rs1733724	10	54223977	intronic	skumo	Ventricular conduction;Electrocardiographic traits	intergenic	21076409, 20062063
rs17331332	4	106808107	intronic	INTS12	Pulmonary function (interaction)	intergenic	23284291
rs17321515	8	126486409	ncRNA intronic	kavoyby	Triglycerides	intergenic	18193044, 18193043
rs17291045	4	161506897	ncRNA intronic	bleeberbo	HIV-1 control	intergenic	20041166
rs17290922	16	57024317	intronic	NLRC5	Schizophrenia	intergenic	23212062
rs1728918	2	27635463	ncRNA intronic	choydo	Crohn's disease	intergenic	23128233
rs1727638	6	72139572	ncRNA intronic	lyjo	AB1-42	intergenic	20932310

rs17275498	2	231420401	intronic	swageebu	Cognitive performance	intergenic	20125193
rs1727307	12	123575742	UTR3	fleegarby	Platelet counts	intronic	21507922
rs17249754	12	90060586	intronic	ATP2B1	Blood pressure;Biomedical quantitative traits	intergenic	21909110, 21572416, 19396169
rs17197037	14	21725654	ncRNA exonic	stersly	Bipolar disorder	intronic	21738484
rs1719271	15	65183801	intronic	PLEKHO2andANKDD1A	Platelet counts	intergenic	22139419
rs17188434	2	157096776	ncRNA intronic	geedo	Menarche (age at onset)	intergenic	21102462
rs17159640	7	112086333	ncRNA exonic	gubler	Mortality among heart failure patients	intronic	20400778
rs17151904	7	25608409	ncRNA intronic	chyky	Carotid atherosclerosis in HIV infection	intergenic	20009918
rs17146964	11	65249145	ncRNA intronic	meeferby	Vertical cup-disc ratio	intergenic	20548946
rs17145738	7	72982874	ncRNA exonic	farflaby	Triglycerides;HDL cholesterol	downstream	20686565, 18193044, 18193043
rs17135859	5	112996654	intronic	byflar	F-cell distribution	intergenic	21326311
rs17129289	1	84232748	ncRNA intronic	plerber	Obesity-related traits	intergenic	23251661
rs17126232	8	17977650	intronic	plyskobu	Obesity	intergenic	21552555
rs1712517	10	105033015	intronic	kloyglerbu	Migraine	intergenic	22683712
rs17122021	11	118145686	intronic	CD3DandMPZL2	Pain	intergenic	19207018
rs17121944	12	59440232	ncRNA intronic	monure	Temperament (bipolar disorder)	intergenic	22365631
rs17115100	10	104591393	UTR3	C10orf26	Parkinson's disease	intronic	19915575
rs17114036	1	56962821	ncRNA exonic	snoyreybo	Coronary heart disease	intronic	21378990
rs17109512	10	100127551	ncRNA intronic	snarskaw	Aspartate aminotransferase;Aspartate aminotransferase	intergenic	21900944
rs17100498	5	143003605	ncRNA intronic	glawja	Response to antipsychotic treatment	intergenic	20195266
rs170934	3	28079085	intronic	mikeme	Multiple	intergenic	22190364

					sclerosis		
rs17086172	18	70227021	intronic	CBLN2	Airflow obstruction;Airflow obstruction	intergenic	22837378
rs17085106	18	69439073	ncRNA intronic	smorsar	Orofacial clefts	intergenic	19656524
rs17081935	4	57823476	intronic	slorspeeby	Height	intergenic	20881960
rs17079928	13	24654228	intronic	SPATA13andC1QTNF9	Orofacial clefts	intergenic	22419666
rs17079773	13	24598384	intronic	SPATA13andC1QTNF9	Inattentive symptoms	intergenic	18821565
rs17079247	13	85665879	ncRNA intronic	tarcha	Bipolar disorder (mania)	intergenic	23326512
rs17057678	6	130031493	intronic	C6orf191andARHGAP18	Antineutrophil cytoplasmic antibody-associated vasculitis;Antineutrophil cytoplasmic antibody-associated vasculitis	upstream	22808956
rs17056274	18	72787046	promoter	zerploy	Estradiol levels	intergenic	22675492
rs17054265	9	91260562	intronic	LOC286238	Obesity-related traits	intergenic	23251661
rs17050782	4	140423134	intronic	SETD7	Smoking behavior	intergenic	19247474
rs17049741	4	138582711	ncRNA intronic	spargar	Obesity-related traits	intergenic	23251661
rs17043947	2	22736987	ncRNA intronic	spyjobu	Self-rated health	intergenic	20707712
rs17036170	3	12330411	UTR5	PPARG	Drug-induced liver injury;Drug-induced liver injury	intronic	22968431
rs17031508	4	102560290	intronic	BANK1	Blood pressure	intergenic	22763476
rs17025426	1	110645327	ncRNA intronic	kasiyo	Visceral adipose tissue/subcutaneous adipose tissue ratio	intergenic	22589738
rs17023900	3	87134800	ncRNA intronic	toseyu	Prostate cancer	intergenic	22923026
rs1701704	12	56412487	intronic	IKZF4	Type 1 diabetes autoantibodies;Type 1 diabetes;Asthma	intergenic	21829393, 21804548, 20596022, 18198356

					;Alopecia areata		
rs17007695	4	142709723	UTR3	sweyplaby	Response to treatment for acute lymphoblastic leukemia	intergenic	19176441
rs16996151	4	89978800	intronic	FAM13A	Response to antipsychotic therapy (extrapyramidal side effects)	upstream	19875103
rs16970672	17	75944650	intronic	riheri	Alzheimer's disease	intergenic	22005930
rs16966460	15	38511983	ncRNA intronic	poree	Bipolar disorder	intergenic	18711365
rs16965962	13	105807023	intronic	charserby	Response to citalopram treatment	intergenic	22760553
rs16965039	16	57047299	intronic	NLRC5	Coronary heart disease	intergenic	21347282
rs16961543	16	59940188	ncRNA intronic	noyglo	Obesity-related traits	intergenic	23251661
rs16955379	16	81489373	ncRNA exonic	glyspu	Type 2 diabetes	intronic	22158537
rs16951095	18	7042911	UTR5	LAMA1	Non-small cell lung cancer	intronic	20876614
rs16948048	17	47440466	intronic	warey	Diastolic blood pressure	upstream	19430483
rs16940202	16	86014241	promoter	foyspa,gerzy	Ulcerative colitis	intergenic	21297633
rs16939046	8	76147954	ncRNA intronic	snokey	Information processing speed	intergenic	21130836
rs16937883	9	20098711	intronic	chaspar	Asthma (toluene diisocyanate-induced)	intergenic	19187332
rs16928297	11	2486120	UTR3	KCNQ1	QT interval	intronic	23166209
rs16921914	11	31210771	intronic	DCDC1	Bone mineral density (spine)	intergenic	19801982
rs16920624	10	20810525	ncRNA intronic	kahero	Response to antidepressants	intergenic	20360315
rs16912238	9	125573030	intronic	PDCL	Obesity-related traits	intergenic	23251661
rs16910421	11	12070665	intronic	suriro	Visceral fat	intergenic	22589738
rs16910061	9	97314741	ncRNA intronic	zortoby	Height	intergenic	19893584
rs16909318	8	82445224	exonic	gluvyby	Visceral fat	intergenic	22589738
rs16902094	8	128320346	ncRNA intronic	LOC727677	Prostate cancer	intergenic	19767754

rs1689800	1	182168885	ncRNA intronic	nasayo	HDL cholesterol	intergenic	20686565
rs16879765	7	37989095	UTR3	GPR141andTXNDC3andEPDR1	Dupuytren's disease	intronic	21732829
rs16869652	6	33851173	intronic	niyura	Schizophrenia	intergenic	22885689
rs16862782	3	187687890	ncRNA intronic	keyswerby	Myopia (pathological)	intergenic	23049088
rs16861531	2	14398052	intronic	snordar	Response to antidepressant treatment	intergenic	22041458
rs16858228	2	12212445	ncRNA intronic	smeydar	Obesity-related traits	intergenic	23251661
rs16854884	3	143778708	intronic	C3orf58	Economic and political preferences (feminism/equality)	intergenic	22566634
rs16851585	1	177568799	intronic	zawfobu	Age-related macular degeneration	intergenic	23326517
rs16850885	4	75170140	intronic	skerflor	Immune reponse to smallpox (secreted IL-1beta)	intergenic	22610502
rs16844716	1	199356516	intronic	susayo	Dialysis-related mortality	intergenic	21546767
rs16839962	2	156925286	ncRNA intronic	geedo	Response to statin therapy	intergenic	20339536
rs16839626	2	204089730	ncRNA exonic	jeyklu	Obesity-related traits	intergenic	23251661
rs16838131	4	6058497	UTR5	JAKMIP1	Response to angiotensin II receptor blocker therapy	intronic	22566498
rs16832015	1	160421916	ncRNA intronic	risayo	Cognitive performance	intergenic	19734545
rs16830359	1	43596384	intronic	FAM183A	Cardiac hypertrophy	intergenic	21348951
rs16827293	2	150509332	ncRNA intronic	romemo	Waist-to-hip circumference ratio (interaction)	intergenic	23192594
rs167769	12	57503775	UTR5	STAT6	Eosinophilic esophagitis (pediatric)	intronic	20208534
rs1668357	7	38004406	intronic	SFRP4	Myopia (pathological)	intergenic	23049088
rs164898	1	91569689	ncRNA	stymawbo	Metabolic	intergenic	20694148

			intronic		syndrome		
rs1629826	12	54909144	ncRNA exonic	sleejoyby	Obesity-related traits	intronic	23251661
rs1625895	17	7578115	UTR3	TP53	Sex hormone-binding globulin levels	intronic	22829776
rs161645	5	104069917	ncRNA intronic	dygey	Depression (quantitative trait)	intergenic	23290196
rs1605834	2	22576100	ncRNA intronic	spyjobu	Bipolar disorder and schizophrenia	intergenic	20889312
rs160441	8	90656988	intronic	smarzeeby	Tuberculosis	intergenic	20694014
rs1593	4	187195551	UTR3	F11	Activated partial thromboplastin time	intronic	22703881
rs1577917	6	86691940	ncRNA intronic	shersnawbu	Response to antipsychotic treatment	intergenic	20195266
rs1572438	6	858970	ncRNA exonic	roykleyby	Aging	intergenic	22773346
rs1570854	10	3418124	intronic	loylor	Economic and political preferences (environmentalism)	intergenic	22566634
rs1569476	1	169608917	intronic	SELP	Total ventricular volume	intergenic	21116278
rs1568889	11	28009463	intronic	skawmee	Bipolar disorder	intergenic	21254220
rs1566039	5	6821914	ncRNA intronic	tareyu	Sphingolipid levels	intergenic	22359512
rs1564892	12	104445742	ncRNA exonic	zojawby	Corneal structure	intergenic	23291589
rs1564282	4	852313	exonic	GAK	Parkinson's disease (familial)	intronic	18985386
rs1562430	8	128387852	ncRNA intronic	LOC727677	Breast cancer	intergenic	21263130, 20453838
rs1558744	12	68504592	ncRNA intronic	LOC341333	Ulcerative colitis	intergenic	20228799, 19122664
rs1551398	8	126540051	ncRNA intronic	kavoyby,ryla	Crohn's disease	intergenic	18587394
rs1538138	6	82794594	intronic	yuwame	Corneal structure	intergenic	23291589
rs1537377	9	22169700	ncRNA intronic	busorby	Endometriosis	intergenic	23104006
rs1536827	10	135306158	UTR3	fleyma	Visceral adipose tissue/subcutane	intergenic	22589738

					ous adipose tissue ratio		
rs1534422	2	12640741	ncRNA intronic	smeydar	Type 1 diabetes	intergenic	19430480
rs1532815	1	165180089	ncRNA exonic	lawbar	Response to acetaminophen (hepatotoxicity)	intronic	21177773
rs1532278	8	27466315	UTR3	CLU	Alzheimer's disease (late onset)	intronic	21460841
rs1529093	2	177909595	ncRNA intronic	koyoyo	Non-alcoholic fatty liver disease histology (other)	intergenic	20708005
rs1523288	3	165280209	intronic	skersweyby	Heart failure	intergenic	20445134
rs1520333	8	79401038	ncRNA intronic	slukor	Multiple sclerosis	intergenic	21833088
rs1520223	12	102726338	ncRNA intronic	rerblorbu	Height	intergenic	19893584
rs1514178	1	61205469	intronic	riyuro	Phospholipid levels (plasma)	intergenic	21829377
rs1498095	3	42093422	intronic	TRAK1	Visceral adipose tissue/subcutaneous adipose tissue ratio	intergenic	22589738
rs1495377	12	71577101	intronic	TSPAN8	Type 2 diabetes;Creutzfeldt-Jakob disease (variant)	intergenic	22137330, 17554300
rs1493682	4	121041286	ncRNA intronic	sinayu	HIV-1 viral setpoint	intergenic	22174851
rs1491818	11	29195137	ncRNA intronic	merfee	Amyotrophic lateral sclerosis (age of onset)	intergenic	22959728
rs1490075	3	176422652	ncRNA intronic	sikume	Response to amphetamines	intergenic	22952603
rs1488902	11	89345025	intronic	NOX4	Amyotrophic lateral sclerosis	intergenic	20801717
rs1476442	7	91272344	ncRNA intronic	yuhuya	Obesity-related traits	intergenic	23251661
rs1474476	14	22607502	intronic	TRAV37	Obesity-related traits	intergenic	23251661
rs1471633	1	145723739	intronic	PDZK1	Urate levels	intergenic	23263486
rs1471403	4	88775243	ncRNA intronic	ninayu	Bone mineral density (spine);Bone mineral density (hip)	intergenic	19801982

rs1464108	12	131022010	intronic	RIMBP2	Alzheimer's disease	intergenic	22005930
rs1463984	9	108427062	promoter	nutorby	Cognitive performance	intergenic	20125193
rs1463525	3	174961387	ncRNA exonic	bloykoybu	Systemic lupus erythematosus	intronic	21408207
rs1457451	2	65862378	ncRNA intronic	KRT18P33	Iron status biomarkers	intergenic	19084217
rs1456315	8	128103937	ncRNA intronic	bleyvyby	Prostate cancer	intergenic	23023329, 20676098
rs1455244	18	11494199	intronic	kihama	Schizophrenia	intergenic	23212062
rs1449984	2	23414651	ncRNA intronic	peedo	Depression (quantitative trait)	intergenic	20800221
rs1444418	10	64560470	ncRNA intronic	bolor	Atopic dermatitis	intergenic	23042114
rs1440581	4	89226422	intronic	HERC6	Metabolite levels	intergenic	22916037, 22286219
rs1440072	2	223936738	intronic	KCNE4	Waist circumference;Body mass index	intergenic	20966902
rs1432295	2	61066666	ncRNA intronic	FLJ16341	Hodgkin's lymphoma	intergenic	21037568
rs1427593	2	137555224	intronic	THSD7B	Pancreatic cancer	intergenic	20686608
rs1425609	3	162681995	ncRNA intronic	zyskawby	Aging (time to death)	intergenic	21782286
rs1420956	18	25167945	intronic	wahima	Obesity-related traits	intergenic	23251661
rs1420101	2	102957716	exonic	IL1RL1andIL18R1	Eosinophil counts	intronic	19198610
rs1417437	1	70154441	intronic	LRRC7	Orofacial clefts	intergenic	22419666
rs1417352	6	106899226	UTR3	charjey	Select biomarker traits	intergenic	17903293
rs1417210	10	73135919	ncRNA intronic	doynoby	Vitiligo	intergenic	22951725
rs141215807	9	19213634	intronic	PLIN2	Metabolite levels (HVA)	intergenic	23319000
rs1411916	9	80940576	UTR3	speeplorbu	Visceral fat	intronic	22589738
rs1408272	6	25842951	intronic	HIST1H2APS2andSLC17A3	Mean corpuscular hemoglobin;Iron status biomarkers;Hematology traits;Red blood cell traits	intergenic	21149283, 19862010, 19853236, 23222517
rs1406961	20	61895920	intronic	NKAIN4	Cardiovascular	intergenic	21779381

					disease risk factors		
rs140522	22	50971266	UTR5	ODF3BandTYMPandSCO2	Multiple sclerosis;Red blood cell traits	upstream	21833088, 23222517
rs1404697	7	108544461	ncRNA exonic	hanare	Smoking behavior	intergenic	22006218
rs1402837	2	169757354	intronic	SPC25	Glycated hemoglobin levels	upstream	19096518
rs1402279	12	77729385	ncRNA intronic	gluno	Smoking behavior	intergenic	19247474
rs1398217	18	44752238	intronic	SKOR2	Menarche (age at onset)	intergenic	21102462
rs1388551	3	22292571	intronic	ZNF385D	Waist-to-hip circumference ratio (interaction)	intergenic	23192594
rs1387153	11	92673828	promoter	fymaw	Type 2 diabetes;Metabolic syndrome (bivariate traits);Glycated hemoglobin levels;Fasting plasma glucose	intergenic	21386085, 20858683, 20581827, 19060909
rs1386330	11	87819427	ncRNA intronic	vermer	Multiple sclerosis (age of onset)	intergenic	19010793
rs13831	20	57475191	UTR3	GNAS	Event-related brain oscillations	intronic	21184583
rs1371867	8	101330209	intronic	RNF19A	Atrioventricular conduction	intergenic	21041692
rs1371737	7	9189541	intronic	snoyblo	Obesity-related traits	intergenic	23251661
rs1368578	12	76476956	ncRNA exonic	pufawby	Obesity-related traits	intronic	23251661
rs1364705	8	120224806	ncRNA exonic	sporbla	Hippocampal atrophy	intronic	19668339
rs1361108	6	126767600	intronic	CENPW	Menarche (age at onset);Height	intergenic	21998595, 21102462
rs1360517	9	13007129	ncRNA intronic	TDPX2	AIDS	intergenic	19754311
rs1351394	12	66351826	UTR3	HMGA2	Height	intronic	20881960
rs1350666	4	75224590	intronic	skerflor	Attention deficit hyperactivity disorder	intergenic	18821565

rs1350172	10	63348767	intronic	reesterby	Response to taxane treatment (placlitaxel)	intergenic	23006423
rs134882	22	42670965	intronic	LOC388906	Bipolar disorder and schizophrenia	downstream	20889312
rs1344694	2	216893637	intronic	PECRandMREG	Alcohol dependence	intergenic	22004471
rs13438712	7	105542580	intronic	CDHR3	Obesity-related traits	intergenic	23251661
rs13428812	2	25492467	exonic	blarjarbu	Crohn's disease	intronic	21102463
rs1342371	6	44739949	ncRNA intronic	farglawby	Obesity-related traits	intergenic	23251661
rs1341239	6	22304204	intronic	flarblor	Paget's disease	intergenic	21623375
rs13409348	2	79539988	intronic	CTNNA2	Bipolar disorder	intergenic	19416921
rs13403276	2	219081297	ncRNA exonic	lawdee	Obesity-related traits	upstream	23251661
rs13401620	2	120513133	intronic	zeystuby	Breast size	intergenic	22747683
rs13398206	2	199169096	ncRNA intronic	sweedee	Prostate cancer (gene x gene interaction)	intergenic	22219177
rs13390159	2	237156549	UTR3	ASB18	Response to statin therapy	intronic	20339536
rs13381277	18	74318610	intronic	LOC284276	Dental caries	intergenic	23064961
rs13373941	1	239644171	intronic	CHRM3	Obesity-related traits	intergenic	23251661
rs13358260	5	78845711	ncRNA intronic	jersterbu	Adiponectin levels	intergenic	21700879
rs13330107	16	76878862	ncRNA intronic	bodar	Inattentive symptoms	intergenic	18821565
rs13314993	3	33015469	intronic	shufubo	Celiac disease	intergenic	20190752
rs1329568	9	37037976	UTR3	jeeteebo	Response to tocilizumab in rheumatoid arthritis	intergenic	22491018
rs1329424	1	196646176	UTR5	CFH	Age-related macular degeneration	intronic	20385819, 23326517
rs1329189	10	129998372	ncRNA intronic	plerpaby	Orofacial clefts	intergenic	22419666
rs13281615	8	128355618	ncRNA intronic	LOC727677	Breast cancer	intergenic	17529967
rs13273073	8	23584226	ncRNA intronic	toyvawbu	Treatment response for severe sepsis;Treatment response for severe sepsis	intergenic	22310353

rs1327235	20	10969030	ncRNA intronic	wasara	Systolic blood pressure;Diastolic blood pressure;Blood pressure	intergenic	21909115, 21909110
rs1326986	10	19929513	intronic	C10orf112	Ankylosing spondylitis	intergenic	20062062
rs13254738	8	128104343	ncRNA intronic	bleyvyby	Prostate cancer	intergenic	22923026
rs13252298	8	128095156	ncRNA intronic	bleyvyby	Prostate cancer	intergenic	21743057
rs1324015	13	43727849	ncRNA intronic	pazo	Cognitive performance	intergenic	19734545
rs1323292	1	192541021	ncRNA intronic	risimo	Multiple sclerosis	intergenic	21833088
rs13228694	7	99940307	intronic	PILRB	Obesity-related traits	intergenic	23251661
rs13210693	6	109598964	intronic	FLJ37396	Ankylosing spondylitis	intergenic	22138694
rs1320976	1	169073346	promoter	swawler	QT interval	intergenic	23166209
rs13208776	6	168941624	exonic	ferflarby	Vitiligo	intronic	19890347
rs1320333	2	679179	intronic	snawdor	Obesity-related traits	intergenic	23251661
rs13195786	6	10163968	ncRNA intronic	blufloybu	Calcium levels	intergenic	20705733
rs13194984	6	26500563	exonic	BTN1A1	Iron status biomarkers	upstream	19084217
rs13191343	6	31241109	intronic	HLA-BandHLA-C	Psoriatic arthritis	intergenic	20953186
rs1317209	1	20140036	promoter	choybo	Ulcerative colitis	downstream	20228799
rs1317082	3	169497585	UTR5	toremo	Telomere length	intronic	23001564
rs13137105	4	76416387	UTR3	RCHY1	Response to tocilizumab in rheumatoid arthritis	intronic	22491018
rs13129697	4	9926967	UTR3	beygo	Uric acid levels;Urate levels;Biochemical measures	intronic	22229870, 20884846, 19260141
rs13098911	3	46235201	intronic	CCR3	Celiac disease	intergenic	20190752
rs13068298	3	143772247	intronic	C3orf58	Economic and political preferences (feminism/equality)	intergenic	22566634
rs13053817	22	29847722	intronic	RFPL1S	Carotid atherosclerosis	intergenic	20009918

					in HIV infection		
rs1305088	13	29278450	UTR5	SLC46A3	Non-alcoholic fatty liver disease histology (other)	intronic	20708005
rs13038095	20	46425576	ncRNA intronic	vervey	Atrial fibrillation	intergenic	20173747
rs13026414	2	57934055	ncRNA intronic	klardaw,klodaw	Epilepsy (generalized)	intergenic	22949513
rs1302019	6	147971574	intronic	SAMD5	Myopia (pathological)	intergenic	23049088
rs13017599	2	61164331	UTR3	PUS10andRPS12P3	Rheumatoid arthritis;Psoriatic arthritis	intergenic	22170493, 19503088
rs13015714	2	102971865	intronic	IL1RL1andIL18R1	Celiac disease;Atopic dermatitis	intergenic	23042114, 18311140
rs13010713	2	181996045	intronic	mamemo	Celiac disease	intergenic	20190752
rs1299548	7	7302293	ncRNA intronic	storka	Visceral adipose tissue adjusted for BMI	intergenic	22589738
rs12988934	2	182323665	UTR3	ITGA4	White blood cell types	intronic	21738478
rs12980275	19	39731783	intronic	NCCRP1	Lipid levels in hepatitis C treatment	intergenic	22497812
rs1296028	8	11698747	intronic	FDFT1	Parkinson's disease	intergenic	22451204
rs12949531	17	13733806	ncRNA intronic	moteru	Systemic lupus erythematosus	intergenic	19165918
rs1294421	6	6743149	intronic	senore	Waist-hip ratio	intergenic	20935629
rs12938916	17	55866287	ncRNA intronic	stoyda	Bipolar disorder	intergenic	21254220
rs12928822	16	11403893	intronic	C16orf75	Celiac disease	intergenic	20190752
rs12907914	15	39315358	ncRNA intronic	rureru	Cardiac hypertrophy	intergenic	21348951
rs12901682	15	78833223	UTR5	PSMA4	Response to tocilizumab in rheumatoid arthritis	intronic	22491018
rs12793173	11	34834204	ncRNA intronic	zyflorbu	Cystic fibrosis severity	intergenic	21602797
rs12777823	10	96405502	intronic	CYP2C18andCYP2C19	Response to clopidogrel therapy	intergenic	19706858
rs12772794	10	20674653	ncRNA intronic	kahero	Neutrophil count	intergenic	21507922
rs12772169	10	96405329	intronic	CYP2C18andCY	Acenocoumarol	intergenic	19578179

				P2C19	maintenance dosage		
rs12753569	1	76484014	ncRNA intronic	geybu	Personality dimensions	intergenic	20691247
rs12742923	1	83489844	ncRNA intronic	stoybaw	HIV-associated dementia;HIV-associated dementia	intergenic	22628157
rs1273516	19	15677710	intronic	weejaw	Response to tocilizumab in rheumatoid arthritis	intergenic	22491018
rs12731740	1	208024820	intronic	C1orf132	Biomedical quantitative traits	intergenic	19396169
rs12726330	1	155108167	exonic	RAG1AP1	Parkinson's disease	upstream;downstream	22451204
rs12714207	2	88315793	promoter	choperbo	Bilirubin levels	intergenic	19414484
rs12696304	3	169481271	promoter	bloyjerbu	Telomere length	intergenic	21573004, 20139977
rs12679254	8	74274191	intronic	geyteybo	Inattentive symptoms	intergenic	18821565
rs12677663	8	74007347	intronic	C8orf84	Crohn's disease	intergenic	22412388
rs12669076	7	96415055	ncRNA intronic	vokar	Immunoglobulin A;Immunoglobulin A	intergenic	20694011
rs1265879	14	50877983	intronic	CDKL1	Cognitive performance	intergenic	20125193
rs12657996	5	158904313	ncRNA intronic	smarjoy	Response to taxane treatment (placlitaxel)	intergenic	23006423
rs12653946	5	1895829	intronic	tojy	Prostate cancer	intergenic	22923026, 20676098
rs12646107	4	174515682	ncRNA exonic	swobeybo	Response to amphetamines	intergenic	22952603
rs12644436	4	88805208	ncRNA intronic	ninayu	HIV-1 viral setpoint	intergenic	22174851
rs12636651	3	46282391	intronic	CCR3	Monocyte chemoattractant protein-1;Obesity-related traits;Monocyte chemoattractant protein-1	intergenic	23017229, 23251661
rs12634229	3	112376308	intronic	CCDC80	Atopic dermatitis	intergenic	23042114
rs12618573	2	240843638	UTR3	NDUFA10	Response to	intergenic	22952603

					amphetamines		
rs12604483	18	53855525	UTR5	rihima	HIV-1 susceptibility	intergenic	21160409
rs12602978	17	25567080	ncRNA intronic	fluwee	Dental caries	intergenic	23064961
rs1256531	14	65747759	ncRNA intronic	mohoru	Conduct disorder (symptom count)	intergenic	20585324
rs12529514	6	14096658	ncRNA intronic	mewame	Rheumatoid arthritis	intergenic	22446963
rs12526186	6	30736151	ncRNA intronic	lojaw	Response to antipsychotic treatment	intergenic	19721433
rs12524865	6	134196674	ncRNA intronic	pajer	Coronary heart disease	intergenic	22751097
rs12518099	5	89546109	intronic	skawja	Type 2 diabetes and other traits	intergenic	19734900
rs1250307	10	31495974	intronic	beefoy	Disc degeneration (lumbar)	intergenic	22993228
rs12500426	4	95514609	UTR3	snawfloy	Prostate cancer	intronic	19767753
rs1247318	6	161333937	ncRNA intronic	gloyjo	Aging	intergenic	22773346
rs12472274	2	239095422	UTR5	ILKAP	Phospholipid levels (plasma)	intronic	22359512
rs12447804	16	58075282	exonic	veyseybo	Pulmonary function;Pulmonary function (interaction)	intronic	21946350, 23284291
rs12434047	14	53837485	ncRNA intronic	horumi	Economic and political preferences (fairness)	intergenic	22566634
rs12431733	14	54290830	ncRNA intronic	siheru	Parkinson's disease	intergenic	19915575
rs12431307	13	80644618	intronic	semimi	Obesity-related traits	intergenic	23251661
rs12423247	12	96858362	intronic	kanar	Phospholipid levels (plasma)	intergenic	22359512
rs1240773	11	116520527	ncRNA intronic	keefaw	HIV-1 susceptibility	intergenic	21160409
rs12393627	X	2885723	intronic	ARSE	Height	intergenic	22021425, 21998595
rs12388359	X	10231691	ncRNA intronic	plymaw	Alcohol dependence	intergenic	19581569
rs12356193	10	61413353	exonic	SLC16A9	Uric acid levels	intronic	19503597
rs12328675	2	165540800	UTR3	COBLL1	HDL cholesterol	downstrea	20686565

						m	
rs12321565	12	68532077	ncRNA intronic	LOC341333	Bipolar disorder and schizophrenia	intergenic	20889312
rs12295638	11	26605331	ncRNA exonic	slarmar	Obesity (extreme)	intronic	19553259
rs12274302	11	90586900	intronic	boymaw	HIV-1 control	intergenic	20041166
rs12269901	11	116973929	ncRNA intronic	wyna	Coronary heart disease	intergenic	21347282
rs12261589	10	79540835	promoter	foklabu,wawnawby	Obesity-related traits	intergenic	23251661
rs1223271	20	13296912	intronic	TASP1	Parkinson's disease	intergenic	19915575
rs12216125	6	25997458	ncRNA intronic	smujar,vysnerby	Iron status biomarkers	intergenic	19084217
rs12206204	6	26116982	intronic	HIST1H2BC	Bilirubin levels	intergenic	19414484
rs12200560	6	97080198	ncRNA intronic	kiyora	Coronary heart disease	intergenic	22319020
rs12198063	6	77290799	intronic	reypoyby	Capecitabine sensitivity	intergenic	22864933
rs12191877	6	31252925	intronic	HLA-BandHLA-C	Psoriasis	intergenic	20953188, 19169254
rs12188300	5	158829527	ncRNA exonic	smargey	Psoriatic arthritis	intergenic	20953186
rs12175489	6	31377587	UTR5	MICAandHCP5	Visceral adipose tissue adjusted for BMI	intronic	22589738
rs12159200	22	41042091	intronic	MKL1	Treatment response for severe sepsis;Treatment response for severe sepsis	intergenic	22310353
rs12155172	7	20994491	ncRNA intronic	jawja	Prostate cancer	intergenic	19767753
rs12150660	17	7521915	intronic	SHBG	Testosterone levels;Sex hormone-binding globulin levels	intergenic	22829776, 21998597
rs12148488	15	75382542	intronic	PPCDC	Caffeine consumption	intergenic	21490707
rs12145922	1	89146234	ncRNA intronic	borbar	Liver enzyme levels (gamma-glutamyl transferase)	intergenic	22001757
rs12143943	1	204572071	UTR3	MDM4	Cognitive performance	intergenic	19734545

rs12130212	1	209727257	ncRNA intronic	kleybee	Obesity (extreme)	intergenic	21935397
rs12129861	1	145725689	intronic	PDZK1	Uric acid levels	intergenic	19503597
rs1211375	16	240280	UTR5	LUC7L	Mean corpuscular volume;Mean corpuscular hemoglobin;Hematology traits	intronic	23263863
rs12110693	6	122158270	ncRNA intronic	skoytoybo	Biomedical quantitative traits	intergenic	19396169
rs12109285	5	21749348	ncRNA intronic	susleebu	Response to tocilizumab in rheumatoid arthritis	intergenic	22491018
rs1208285	6	134158800	exonic	MGC34034	Infantile hypertrophic pyloric stenosis	ncRNA exonic	22306654
rs12079716	1	114444594	UTR5	AP4B1	Obesity-related traits	intronic	23251661
rs12073504	1	2452979	exonic	PANK4	Obesity-related traits	intronic	23251661
rs12067906	1	192431998	intronic	shojoybu	Blood pressure	intergenic	22763476
rs12049330	1	110031188	exonic	ATXN7L2	Major depressive disorder	intronic	20125088
rs12046278	1	10799577	ncRNA exonic	weyply	Systolic blood pressure	intronic	19430479
rs12035082	1	172898377	ncRNA intronic	darobo,ferplo	Crohn's disease	intergenic	17554261
rs12032672	1	88625636	ncRNA intronic	beybar,mosimo	Periodontal microbiota	intergenic	22699663
rs12029080	1	95053353	intronic	tiyaro	D-dimer levels	intergenic	21502573
rs11995824	8	120012700	intronic	COLEC10	Bone mineral density (hip)	intergenic	19801982
rs11986414	8	1746950	exonic	verlu	Gaucher disease severity	intergenic	22388998
rs1198588	1	98552832	ncRNA intronic	meseyo	Schizophrenia	intergenic	22688191
rs11984041	7	19031935	UTR5	misimu	Stroke	intronic	22306652
rs11978267	7	50466304	exonic	pyblorby	Acute lymphoblastic leukemia (childhood)	intronic	19684603
rs11959928	5	39397132	exonic	rorplerby	Chronic kidney disease	intronic	20383146
rs11937061	4	78136933	intronic	CCNG2	Response to	intergenic	22491018

					tocilizumab in rheumatoid arthritis		
rs11931074	4	90639515	ncRNA intronic	saso	Parkinson's disease	intergenic	19915576
rs11915082	3	195809139	UTR5	TFRC	Mean corpuscular hemoglobin	upstream	19862010
rs1190739	X	136055295	intronic	fleeny	Coronary heart disease	intergenic	21347282
rs11900673	2	62452661	UTR3	B3GNT2	Rheumatoid arthritis	downstream	22446963
rs11889862	2	150697148	ncRNA intronic	romemo	Menopause (age at onset)	intergenic	19448619
rs11870477	17	67802352	ncRNA intronic	beeto	Response to TNF-alpha inhibitors in rheumatoid arthritis	intergenic	22569225
rs11865038	16	31095171	UTR3	VKORC1andPRSS53	Parkinson's disease	intronic	22451204
rs11858836	15	78783277	exonic	IREB2	Chronic obstructive pulmonary disease	intronic	22080838
rs11857380	15	58712203	intronic	LIPC	Obesity-related traits	intergenic	23251661
rs11847697	14	30515112	intronic	PRKD1	Body mass index	intergenic	20935630
rs11845134	14	22782377	intronic	TRAV37	Obesity-related traits	intergenic	23251661
rs11842874	13	113694509	exonic	goyzarby	Osteoarthritis	intronic	21871595
rs11823543	11	116649135	UTR3	ZNF259	Triglycerides-Blood Pressure (TG-BP)	downstream	21386085
rs11809789	1	82074852	ncRNA intronic	blarboy	Bilirubin levels	intergenic	22085899
rs11790994	9	98429266	intronic	blarley	Inattentive symptoms	intergenic	18821565
rs1178979	7	72856430	UTR3	BAZ1B	Triglycerides	intronic	20864672
rs1178977	7	72857049	UTR5	BAZ1B	Urate levels	intronic	23263486
rs11786458	8	40252701	ncRNA intronic	blawkor	Inattentive symptoms	intergenic	18821565
rs11782819	8	10334781	exonic	rozawby	Alzheimer's disease	intergenic	20452100
rs11781622	8	24378741	intronic	ADAM7	White matter integrity	intergenic	23218918
rs11773845	7	116191301	exonic	voyju	PR interval	intronic	23139255

rs11773103	7	86821980	UTR5	DMTF1	Bipolar disorder and major depressive disorder (combined)	intronic	20351715
rs117616209	17	7142851	intronic	PHF23	Metabolite levels	upstream;downstream	22286219
rs11761231	7	131370039	ncRNA intronic	suhuya	Rheumatoid arthritis	intergenic	17554300
rs11752626	6	118788652	ncRNA exonic	swawgloyby	QT interval	intronic	23166209
rs11747270	5	150258867	intronic	slowuby	Crohn's disease	intergenic	18587394
rs11745587	5	131796922	UTR3	C5orf56andRAD50	Asthma	ncRNA intronic	22561531
rs11730243	4	106463713	ncRNA intronic	tenayu	Economic and political preferences (time)	intergenic	22566634
rs11724635	4	15737101	intronic	FAM200BandBST1	Parkinson's disease	intergenic	21292315
rs11719664	3	21955198	intronic	ZNF385D	Attention deficit hyperactivity disorder	intergenic	18821565
rs11711441	3	182821275	intronic	MCCC1	Parkinson's disease	intergenic	21292315
rs11710433	3	5705052	intronic	mydarbo	Bipolar disorder (mood-incongruent)	intergenic	23092984
rs11710077	3	38657899	exonic	cheefarbo	Ventricular conduction	intronic	21076409
rs11696845	20	43371320	intronic	LOC79015	Obesity-related traits	intergenic	23251661
rs1168987	14	36397680	ncRNA intronic	merloyby	Response to antineoplastic agents	intergenic	21659360
rs11686135	2	4318976	intronic	memiyo	Pulmonary function decline	intergenic	22424883
rs11676922	2	100806940	intronic	AFF3	Rheumatoid arthritis	intergenic	20453842
rs11674248	2	24154192	ncRNA exonic	keejarbu	Obesity-related traits	intergenic	23251661
rs11662721	18	19261413	ncRNA exonic	jusaw	Phospholipid levels (plasma)	intronic	22359512
rs11654749	17	69125606	intronic	lorrey	Pulmonary function (interaction)	intergenic	23284291
rs11648796	16	792190	promoter	NARFL	Height	intergenic	20881960
rs11646411	16	82746937	exonic	taseri	Attention deficit	intronic	18839057

					hyperactivity disorder		
rs11641231	16	86381184	ncRNA intronic	snawrar	Response to statin therapy	intergenic	20339536
rs11637235	15	48633153	UTR3	DUT	Protein quantitative trait loci	intronic	18464913
rs11636768	15	87695511	ncRNA intronic	gerdarby	Migraine	intergenic	21448238
rs11630290	15	63915786	UTR5	HERC1	Iris characteristics	intronic	21835309
rs11628318	14	103040087	ncRNA intronic	storswee	Platelet counts	intergenic	22139419
rs11626056	14	52233276	ncRNA exonic	leebyby	Hippocampal atrophy	intergenic	19668339
rs11624704	14	78786077	intronic	NRXN3	Obesity	intergenic	21552555
rs11623869	14	103883633	UTR5	MARK3	Bone mineral density	intronic	22504420
rs11618202	13	31113379	exonic	fawchy	MRI atrophy measures	intergenic	21116278
rs11616188	12	6502742	promoter	sneyzeeby	Ankylosing spondylitis	intergenic	21743469
rs11615916	12	62610860	intronic	FAM19A2	Pulmonary function decline	intergenic	22424883
rs11615274	12	73601132	ncRNA intronic	blushy	Body mass index and cholesterol (psychopharmacological treatment)	intergenic	22417934
rs1161463	13	29431338	intronic	MTUS2	Attention deficit hyperactivity disorder symptoms (interaction)	intergenic	18846501
rs11613352	12	57792580	intronic	R3HDM2andSTAC3	Triglycerides;HDL cholesterol	intergenic	20686565
rs11612508	12	12657513	exonic	spoyfoyby	Inflammatory bowel disease	intronic	23128233
rs11610206	12	47639526	intronic	doner	Alzheimer's disease	intergenic	19118814
rs11602954	11	202856	UTR3	BET1L	Mean platelet volume;Bone mineral density	downstream	19820697, 22504420
rs11597390	10	101861435	ncRNA intronic	tehoro	Liver enzyme levels	intergenic	18940312
rs11593576	10	81015896	exonic	snawnawby	Vitiligo	intronic	20526339
rs11590090	1	113313563	intronic	lorleybo	Hyperactive-	intergenic	18821565

					impulsive symptoms		
rs1158867	2	128177377	UTR5	PROC	Protein C levels	intronic	20802025
rs11574637	16	31368874	exonic	ITGAX	Systemic lupus erythematosus	intronic	18204098
rs11574514	16	67971380	UTR5	PSMB10andCTRL	Crohn's disease	upstream	22412388
rs11564258	12	40792300	intronic	snornaw	Crohn's disease;Inflammatory bowel disease	intergenic	21102463, 23128233
rs1154155	14	23002684	intronic	TRAV37	Narcolepsy	intergenic	20711174, 19412176
rs1148186	10	28617635	ncRNA intronic	pyloy	Pulmonary function decline	intergenic	22424883
rs1137	2	74939176	UTR3	sydor	Myopia (pathological)	intergenic	22685421
rs112553552	3	101411978	UTR5	rowamo	Response to amphetamines	intergenic	22952603
rs11249215	1	25297184	UTR5	smakler	Ankylosing spondylitis	intergenic	21743469
rs11245052	10	128267640	intronic	C10orf90	Metabolite levels (MHPG)	intergenic	23319000
rs1124480	3	13857969	ncRNA exonic	zoderbo	Chronic obstructive pulmonary disease-related biomarkers	intergenic	23144326
rs11243437	9	134450073	UTR3	bawlo	Response to amphetamines	intergenic	22952603
rs11242704	6	1535998	ncRNA intronic	skeejor	Response to hepatitis C treatment	intergenic	22095909
rs11239930	1	146560564	intronic	NBPF13P	AIDS progression	intergenic	21502085
rs1122608	19	11163601	exonic	chyflor	Myocardial infarction (early onset);Coronary heart disease	intronic	21378990, 19198609
rs11223996	11	134626887	intronic	setimi	Myopia (pathological)	intergenic	23049088
rs11206510	1	55496039	intronic	PCSK9	Myocardial infarction (early onset);LDL cholesterol;Coronary heart disease	intergenic	21378990, 20864672, 19198609, 19060906, 18193043
rs11191548	10	104846178	UTR3	NT5C2	Systolic blood	intergenic	21909115,

					pressure;Blood pressure		21909110, 21572416, 19430483
rs11190140	10	101291593	promoter	terpeeby	Ulcerative colitis;Crohn's disease	intergenic	20228799, 18587394
rs11175593	12	40601940	ncRNA intronic	royshy,snunaw	Crohn's disease	intergenic	18587394
rs1117324	2	21840093	ncRNA intronic	floydor	Response to antipsychotic treatment	intergenic	20195266
rs11170631	12	54041192	intronic	ATP5G2andRPL31P51	Height	intergenic	20189936
rs11169552	12	51155663	ncRNA intronic	keechar	Colorectal cancer	intergenic	20972440
rs11168351	12	48403765	intronic	COL2A1	Bipolar disorder and schizophrenia	intergenic	20889312
rs111649495	5	118356415	ncRNA intronic	voyja	Response to amphetamines	intergenic	22952603
rs11163372	1	82247248	intronic	LPHN2	Temperament	intergenic	22832960
rs11155133	6	141169825	ncRNA intronic	blujer	Acute lymphoblastic leukemia (childhood)	intergenic	19684603
rs11139399	9	84372741	ncRNA intronic	plorlaw	Hippocampal atrophy	intergenic	22745009
rs11132733	4	190641170	intronic	pleyspawby	Testosterone levels	intergenic	22675492
rs11129640	3	35142511	ncRNA intronic	lawfobo	Entorhinal cortical thickness	intergenic	21116278
rs11123170	2	113978940	UTR3	PAX8	Renal function-related traits (BUN)	intronic	22797727
rs11113894	12	108841863	ncRNA intronic	cheychaw	Obesity-related traits	intergenic	23251661
rs1110183	9	38456365	intronic	LOC340501	Hypertension	intergenic	22384028
rs11078927	17	38064405	UTR5	GSDMB	Asthma	intronic	21804549
rs11078697	17	7469229	UTR3	SENP3	IgM levels	ncRNA intronic	23118916
rs11074889	16	10633165	ncRNA exonic	fawru	Attention deficit hyperactivity disorder	intronic	20732626
rs11071720	15	63341996	ncRNA exonic	garstaw	Mean platelet volume	intronic	19820697
rs11070098	13	95505262	ncRNA intronic	swonor	Obesity-related traits	intergenic	23251661

rs1106766	12	57809456	UTR5	merno	Urate levels	intergenic	20884846
rs11066453	12	113365621	ncRNA intronic	sehere	Gamma glutamyl transpeptidase	intergenic	21909109
rs11059374	12	128304418	ncRNA intronic	kuhure	Response to amphetamines	intergenic	22952603
rs11052552	12	9855958	promoter	kohire	Type 1 diabetes	intergenic	17554300
rs11036238	11	5225635	promoter	smufee	Malaria	intergenic	19465909
rs11023787	11	15952294	ncRNA exonic	kiriro	Wrist bone mass	intergenic	21104366
rs11021499	11	95992129	exonic	muslyby	Waist-to-hip circumference ratio (interaction)	intronic	23192594
rs11009175	10	33294775	ncRNA intronic	roymo	Depression (quantitative trait)	intergenic	20800221
rs11001819	10	78315224	ncRNA exonic	janawby	Pulmonary function;Pulmonary function (interaction)	intronic	21946350, 23284291
rs10995356	10	64655913	intronic	EGR2	Temperament	intergenic	22832960
rs10993994	10	51549496	intronic	LOC10431,MSMBandNCOA4	Prostate-specific antigen levels;Prostate cancer	upstream	21743057, 21160077, 20676098, 18264097, 18264096, 23269536
rs10988449	9	132370360	UTR5	METTL11A	Response to antidepressant treatment	intergenic	22041458
rs10984561	9	122258577	intronic	neyplerbu	Pulmonary function decline	intergenic	22424883
rs10961577	9	14562314	intronic	ZDHHC21	Visceral adipose tissue adjusted for BMI	intergenic	22589738
rs10957125	8	61194170	exonic	forwoby	Response to amphetamines	upstream	22952603
rs10953541	7	107244545	ncRNA exonic	slarskerby	Coronary heart disease	intronic	21378988
rs10948172	6	44777691	ncRNA intronic	farglawby	Osteoarthritis	intergenic	22763110
rs10947055	6	30093364	UTR3	harara	Cardiac hypertrophy	intergenic	21348951
rs10945919	6	164186677	intronic	sinire	Response to TNF antagonist treatment	intergenic	18615156
rs10937275	3	186650790	UTR5	chonabu	Drug-induced	intronic	19483685

					liver injury (flucloxacillin)		
rs10936797	3	174194243	intronic	plawskawby	Obesity-related traits	intergenic	23251661
rs10936632	3	170130102	ncRNA intronic	doyjoybu	Prostate cancer	intergenic	21743467
rs10929808	2	12568996	ncRNA intronic	smeydar	Bipolar disorder and schizophrenia	intergenic	20889312
rs1092913	5	10467702	intronic	ROPN1L	Breast cancer	intergenic	21424380
rs10916025	1	226855889	ncRNA exonic	neebo	Subcutaneous adipose tissue	intronic	22589738
rs10915864	1	225901006	ncRNA intronic	keeler,leebo	Obesity-related traits	intergenic	23251661
rs10905868	10	10974256	intronic	CELF2	Obesity-related traits	intergenic	23251661
rs10905099	10	7089016	ncRNA intronic	monaro	Orofacial clefts	intergenic	22419666
rs10900020	10	44827197	intronic	CXCL12	Schizophrenia	intergenic	23212062
rs10899489	11	78095373	exonic	ZNF75CP	Menarche (age at onset)	intronic	21102462
rs10897449	11	63592621	UTR5	C11orf84	Electroencephalographic traits in alcoholism	intronic	22554406
rs10888935	1	56060951	intronic	yusimo	Inflammatory biomarkers	intergenic	22228203
rs10883365	10	101287764	ncRNA exonic	flymy,terpeeby	Crohn's disease	intergenic	17554300, 17554261
rs10876864	12	56401085	promoter	IKZF4	Vitiligo	intergenic	22951725
rs10876550	12	54712308	intronic	COPZ1	Mean platelet volume	intergenic	22139419
rs10876432	12	53731891	intronic	SP7	Bone mineral density (spine)	intergenic	19079262
rs10871290	16	74472696	intronic	GLG1	Breast cancer	intergenic	18463975
rs10866713	5	158918894	ncRNA intronic	smarjoy	Multiple sclerosis	intergenic	22190364
rs10865035	2	100835734	intronic	LOC150577	Rheumatoid arthritis	intergenic	20453842
rs10858945	12	90443482	intronic	monare	Optic disc size (cup)	intergenic	20395239
rs10852932	17	2143460	UTR5	SMG6	Aortic root size	intronic	19584346
rs10849605	12	1064438	intronic	RAD52	Lung cancer	intergenic	22899653
rs10848704	12	2882544	intronic	bynaw	Quantitative traits	intergenic	19197348
rs1084651	6	161089817	intronic	LPA	HDL cholesterol	intergenic	20686565
rs10841496	12	20521654	intronic	mehure	Male infertility	upstream	19478329
rs10841397	12	20063968	intronic	reegyby	Response to amphetamines	intergenic	22952603

rs10841287	12	19779793	ncRNA intronic	shoyzeeby	Obesity-related traits	intergenic	23251661
rs10838801	11	48098280	UTR3	flawfloybu	Height	intronic	20881960
rs10824026	10	75421208	ncRNA intronic	warmoby	Atrial fibrillation	intergenic	22544366
rs10805321	4	13914373	ncRNA intronic	LOC391636	Response to antipsychotic therapy (extrapyramidal side effects)	intergenic	21990027
rs10797432	1	2501338	ncRNA intronic	geyvoybo	Ulcerative colitis	intergenic	23128233
rs10792665	11	82649768	intronic	C11orf82andRPL7AP54	Obesity-related traits	intergenic	23251661
rs10776733	1	112107669	intronic	RAP1A	Obesity-related traits	intergenic	23251661
rs10774214	12	4368352	intronic	moynee	Colorectal cancer	intergenic	23263487
rs10771515	12	9463806	exonic	LOC642846	Obesity-related traits	ncRNA exonic	23251661
rs10761779	10	65274927	intronic	soymaby	Liver enzyme levels	intergenic	18940312
rs10758161	9	32755028	ncRNA intronic	LOC100288596	Immune response to anthrax vaccine;Immune response to anthrax vaccine	intergenic	22658931
rs10744304	12	127813741	ncRNA intronic	skorswee	Depression (quantitative trait)	intergenic	20800221
rs10743889	12	10521389	intronic	herare	Obesity-related traits	intergenic	23251661
rs10738760	9	2691186	intronic	norrorby	Vascular endothelial growth factor levels	intergenic	21757650
rs1055144	7	25871109	ncRNA exonic	porblar	Waist-hip ratio	intergenic	20935629
rs1055129	17	73872948	UTR5;UTR3	natima;TRIM47	White matter hyperintensity burden	intronic	21681796
rs1052483	2	219934348	ncRNA exonic	wulu	Height	intergenic	18391951
rs10521222	16	51158710	intronic	vorglu	C-reactive protein	intergenic	21300955
rs10519131	15	62001132	ncRNA intronic	vorkley	Parkinson's disease	intergenic	22451204

rs10516541	4	108115222	intronic	DKK2	Mean forced vital capacity from 2 exams	intergenic	17903307
rs10515148	5	71716928	intronic	ZNF366andMRPS27	Hip geometry	intergenic	17903296
rs10514995	5	65739439	intronic	FLJ46010	RR interval (heart rate)	intergenic	20031603
rs10514688	3	34962669	ncRNA intronic	lawfobo	Tonometry	intergenic	17903302
rs10513789	3	182760073	UTR3	MCCC1	Parkinson's disease	intronic	21738487
rs10513025	5	9623622	ncRNA exonic	gaskeybu	Autism	intergenic	19812673
rs10510634	3	30346968	intronic	flawfybo	Fasting plasma glucose	intergenic	17903298
rs10510138	10	126438019	intronic	METTL10andFAM53B	Obesity-related traits	intergenic	23251661
rs10509540	10	90023033	intronic	RNLS,nehoro	Type 1 diabetes	intergenic	19430480
rs10507577	13	53968091	ncRNA intronic	nemeri	Select biomarker traits	intergenic	17903293
rs10506821	12	80496923	exonic	RPL26P32	Hip geometry	intergenic	17903296
rs10505477	8	128407443	ncRNA intronic	LOC727677	Colorectal cancer	intergenic	17618283
rs10501320	11	47293799	UTR5	MADD	Proinsulin levels	intronic	21873549
rs10500569	16	72756101	intronic	wateri	Metabolite levels	intergenic	22916037
rs10499504	7	17561583	ncRNA intronic	marky	Response to antipsychotic treatment	intergenic	20195266
rs10496166	2	69063909	ncRNA exonic	stordor	RR interval (heart rate)	intergenic	20031603
rs10493340	1	63591129	ncRNA intronic	sorbor	Blood pressure	intergenic	17903302
rs10492681	13	40807483	ncRNA intronic	serare	Select biomarker traits	intergenic	17903293
rs10492096	12	6580582	intronic	TAPBPL	Hip geometry	upstream	17903296
rs10490113	2	59499347	ncRNA intronic	miho	Breast cancer	intergenic	17903305
rs10489087	4	13794416	ncRNA intronic	LOC391636	Hemostatic factors and hematological phenotypes	intergenic	17903294
rs10486201	7	7938596	intronic	GLCCI1	Obesity-related traits	intergenic	23251661
rs10485165	6	89112817	intronic	sposmawby	Select biomarker traits	intergenic	17903293
rs10484761	6	40802261	ncRNA intronic	hasiya	Esophageal cancer	intergenic	21642993

rs10484554	6	31274555	ncRNA intronic	rosiya	Psoriasis;AIDS progression	intergenic	20953190, 19115949, 18369459
rs10484434	6	26031613	ncRNA intronic	smujar	HIV-1 viral setpoint	downstream	22174851
rs10484128	14	98642572	ncRNA intronic	vydyby	Hemostatic factors and hematological phenotypes	intergenic	17903294
rs10466868	12	131939920	ncRNA intronic	yuhere	Protein quantitative trait loci	intergenic	18464913
rs10458787	10	4655565	ncRNA intronic	LOC100216001	Body mass index	intergenic	20397748
rs1044826	3	139073011	ncRNA exonic	sorimo	Obesity-related traits	intronic	23251661
rs10429924	1	244390564	promoter	remaha	Schizophrenia	intergenic	22885689
rs10423754	19	54604822	intronic	OSCAR	Response to taxane treatment (docetaxel)	upstream	23006423
rs10411161	19	52372976	intronic	ZNF649andZNF577	Breast cancer	intergenic	21424380
rs10404998	19	22608374	ncRNA intronic	blerwy	Dental caries	intergenic	23259602
rs10401969	19	19407718	UTR3	SF4	Triglycerides;LDL cholesterol;"Cholesterol, total"	intronic	20864672, 20686565, 19060906
rs1037757	18	56752054	UTR3	komiri	Alzheimer's disease (age of onset)	intergenic	22005931
rs1035942	19	7199803	ncRNA exonic	noywa	Urate levels	intronic	23263486
rs1027643	5	91893792	ncRNA intronic	blawgey	Wilms tumor	intergenic	22544364
rs10273639	7	142456928	intronic	TRBV	Pancreatitis	upstream	23143602
rs10266483	7	63733331	intronic	ZNF727	Response to statin therapy	intergenic	20339536
rs1024161	2	204721752	intronic	soystuby	Graves' disease;Alopecia areata	intergenic	21841780, 20596022
rs1023252	1	11899033	UTR3	CLCN6	Natriuretic peptide levels	intronic	21273288
rs10229603	7	112628373	ncRNA intronic	flarkaw	Attention deficit hyperactivity disorder and conduct disorder	intergenic	18951430
rs10227331	7	157294938	ncRNA	worjoy	Inattentive	intergenic	18821565

			intronic		symptoms		
rs10219495	12	92890520	promoter	swyjuby	Sudden cardiac arrest	intergenic	21658281
rs10211524	2	65208074	intronic	tamomo	Metabolite levels	intergenic	22916037
rs1020410	2	176784138	intronic	KIAA1715	Obesity-related traits	intergenic	23251661
rs10187424	2	85794297	intronic	VAMP8andVAMP5	Prostate cancer	intergenic	21743467
rs1018326	2	182007800	intronic	mamemo	Ankylosing spondylitis	intergenic	20062062
rs10174126	2	235960893	UTR5	SH3BP4	Orofacial clefts	intronic	22419666
rs10172965	2	195871819	ncRNA intronic	seestuby	Temperament	intergenic	22832960
rs10170236	2	150457624	ncRNA intronic	romemo	Acute lymphoblastic leukemia (childhood)	intergenic	23007406
rs1016883	2	198881668	UTR5	mona	Ulcerative colitis	intronic	23128233
rs1016343	8	128093297	ncRNA intronic	bleyvyby,kloger	Prostate cancer	intergenic	21743057, 18264097
rs10162002	13	24042510	intronic	sporzor	Hypothyroidism	intergenic	22493691
rs1015657	17	68878487	ncRNA intronic	forto	Formal thought disorder in schizophrenia	intergenic	22648509
rs10155981	7	22756463	ncRNA intronic	yuhiya	Bilirubin levels	intergenic	22085899
rs1015213	8	52887541	ncRNA intronic	sorvawbu	Glaucoma (primary open-angle)	intergenic	22922875
rs10136766	14	106232585	intronic	IGHV	IgG levels	intergenic	23225573
rs1013063	21	35698390	intronic	MRPS6andSLC5A3andC21orf82	Body mass index (interaction)	intergenic	23192594
rs10125054	9	114798037	UTR5	sutira	Obesity-related traits	ncRNA intronic	23251661
rs10104895	8	61876044	ncRNA intronic	veegee	Pulmonary function decline	intergenic	22424883
rs10092658	8	130980472	intronic	FAM49B	Protein quantitative trait loci	intergenic	18464913
rs1009170	14	92636713	ncRNA exonic	koydaby	Dialysis-related mortality	intergenic	21546767
rs10091374	8	71386904	ncRNA intronic	ninera	Cardiac Troponin-T levels	intergenic	23247143
rs10088218	8	129543949	intronic	sluskobu	Ovarian cancer	intergenic	20852632

rs10049246	3	185686741	UTR5	LOC344887	Attention deficit hyperactivity disorder symptoms (interaction)	ncRNA intronic	18846501
rs10048158	17	64236318	intronic	APOH	β2-Glycoprotein I (β2-GPI) plasma levels	intergenic	23279374
rs10045431	5	158814533	ncRNA exonic	smargey	Crohn's disease	intergenic	20570966, 18587394
rs1004467	10	104594507	UTR3	CYP17A1	Systolic blood pressure	intronic	19430479
rs10033900	4	110659067	intronic	CFI	Age-related macular degeneration (GA);Age-related macular degeneration (CNV);Age-related macular degeneration	intergenic	22705344, 21665990, 20385826
rs10033464	4	111720761	ncRNA exonic	titime	Atrial fibrillation/atrial flutter	intergenic	17603472
rs10030601	4	150725212	intronic	LOC285423	Epilepsy (generalized)	intergenic	22949513
rs1002979	3	112497880	intronic	LOC100129297	Prostate cancer (gene x gene interaction)	intergenic	22219177
rs1000778	11	61655305	exonic	fawmuby	Sphingolipid levels	intronic	19798445
rs1000597	7	30937178	intronic	INMTandFAM188BandAQP1	Nephrolithiasis	intergenic	22396660
rs1000113	5	150240076	intronic	slowuby	Crohn's disease	intergenic	17554300

Table S5. Functional annotation of those non-RefSeq-exonic GWAS SNPs that relocated in Ensembl coding regions

GWAS SNP IDs	Associated diseases or traits	Associated genes	Associated transcripts	Associated proteins	Mutation type	GO biological process	GO cellular component	GO molecular function
rs9272535	Chronic lymphocytic leukemia	ENSG00000196735	ENST00000482745	ENSP00000436546	synonymous			
rs890835	Menopause (age at onset)	ENSG00000146083	ENST00000506378	ENSP00000425253	synonymous			zinc ion binding;
rs75825892	Obesity-related traits	ENSG00000185739	ENST00000572111	ENSP00000461179	non-synonymous	GTP catabolic process;	sarcoplasmic reticulum lumen;	GTPase activity; GTP binding;
rs7204371	Obesity-related traits	ENSG00000102977	ENST00000602850	ENSP00000473595	non-synonymous	skeletal system development; segmentation; protection from non-homologous end joining at telomere; negative regulation of telomere maintenance via telomerase; embryonic limb morphogenesis; intracellular protein transport; urogenital system development; positive regulation of telomerase activity; telomere assembly; positive regulation of single-stranded telomeric DNA binding; protein localization to chromosome, telomeric region;	nucleoplasm; nuclear telomere cap complex;	DNA binding; DNA polymerase binding;
rs61744862	Obesity-related traits	ENSG00000133030	ENST00000313485	ENSP00000317786	synonymous		cytoskeleton; cytoplasm;	actin binding; phospholipid binding;
rs4246905	Ulcerative colitis; Inflammatory bowel	ENSG00000181634	ENST00000374044	ENSP00000363156	non-synonymous	activation of NF-kappaB-inducing kinase	integral to plasma membrane;	death receptor binding; cytokine

	disease					activity; positive regulation of cytokine secretion; activation of cysteine-type endopeptidase activity involved in apoptotic process; cytokine metabolic process;		activity; tumor necrosis factor receptor binding;
rs1420101	Eosinophil counts	ENSG00000115602	ENST00000427077; ENST00000393393	ENSP00000391120; ENSP00000377052 (identical protein)	non-synonymous	negative regulation of interferon-gamma production; negative regulation of I-kappaB kinase/NF-kappaB cascade; negative regulation of T-helper 1 type immune response; cytokine-mediated signaling pathway; positive regulation of interleukin-5 production; positive regulation of chemokine secretion; positive regulation of inflammatory response; innate immune response;	extracellular space; integral to membrane; intracellular; external side of plasma membrane;	receptor signaling protein activity; interleukin-33 receptor activity; interleukin-33 binding; interleukin-1 receptor activity;
rs1023252	Natriuretic peptide levels	ENSG00000011021	ENST00000376496	ENSP00000365679	non-synonymous	transmembrane transport; chloride transport; signal transduction; cell volume homeostasis; response to mechanical stimulus;	integral to membrane; endosome membrane;	voltage-gated chloride channel activity; ATP binding; antiporter activity;

Table S6. Functional annotation of those non-RefSeq-exonic GWAS SNPs that relocated in UCSC coding regions

GWAS SNP IDs	Associated diseases or traits	Associated genes	Associated transcripts	Associated proteins	Mutation type	GO biological process	GO cellular component	GO molecular function
rs9604529	Response to tocilizumab in rheumatoid arthritis	FLJ44054	uc001vuc.3	uc001vuc.3	unknown			zinc ion binding; protein binding;
rs890835	Menopause (age at onset)	RNF44	uc003mel.1	uc003mel.1	synonymous			
rs886424	Bipolar disorder and schizophrenia	AK098012	uc003nrp.1	uc003nrp.1	unknown			
rs399604	Platelet counts	HLA-DOA	uc010jui.3	uc010jui.3	synonymous	T cell costimulation; antigen processing and presentation of exogenous peptide antigen via MHC class II; regulation of T cell differentiation; T cell receptor signaling pathway; interferon-gamma-mediated signaling pathway;	lysosomal membrane; MHC class II protein complex; integral to membrane; endosome membrane;	MHC class II receptor activity; protein binding;
rs3117035	RR interval (heart rate)	HLA-DPB2	uc003ocv.1	uc003ocv.1	stop-gain	T cell costimulation; antigen processing and presentation of peptide or	lysosomal membrane; MHC class II protein complex; integral to membrane; endosome membrane;	

						polysaccharide antigen via MHC class II; T cell receptor signaling pathway; interferon-gamma-mediated signaling pathway;	endoplasmic reticulum membrane; Golgi apparatus;	
rs2277862	Cholesterol, total	FER1L4	uc010gfg.1;	uc010gfg.1	non-synonymous		integral to membrane;	protein binding;
rs2259816	C-reactive protein; Coronary heart disease	HNF1A	uc021rez.1; uc001tzf.3; uc021rex.1; uc021rey.1	uc021rez.1; uc001tzf.3; uc021rex.1; uc021rey.1	synonymous	glucose import; embryonic limb morphogenesis; fatty acid biosynthetic process; reproductive structure development; blastocyst development; regulation of Wnt receptor signaling pathway; response to glucose stimulus; reverse cholesterol transport; protein localization; regulation of	pronucleus; cytoplasm; transcription factor complex; photoreceptor outer segment;	protein homodimerization activity; double-stranded DNA binding; transcription factor binding; RNA polymerase II core promoter proximal region sequence-specific DNA binding transcription factor activity involved in positive regulation of transcription; transcription regulatory

						pronephros size; liver development; cholesterol metabolic process; positive regulation of transcription initiation from RNA polymerase II promoter; glucose homeostasis; SMAD protein signal transduction; response to oxidative stress; positive regulation of transcription from RNA polymerase II promoter; endocrine pancreas development; bile acid and bile salt transport; placenta development; negative		region DNA binding; protein heterodimerization activity; sequence-specific DNA binding;

						regulation of transcription from RNA polymerase II promoter; bone resorption; heme biosynthetic process; chromatin remodeling; regulation of insulin secretion; paraxial mesoderm formation; bile acid biosynthetic process; renal glucose absorption; fatty acid transport;		
rs2070488	Electrocardiographic conduction measures	XYLB	uc003cid.1	uc003cid.1	non-synonymous	D-xylose metabolic process; phosphorylation; xylulose catabolic process; generation of precursor metabolites and energy;		xylulokinase activity; ATP binding;

Table S7. Functional annotation of those non-RefSeq-exonic GWAS SNPs that relocated in AceView coding regions

GWAS SNP IDs	Associated diseases or traits	Associated genes	Associated transcripts	Associated proteins	Mutation type	GO biological process	GO cellular component	GO molecular function
rs35079168	Intelligence	latybu	latybu.aAug10-unspliced	latybu.aAug10-unspliced	unknown			
rs11136000	Alzheimer's disease	smawjarby	smawjarby.aAug10-unspliced	smawjarby.aAug10-unspliced	unknown	transcription, DNA-dependent; regulation of dendritic spine morphogenesis; regulation of synapse assembly;	membrane; membrane fraction; actin cytoskeleton; cytosol; postsynaptic density;	protein kinase C binding; actinin binding; actin binding;
rs9608102	Immune response to smallpox vaccine (IL-6)	BCR	BCR.hAug10	BCR.hAug10	unknown	negative regulation of inflammatory response; negative regulation of cell migration; response to lipopolysaccharide; negative regulation of neutrophil degranulation; regulation of Rho protein signal transduction; positive regulation of phagocytosis; actin cytoskeleton organization; inner ear morphogenesis; brain development	cytosol; plasma membrane;	protein binding; Rac GTPase activator activity; phospholipid binding; protein serine/threonine kinase activity; Rho guanyl-nucleotide exchange factor activity; ATP binding;

						; protein phosphorylation; regulation of cell cycle; neuromuscular process controlling balance; positive regulation of GTPase activity; serine family amino acid metabolic process;		
rs9604529	Response to tocilizumab in rheumatoid arthritis	FLJ44054	FLJ44054.bAug10; FLJ44054.aAug10	FLJ44054.bAug10; FLJ44054.aAug10	unknown			
rs9442235	Cognitive performance	teymorbu	teymorbu.aAug10-unspliced	teymorbu.aAug10-unspliced	unknown			
rs9295536	Neuroblastoma	klojar	klojar.aAug10-unspliced	klojar.aAug10-unspliced	nonsynonymous			
rs890835	Menopause (age at onset)	RNF44	RNF44.cAug10	RNF44.cAug10	synonymous			zinc ion binding;
rs8176704	Coagulation factor levels	foytoyby	foytoyby.aAug10-unspliced	foytoyby.aAug10-unspliced	nonsynonymous			
rs805303	Systolic blood pressure;Hypertension;Diastolic blood pressure	BAT3	BAT3.vaAug10	BAT3.vaAug10	synonymous	transport; lung development; synaptonemal complex assembly; tail-anchored membrane protein insertion into ER membrane; regulation of cell proliferation; kidney	BAT3 complex; nucleus;	Hsp70 protein binding; proteasome binding; polyubiquitin binding; ribosome binding;

						development; antigen processing and presentation of peptide antigen via MHC class I; spermatogenesis; protein stabilization; DNA damage response, signal transduction by p53 class mediator resulting in induction of apoptosis; internal peptidyl-lysine acetylation; brain development; negative regulation of proteasomal ubiquitin-dependent protein catabolic process; embryo development; cell differentiation; intrinsic apoptotic signaling pathway in response to endoplasmic reticulum stress; chromatin		

						modification;		
rs769449	C-reactive protein	APOE	APOE.fAug10	APOE.fAug10	unknown			
rs7571971	Progressive supranuclear palsy	EIF2AK3	EIF2AK3.bAug10	EIF2AK3.bAug10	unknown	positive regulation of protein binding; bone mineralization; negative regulation of translational initiation in response to stress; chondrocyte development; endoplasmic reticulum organization; positive regulation of signal transduction; insulin secretion; protein homooligomerization; ER overload response; fat cell differentiation; activation of cysteine-type endopeptidase activity involved in apoptotic process; endocrine pancreas development; regulation of fatty acid	integral to membrane; endoplasmic reticulum membrane; ribosome;	translation initiation factor activity; eukaryotic translation initiation factor 2alpha kinase activity; identical protein binding; ATP binding; protein phosphatase binding;

						metabolic process; insulin-like growth factor receptor signaling pathway; protein autophosphorylation; SREBP signaling pathway; activation of signaling protein activity involved in unfolded protein response; calcium-mediated signaling; virus-infected cell apoptotic process; negative regulation of myelination; lactation; serine family amino acid metabolic process;		
rs746630	Obesity-related traits	fava	fava.bAug10; fava.aAug10	fava.bAug10; fava.aAug10	synonymous			
rs735396	N-glycan levels	C12orf43	C12orf43.iAug10-unspliced	C12orf43.iAug10-unspliced	nonsynonymous			
rs7204371	Obesity-related traits	ACD	ACD.dAug10; ACD.kAug10; ACD.iAug10	ACD.dAug10; ACD.kAug10; ACD.iAug10	synonymous	skeletal system development; segmentation; protection from	nucleoplasm; nuclear telomere cap complex;	DNA binding; DNA polymerase binding;

						non-homologous end joining at telomere; negative regulation of telomere maintenance via telomerase; embryonic limb morphogenesis; intracellular protein transport; urogenital system development; positive regulation of telomerase activity; telomere assembly; positive regulation of single-stranded telomeric DNA binding; protein localization to chromosome, telomeric region;		
rs7105056	Immune reponse to smallpox (secreted IL-12p40)	SDHD_	SDHD_.lAug10	SDHD_.lAug10	nonsynonymous	retinal metabolic process; oxidation-reduction process; carotene catabolic process; retinoic acid metabolic	mitochondrion;	metal ion binding; oxidoreductase activity, acting on single donors with incorporation of molecular oxygen,

						process; regulation of mitochondrial membrane potential; regulation of reactive oxygen species metabolic process;		incorporation of two atoms of oxygen;
rs7090512	Multiple sclerosis	kakloybu	kakloybu.aAug10-unspliced	kakloybu.aAug10-unspliced	nonsynonymous			
rs7044529	Central corneal thickness;Corneal structure	mortarby	mortarby.aAug10-unspliced	mortarby.aAug10-unspliced	nonsynonymous			
rs6904029	Vitiligo	HCG9	HCG9.bAug10-unspliced; HCG9.aAug10	HCG9.bAug10-unspliced; HCG9.aAug10	nonsynonymous			
rs6586282	Homocysteine levels	CBS	CBS.qAug10-unspliced	CBS.qAug10-unspliced	unknown			
rs61744862	Obesity-related traits	MPRIP	MPRIP.aAug10	MPRIP.aAug10	nonsynonymous		cytoskeleton; cytoplasm;	actin binding; phospholipid binding;
rs4985167	Phospholipid levels (plasma)	LOC728138	LOC728138.bAug10-unspliced; LOC728138.aAug10	LOC728138.bAug10-unspliced; LOC728138.aAug10	nonsynonymous		integral to membrane;	
rs4815617	Asthma	C20orf29and MAVS	C20orf29and MAVS.iAug10	C20orf29and MAVS.iAug10	unknown			
rs4785763	Melanoma	AFG3L1	AFG3L1.cAug10	AFG3L1.cAug10	synonymous	mitochondrial protein processing; mitochondrial fusion; proteolysis; protein catabolic process; cristae formation;	mitochondrion; integral to membrane;	nucleoside-triphosphatase activity; zinc ion binding; metalloendopeptidase activity; ATP binding;
rs4773144	Coronary heart disease	slozoy	slozoy.aAug10-unspliced	slozoy.aAug10-unspliced	nonsynonymous			

rs4670779	Bone mineral density (spine)	STRAP.1	STRAP.1.aAug10-unspliced	STRAP.1.aAug10-unspliced	nonsynonymous	negative regulation of transforming growth factor beta receptor signaling pathway; RNA splicing; negative regulation of transcription from RNA polymerase II promoter; mRNA processing;	cell junction; spliceosomal complex; mitochondrion; plasma membrane;	receptor binding; kinase activity;
rs4434872	Conduct disorder (symptom count)	stoyrubo	stoyrubo.aAug10	stoyrubo.aAug10	unknown			
rs425215	Common traits (Other)	stato	stato.aAug10-unspliced	stato.aAug10-unspliced	unknown			
rs4246905	Ulcerative colitis;Inflammatory bowel disease	TNFSF15	TNFSF15.cAug10-unspliced	TNFSF15.cAug10-unspliced	nonsynonymous	activation of NF-kappaB-inducing kinase activity; positive regulation of cytokine secretion; immune response; activation of cysteine-type endopeptidase activity involved in apoptotic process; cytokine metabolic process;	extracellular space; integral to plasma membrane;	death receptor binding; cytokine activity; tumor necrosis factor receptor binding;
rs415890	Crohn's disease	sheysnaby	sheysnaby.aAug10-unspliced	sheysnaby.aAug10-unspliced	nonsynonymous			

rs399604	Platelet counts	HLA-DOA	HLA-DOA.dAug10; HLA-DOA.eAug10	HLA-DOA.dAug10; HLA-DOA.eAug10	synonymous	T cell costimulation; antigen processing and presentation of exogenous peptide antigen via MHC class II; regulation of T cell differentiation; T cell receptor signaling pathway; interferon-gamma-mediated signaling pathway;	lysosomal membrane; MHC class II protein complex; integral to membrane; endosome membrane;	MHC class II receptor activity;
rs3809346	Coronary artery calcification	slozoy	slozoy.aAug10-unspliced	slozoy.aAug10-unspliced	nonsynonymous			
rs3803662	Breast cancer (male);Breast cancer;Breast cancer (male)	LOC643714	LOC643714.cAug10	LOC643714.cAug10	unknown			
rs3795578	Response to acetaminophen (hepatotoxicity)	mimo	mimo.aAug10-unspliced	mimo.aAug10-unspliced	unknown			
rs3764913	Metabolite levels	ACADL	ACADL.eAug10	ACADL.eAug10	synonymous	long-chain fatty acid catabolic process; temperature homeostasis; negative regulation of fatty acid oxidation; regulation of	mitochondrial membrane; mitochondrial matrix;	fatty-acyl-CoA binding; palmitoyl-CoA oxidase activity; long-chain-acyl-CoA dehydrogenase activity; flavin adenine

						cholesterol metabolic process; fatty acid beta-oxidation using acyl-CoA dehydrogenase; carnitine catabolic process; negative regulation of fatty acid biosynthetic process; carnitine metabolic process, CoA-linked; protein homotetramerization; acyl-CoA metabolic process; electron transport;		dinucleotide binding;
rs3744028	White matter hyperintensity burden	TRIM65	TRIM65.dAug10	TRIM65.dAug10	nonsynonymous		intracellular;	zinc ion binding;
rs361433	Capecitabine sensitivity	ritemu	ritemu.aAug10	ritemu.aAug10	unknown			
rs3117035	RR interval (heart rate)	HLA-DPB2	HLA-DPB2.aAug10	HLA-DPB2.aAug10	stopgain	T cell costimulation; antigen processing and presentation of peptide or polysaccharide antigen via MHC class II; T cell receptor signaling pathway; interferon-ga	lysosomal membrane; MHC class II protein complex; integral to membrane; endosome membrane; endoplasmic reticulum membrane; Golgi apparatus;	

						mma-mediated signaling pathway;		
rs2981579	Breast cancer	myraby	myraby.aAug10-unspliced	myraby.aAug10-unspliced	synonymous			
rs2967951	Body mass index	ROPN1L	ROPN1L.eAug10-unspliced	ROPN1L.eAug10-unspliced	nonsynonymous	ciliary or flagellar motility; signal transduction; regulation of protein kinase activity;	cytoplasm; motile cilium;	protein binding; cAMP-dependent protein kinase regulator activity;
rs28493229	Kawasaki disease	ITPKC	ITPKC.bAug10-unspliced	ITPKC.bAug10-unspliced	unknown		cytoplasm; nucleus;	calmodulin binding; inositol-1,4,5-trisphosphate 3-kinase activity; ATP binding;
rs274546	Height	glarstorby	glarstorby.aAug10-unspliced	glarstorby.aAug10-unspliced	synonymous			
rs2730245	Height	smarkee	smarkee.aAug10-unspliced	smarkee.aAug10-unspliced	synonymous			
rs2677744	Attention deficit hyperactivity disorder	MAN2A2	MAN2A2.uAug10	MAN2A2.uAug10	unknown	mannose metabolic process; protein N-linked glycosylation via asparagine;	integral to membrane; Golgi membrane;	zinc ion binding; hydrolase activity, hydrolyzing N-glycosyl compounds; mannosyl-oligosaccharide 1,3-1,6-alpha-mannosidase activity; carbohydrate binding;
rs2395029	Psoriasis;HIV-1 control;Drug-induced liver injury (flucloxacillin);AIDS progression	MICAandHCP5	MICAandHCP5.kAug10-unspliced	MICAandHCP5.kAug10-unspliced	nonsynonymous	defense response;		
rs2382817	Inflammatory bowel	TMBIM1	TMBIM1.oAug10-unspliced	TMBIM1.oAug10-unspliced	synonymous			

	disease							
rs2303369	Menopause (age at onset)	FNDC4	FNDC4.cAug10	FNDC4.cAug10	synonymous		integral to membrane; endoplasmic reticulum; plasma membrane;	
rs2286276	Triglycerides	TBL2	TBL2.rAug10	TBL2.rAug10	synonymous			
rs2284746	Pulmonary function;Height;Pulmonary function (interaction)	jorplo	jorplo.aAug10-unspliced	jorplo.aAug10-unspliced	unknown			
rs2278170	Amyotrophic lateral sclerosis	OR52K3P	OR52K3P.aAug10-unspliced	OR52K3P.aAug10-unspliced	unknown	detection of chemical stimulus involved in sensory perception of smell; G-protein coupled receptor signaling pathway, coupled to cyclic nucleotide second messenger;	plasma membrane; integral to membrane;	G-protein coupled receptor activity; olfactory receptor activity;
rs2277862	Cholesterol, total	FER1L4	FER1L4.bAug10	FER1L4.bAug10	nonsynonymous		integral to membrane;	
rs2259816	C-reactive protein;Coronary heart disease	HNF1A	HNF1A.cAug10	HNF1A.cAug10	synonymous	glucose import; embryonic limb morphogenesis; fatty acid biosynthetic process; negative regulation of transcription, DNA-dependent; reproductive	pronucleus; cytoplasm; transcription factor complex; photoreceptor outer segment;	protein homodimerization activity; double-stranded DNA binding; RNA polymerase II core promoter proximal region sequence-specific DNA binding

						structure development; blastocyst development; regulation of Wnt receptor signaling pathway; response to glucose stimulus; reverse cholesterol transport; protein localization; regulation of pronephros size; liver development; cholesterol metabolic process; positive regulation of transcription initiation from RNA polymerase II promoter; glucose homeostasis; SMAD protein signal transduction; response to oxidative stress; positive regulation of transcription from RNA polymerase II promoter; endocrine pancreas development		transcription factor activity involved in positive regulation of transcription; transcription regulatory region DNA binding; protein heterodimerization activity; sequence-specific DNA binding;

						; bile acid and bile salt transport; placenta development; bone resorption; heme biosynthetic process; chromatin remodeling; regulation of insulin secretion; paraxial mesoderm formation; bile acid biosynthetic process; renal glucose absorption; fatty acid transport;		
rs2255221	HIV-1 control	MICAandHCP5	MICAandHCP5.kAug10-unspliced	MICAandHCP5.kAug10-unspliced	nonsynonymous	defense response;		
rs2239557	Common traits (Other)	LIN52	LIN52.eAug10-unspliced	LIN52.eAug10-unspliced	unknown			
rs2102808	Parkinson's disease	skawsterby	skawsterby.aAug10-unspliced	skawsterby.aAug10-unspliced	nonsynonymous	RNA-dependent DNA replication; proteolysis; DNA integration; regulation of RNA metabolic process;		ribonuclease H activity; RNA-directed DNA polymerase activity; RNA binding; aspartic-type endopeptidase activity;
rs2079742	Urate levels	rekiri	rekiri.aAug10-unspliced	rekiri.aAug10-unspliced	synonymous			
rs2072590	Ovarian cancer	LOC401022	LOC401022.aAug10	LOC401022.aAug10	nonsynonymous			
rs204993	Asthma	loyseeby	loyseeby.aAug10-unspliced	loyseeby.aAug10-unspliced	synonymous			
rs1981483	Disc	NHLRC4and	NHLRC4andP	NHLRC4andP	nonsynonym			

	degeneration (lumbar)	PIGQ	IGQ.nAug10-unspliced	IGQ.nAug10-unspliced	ous			
rs16909318	Visceral fat	gluvyby	gluvyby.aAug10	gluvyby.aAug10	synonymous			
rs1564282	Parkinson's disease (familial)	GAK	GAK.sAug10-unspliced	GAK.sAug10-unspliced	synonymous			
rs1420101	Eosinophil counts	IL1RL1andIL18R1	IL1RL1andIL18R1.hAug10	IL1RL1andIL18R1.hAug10	nonsynonymous	negative regulation of interferon-gamma production; positive regulation of macrophage activation; negative regulation of I-kappaB kinase/NF-kappaB cascade; negative regulation of T-helper 1 type immune response; cytokine-mediated signaling pathway; positive regulation of interleukin-5 production; positive regulation of chemokine secretion; positive regulation of inflammatory response; innate immune response;	extracellular space; intracellular; external side of plasma membrane; interleukin-1 receptor complex;	receptor signaling protein activity; interleukin-33 receptor activity; interleukin-33 binding; interleukin-1 receptor activity;
rs13428812	Crohn's disease	blarjarbu	blarjarbu.aAug10-unspliced	blarjarbu.aAug10-unspliced	nonsynonymous			

rs13208776	Vitiligo	ferflarby	ferflarby.aAug10	ferflarby.aAug10	unknown			
rs13194984	Iron status biomarkers	BTN1A1	BTN1A1.bAug10-unspliced	BTN1A1.bAug10-unspliced	unknown			
rs12726330	Parkinson's disease	RAG1AP1	RAG1AP1.aAug10; RAG1AP1.qAug10	RAG1AP1.aAug10; RAG1AP1.qAug10	unknown	positive regulation of gene expression, epigenetic; glucoside transport; DNA recombination;	Golgi membrane; plasma membrane; integral to membrane; nucleus;	glucoside transmembrane transporter activity;
rs12447804	Pulmonary function;Pulmonary function (interaction)	veyseybo	veyseybo.aAug10-unspliced	veyseybo.aAug10-unspliced	unknown			
rs12356193	Uric acid levels	SLC16A9	SLC16A9.fAug10	SLC16A9.fAug10	synonymous	urate metabolic process;	membrane;	
rs1208285	Infantile hypertrophic pyloric stenosis	MGC34034	MGC34034.aAug10	MGC34034.aAug10	nonsynonymous			
rs12073504	Obesity-related traits	PANK4	PANK4.fAug10	PANK4.fAug10	nonsynonymous			
rs12049330	Major depressive disorder	ATXN7L2	ATXN7L2.hAug10-unspliced	ATXN7L2.hAug10-unspliced	nonsynonymous			
rs11986414	Gaucher disease severity	verlu	verlu.aAug10-unspliced	verlu.aAug10-unspliced	synonymous			
rs11978267	Acute lymphoblastic leukemia (childhood)	pyblorby	pyblorby.aAug10-unspliced	pyblorby.aAug10-unspliced	nonsynonymous			
rs11959928	Chronic kidney disease	rorplerby	rorplerby.aAug10-unspliced	rorplerby.aAug10-unspliced	nonsynonymous			
rs11858836	Chronic obstructive pulmonary disease	IREB2	IREB2.dAug10	IREB2.dAug10	unknown	regulation of translation; erythrocyte homeostasis; protoporphyrinogen IX biosynthetic	cytosol; mitochondrion;	iron-responsive element binding; metal ion binding; 4 iron, 4 sulfur cluster

						process; osteoclast differentiation; cellular iron ion homeostasis; iron ion transport; post-embryonic development; intestinal absorption;		binding; protein binding;
rs11842874	Osteoarthritis	goyzarby	goyzarby.aAug10-unspliced	goyzarby.aAug10-unspliced	synonymous			
rs11782819	Alzheimer's disease	rozawby	rozawby.bAug10	rozawby.bAug10	unknown			
rs11773845	PR interval	voyju	voyju.aAug10-unspliced	voyju.aAug10-unspliced	synonymous			
rs11710077	Ventricular conduction	cheefarbo	cheefarbo.aAug10-unspliced	cheefarbo.aAug10-unspliced	synonymous			
rs11646411	Attention deficit hyperactivity disorder	taseri	taseri.aAug10-unspliced	taseri.aAug10-unspliced	unknown			
rs11618202	MRI atrophy measures	fawchy	fawchy.aAug10-unspliced	fawchy.aAug10-unspliced	unknown			
rs11612508	Inflammatory bowel disease	spoyfoyby	spoyfoyby.aAug10-unspliced	spoyfoyby.aAug10-unspliced	stoploss			
rs11593576	Vitiligo	snawnawby	snawnawby.aAug10-unspliced	snawnawby.aAug10-unspliced	unknown			
rs11574637	Systemic lupus erythematosus	ITGAX	ITGAX.fAug10	ITGAX.fAug10	nonsynonymous	leukocyte migration; defense response to virus; organ morphogenesis; integrin-mediated signaling pathway; blood coagulation; cell adhesion;	external side of plasma membrane; integrin complex;	receptor activity; protein binding;

rs1122608	Myocardial infarction (early onset);Coronary heart disease	chyflor	chyflor.aAug10-unspliced	chyflor.aAug10-unspliced	synonymous			
rs11021499	Waist-to-hip circumference ratio (interaction)	muslyby	muslyby.aAug10-unspliced	muslyby.aAug10-unspliced	nonsynonymous			
rs10957125	Response to amphetamines	forwoby	forwoby.aAug10-unspliced	forwoby.aAug10-unspliced	unknown			
rs10899489	Menarche (age at onset)	ZNF75CP	ZNF75CP.aAug10-unspliced	ZNF75CP.aAug10-unspliced	synonymous	viral reproduction; regulation of transcription, DNA-dependent;	transcription factor complex;	sequence-specific DNA binding transcription factor activity; DNA binding; zinc ion binding;
rs10771515	Obesity-related traits	LOC642846	LOC642846.dAug10; LOC642846.fAug10	LOC642846.dAug10; LOC642846.fAug10	nonsynonymous	DNA duplex unwinding; positive regulation of cell proliferation; activation of signaling protein activity involved in unfolded protein response; S phase of mitotic cell cycle; mitotic sister chromatid segregation; sister chromatid cohesion; G2/M transition of mitotic cell cycle;	nucleoplasm; nucleolus; midbody; nuclear chromatin; spindle pole; replication fork;	protein binding; DNA binding; RNA binding; ATP-dependent DNA helicase activity; ATP binding;

						interspecies interaction between organisms;		
rs10506821	Hip geometry	RPL26P32	RPL26P32.aAug10-unspliced	RPL26P32.aAug10-unspliced	unknown	translational initiation; viral transcription; rRNA processing; ribosomal large subunit biogenesis; translational elongation; translational termination; SRP-dependent cotranslational protein targeting to membrane; nuclear-transcribed mRNA catabolic process, nonsense-mediated decay;	cytosolic large ribosomal subunit;	RNA binding; structural constituent of ribosome; protein binding;
rs1000778	Sphingolipid levels	fawmuby	fawmuby.aAug10-unspliced	fawmuby.aAug10-unspliced	synonymous			
